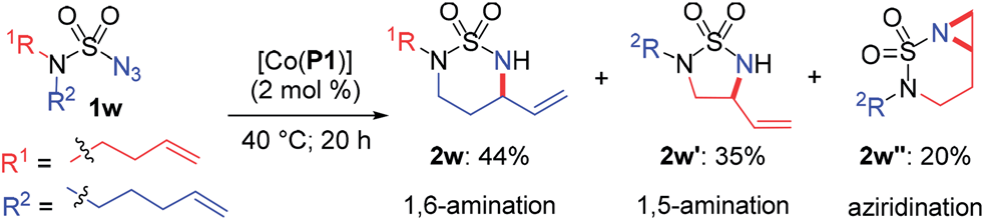# Intramolecular 1,5-C(sp^3^)–H radical amination *via* Co(ii)-based metalloradical catalysis for five-membered cyclic sulfamides†
†Electronic supplementary information (ESI) available. CCDC 1480993–1480996. For ESI and crystallographic data in CIF or other electronic format see DOI: 10.1039/c6sc02231f


**DOI:** 10.1039/c6sc02231f

**Published:** 2016-07-28

**Authors:** Hongjian Lu, Kai Lang, Huiling Jiang, Lukasz Wojtas, X. Peter Zhang

**Affiliations:** a Department of Chemistry , Merkert Chemistry Center , Boston College , Chestnut Hill , Massachusetts 02467 , USA . Email: peter.zhang@bc.edu; b Department of Chemistry , University of South Florida , Tampa , FL 33620 , USA; c The Institute of Chemistry & Biomedical Sciences , Nanjing University , Nanjing , 210093 , P. R. China . Email: hongjianlu@nju.edu.cn

## Abstract

Co(ii)-based metalloradical catalysis (MRC) proves effective for intramolecular 1,5-C–H amination of sulfamoyl azides under neutral and nonoxidative conditions, providing a straightforward approach to access strained 5-membered cyclic sulfamides with nitrogen gas as the only byproduct. The metalloradical amination system is applicable to different types of C(sp^3^)–H bonds and has a high degree of functional group tolerance. Additional features of the Co(ii)-catalyzed 1,5-C–H amination include excellent chemoselectivity toward allylic and propargylic C–H bonds. The unique reactivity and selectivity profile of the Co(ii)-catalyzed 1,5-C–H amination is attributed to the underlying radical mechanism of MRC.

## Abstract 2

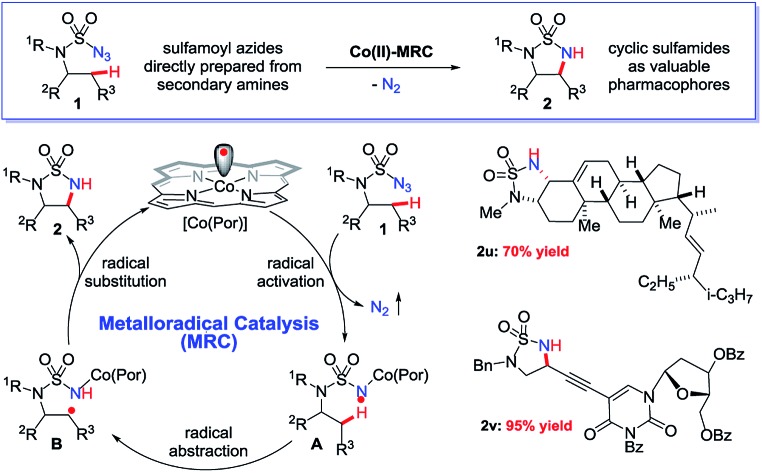
Synthesis of strained five-membered cyclic sulfamides has been achieved for the first time by intramolecular 1,5-C(sp^3^)–H amination *via* Co(ii)-based metalloradical catalysis.

## Introduction

**Scheme 1:**
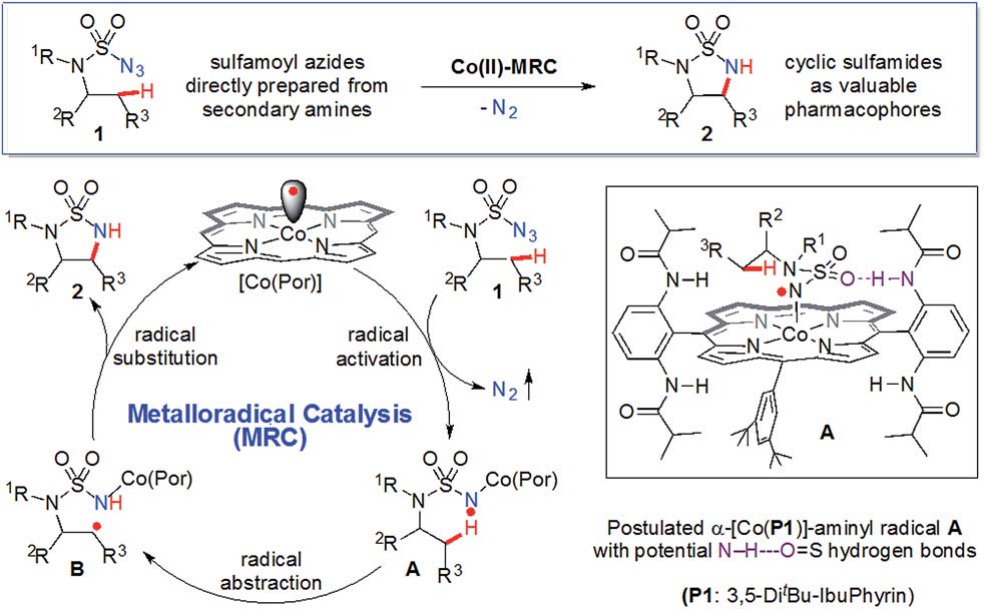
Metalloradical approach for 5-membered cyclic sulfamides *via* radical C(sp^3^)–H amination.

**Fig. 1:**
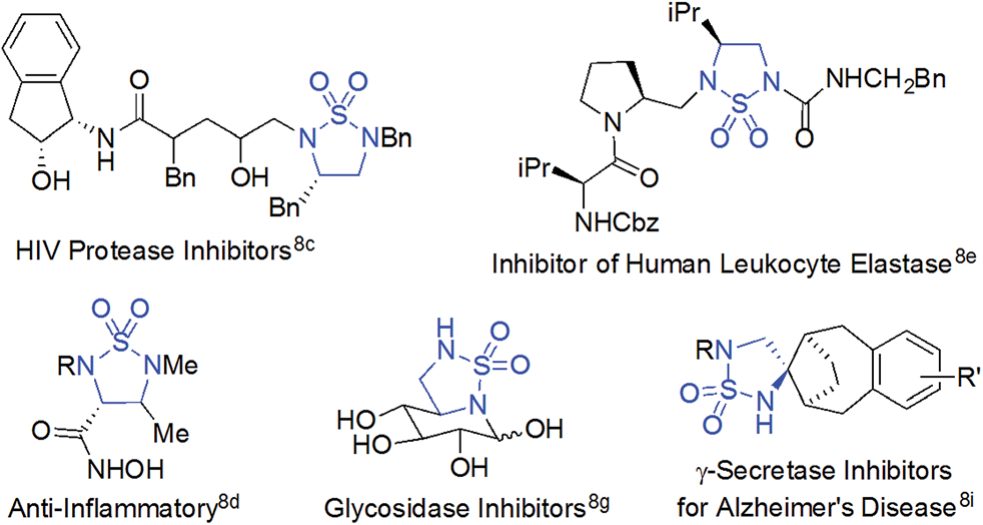
Selected examples of biologically active molecules containing the 5-membered cyclic sulfamide motif.

Radical chemistry has recently demonstrated increasing applications in organic synthesis, which has been traditionally dominated by the development of synthetic methods based on ionic chemistry.^1^ Among other approaches,^2^ metalloradical catalysis (MRC), which involves the development of metalloradical complexes as potential open-shell catalysts for initiating as well as controlling homolytic radical reactions, provides a fundamentally new strategy to address some long-standing challenges associated with radical chemistry.^3^,^4^ As stable metalloradicals, cobalt(ii) complexes of porphyrins [Co(Por)] have recently emerged as a unique class of catalysts for C–H amination through a 1e radical mechanism.^5^,^6^ Supported by porphyrin ligands bearing amide functionalities, the Co(ii)-based MRC has been shown to be particularly effective in activating various sulfamoyl azides for intramolecular radical amination of different types of C–H bonds, leading to the formation of 6-membered cyclic sulfamides with a high control of reactivities and selectivities.^7^ The success of the metalloradical system is attributed to the stabilization of the key α-Co(iii)-aminyl radical intermediates (also known as Co(iii)-nitrene radicals) through hydrogen-bonding interaction with the amide group of the porphyrin ligand as well as the high propensity of the α-metalloaminyl radicals toward 1,6-H-atom abstraction, followed by facile 6-*exo-tet* radical cyclization.^5^,^7^ To date, the feasibility of the α-metalloaminyl radicals for 1,5-H-atom abstraction to form the corresponding ε-Co(iii)-alkyl radicals (Scheme 1: **A** to **B**) and subsequent 5-*exo-tet* radical cyclization to generate the strained 5-membered cyclic sulfamides (Scheme 1: **B** to **2**) remains unexplored. If successful, the catalytic C–H amination process (Scheme 1) would be highly attractive as the resulting 5-membered cyclic sulfamides have found important applications in the development of pharmaceutical agents (Fig. 1).^8^

A number of different metal-based catalytic systems have been developed for regioselective intramolecular C(sp^3^)–H amination to prepare *N*-heterocycles of variable ring size and functionality.^9^,^10^ Although considerable advances have been made, there has been no previous reports of the efficient synthesis of 5-membered cyclic sulfamides *via* metal-catalyzed intramolecular C–H amination.^11^–^13^ It is evident that catalytic 1,5-C(sp^3^)–H amination for the formation of 5-membered cyclic sulfamides is a challenging process, which is presumably attributable to the potentially strained [3.1.0]-bicyclic transition state associated with the asynchronous concerted mechanism that is shared by most catalytic C–H amination systems *via* metallonitrene intermediates.9j,^14^ Considering its stepwise radical mechanism through less-strained 6-membered monocyclic transition states (Scheme 1: **A**) followed by low-barrier radical substitution,5*a* we anticipated the possibility of applying MRC to address the challenges of this transformation (Scheme 1). Herein, we report that metalloradical catalysts [Co(Por)] are highly effective in activating sulfamoyl azides for intramolecular 1,5-C–H radical amination under neutral and nonoxidative conditions, affording the strained 5-membered cyclic sulfamides in high yields, with nitrogen gas as the only byproduct. In addition to its simple and practical protocol, the Co(ii)-based metalloradical system exhibits excellent chemoselectivity and high functional group tolerance.

## Results and discussion

**Scheme 2:**
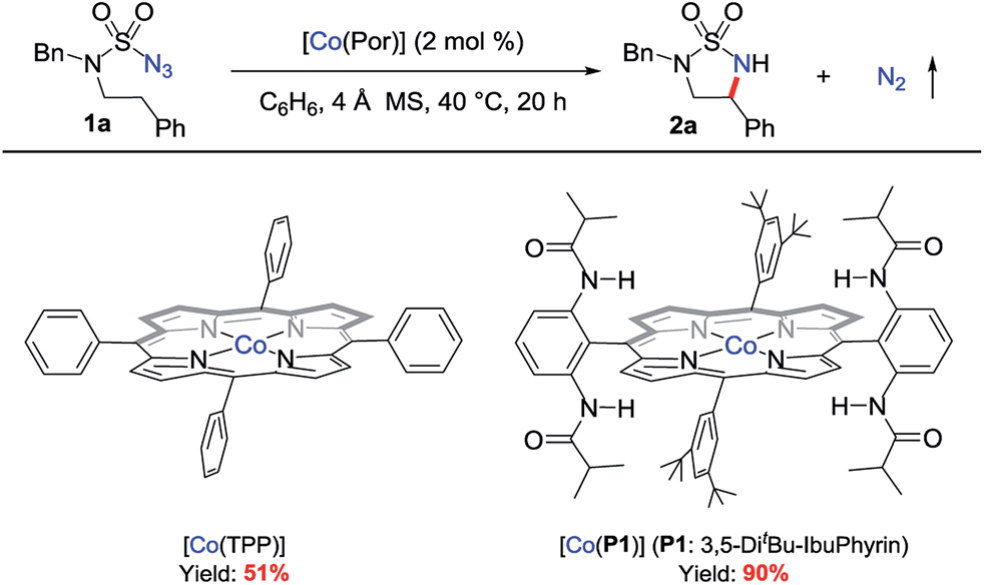
Ligand effect on Co(ii)-catalyzed 1,5-C(sp^3^)–H metalloradical amination.

At the outset of this project, the sulfamoyl azide **1a**,^15^ which contains benzylic C–H bonds for potential 1,5-H-atom abstraction, was selected as the model substrate to test the possibility of intramolecular 1,5-C(sp^3^)–H amination *via* MRC (Scheme 2). Initial experiments showed that the metalloradical complex [Co(TPP)] (TPP: tetraphenylporphyrin), which is commercially available, could activate azide **1a** for the intramolecular radical amination of the benzylic C(sp^3^)–H bond, affording the strained 5-membered cyclic sulfamide **2a** in 51% yield. Further optimization experiments indicated that [Co(**P1**)], which is supported by the *D*_2h_-symmetric amidoporphyrin 3,5-Di^*t*^Bu-IbuPhyrin (**P1**), was a superior metalloradical catalyst for the radical C–H amination reaction, leading to the formation of the desired **2a** in 90% yield (Scheme 2). The enhanced catalytic activity of [Co(**P1**)] over [Co(TPP)] is ascribed to the stabilization of the key α-Co(iii)-aminyl radical intermediate by the amide functionalities through hydrogen-bonding interaction (Scheme 1, **A**).5a,b,6c,d,^7^

Under the optimized conditions, the [Co(**P1**)]-based metalloradical system was shown to be effective for intramolecular 1,5-C(sp^3^)–H radical amination of a wide range of sulfamoyl azide substrates (Table 1). In addition to effective amination reactions of benzylic C–H bonds with varied electronic properties (entries 1–4), the Co(ii)-based catalytic system could efficiently aminate α-C(sp^3^)–H bonds of heteroaromatic rings such as furan (entry 5) and thiophene (entry 6), without complication from potential reactions with the heteroatoms. As exemplified by the high-yielding formation of *trans*-cyclic sulfamide **2c**, excellent diastereoselectivity could be achieved (entry 3). The metalloradical amination by [Co(**P1**)] could also be applied for non-benzylic C–H substrates, as demonstrated with the successful formation of the cyclic sulfamide **2g** and bicyclic sulfamide **2h** in respectable yields (entries 7 and 8), along with the corresponding 6-membered structure product formation.12*g* Moreover, even the challenging electron-deficient C(sp^3^)–H substrates, such as α-C–H bonds of esters and amides, could be aminated smoothly, producing α,β-diamino acid derivatives (entries 9 and 10).7*c* Besides secondary C–H bonds, this system proceeded successfully with more sterically hindered tertiary C(sp^3^)–H bonds as well (entries 7 and 11). It is notable that the α,β-diamino acid derivative **2k** bearing a quaternary α-carbon center could be synthesized in near quantitative yield (entry 11). Different *N*-substituents in the azide substrates were effectively tolerated in the C–H amination process. For example, sulfamoyl azides containing both electron-donating and electron-withdrawing *N*-substituents, such as *N*-benzyl (entry 1), *N*-methyl (entry 2), *N*-Boc groups (entry 11), and *N*-4-methoxybenzyl (entry 15), groups proved to be suitable substrates.

**Table 1:** Intramolecular 1,5-C(sp^3^)–H radical amination of sulfamoyl azides by metalloradical catalyst [Co(**P1**)]^*a*^

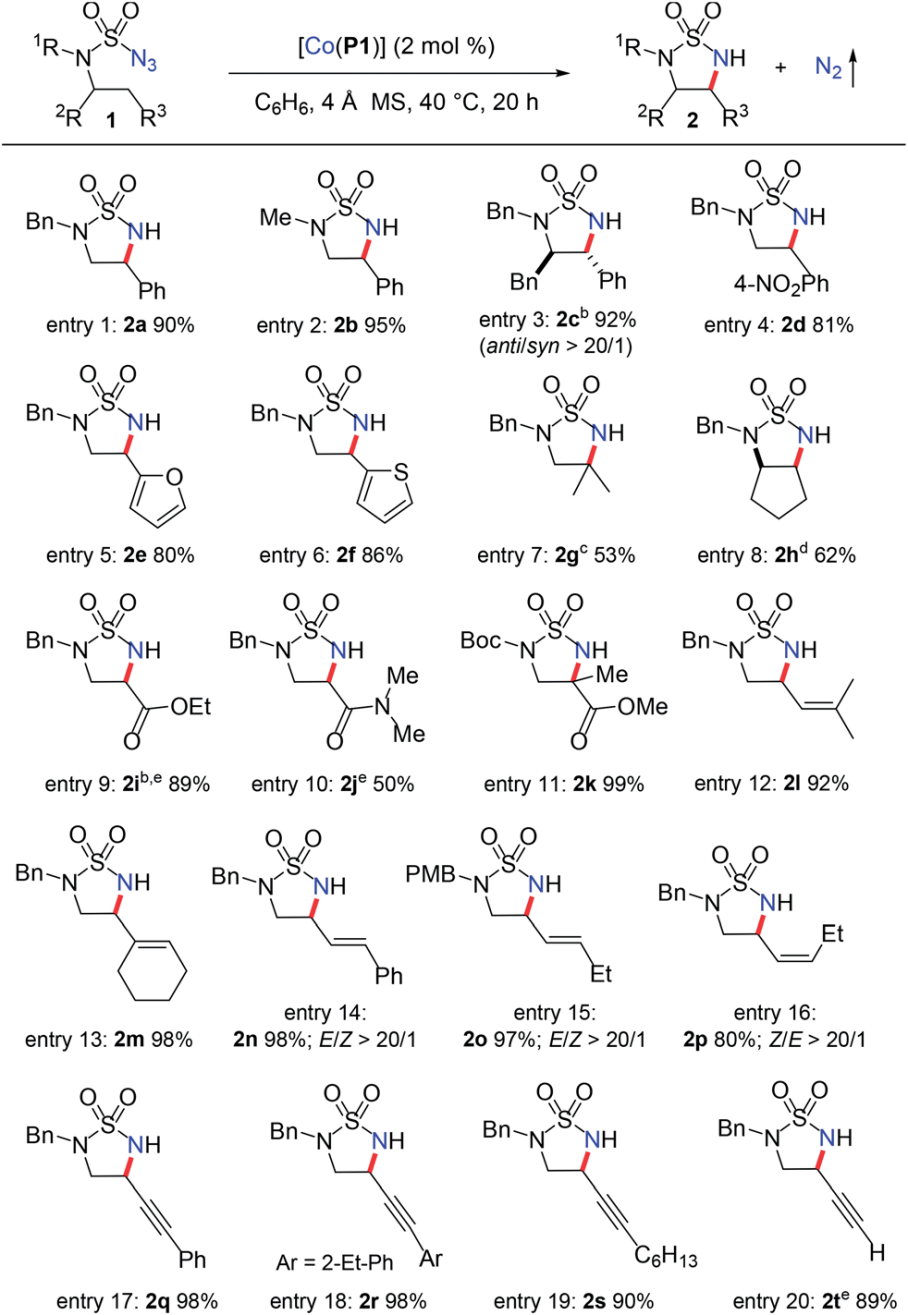

^*a*^Performed in C_6_H_6_ at 40 °C for 20 h using 2 mol% [Co(**P1**)] under N_2_ in the presence of 4 Å MS; [azide **1a**] = 0.10 M; isolated yields.

^*b*^Confirmed by X-ray crystallographic structure analysis.

^*c*^Yield based on ^1^H NMR analysis of purified mixture of 1,5- and 1,6-products, 37% 6-membered ring product was also obtained.

^*d*^18% of the 6-membered ring product was obtained.

^*e*^5 mol% [Co(**P1**)].

The [Co(**P1**)]-catalyzed 1,5-C–H radical amination system exhibited excellent chemoselectivity towards allylic C–H bonds without affecting the C=C π bonds.12e,f,^16^ For instance, the allylic C–H bonds of electron-rich trisubstituted alkenes were effectively aminated in high yields to afford the corresponding 5-membered cyclic sulfamides **2l** and **2m** without observation of the corresponding aziridination products (entries 12 and 13). Furthermore, this amination process was shown to be stereospecific regarding the stereochemistry of the alkene units as exemplified by the catalytic reactions of both *trans*- and *cis*-alkene-derived sulfamoyl azide substrates (entries 14–16). Under the standard conditions, the expected allylic C–H amination products **2n**, **2o** and **2p** were formed in high yields with excellent stereospecificity as well as chemoselectivity. The fact that no olefin isomerization was observed during these catalytic amination reactions suggests the 5-*exo-tet* radical cyclization of the corresponding ε-Co(iii)-allylic radical (Scheme 1: **B** to **2**) proceeds with a low barrier and even faster than the facile *trans*- and *cis*-C=C π bond isomerization.^17^ The Co(ii)-based MRC is among the few catalytic systems that are effective for amination of propargylic C–H bonds without affecting the electron-rich C≡C π bonds.7d,^18^,^19^ Functionalization of propargylic C–H bonds of both aryl-conjugated and alkyl-substituted alkynes led to the formation of the desired 5-membered cyclic sulfamides in high yields (entries 17–19). Moreover, the propargylic C–H bond of unprotected terminal alkynes could be also aminated selectively without interference from the acidic terminal C(sp)–H bond, as demonstrated by the formation of the cyclic sulfamide **2t** in 89% yield.

**Scheme 3:**
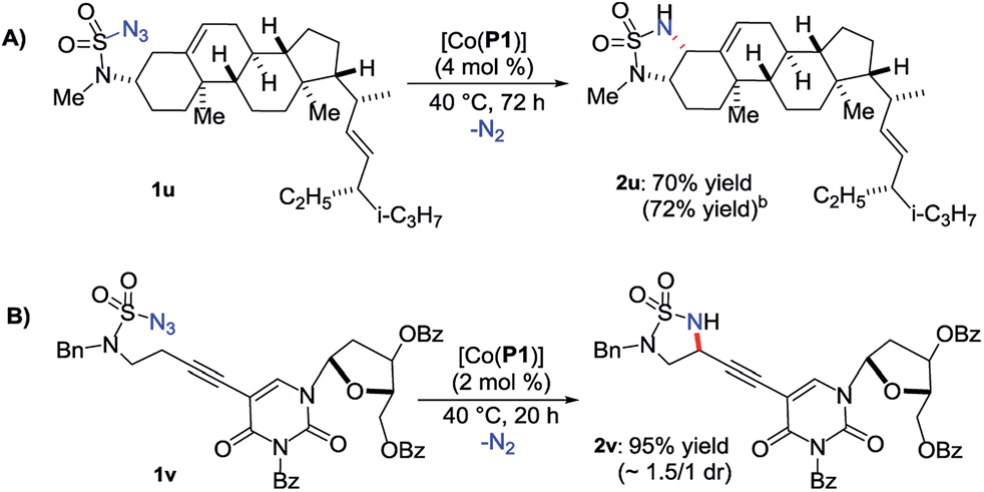
Late-stage functionalization of complex molecules by Co(ii)- based 1,5-C–H radical amination^*a*^. (a) Isolated yields. (b) On 0.5 mmol scale.

The demonstrated chemoselectivity and functional group tolerance of [Co(**P1**)]-catalyzed 1,5-C–H amination made it possible for late-stage functionalization of complex molecules in a predicable fashion. For example, when stigmasterol-based azide **1u**, which was directly prepared from the corresponding amine by a one-step procedure (ESI†),^15^ was used as a substrate, 1,5-amination of the allylic C–H bond among various C–H and C=C bonds was chemoselectively achieved, providing the fused multicyclic sulfamide **2u** in 70% yield (Scheme 3A) with the *cis*-stereoisomer only.^20^ We also showed that the reaction could be effectively scaled up to 0.5 mmol in a similar 72% yield. As a further demonstration of the functional group tolerance of the current catalytic system, when the deoxyuridine-based substrate **1v**, which was prepared directly from the corresponding deoxyuridine-based amine (ESI†),^15^ was treated with [Co(**P1**)], the propargylic C–H bond was selectively aminated to afford the deoxyuridine-derived 5-membered cyclic sulfamide **2v** in 95% yield (Scheme 3B). Direct modification of highly functionalized amine compounds with known bioactivities may offer an attractive opportunity to obtain unexplored 5-membered cyclic sulfamides like **2u** and **2v** for the study of interesting biological activities.^8^ In addition, the resulting cyclic sulfamides **2** bearing various functionalities may also serve as efficient precursors for the preparation of the valuable corresponding 1,2-diamines.^21^ For example, cyclic sulfamide **2m** was effectively converted to the corresponding unprotected 1,2-diamine derivative **2m′** in 91% yield (eqn (1)).
1

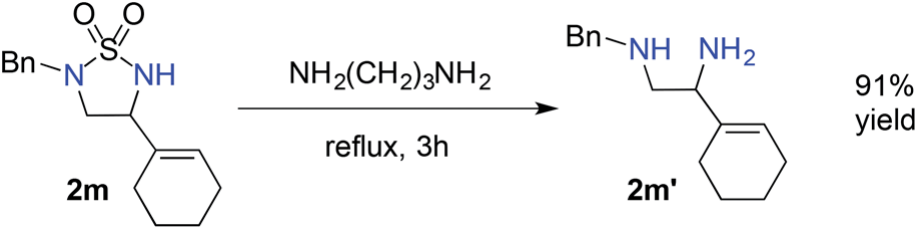




**Fig. 2:**
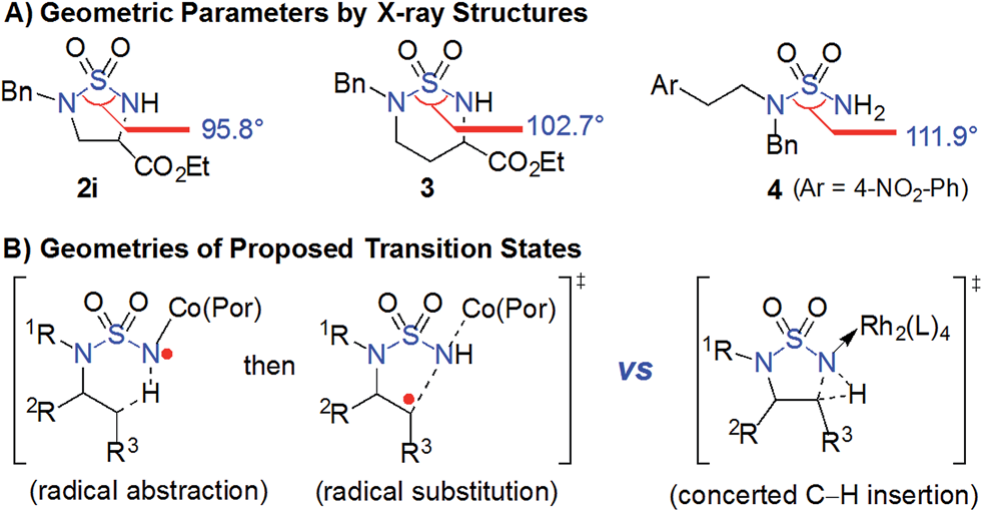
(A) Comparison of geometric parameters between acyclic and cyclic sulfamides based on X-ray structures. (B) Geometries of proposed transitional states for stepwise (left) and concerted (right) processes of intramolecular 1,5-C(sp^3^)–H amination.

The catalytic capability of Co(ii)-based metalloradical catalysis (MRC) to facilitate intramolecular 1,5-C(sp^3^)–H amination to form 5-membered cyclic sulfamides is uniquely remarkable. The challenge of this type of transformation can be appreciated by examining the geometric parameters of 5-membered cyclic sulfamides in comparison with those of acyclic and 6-membered cyclic sulfamides. To this end, we synthesized and structurally characterized sulfamides **2i**, **3** and **4** by X-ray crystallography (ESI†). As illustrated in Fig. 2A, while the N–S–N bond angle of the 6-membered cyclic sulfamide **3** is 9.2° smaller than that of acyclic sulfamide **4**, the deviation in the N–S–N bond angle for 5-membered cyclic sulfamide **2i** from **4** is considerably larger (16.1°),^22^ signifying the great angle strain inherent in the 5-membered cyclic sulfamide structure. It would be expected to have even greater ring strain for the corresponding [3.1.0]-bicyclic transition state (Fig. 2B; right) associated with intramolecular 1,5-C(sp^3^)–H amination *via* the asynchronous concerted mechanism through metallonitrene intermediates.^12^,^14^ This presumably accounts for the previous absence of effective catalytic systems for 1,5-C(sp^3^)–H amination towards formation of 5-membered cyclic sulfamides.^11^,^12^ Through a fundamentally different pathway involving the two-step radical cascade (Scheme 1: **A** to **B** and then **B** to **2**), Co(ii)-based metalloradical catalysis (Fig. 2B; left) effectively obviates a highly strained [3.1.0]-bicyclic transition state, allowing for efficient construction of 5-membered cyclic sulfamides.^6^–^8^

Together with the previous reports on intramolecular 1,6-C(sp^3^)–H amination to form 6-membered cyclic sulfamides,^7^ the current work reveals the versatile pathways of Co(ii)-based MRC for selective amination.^23^ Although the key α-metalloaminyl radical intermediates are capable of undergoing both 1,5- and 1,6-H-atom abstraction followed by facile 5- and 6-*exo-tet* radical cyclization, respectively, the differentiation of the two pathways toward a selective catalytic process can be effectively achieved by Co(ii)-MRC for most substrates in a predictable fashion.^7^

## Conclusions

In summary, by applying the concept of metalloradical catalysis (MRC), a new approach has been successfully demonstrated for addressing the challenges of intramolecular 1,5-C(sp^3^)–H amination to construct strained 5-membered cyclic sulfamides. The metalloradical complex [Co(**P1**)] is an effective catalyst with the capability of activating a broad scope of sulfamoyl azides for intramolecular 1,5-amination of different types of C(sp^3^)–H bonds with high stereospecificity, providing straightforward access to the potentially bioactive 5-membered cyclic sulfamide compounds in high yields. The Co(ii)-based catalytic system can be simply operated under neutral and non-oxidative conditions without the need for any additives, generating nitrogen gas as the only byproduct. Furthermore, this 1,5-C(sp^3^)–H amination process features excellent chemoselectivity and functional group tolerance, allowing for late-stage functionalization of complex molecules. The success in addressing this challenging amination process by Co(ii)-MRC is believed to be directly related to the underlying radical mechanism involving the key α-Co(iii)-aminyl radical intermediate.

## Supplementary Material

Supplementary information

Supplementary information

Crystal structure data
