# Supplementary material for: Intramolecular 1,5-C(sp3)–H radical amination via Co(ii)-based metalloradical catalysis for five-membered cyclic sulfamides
Source: Chem Sci. 2016 Jul 28;7(12):6934–9. doi: 10.1039/c6sc02231f (PMC5271564; doi:10.1039/c6sc02231f)

Electronic Supplementary Material (ESI) for Chemical Science.
This journal is © The Royal Society of Chemistry 2016

Supporting Information

Intramolecular 1,5-C(sp^3)–H Radical Amination via Co(II)-Based Metalloradical Catalysis for Five-Membered Cyclic Sulfamides

Hongjian Lu,[*#†‡] Kai Lang,[#§†] Huiling Jiang,[†] Lukasz Wojtas[†] and X. Peter Zhang[*§†]

[§]Department of Chemistry, Merkert Chemistry Center, Boston College, Chestnut Hill, Massachusetts 02467, United States

†Department of Chemistry, University of South Florida, Tampa, Florida 33620, United States

‡The Institute of Chemistry & Biomedical Sciences, Nanjing University, Nanjing, 210093, P. R. China

Table of Contents

Schemes S1, S2 and S3 SI-2

General Considerations SI-3

The Procedure for the Synthesis of Co(**P1**) SI-3

General Method for the Synthesis of Sulfamoyl Azides **1** SI-5

General Procedure for C–H Amination of Sulfamoyl Azides SI-19

The Synthetic Procedures for the Synthesis of Compounds **3** and **4** SI-30

The Procedures for Scheme S2 and S3 SI-31

X-ray Crystallography for Compounds **2c**, **2i**, **3** and **4** SI-32

DSC Spectrogram of a Sulfamoyl Azide SI-38

Scheme S1

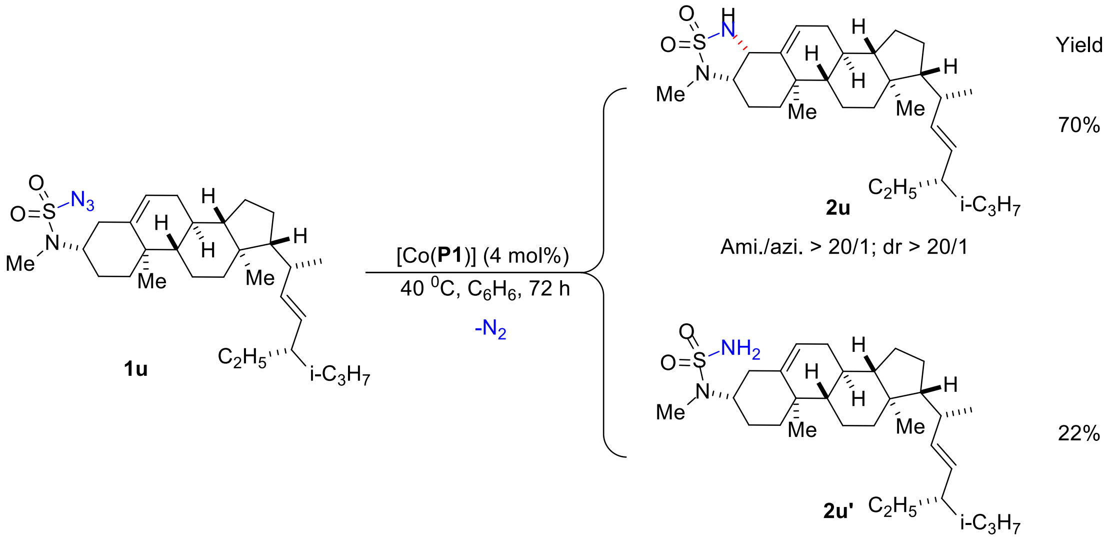

Scheme S2 (Recycle of 2u′ back to 1u)

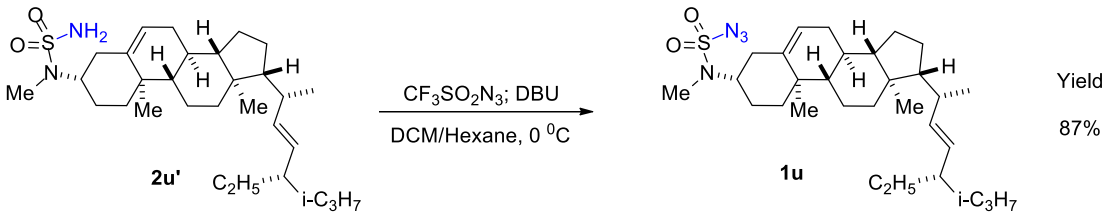

Scheme S3

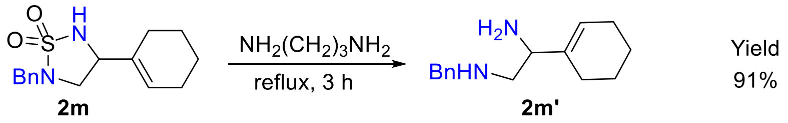

General Considerations. All C–H amination reactions were performed under nitrogen in oven-dried glassware following standard Schlenk techniques. 4 Å molecular sieves were dried in a vacuum oven prior to use. Anhydrous C_6H_6 was purchased from Sigma-Aldrich and used without further purification. [Co(**P1**)] was prepared by following the literature.[1] Thin layer chromatography was performed on Merck TLC plates (silica gel 60 F254). Flash column chromatography was performed with ICN silica gel (60 Å, 230-400 mesh, 32-63 μm). ^{1}H NMR and ^{13}C NMR were recorded on a Bruker250, Varian Inova300, Inova400 or Inova500 instrument with chemical shifts reported relative to residual solvent. Infrared spectra were measured with a Nicolet Avatar 320 spectrometer with a Smart Miracle accessory. HRMS data were obtained on an Agilent 1100 LC/MS/TOF mass spectrometer. **Note: Some azides could be explosive and should be handled carefully.**

The Procedure for Synthesis of Co(P1)[1]

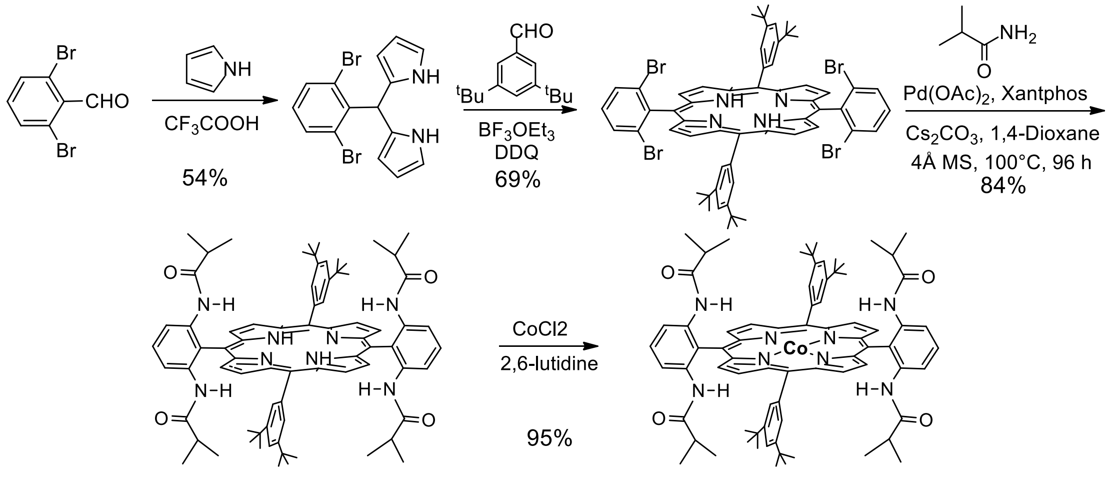

The compound was prepared according to the known procedure.[1] Meso-(2,6-dibromophenyl) dipyrromethane. A solution of 2,6-dibromobenzaldehyde (15.86 g, 60 mmol) in pyrrole (104 mL, 1.5 mol) was purged with argon for 30 min and treated under stirring with CF_3COOH (0.1 equiv, 6.0 mmol) for 3 h at ambient temperature. The solution of NaOH (240 mL, 0.1 mol/L) was then added and the mixture was extracted with EtOAc (3 x 100 mL). The combined organic layer was concentrated *in vacuo* until all pyrrole was completely removed. The dark residue was chromatographed on silica (gradient elution: 20/1→10/1 Hexane/EtOAc) to yield meso-(2,6-dibromophenyl)dipyrromethane (12.2 g, 54%) as a yellow solid. ^{1}H NMR (500 MHz, $CDCl_3$): δ 8.31 (brs, 2H), 7.58 (d, J = 7.5 Hz, 2H), 6.97 (t, J = 8.0 Hz, 1H), 6.76-6.73 (m, 2H), 6.55 (s, 1H), 6.23-6.19 (q, J = 3.0 Hz, 2H), 6.13-6.08 (m, 2H). ^{13}C NMR(125 MHz, $CDCl_3$): δ 139.3, 133.9 (brs), 129.4,129.1, 116.8, 108.7, 107.7, 44.6.

5,15-Bis(2,6-dibromophenyl)-10,20-bis[3,5-di(*tert*-butyl)phenyl]porphyrin. A mixture of meso-(2,6-dibromophenyl)dipyrromethane (380 mg, 1.0 mmol), aldehyde (218 mg, 1.0 mmol), and molecular sieves (4Å, 0.300 g) in chloroform (150 ml) was purged with nitrogen for 10 min. Boron trifluoride diethyl etherate (0.1 mL) was

[1] Chen, Y.; Fields, K. B.; Zhang, X. P. *J. Am. Chem. Soc.* **2004**, *126*, 14718.

added dropwise via a syringe and the flask was wrapped with aluminum foil to shield it from light. The solution was stirred under a nitrogen atmosphere at room temperature for 3 h, and 2,3-dichloro-5,6-dicyano-1,4-benzoquinone (DDQ) (272 mg, 1.2 mmol) was added as powder at one time. After 30 min, 1 mL of triethylamine was added. The reaction solution was then directly poured on the top of a silica gel column that was packed with dichloromethane. The column was eluted with dichloromethane. The fractions containing product were collected and concentrated on a rotary evaporator. The residue was washed several times with hexanes to afford the pure solid (398 mg, yield: 69%). ^{1}H NMR (300 MHz, $CDCl_3$): δ 8.90 (d, J = 4.8 Hz, 4H), 8.65 (d, J = 4.8 Hz, 4H), 8.11 (d, J = 1.5 Hz, 4H), 8.01 (d, J = 8.1 Hz, 4H), 7.79 (t, J = 1.8 Hz, 2H), 7.51 (t, J = 8.1 Hz, 2H), 1.53 (s, 36H), -2.52 (s, 2H).

3,5-Di^{t}Bu-IbuPhyrin **(P1).** An oven-dried Schlenk tube equipped with a stirring bar was degassed on a vacuum line and purged with nitrogen. The tube was then charged with above obtained 5,15-Bis(2,6-dibromophenyl)-10,20-bis[substituted-phenyl] porphyrin (231 mg, 0.2 mmol, 1 equiv), isobutyramide (278 mg, 3.2 mmol, 16 equiv), $Pd(OAc)_2$ (18 mg, 0.08 mmol, 40 mol%), Xantphos (92 mg, 0.16 mmol, 80 mol%), Cs_2CO_3 (1.04 g, 3.2 mmol, 16 equiv). The tube was capped with a Teflon screw cap, evacuated, and backfilled with nitrogen. After the Teflon screw cap was replaced with a rubber septum, 1,4-dioxane (10 mL) was added via syringe. The tube was purged with nitrogen (1-2 mins) and the septum was then replaced with the Teflon screw cap and sealed. The reaction mixture was heated in an oil bath at 100℃ with stirring for 72 hours. After removed solvent, the pure compound was isolated by flash column chromatography (gradient elution: 4/1→2/1 Hexanes/EtOAc) as a purple solid (198 mg, 84%), TLC R_f = 0.56 (1/1 Hexanes/EtOAc). ^{1}H NMR (400 MHz, $CDCl_3$): δ 8.97 (d, J = 4.4 Hz, 4H), 8.85 (d, J = 4.8 Hz, 4H), 8.48 (d, J = 7.6 Hz, 4H), 8.00 (s, 4H), 7.90-7.85 (m, 4H), 6.46 (s, 4H), 1.52 (s, 36H), 1.20 (m, 4H), 0.31 (d, J = 7.4 Hz, 24H), -2.53 (s, 2H).

[Co(3,5-Di^{t}Bu-IbuPhyrin)] **(**[Co(**P1**)]). 3,5-Di^{t}Bu-IbuPhyrin **(P1)** (590 mg, 0.5 mmol) and anhydrous $CoCl_2$ (650 mg, 5.0 mmol) were placed in an oven-dried, resealable Schlenk tube. The tube was capped with a Teflon screwcap, evacuated, and backfilled with nitrogen. The screw cap was replaced with a rubber septum, dry THF (20 mL) and 2,6-lutidine (268 mg, 2.5 mmol) were added via syringe. The tube was purged with nitrogen for 1-2 minutes, and then the septum was replaced with the Teflon screw cap. The tube was sealed, and its contents were heated in an oil bath at 100 °C with stirring overnight. The resulting mixture was cooled to room temperature, taken up in ethyl acetate, and transferred to a separatory funnel. The mixture was washed with water 3 times and concentrated. The pure compound was isolated by flash column chromatography (gradient elution: 4/1→2/1 Hexanes/EtOAc), red solid (587.3 mg, yield: 95%), TLC R_f = 0.56 (1/1 Hexanes/EtOAc). UV–vis ($CHCl_3$), λ_{max}nm (log ε): 415(5.23), 530(4.19).

The Method for the Synthesis of Sulphuryl Azide ($N_3SO_2N_3$)[2]

To a solution of sodium azide (2.6 g, 40 mmol) and pyridine (1.58 g, 20 mmol) in acetonitrile (50 ml) at 0 °C, sulphuryl chloride (1.34 g, 10 mmol) in acetonitrile (20 ml) was added dropwise for 10 min. Then the reaction mixture was stirred for a further 1 h at room temperature. After addition of 30 ml DCM, the mixture was poured into ice-cold water and extracted with DCM (3 x 20 mL) The combined organic layer was washed with hydrochloric acid (1 mol/L in H_2O), water, potassium hydroxide (1 mol/L in H_2O), hydrochloric acid (1 mol/L in H_2O), and water. After drying (Na_2SO_4), the sulphuryl azide solution was used directly for the subsequent reaction. This solution can be stored in the refrigerator for at least two months without significant decomposition.

General Method for the Synthesis of Sulfamoyl Azides

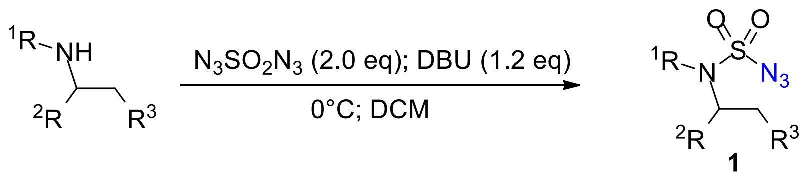

To a solution of $N_3SO_2N_3$ (2 equiv, 0.25 mol/L in DCM) at 0 °C, a mixture of amine (1 equiv) and DBU (1.2 equiv) in DCM was added dropwise via syringe. The reaction showed almost complete consumption of the starting amine after 5 min to 3 hours when monitored by TLC, then the majority of the solvent was removed under reduced pressure at room temperature. Purification of this mixture by chromatography on silica gel (as given below) afforded the sulfamoyl azide. **Note: Some azides could be explosive and should be handled carefully.** Based on the DSC spectrogram of sulfamoyl azide on page SI-37, this type of azide is stable under the reaction conditions used.

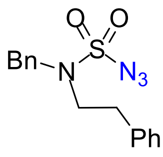 **1a**

N-Benzyl-2-phenylethan-1-sulfamoyl azide (**1a**) was obtained in 86% yield through General Method from the commercially available amine (*N*-Benzyl-2-phenethylamine, cas: 3647-71-0). Purified by chromatography on silica gel (gradient elution: 30/1→20/1 Hexanes/EtOAc), colorless liquid, TLC R_f = 0.73 (10/1 Hexanes/EtOAc). ^{1}H NMR (500 MHz, $CDCl_3$): δ 7.43-7.22 (m, 8H), 7.10 (d, J = 7.0 Hz, 2H), 4.43 (s, 2H), 3.44 (t, J = 8.0 Hz, 2H), 2.85 (t, J = 8.0 Hz, 2H). ^{13}C NMR (125 MHz, $CDCl_3$): δ 137.5, 134.4, 128.9, 128.7, 128.6, 128.6, 128.5, 126.7, 52.9, 49.9, 34.3. IR (neat, cm^{-1}): 2123(N_3), 1380, 1204, 1164, 734, 697.

---

[2] (a) Nojima, M. *J. Chem. Soc., Perkin Trans. 1*, **1979**, 1811. (b) Lu, H. J.; Hu, Y.; Jiang, H. L.; Wojtas, L.; Zhang, X. P. *Org. Lett.* **2012**, *14*, 5158.

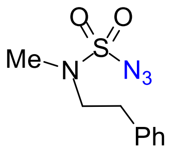

1b

N-Methyl-2-phenylethan-1-sulfamoyl azide **(1b)** was obtained in 71% yield through General Method from the commercially available amine (*N*-methylphenethylamine, cas: 589-08-2). Purified by chromatography on silica gel (gradient elution: 30/1→20/1 Hexane/EtOAc), colorless liquid, TLC R_f = 0.55 (10/1 Hexane/EtOAc). ^{1}H NMR (400 MHz, $CDCl_3$): δ 7.34-7.29 (m, 2H), 7.26-7.19 (m, 3H), 3.47 (t, J = 8.0 Hz, 2H), 2.92 (t, J = 8.0 Hz, 2H), 2.91 (s, 3H). ^{13}C NMR (100 MHz, $CDCl_3$): δ 137.4, 128.7, 126.9, 53.0, 36.3, 34.1. IR (neat, cm^{-1}): 2123(N_3), 1380, 1205, 1163, 964, 736, 698.

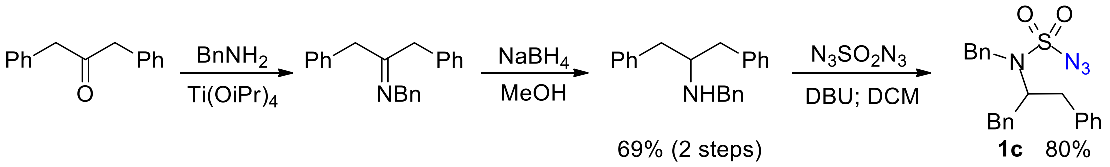

At room temperature, to a 1,3-diphenylpropanone (1.0 g, 4.7 mmol) DCM solution (20 mL) was added $Ti(OiPr)_4$ (1.5 mL, 5.2 mmol), followed by the addition of benzylamine (0.8 mL, 7.3 mmol). The reaction mixture was stirred for 1 hour and the solvent was removed. The residue was dissolved into MeOH (20 mL) and cooled to 0 °C. Then $NaBH_4$ (0.53 g, 14.1 mmol) was added to this solution and the reaction mixture was slowly warmed up to room temperature and stirred for 2 hours. Then the solvent was removed and the residue was purified by flash silica gel chromatography (gradient elution: 2/1→1/1 Hexane/EtOAc) to give the desired amine product *N*-benzyl-1,3-diphenylpropan-2-amine as yellow oil (980 mg, 69% yield). ^{1}H NMR (500 MHz, $CDCl_3$): δ 7.35-7.19 (m, 10H), 7.19-7.11 (m, 3H), 7.10-7.03 (m, 2H), 3.79 (s, 2H), 3.08 (quin, J = 6.6 Hz, 1H), 2.83-2.71 (m, 4H), 1.56 (brs, 1H). ^{13}C NMR (125 MHz, $CDCl_3$): δ 140.3, 139.4, 129.4, 128.4, 128.3, 127.9, 126.8, 126.2, 59.7, 51.3, 40.7. HRMS (ESI) ($[M+H]^+$) Calcd. for $C_{22}H_{24}N^+$: 302.1904, Found: 302.1922.

N-Benzyl-1-benzyl-2-phenylethan-1-sulfamoyl azide **(1c)** was obtained in 80% yield following General Method from the *N*-benzyl-1,3-diphenylpropan-2-amine. Purified by chromatography on silica gel (gradient elution: 16/1→8/1 Hexane/EtOAc), colorless oil, TLC R_f = 0.6 (8/1 Hexane/EtOAc). ^{1}H NMR (500MHz, $CDCl_3$): δ 7.35-7.20 (m, 11H), 7.02 (d, J = 7.0 Hz, 4H), 4.24 (s, 2H), 3.99 (t, J = 6.5 Hz, 1H), 3.06 (dd, J = 7.5, 14.0 Hz, 2H), 2.81 (dd, J = 7.5, 14.0 Hz, 2H). ^{13}C NMR (125 MHz, $CDCl_3$): δ 137.9, 135.2, 129.0, 128.8, 128.7, 128.6, 128.3, 126.7, 65.2, 52.7, 38.9. IR (neat, cm^{-1}): 2123(N_3), 1454, 1376, 1164, 907, 729, 698, 601.

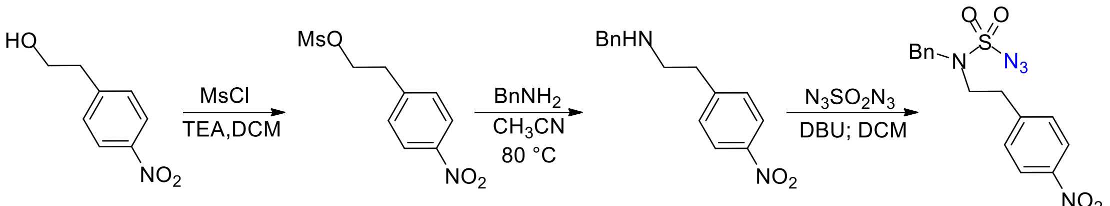

94% (2 steps) **1d** 85%

To a round bottom flask 2-(4-nitrophenyl)ethan-1-ol (334 mg, 2 mmol) (commercially available, cas: 100-27-6) in DCM (15 mL) was added methanesulfonyl chloride (0.23 mL, 3 mmol) followed by the addition of triethyl amine (0.85 mL, 6 mmol), the precipitate was formed immediately. The reaction mixture was stirred at room temperature for about 2 hours until the alcohol was fully consumed based on TLC. DCM (30 mL) was added into the reaction mixture and washed by water (50 mL). The aqueous solution was extracted by DCM (3 x 20 mL). The combined organic layer was dried over anhydrous sodium sulfate. The solvent was removed and CH_3CN (20 mL) was added, followed by the addition of benzylamine (0.44 mL, 4 mmol). The reaction was heated at 80° C for 6 hours. Then the solvent was removed and the residue was purified by flash silica gel chromatography (2/1 to 1/1 Hexane/EtOAc) to give *N*-benzyl-2-(4-nitrophenyl)ethyl-1-amine (480 mg, 94% yield), which was a known compound[3] and used directly for the next step. ^{1}H NMR (500 MHz, $CDCl_3$): δ 8.15 (d, J = 8.3 Hz, 2H), 7.37 (d, J = 8.3 Hz, 2H), 7.35-7.25 (m, 5H), 3.82 (s, 2H), 3.03-2.87 (m, 4 H).

N-Benzyl-2-(4-nitrophenyl)ethyl-1-sulfamoyl azide **(1d)** was obtained in 85% yield through General Method from *N*-benzyl-2-(4-nitrophenyl)ethyl-1-amine. Purified by chromatography on silica gel (8/1 Hexane/EtOAc), white solid, TLC R_f = 0.35 (4/1 Hexane/EtOAc). ^{1}H NMR (500 MHz, $CDCl_3$): δ 8.09 (d, J = 9.0 Hz, 2H), 7.39-7.36 (m, 3H), 7.32-7.28 (m, 2H), 7.18 (d, J = 8.5 Hz, 2H), 4.42 (s, 2H), 3.45 (t, J = 7.5 Hz, 2H), 2.90 (t, J = 7.5 Hz, 2H). ^{13}C NMR (125 MHz, $CDCl_3$): δ 146.9, 145.1, 134.1, 129.6, 129.0, 128.8, 128.7, 123.8, 53.6, 49.6, 34.4. IR (neat, cm^{-1}): 2125(N_3), 1601, 1517, 1378, 1344, 1164, 1109, 696.

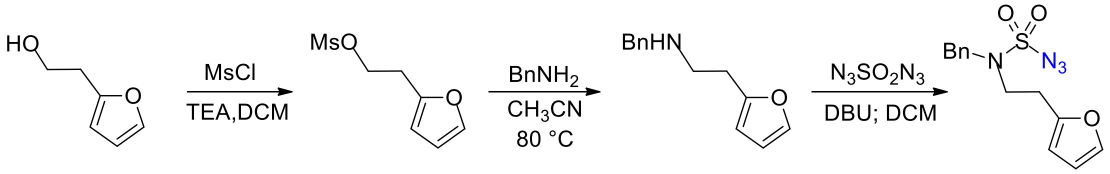

56% (2 steps) **1e** 80%

To a round bottom flask 2-(furan-2-yl)ethan-1-ol (224 mg, 2 mmol) (prepared according to the reference[4]) in DCM (15 mL) was added methanesulfonyl chloride (0.23 mL, 3 mmol) followed by the addition of triethyl amine (0.85 mL, 6 mmol), the precipitate was formed immediately. The reaction mixture was stirred at room temperature for about 2 hours until the alcohol was fully consumed based on TLC. DCM (30 mL) was added into the reaction mixture and washed by water (50 mL). The aqueous solution was extracted by DCM (3 x 20 mL). The combined organic

[3] Munro-Leighton, C.; Delp, S. A.; Alsop, N. M.; Blue, E. D.; Gunnoe, T. B. *Chem. Commun.* **2008**, 111.

[4] Larson, G. L.; Fry, J. L. *Org. Reactions* (Hoboken, NJ, United States), **2008**, *71*, 1.

layer was dried over anhydrous sodium sulfate. The solvent was removed and CH_3CN (20 mL) was added, followed by the addition of benzylamine (0.44 mL, 4 mmol). The reaction was heated at 80° C for 6 hours. Then the solvent was removed and the residue was purified by flash silica gel chromatography (2/1 to 1/1 Hexane/EtOAc) to give *N*-benzyl-2-(furan-2-yl)ethan-1-amine (225 mg, 56% yield), which was a known compound[5] and used directly for the next step. ^{1}H NMR (500 MHz, $CDCl_3$): δ 7.36-7.29 (m, 5H), 7.28-7.22 (m, 1H), 6.30 (dd, *J* = 2.0, 2.9 Hz, 1H), 6.12-6.01 (m, 1H), 3.82 (s, 2H), 2.97-2.91 (m, 2H), 2.89-2.83 (m, 2H), 1.61 (brs, 1H).

N-Benzyl-2-(furan-2-yl)ethan-1-sulfamoyl azide (**1e**) was obtained in 80% yield through the General Method from *N*-benzyl-2-(furan-2-yl)ethan-1-amine. Purified by chromatography on silica gel (16/1 Hexane/EtOAc), colorless oil TLC R_f = 0.5 (16/1 Hexane/EtOAc). ^{1}H NMR (400 MHz, $CDCl_3$): δ 7.41-7.30 (m, 6H), 6.28 (dd, *J* = 2.0, 3.2 Hz, 1H), 6.05 (dd, *J* = 0.8, 3.2 Hz, 1H), 4.35 (s, 2H), 3.51 (t, *J* = 7.2 Hz, 2H), 2.90 (t, *J* = 7.2 Hz, 2H). ^{13}C NMR (125 MHz, $CDCl_3$): δ 151.4, 141.6, 134.5, 128.9, 128.5, 128.4, 110.5, 107.0, 52.9, 47.0, 26.8. IR (neat, cm^{-1}): 2126(N_3), 1379, 1194, 1165, 906, 727.

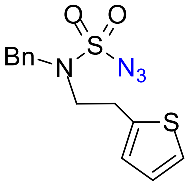

1f

N-Benzyl-2-(thiophen-2-yl)ethan-1-sulfamoyl azide (**1f**) was obtained in 85% yield through the General Method from *N*-benzyl-2-(thiophen-2-yl)ethan-1-amine (prepared according to the literature[6]). Purified by chromatography on silica gel (16/1 Hexane/EtOAc), colorless oil, TLC R_f = 0.55 (10/1 Hexane/EtOAc). ^{1}H NMR (400 MHz, $CDCl_3$): δ 7.42-7.32 (m, 5H), 7.16 (d, *J* = 4.4 Hz, 1H), 6.94-6.91 (m, 1H), 6.78 (d, *J* = 2.8 Hz, 1H), 4.42 (s, 2 H), 3.48 (t, *J* = 7.6 Hz, 2H), 3.05 (t, *J* = 8.0 Hz, 2H). ^{13}C NMR (125 MHz, $CDCl_3$): δ 139.6, 134.4, 129.0, 128.6, 128.6, 127.1, 125.7, 124.2, 53.2, 50.0, 28.5. IR (neat, cm^{-1}): 2126(N_3), 1380, 1204, 1167, 905, 727, 698.

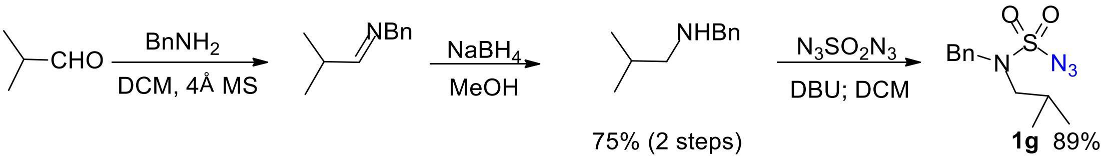

At room temperature, to a isobutyl aldehyde (500 mg, 7 mmol) DCM solution (25 mL), oven-dried 4 Å molecular sieves (1.0 g) was added, followed by the addition of benzylamine (0.84 mL, 7.7 mmol). The reaction mixture was stirred for 2 hours and the solvent was removed. The residue was dissolved into MeOH (15 mL) and cooled to 0 °C. Then $NaBH_4$ (770 mg, 21 mmol) was slowly added to this solution and the reaction mixture was warmed up to room temperature and stirred for 2 hours. Then

[5] Treus, M.; Harwood, L. M.; Estevez, J. C.; Salas, C.; Drew, M. G. B.; Estevez, R. J. *Synlett* **2013**, *24*, 2221.

[6] Sathiyaraj, E.; Thirumaran, S. *Spectrochim. Acta. A. Mol. .Biomol. Spectrosc.* **2012**, *97*, 575.

the solvent was removed and the residue was purified by flash silica gel chromatography (4/1 Hexane/EtOAc) to give the desired amine product *N*-benzyl-2-methylpropan-1-amine as yellow oil (850 mg, 75% yield). ^{1}H NMR (500 MHz, $CDCl_3$): δ 7.43-7.24 (m, 5H), 3.78 (s, 2H), 2.46 (d, J = 7.0 Hz, 2H), 1.84-1.74 (m, 1H), 1.29 (brs, 1H), 0.93 (d, J = 7.0 Hz, 6H). ^{13}C NMR (125 MHz, $CDCl_3$): δ 140.7, 128.3, 128.0, 126.8, 57.5, 54.1, 28.3, 20.7. HRMS (ESI) ($[M+H]^+$) Calcd. for $C_{11}H_{18}N^+$: 164.1434, Found: 164.1431.

N-Benzyl-2-methylpropan-1-sulfamoyl azide (**1g**) was obtained (240 mg, 89% yield) following the General Method from the *N*-benzyl-2-methylpropan-1-amine. Purified by chromatography on silica gel (gradient elution: 16/1→8/1 Hexane/EtOAc), colorless oil, TLC R_f = 0.6 (8/1 Hexane/EtOAc). ^{1}H NMR (500 MHz, $CDCl_3$): δ 7.40-7.33 (m, 5H), 4.47 (s, 2H), 3.04 (d, J = 7.5 Hz, 2H), 1.94-1.82 (m, 1H), 0.88 (d, J = 7.0 Hz, 6H). ^{13}C NMR (125 MHz, $CDCl_3$): δ 134.6, 128.8, 128.7, 128.4, 56.2, 53.3, 26.7, 19.9. IR (neat, cm^{-1}): 2964, 2121(N_3), 1372, 1205, 1183, 1159, 1024, 780, 735.

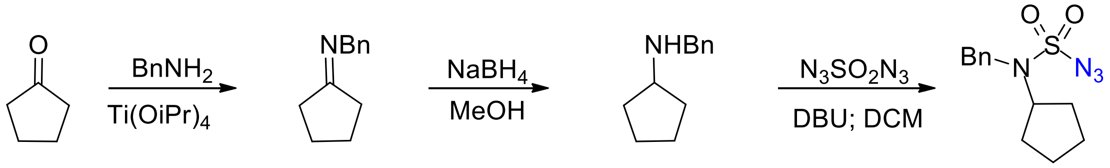

85% (2 steps) **1h** 88%

At room temperature, to a cyclopentanone (1.0 g, 12 mmol) DCM solution (35 mL) was added $Ti(OiPr)_4$ (3.8 mL, 12.8 mmol), followed by the addition of benzylamine (2.0 mL, 18 mmol). The reaction mixture was stirred for 1 hour and the solvent was removed. The residue was dissolved into MeOH (25 mL) and cooled to 0 °C. Then $NaBH_4$ (1.3 g, 36 mmol) was slowly added to this solution and the reaction mixture was warmed up to room temperature and stirred for 2 hours. Then the solvent was removed and the residue was purified by flash silica gel chromatography (4/1 Hexane/EtOAc) to give the desired amine product *N*-benzylcyclopentanamine as yellow oil (1.8 g, 85% yield). ^{1}H NMR (500 MHz, $CDCl_3$): δ 7.33 (d, J = 4.0 Hz, 4H), 7.27-7.23 (m, 1H), 3.79 (s, 2H), 3.13 (q, J = 6.5 Hz, 1H), 1.91-1.83 (m, 2H), 1.76-1.67 (m, 2H), 1.59-1.51 (m, 2H), 1.43-1.35 (m, 2H). ^{13}C NMR (125 MHz, $CDCl_3$): δ 140.8, 128.3, 128.1, 126.8, 59.1, 52.8, 33.2, 24.1. HRMS (ESI) ($[M+H]^+$) Calcd. for $C_{12}H_{18}N^+$: 176.1434, Found: 176.1432.

N-Benzyl-2-cyclopenyl-1-sulfamoyl azide (**1h**) was obtained (246 mg, 88% yield) following the General Method from the *N*-benzylcyclopentanamine. Purified by chromatography on silica gel (gradient elution: 16/1→8/1 Hexane/EtOAc), colorless oil, TLC R_f = 0.75 (8/1 Hexane/EtOAc). ^{1}H NMR (500 MHz, $CDCl_3$): δ 7.36-7.34 (m, 4H), 7.32-7.26 (m, 1H), 4.43 (s, 2H), 4.28-4.20 (m, 1H), 1.91-1.86 (m, 2H), 1.68-1.52 (m, 6H). ^{13}C NMR (125 MHz, $CDCl_3$): δ 137.0, 128.6, 127.7, 126.9, 61.2, 49.0, 29.3, 23.4. IR (neat, cm^{-1}): 2958, 2119(N_3), 1374, 1204, 1161, 1055, 860, 731.

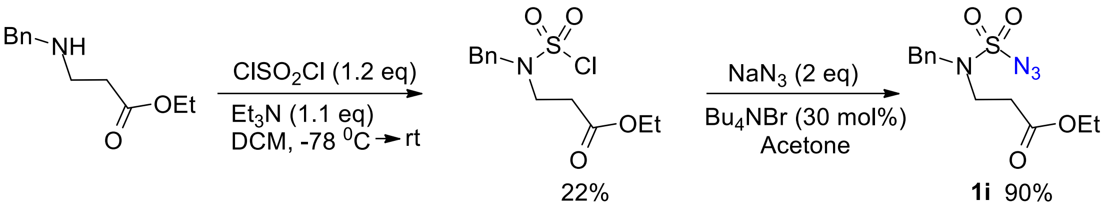

To a solution of $ClSO_2Cl$ (0.25 mol/L in DCM, 6.0 mmol) at -78 °C, a mixture of Ethyl 3-(benzylamino)propanoate (1.04 g, 5.0 mmol) and Et_3N (0.77 ml, 5.5 mmol) in DCM was added dropwise via syringe. The reaction mixture was stirred for overnight at -78 ^{0}C. After warming up to room temperature, the majority of the solvent was removed under reduced pressure at room temperature. The crude sulfamoyl chloride (340 mg, 22%) was obtained through a short silica gel column and used directly for the subsequent reaction.

Sodium azide (143 mg, 2.2 mmol) and Bu_4NBr (106 mg, 0.33 mmol) was added in portions to the crude sulfamoyl chloride in 10 mL acetone and the reaction was monitored by crude ^{1}H NMR until completion. After the reaction was completed (about 10 h), the flask underwent rotary evaporation at room temperature until most of the acetone was removed. The reaction mixture was purified by chromatography on silica gel (gradient elution: 30/1→20/1 Hexane/EtOAc), colorless liquid, TLC R_f = 0.39 (4/1 Hexane/EtOAc). The fractions containing product were collected and concentrated by rotary evaporation at room temperature to afford the azide **1i** (310 mg, 90%). ^{1}H NMR (250 MHz, $CDCl_3$): δ 7.35 (s, 5H), 4.47 (s, 2H), 4.09 (q, J = 7.3 Hz, 2H), 3.52 (t, J = 7.3 Hz, 2H), 2.56 (t, J = 7.3 Hz, 2H), 1.22 (t, J = 7.3 Hz, 3H). ^{13}C NMR (62.9 MHz, $CDCl_3$): δ 170.8, 134.4, 128.9, 128.5, 128.5, 60.9, 53.3, 44.3, 33.0, 14.1. IR (neat, cm^{-1}): 2127(N_3), 1732, 1380, 1193, 1164, 770, 736, 698.

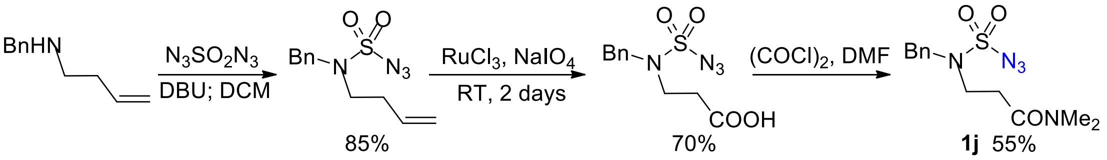

N-Benzyl-2-(vinyl)ethan-1-sulfamoyl azide was obtained in 85% yield through General Method from the commercially available amine (*N*-benzylbut-3-en-1-amine, cas: 17150-62-8). Purified by chromatography on silica gel (16/1 Hexane/EtOAc), colorless oil, TLC R_f = 0.5 (8/1 Hexane/EtOAc). ^{1}H NMR (500 MHz, $CDCl_3$): δ 7.64 -7.29 (m, 5H), 5.78-5.58 (m, 1H), 5.17-5.00 (m, 2H), 4.49 (s, 2H), 3.41-3.19 (m, 2H), 2.43-2.22 (m, 2H). ^{13}C NMR (125 MHz, $CDCl_3$): δ 134.6, 133.8, 128.9, 128.5, 117.8, 52.6, 47.9, 31.9. IR (neat, cm^{-1}): 2122(N_3), 1377, 1204, 1165, 923, 763, 735, 698.

To a solution of *N*-benzyl-2-(vinyl)ethan-1-sulfamoyl azide (1.6 g, 6 mmol) in CH_3CN (3 mL), CCl_4 (4 mL) and H_2O (4 mL) was added sodium periodate (2.6 g, 12 mmol) and ruthenium(III) chloride hydrate (60 mg, 0.25 mmol). The reaction mixture was stirred for 48h at room temperature until the consumption of starting material based on TLC. After addition of 50 ml of EtOAc, the reaction mixture was washed by water (80 mL) and the aqueous layer was extracted by EtOAc (3 x 40 mL). The combined organic layer was dried over anhydrous sodium sulfate. The solvent was

removed by vacuum and the residue was purified by chromatography on silica gel (3/1 to 1/1 Hexane/EtOAc) to give the desired product carboxylic acid containing sulfamoyl azide as yellow wax (1.2 g, 70% yield), TLC R_f = 0.3 (1/1 Hexane/EtOAc). ^{1}H NMR (500 MHz, $CDCl_3$): δ 7.50-7.32 (m, 5H), 4.50 (s, 2H), 3.54 (t, J = 7.3 Hz, 2H), 2.73-2.56 (m, 2H). ^{13}C NMR (125 MHz, $CDCl_3$): δ 176.3, 134.3, 129.1, 128.7, 128.5, 53.6, 44.0, 32.7. IR (neat, cm^{-1}): 2129(N_3), 1712, 1378, 1266, 1195, 1166, 733, 700.

To a solution of the above carboxylic acid containing sulfamoyl azide (284 mg, 1 mmol) in DCM (10 mL) at 0 ˚C, was added oxalyl chloride (0.17 mL, 2 mmol) and DMF (0.1 mL). The reaction mixture was stirred at 0 ˚C for 2 hours then at room temperature for 3 hours. The solvent was removed and the residue was purified by chromatography on silica gel (gradient elution: 3/1→2/1 Hexane/EtOAc) to give the desired product (**1j**) (171 mg, 55%), colorless liquid, TLC R_f = 0.5 (2/1 Hexane/EtOAc). ^{1}H NMR (400 MHz, $CDCl_3$): 7.41-7.33 (m, 5H), 4.52 (s, 2H), 3.60 (t, J = 7.0 Hz, 2H), 2.90 (s, 6H), 2.55 (t, J = 7.0 Hz, 2H). ^{13}C NMR (125 MHz, $CDCl_3$): δ 169.9, 134.9, 128.9, 128.6, 128.4, 53.9, 45.4, 37.0, 35.3, 32.3. IR (neat, cm^{-1}): 2126(N_3), 1638, 1377, 1166, 1024, 907, 726.

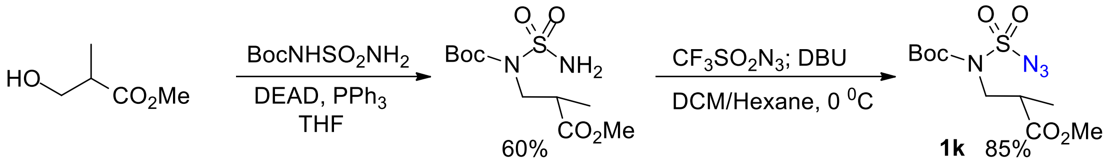

N-Boc-protected sulfamide was prepared according to the method described in the literature.[7] Diethylazodicarboxylate (0.63 mL, 4.0 mmol, 1.3 equiv) was added dropwise to an ice-cold mixture of Methyl 3-hydroxy-2-methylpropionate (354 mg, 3.0 mmol), $BocNHSO_2NH_2$ (0.79 g, 4.0 mmol, 1.3 equiv), and PPh_3 (1.05 g, 4.0 mmol, 1.3 equiv) in THF (15 mL). The solution was warmed to 23 °C and the mixture was stirred for about 6 h until TLC showed complete consumption of the starting alcohol. After this time, all volatile materials were removed under reduced pressure. The product was purified by chromatography on silica gel (4/1 Hexanes/EtOAc), to give *N*-Boc-protected sulfamide as a white solid (530 mg, yield: 60 %). ^{1}H NMR (400 MHz, $CDCl_3$): δ 5.40 (brs, 2H), 3.98 (dd, J = 14.8, 8.8 Hz, 1H), 3.77 (dd, J = 14.8, 5.2 Hz, 1H), 3.71 (s, 3H), 2.90-2.75 (m, 1H), 1.54 (s, 9H), 1.16 (d, J = 6.8 Hz, 3H).

To a solution of the above obtained *N*-Boc-protected sulfamide ester (60 mg, 0.2 mmol) and DBU (46 mg, 0.3 mmol) in DCM at 0 ^{0}C, a solution of $CF_3SO_2N_3$ (about 0.5 mmol) in hexane was added dropwise. The reaction showed almost complete consumption of the starting sulfamide (about 5 min.) when monitored by TLC. Then the majority of the DCM was removed carefully under reduced pressure at room temperature. Purification of this mixture by chromatography on silica gel (10:1 Hexanes/EtOA) afforded the corresponding *N*-Boc-protected sulfamoyl azide (**1k**) in 85% yield (55 mg), colorless liquid, TLC R_f = 0.36 (4/1 Hexanes/EtOAc). ^{1}H NMR (400 MHz, $CDCl_3$): δ 4.00 (dd, J = 7.6, 14.8 Hz, 1H), 3.76 (dd, J = 7.2, 14.8 Hz, 1H), 3.67 (s, 3H), 2.93-2.81 (m, 1H), 1.53 (s, 9H), 1.56 (d, J = 7.2 Hz, 3H). ^{13}C NMR (100

[7] Kurokawa, T.; Kim, M.; Du Bois, J. *Angew. Chem., Int. Edit.* **2009**, *48*, 2777.

MHz, $CDCl_3$): δ 174.0, 150.5, 86.4, 51.9, 51.0, 39.1, 27.9, 14.4. IR (neat, cm^{-1}): 2150(N_3), 1734, 1396, 1371, 1254, 1176, 1139, 1009, 756, 722.

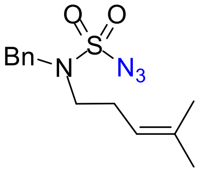

1l

N-Benzyl-4-methylpent-3-en-1-sulfamoyl azide (**1l**) was obtained in 95% yield through General Method from *N*-benzyl-4-methylpent-3-en-1-amine which was prepared according to the reference.[8] Purified by chromatography on silica gel (gradient elution: 30/1→20/1 Hexane/EtOAc), colorless liquid, TLC R_f = 0.52 (10/1 Hexane/EtOAc). ^{1}H NMR (250 MHz, $CDCl_3$): δ 7.37-7.31 (m, 5H), 4.99-4.91 (m, 1H), 4.46 (s, 2H), 3.20-3.13 (m, 2H), 2.22 (q, *J* = 7.5 Hz, 2H), 1.65 (s, 3H), 1.53 (s, 3H). ^{13}C NMR (62.9 MHz, $CDCl_3$): δ 135.1, 134.6, 128.8, 128.5, 128.4, 119.1, 52.5, 48.1, 26.4, 25.6, 17.6. IR (neat, cm^{-1}): 2123(N_3), 1455, 1380, 1207, 1165, 778, 735, 698.

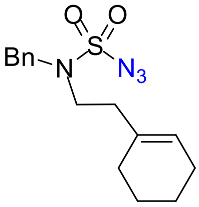

1m

N-Benzyl-2-(cyclohex-1-en-1-yl)ethan-sulfamoyl azide (**1m**) was obtained in 95% yield through General Method from *N*-benzyl-2-(cyclohex-1-en-1-yl) ethanamine which was prepared according to the reference.[9] Purified by chromatography on silica gel (gradient elution: 30/1→20/1 Hexane/EtOAc), colorless liquid, TLC R_f = 0.52 (10/1 Hexane/EtOAc). ^{1}H NMR (250 MHz, $CDCl_3$): δ 7.37 (brs, 5H), 5.40 (brs, 1H), 4.47 (s, 2H), 3.28 (t, *J* = 8.0 Hz, 2H), 2.17 (t, *J* = 7.8 Hz, 2H), 1.94 (brs, 2H), 1.83 (brs, 2H), 1.62-1.46 (m, 4H). ^{13}C NMR (62.9 MHz, $CDCl_3$): δ 134.6, 133.4, 128.7, 128.4, 128.3, 124.0, 52.2, 47.1, 35.7, 27.9, 25.1, 22.6, 22.0. IR (neat, cm^{-1}): 2122(N_3), 1381, 1205, 1165, 768, 735, 698.

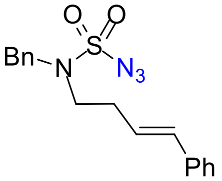

1n

N-Benzyl-2-(phenyl-vinyl)ethan-sulfamoyl azide (**1n**) was obtained in 75% yield through General Method from (*E*)-*N*-benzyl-4-phenylbut-3-en-1-amine which was

[8] Ahmed, S. Baker, L. A. Grainger, R. S. Innocenti, P. Quevedo, C. E. *J. Org. Chem.*, **2008**, *73*, 8116.
[9] Shao, Z.; Chen, J.; Tu, Y.; Li, L.; Zhang, H. *Chem. Comm.* **2003**, *15*, 1918.

prepared according to the reference.[10] Purified by chromatography on silica gel (16/1 Hexane/EtOAc), colorless oil, TLC R_f = 0.5 (10/1 Hexane/EtOAc). ^{1}H NMR (400 MHz, $CDCl_3$): δ 7.42-7.34 (m, 5H), 7.29 (d, *J* = 4.4 Hz, 4H), 7.26-7.20 (m, 1H), 6.38 (d, *J* = 15.6 Hz, 1H), 6.01 (dq, *J* = 16.0, 7.2 Hz, 1H), 4.51 (s, 2H), 3.37 (q, *J* = 7.2 Hz, 2H), 2.46 (dq, *J* = 1.2, 7.2 Hz, 2H). ^{13}C NMR (100 MHz, $CDCl_3$): δ 136.9, 134.5, 132.9, 128.9, 128.5, 128.5, 127.4, 126.1, 125.2, 52.9, 48.3, 31.4. IR (neat, cm^{-1}): 2126(N_3), 1380, 1265, 1166, 907, 729, 701.

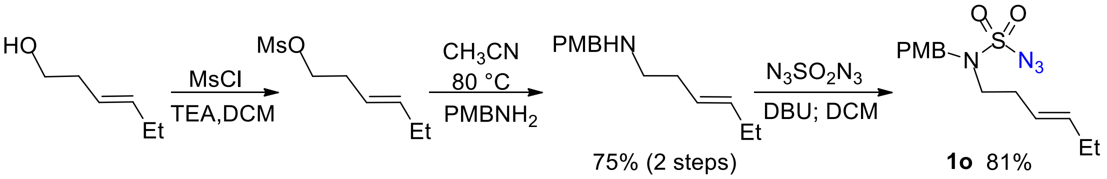

To a round bottom flask containing (*E*)-hex-3-en-1-ol (500 mg, 5 mmol) in DCM (15 mL) was added methanesulfonyl chloride (0.58 mL, 7.5 mmol) followed by the addition of triethyl amine (1.41 mL, 10 mmol), the precipitate was formed immediately. The reaction mixture was stirred at room temperature for about 2 hours until the alcohol was fully consumed based on TLC. DCM (30 mL) was added into the reaction mixture and washed by water (50 mL). The aqueous solution was extracted by DCM (3 x 20 mL). The combined organic layer was dried over anhydrous sodium sulfate. The solvent was removed and CH_3CN (20 mL) was added, followed by the addition of (4-methoxyphenyl) methanamine (1.37 g, 10 mmol). The reaction was heated at 80 ˚C for 6 hours. Then the solvent was removed and the residue was purified by flash silica gel chromatography (2/1 to 1/1 Hexane/EtOAc) to give (*E*)-*N*-(4-methoxybenzyl)hex-3-en-1-amine (820 mg, 75% yield), which were used directly for the next step.

(E)-*N*-(4-methoxybenzyl)hex-3-en-1-sulfamoyl azide (**1o**) was obtained in 81% yield through General Method from (*E*)-*N*-(4-methoxybenzyl)hex-3-en-1-amine. Purified by chromatography on silica gel (16/1 Hexane/EtOAc), white solid, TLC R_f = 0.5 (10/1 Hexane/EtOAc). ^{1}H NMR (500 MHz, $CDCl_3$): δ 7.26 (d, *J* = 8.5 Hz, 2H), 6.90 (d, *J* = 9.0 Hz, 2H), 5.51 (dt, *J* = 15.5, 6.5 Hz, 1H), 5.24 (dtt, *J* = 15.5, 7.0, 1.5 Hz, 1H), 4.41 (s, 2H), 3.82 (s, 3H), 3.21 (t, *J* = 7.5 Hz, 2H), 2.23 (dq, *J* = 1.0, 7.5 Hz, 2H), 2.02-1.94 (m, 2H), 0.95 (t, *J* = 7.5 Hz, 3H). ^{13}C NMR (125 MHz, $CDCl_3$): δ 159.7, 135.5, 130.0, 126.5, 124.0, 114.2, 55.3, 52.0, 48.1, 30.8, 25.6, 13.6. IR (neat, cm^{-1}): 2125(N_3), 1514, 1378, 1250, 1166, 906, 728. 600.

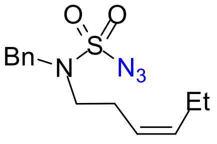

1p

(Z)-*N*-benzylhex-3-en-1-sulfamoyl azide (**1p**) was obtained in 97% yield through General Method from (Z)-*N*-benzylhex-3-en-1-amine which was prepared according

[10] Scheideman, M.; Shapland, P.; Vedejs, E. *J. Am. Chem. Soc.* **2003**, *125*, 10502.

to the reference.[11] Purified by chromatography on silica gel (gradient elution: 30:1→20:1 Hexane/EtOAc), colorless liquid, TLC R_f = 0.57 (10/1 Hexane/EtOAc). ^{1}H NMR (250 MHz, $CDCl_3$): δ 7.35 (brs, 5H), 5.51-5.39 (m, 1H), 5.23-5.10 (m, 1H), 4.46 (s, 2H), 3.22-3.15 (m, 2H), 2.26 (q, J = 7.8 Hz, 2H), 2.00-1.87 (m, 2H), 0.91 (t, J = 7.5 Hz, 3H). ^{13}C NMR (62.9 MHz, $CDCl_3$): δ 135.0, 134.5, 128.9, 128.5, 128.4, 123.4, 52.6, 48.1, 25.5, 20.5, 14.1. IR (neat, cm^{-1}): 2123(N_3), 1456, 1382, 1205, 1166, 790, 765, 736, 698.

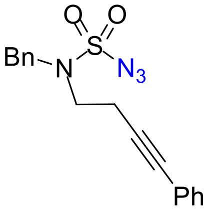

1q

N-Benzyl-4-phenylbut-3-yn-1-sulfamoyl azide (**1q**) was obtained in 75% yield through General Method from *N*-benzyl-4-phenylbut-3-yn-1-amine which was prepared according to the reference.[12] Purified by chromatography on silica gel (16/1 Hexane/EtOAc), white solid, TLC R_f = 0.55 (10/1 Hexane/EtOAc). ^{1}H NMR (400 MHz, $CDCl_3$): δ 7.43-7.28 (m, 10H), 4.63 (s, 2H), 3.50 (t, J = 7.2 Hz, 2H), 2.69 (t, J = 7.2 Hz, 2H). ^{13}C NMR (100 MHz, $CDCl_3$): δ 134.5, 131.5, 129.0, 128.6, 128.5, 128,3, 128.1, 123.0, 85.6, 82.8, 53.2, 47.1, 19.2. IR (neat, cm^{-1}): 2125(N_3), 1491, 1380, 1203, 1165, 907, 728, 692.

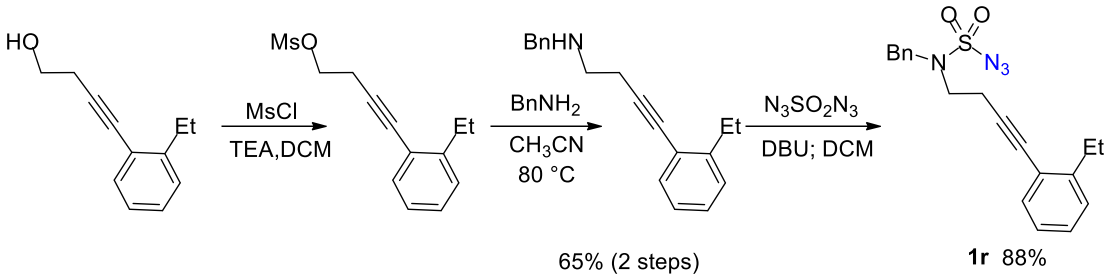

To a round bottom flask 4-(2-ethylphenyl)but-3-yn-1-ol (870 mg, 5 mmol) (commercially available, cas: 1417459-62-1) in DCM (15 mL) was added methanesulfonyl chloride (0.58 mL, 7.5 mmol) followed by the addition of triethyl amine (1.41 mL, 10 mmol), the precipitate was formed immediately. The reaction mixture was stirred at room temperature for about 2 hours until the alcohol was fully consumed based on TLC. DCM (30 mL) was added into the reaction mixture and washed by water (50 mL). The aqueous solution was extracted by DCM (3 x 20 mL). The combined organic layer was dried over anhydrous sodium sulfate. The solvent was removed and CH_3CN (20 mL) was added, followed by the addition of benzylamine (1.07 g, 10 mmol). The reaction was heated at 80 °C for 6 hours. Then the solvent was removed and the residue was purified by flash silica gel

[11] Asensio, G.; Mello, R.; Boix-Bernardini, C.; Gonzalez-Nunez, M. E.; Castellano, G. *J. Org. Chem.* **1995**, *60*, 3692.
[12] Hess, W.; Burton, J. W. *Chem. Eur. J.* **2010**, *16*, 12303.

chromatography (2/1→1/1 Hexane/EtOAc) to give *N*-benzyl-4-(2-ethylphenyl) but-3-yn-1-amine (850 mg, 65% yield). ^{1}H NMR (500 MHz, $CDCl_3$): δ 7.47-7.32 (m, 5H), 7.32-7.26 (m, 1H), 7.24-7.18 (m, 2H), 7.16-7.10 (m, 1H), 3.93-3.85 (m, 2H), 2.97-2.90 (m, 2H), 2.85-2.76 (m, 2H), 2.71 (t, J = 6.1 Hz, 2H), 1.95 (brs, 1H), 1.23 (dt, J = 1.5, 7.6 Hz, 3H). ^{13}C NMR (125 MHz, $CDCl_3$): δ 146.0, 140.1, 132.2, 128.5, 128.2, 128.0, 127.8, 127.0, 125.5, 122.7, 91.3, 80.5, 53.5, 47.8, 27.7, 20.7, 14.8.

N-benzyl-4-(2-ethylphenyl)but-3-yn-1-sulfamoyl azide (**1r**) was obtained in 88% yield through General Method from *N*-benzyl-4-(2-ethylphenyl)but-3-yn-1-amine. Purified by chromatography on silica gel (16/1 Hexane/EtOAc), colorless oil, TLC R_f = 0.5 (8/1 Hexane/EtOAc). ^{1}H NMR (500 MHz, $CDCl_3$): δ 7.41-7.34 (m, 6H), 7.27-7.19 (m, 2H), 7.13 (dt, J = 1.0, 7.5 Hz, 1H), 4.63 (s, 2H), 3.51 (t, J = 7.5 Hz, 2H), 2.78 (q, J = 7.5 Hz, 2H), 2.73 (t, J = 7.5 Hz, 2H), 1.23 (t, J = 7.5 Hz, 3H). ^{13}C NMR (125 MHz, $CDCl_3$): δ 146.1, 134.5, 132.2, 129.0, 128.6, 128.5, 128.3, 127.9, 125.6, 122.0, 88.7, 81.4, 53.2, 47.3, 27.5,19.3, 14.8. IR (neat, cm^{-1}): 2124(N_3), 1455, 1380, 1204, 1165, 986, 940, 754, 698.

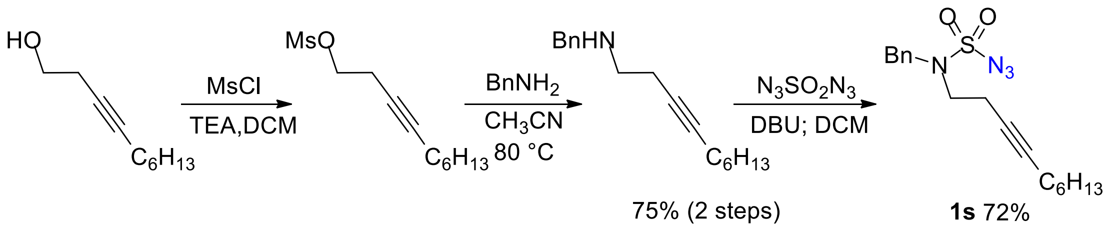

To a round bottom flask dec-3-yn-1-ol (0.31 g, 2 mmol) (commercially available, cas: 51721-39-2) in DCM (15 mL) was added methanesulfonyl chloride (0.34 g, 3 mmol) followed by the addition of triethyl amine (0.61 g, 6 mmol), the precipitate was formed immediately. The reaction mixture was stirred at room temperature for about 2 hours until the alcohol was fully consumed based on TLC. DCM (30 mL) was added into the reaction mixture and washed by water (50 mL). The aqueous solution was extracted by DCM (3 x 20 mL). The combined organic layer was dried over anhydrous sodium sulfate. The solvent was removed and CH_3CN (20 mL) was added, followed by the addition of benzylamine (0.43 g, 4 mmol). The reaction was heated at 80° C for 6 hours. Then the solvent was removed and the residue was purified by flash silica gel chromatography (2/1 to 1/1 Hexane/EtOAc) to give *N*-benzyldec-3-yn-1-amine (364 mg, 75% yield), which were used directly for the next step. ^{1}H NMR (500 MHz, $CDCl_3$): δ 7.36-7.29 (m, 4H), 7.28-7.21 (m, 1H), 3.82 (s, 2H), 2.76 (t, J = 6.5 Hz, 2H), 2.41-2.36 (m, 2H), 2.17-2.12 (m, 2H), 1.67 (brs, 1H), 1.50-1.43 (m, 2H), 1.39-1.19 (m, 6H), 0.88 (t, J = 7.0 Hz, 3H). ^{13}C NMR (125 MHz, $CDCl_3$): δ 140.3, 128.3, 128.4, 126.9, 81.8, 77.7, 53.4, 47.9, 31.3, 29.0, 28.5, 22.5, 19.8, 18.7, 14.0.

N-Benzyldec-3-yn-1-sulfamoyl azide (**1s**) was obtained in 72% yield through General Method from *N*-benzyldec-3-yn-1-amine. Purified by chromatography on silica gel (16/1 Hexane/EtOAc), colorless oil, TLC R_f = 0.4 (8/1 Hexane/EtOAc). ^{1}H NMR (500 MHz, $CDCl_3$): δ 7.41-7.33 (m, 5H), 4.58 (s, 2H), 3.36 (t, J = 7.5 Hz, 2H),

2.43 (tt, J = 2.5, 7.5 Hz, 2H), 2.11 (tt, J = 2.5, 7.0 Hz, 2H), 1.50-1.43 (m, 2H), 1.39-1.22 (m, 6H), 0.89 (t, J = 7.0 Hz, 3H). ^{13}C NMR (125 MHz, $CDCl_3$): δ 134.6, 128.9, 128.5, 128.4, 83.0, 75.7, 53.0, 47.5, 31.3, 28.8, 28.6, 22.5, 18.7, 18.5, 14.0. IR (neat, cm^{-1}): 2124(N_3), 1456, 1381, 1204, 1165, 754, 697, 594.

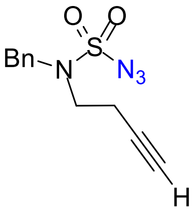

1t

N-Benzylbut-3-yn-1-sulfamoyl azide (**1t**) was obtained in 69% yield through General Method from *N*-benzylbut-3-yn-1-amine which was prepared according to the reference.[13] Purified by chromatography on silica gel (gradient elution: 30/1→20/1 Hexane/EtOAc), colorless liquid, TLC R_f = 0.37 (10/1 Hexane/EtOAc). ^{1}H NMR (250 MHz, $CDCl_3$): δ 7.36 (s, 5H), 4.56 (s, 2H), 3.39 (t, J = 7.3 Hz, 2H), 2.43 (dt, J = 2.8, 7.3 Hz, 2H), 2.03 (t, J = 2.8 Hz, 1H). ^{13}C NMR (125 MHz, $CDCl_3$): δ 134.4, 129.0, 128.6, 128.5, 80.2, 70.8, 53.2, 46.9, 18.2. IR (neat, cm^{-1}): 2128(N_3), 1381, 1265, 1198, 1167, 908, 731, 702.

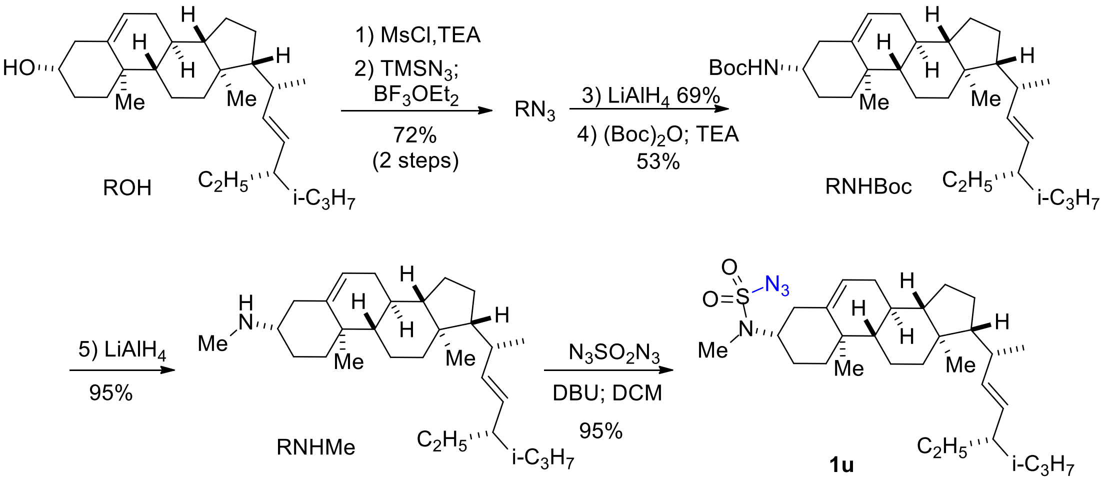

The stigmasterol-derivatived alkyl azide RN_3 (1.0 g, 2.3 mmol), which was obtained in 72% yield (2 steps) from commercial available stigmasterol (cas 83-48-7) according to the reference,[14] was dissolved into anhydrous THF (20 mL) at 0 °C. Lithium aluminium hydride (130 mg, 3.4 mmol) was added to this solution and the reaction mixture was slowly warmed to room temperature for overnight. After quenching the excess of $LiAlH_4$ following Fieser method, the reaction mixture was filtrated through celite. The filtrate was collected and the solvent was removed to give crude primary amine derivative (646 mg, 69%) as a white solid, which was used directly for Boc protection.

This crude amine compound (930 mg, 2.2 mmol) was dissolved into DCM (30 mL)

[13] Iyer, S.; Liebeskind, L. S. *J. Am. Chem. Soc.*, **1987**, *109*, 2759.
[14] Sun, Q.; Cai, S.; Peterson, B. R. *Org. Lett.* **2009**, *11*, 567.

at 0 ˚C, followed by the addition of $(Boc)_2O$ (740 mg, 3.4 mmol) and triethylamine (0.4 mL, 2.8 mmol). The reaction mixture was warmed up to room temperature and stirred for 3 hours. The solvent was removed and the residue was purified by chromatography on silica gel (16/1 Hexane/EtOAc), white solid, TLC R_f = 0.65 (8/1 Hexane/EtOAc) to give the Boc protected amine derivative as a white solid (602 mg, 53% yield). ^{1}H NMR (500 MHz, $CDCl_3$): δ 5.36-5.34 (m, 1H), 5.15 (dd, J = 15.0, 9.0 Hz, 1H), 5.01 (dd, J = 15.0, 8.5 Hz, 1H), 4.40 (brs, 1H), 3.35 (brs, 1H), 2.32 (d, J = 13.0 Hz, 1H), 2.07-1.93 (m, 4H), 1.88-1.81 (m, 2H), 1.73-1.66 (m, 1H), 1.59-0.93 (m, 20H), 1.44 (s, 9H), 1.02 (d, J = 6.5 Hz, 3H), 0.98 (s, 3H), 0.84 (d, J = 6.5 Hz, 3H), 0.82-7.87 (m, 6H).

To a solution of this Boc-protected amine (510 mg, 1 mmol) in THF (4 mL) at 0 ˚C was added lithium aluminium hydride (100 mg, 2.6 mmol). The reaction mixture was warmed to room temperature followed by heating to reflux for 24h. After quenching the reaction following Fieser method, the reaction mixture was filtrated through celite and concentrated to give *N*-methyl amine derivative (403 mg, 95%) as a white solid. ^{1}H NMR (500 MHz, $CDCl_3$): δ 5.34-5.32 (m, 1H), 5.16 (dd, J = 8.5, 15.0 Hz, 1H), 5.02 (dd, J = 8.5, 15.0 Hz, 1H), 2.43 (s, 3H), 2.32-2.23 (m, 2H), 2.09-1.95 (m, 4H), 1.88-1.39 (m, 11H), 1.32-0.92 (m, 10H), 1.03 (d, J = 6.5 Hz, 3H), 1.00 (s, 3H), 0.86 (d, J = 6.0 Hz, 3H), 0.81 (t, J = 7.5 Hz, 6H), 0.70 (s, 3H). ^{13}C NMR (125 MHz, $CDCl_3$): δ 141.7, 138.3, 129.2, 120.7, 60.0, 56.9, 55.9, 51.2, 50.3, 42.2, 40.5, 39.7, 39.7, 38.0, 37.1, 33.6, 31.9, 29.1, 28.9, 25.4, 24.3, 21.2, 21.1, 21.0, 19.4, 19.0, 12.2, 12.0.

Sulfamoyl azide (**1u**) was obtained in 95% yield through General Method from the corresponding methyl amine derivative. Purified by chromatography on silica gel (16/1 Hexane/EtOAc), white solid, TLC R_f = 0.75 (8/1 Hexane/EtOAc). ^{1}H NMR (400 MHz, $CDCl_3$): δ 5.39 (s, 1H), 5.16 (dd, J = 15.2, 8.8 Hz, 1H), 5.02 (dd, J = 15.2, 8.8 Hz, 1H), 3.79-3.67 (m, 1H), 2.91 (s, 3H), 2.47 (t, J = 12.8 Hz, 1H), 2.14 (d, J = 13.2 Hz, 1H), 2.03-1.90 (m, 4H), 1.76-1.71 (m, 3H), 1.58-1.40 (m, 8H), 1.31-1.09 (m, 5H), 1.09-0.93 (m, 3H), 1.02 (d, J = 6.4 Hz, 3H), 1.00 (s, 3H), 0.86-0.79 (m, 9H), 0.70 (s, 3H). ^{13}C NMR (100 MHz, $CDCl_3$): δ 139.4, 138.2, 129.3, 122.9, 59.5, 56.7, 55.9, 51.2, 50.0, 42.2, 40.5, 39.6, 37.8, 36.5, 35.5, 31.9, 31.8, 31.8, 30.0, 28.9, 25.6, 25.4, 24.3, 21.2, 21.1, 21.0, 19.3, 19.0, 12.2, 12.0. IR (neat, cm^{-1}): 2944, 2129(N_3), 1460, 1376, 1265, 1207, 1158, 971, 949, 782, 734, 607.

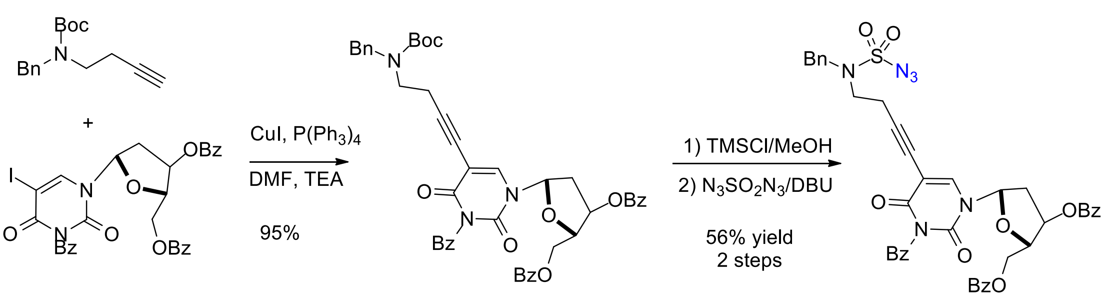

1v

To a solution of 3-*N*-benzoyl 3’5’-di-*O*-benzoyl-5-iodo-2’-deoxyuridine (1.0 g, 1.53 mmol) in anhydrous DMF (10 mL) was added *tert*-butyl

benzyl(but-3-yn-1-yl)carbamate (800 mg, 3.06 mmol), triethylamine (0.42 mL, 3.06 mmol), $Pd(PPh_3)_4$ (105 mg, 0.09 mmol) and finally CuI (35 mg, 0.185 mmol). The reaction was stirred at room temperature for 24 h. The solution was concentrated in vacuo and the residue was purified by silica gel column chromatography (7/1 Hexanes/EtOAc) to afford the product, R_f = 0.42 (1/1 EtOAc/Hexanes) (812 mg, 68% yield) as yellow solid. ^{1}H NMR (500 MHz, $CDCl_3$): δ 8.11-8.01 (m, 4H), 7.92 (d, *J* = 7.8 Hz, 2H), 7.86 (brs, 1H), 7.70-7.64 (m, 1H), 7.63-7.54 (m, 2H), 7.48 (td, *J* = 7.8, 19.6 Hz, 7H), 7.36-7.29 (m, 2H), 7.26-7.16 (m, 2H), 6.39 (dd, *J* = 5.4, 8.3 Hz, 1H), 5.64 (d, *J* = 6.4 Hz, 1H), 4.85-4.71 (m, 2H), 4.61 (brs, 1H), 4.54-4.46 (m, 2H), 3.32 (brs, 1H), 3.23 (brs, 1H), 2.80 (d, *J* = 11.7 Hz, 1H), 2.46 (d, *J* = 18.1 Hz, 2H), 2.42-2.30 (m, 2H), 1.49 (brs, 5H), 1.44 (brs, 4H). HRMS (ESI) ($[M+Na]^+$) Calcd. for $C_{46}H_{43}N_3NaO_{10}^+$: 820.2841, Found: 820.2855.

The de-protection of the Boc-substituted substrate was done as previously. The Boc-protected amine (500 mg, 0.62 mmol) was set to stir in MeOH (10 mL) and DCM (5 mL) in a round-bottom flask and put under a nitrogen atmosphere. The stirring solution was then cooled to 0 °C in an ice bath and then TMSCl (1.26 mL, 10 mmol) was added slowly over the course of ~30 minutes. The reaction was allowed to slowly warm to room temperature and left to react for a subsequent 3 hours. After the course of the reaction had completed, all volatiles were removed under reduced pressure. The non-volatile products dissolved in DCM (20 mL), Et_3N (2.0 ml) and brine (20 mL), then extracted 2 x 20 mL with DCM. The organic extracts were dried over Na_2SO_4, filtered, and reduced under a vacuum with a Rotavap® to give crude amine (350 mg, yield: about 80%) which was used directly in the next step (Note, compound is unstable and need to be converted immediately).

Deoxyuridine-based sulfamoyl azide (**1v**) was obtained in (270 mg) 65% yield through General Method from the above obtained amine derivative. Purified by chromatography on silica gel (40/1 DCM/EtOAc), white solid, TLC R_f = 0.7 (20/1 DCM/EtOAc). ^{1}H NMR (400 MHz, $CDCl_3$): δ 8.07-8.01 (m, 4H), 7.92-7.89 (m, 3H), 7.68-7.56 (m, 3H), 7.50-7.43 (m, 6H), 7.38-7.30 (m, 5H), 6.39 (dd, *J* = 8.0, 5.6 Hz, 1H), 5.64 (d, *J* = 6.4 Hz, 1H), 4.75 (dq, *J* = 3.6, 12.4 Hz, 2H), 4.60 (dd, *J* = 5.6, 3.2 Hz, 1H), 4.54 (s, 2H), 3.31 (t, *J* = 7.2 Hz, 2H), 2.81 (ddd, *J* = 14.4, 5.6, 1.6 Hz, 1H), 2.50-2.46 (m, 2H), 2.43-2.34 (m, 1H). ^{13}C NMR (100 MHz, $CDCl_3$): δ 167.6, 165.9, 165.8, 160.3, 148.2, 140.9, 135.3, 134.4, 133.7, 133.6, 131.0, 130.5, 129.7, 129.5, 129.2, 129.1, 128.9, 128.8, 128.7, 128.6, 128.5, 100.7, 91.1, 86.1, 83.3, 74.8, 72.8, 64.2, 53.2, 46.7, 38.6, 19.3. IR (neat, cm^{-1}): 2130(N_3), 1755, 1714, 1673, 1378, 1264, 1168, 1096, 733, 704.

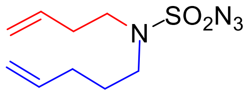

1w

N-homoallylic-*N*-bishomoallylic sulfamoyl azide (**1w**) was obtained in 85% yield through General Method from *N*-homoallylic-*N*-bishomoallylic amine which was prepared according to the reference.[15] Purified by chromatography on silica gel

[15] Paquette, L. A.; Dura, R. D.; Fosnaugh, N.; Stepanian, M. *J. Org. Chem.*, **2006**, *71*, 8438.

(gradient elution: 20/1→10/1 Hexanes/EtOAc), colorless liquid, TLC R_f = 0.54 (10:1 Hexanes/EtOAc). ^{1}H NMR (250 MHz, $CDCl_3$): δ 5.84-5.64 (m, 2H), 5.15-4.97 (m, 4H), 3.33-3.20 (m, 4H), 2.35 (q, J = 7.5 Hz, 2H), 2.06 (q, J = 7.3 Hz, 2H), 1.77-1.64 (m, 2H). ^{13}C NMR (62.9 MHz, $CDCl_3$): δ 136.8, 133.7, 117.7, 115.7, 48.8, 48.6, 32.3, 30.4, 26.9. IR (neat, cm^{-1}): 2121(N_3), 1381, 1206, 1158, 992, 917, 733.

General Procedure for C–H Amination of Sulfamoyl Azides

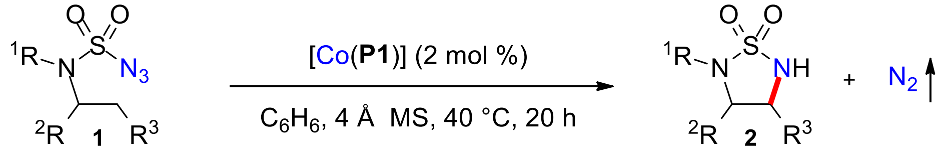

An oven dried Schlenk tube was charged with catalyst (0.002 mmol) and 4Å MS (50 mg), then evacuated and back filled with nitrogen. The Teflon screw cap was replaced with a rubber septum and then an approximately 0.5 ml portion of benzene was added, then azide (0.1 mmol), followed by the remaining benzene (total 1 mL). The Schlenk tube was then purged with nitrogen for 2 minutes and the rubber septum was replaced with a Teflon screw cap. The Schlenk tube was then placed in an oil bath for the desired time and at 40 ˚C. After completion of the reaction, the reaction mixture was purified by flash column chromatography. The fractions containing product were collected and concentrated by rotary evaporation to afford the target compound.

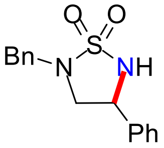
2a

2-Benzyl-4-phenyl-1,2,5-thiadiazolidine 1,1-dioxide (**2a)** was obtained in 90% yield through General Procedure for C–H Amination of Sulfamoyl Azides. Purified by chromatography on silica gel (gradient elution: 4:1→2:1 Hexane/EtOAc), colorless liquid, TLC R_f = 0.15 (10/1 Hexane/EtOAc). ^{1}H NMR (500 MHz, $CDCl_3$): δ 7.41-7.30 (m, 10H), 4.83-4.77 (m, 2H), 4.37, 4.00 (AB q, J = 13.5 Hz, each 1 H), 3.55 (dd, J = 8.0, 9.5 Hz, 1H), 3.12 (dd, J = 8.3, 9.8 Hz, 1H). ^{13}C NMR (125 MHz, $CDCl_3$): δ 138.4, 134.8, 129.0, 128.8, 128.7, 128.2, 126.4, 55.8, 55.0, 50.5. IR (neat, cm^{-1}): 1331, 1285, 1153, 1096, 1019, 753, 696, 684. HRMS (ESI) ([M+H]$^+$) Calcd. for $C_{15}H_{17}N_2O_2S^+$: 289.1005, Found: 289.0999.

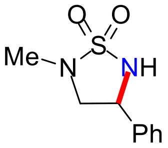
2b

2-Methyl-4-phenyl-1,2,5-thiadiazolidine 1,1-dioxide (**2b**) was obtained in 95% yield through General Procedure for C–H Amination of Sulfamoyl Azides. Purified by chromatography on silica gel (gradient elution: 2:1→1:1 Hexane/EtOAc), colorless liquid, TLC R_f = 0.29 (1/1 Hexane/EtOAc). ^{1}H NMR (400 MHz, $CDCl_3$): δ 7.43-7.32

(m, 5H), 4.82 (q, J = 7.2 Hz, 1H), 4.70 (br, 1H), 3.67 (dd, *J* = 7.2, 9.2 Hz, 1H), 3.21 (t, *J* = 8.8 Hz, 1H), 2.74 (s, 3H). ^{13}C NMR (100 MHz, $CDCl_3$): δ 138.5, 129.0, 128.8, 126.4, 57.8, 55.7, 32.8. IR (neat, cm^{-1}): 1341, 1321, 1281, 1190, 1154, 1104, 1026, 959, 933, 895, 697. HRMS (ESI) ($[M+H]^+$) Calcd. for $C_9H_{13}N_2O_2S^+$: 213.0692**,** Found: 213.0709.

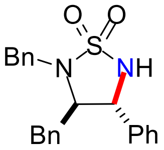

(X-Ray) **2c**

2,3-Dibenzyl-4-phenyl-1,2,5-thiadiazolidine 1,1-dioxide (**2c**) was obtained in 92% yield through General Procedure for C–H Amination of Sulfamoyl Azides. Purified by chromatography on silica gel (4:1 Hexane/EtOAc), white solid, TLC R_f = 0.35 (4/1 Hexane/EtOAc). ^{1}H NMR (500 MHz, $CDCl_3$): δ 7.32-7.19 (m, 11H), 7.08-7.03 (m, 4H), 4.81 (d, *J* = 6.0 Hz, 1H), 4.49 (t, *J* = 5.5 Hz, 1H), 4.33, 4.13 (AB q, *J* = 14.5 Hz, each 1H), 3.64-3.60 (m, 1H), 3.05 (dd, *J* = 6.0, 13.5 Hz, 1H), 2.99 (dd, *J* = 7.5, 13.5 Hz, 1H). ^{13}C NMR (125 MHz, $CDCl_3$): δ 139.0, 136.3, 134.9, 129.4, 128.9, 128.7, 128.6, 128.2, 128.0, 127.0, 126.5, 67.8, 60.4, 50.4, 38.8. IR (neat, cm^{-1}): 1496, 1455, 1265, 1160, 1029, 733, 699. HRMS (ESI) ($[M+Na]^+$) Calcd. for $C_{22}H_{22}N_2NaO_2S^+$: 401.1294**,** Found: 401.1292.

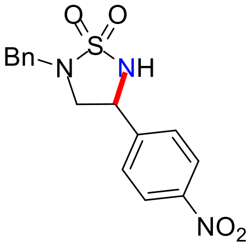

2d

2-Benzyl-4-(4-nitrophenyl)-1,2,5-thiadiazolidine 1,1-dioxide (**2d**) was obtained in 81% yield through General Procedure for C–H Amination of Sulfamoyl Azides. Purified by chromatography on silica gel (3/1 Hexane/EtOAc), white solid, TLC R_f = 0.25 (1/1 Hexane/EtOAc). ^{1}H NMR (500 MHz, CD_3OD): δ 8.25 (d, *J* = 8.5 Hz, 2H), 7.71 (d, *J* = 8.5 Hz, 2H), 7.41-7.32 (m, 5H), 4.97 (t, *J* = 7.5 Hz, 2H), 4.31, 3.99 (AB q, *J* = 13.5 Hz, each 1H), 3.80 (t, *J* = 8.5 Hz, 1H), 3.00 (t, *J* = 8.5 Hz, 1H). ^{13}C NMR (125 MHz, CD_3OD): δ 147.7, 147.6, 135.3, 128.4, 128.3, 127.7, 127.2, 123.4, 54.3, 54.2, 50.0. IR (neat, cm^{-1}): 1600, 1518, 1348, 1298, 1283, 1265, 1159, 1091, 849, 736, 700. HRMS (ESI) ($[M+Na]^+$) Calcd. for $C_{15}H_{15}N_3NaO_4S^+$: 356.0676, Found: 356.0676.

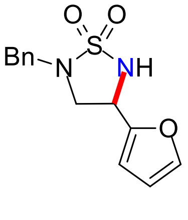

2e

2-Benzyl-4-(furan-2-yl)-1,2,5-thiadiazolidine 1,1-dioxide (**2e)** was obtained in 80% yield through General Procedure for C–H Amination of Sulfamoyl Azides. Purified by chromatography on silica gel (4/1 Hexane/EtOAc), white solid, TLC R_f = 0.35 (4/1 Hexane/EtOAc). ^{1}H NMR (500 MHz, $CDCl_3$): δ 7.41-7.32 (m, 6H), 6.38-6.33 (m, 2H), 4.86 (q, J = 7.0 Hz, 1H), 4.76 (d, J = 6.5 Hz, 1H), 4.34, 4.14 (AB q, J = 14.0 Hz, each 1H), 3.53-3.48 (m, 1H), 3.43 (t, J = 8.5 Hz, 1H). ^{13}C NMR (125 MHz, $CDCl_3$): δ 150.0, 143.3, 134.9, 128.8, 128.6, 128.2, 110.7, 108.5, 52.1, 50.5, 49.7. IR (neat, cm^{-1}): 1332, 1304, 1265, 1166, 731, 699. HRMS (ESI) ($[M+Na]^+$) Calcd. For $C_{13}H_{14}N_2NaO_3S^+$: 301.0617, Found: 301.0610.

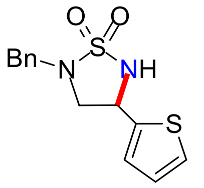

2f

2-Benzyl-4-(thiophen-2-yl)-1,2,5-thiadiazolidine 1,1-dioxide (**2f)** was obtained in 86% yield through General Procedure for C–H Amination of Sulfamoyl Azides. Purified by chromatography on silica gel (4/1 Hexane/EtOAc), white solid, TLC R_f = 0.30 (4/1 Hexane/EtOAc). ^{1}H NMR (500 MHz, $CDCl_3$): δ 7.40-7.29 (m, 5H), 7.29 (d, J = 5.0 Hz, 1H), 7.05 (d, J = 3.5 Hz, 1H), 6.97-6.95 (m, 1H), 5.06 (q, J = 7.0 Hz, 1H), 4.78 (d, J = 5.5 Hz, 1H), 4.32, 4.13 (AB q, J = 14.0 Hz, each 1H), 3.58 (dd, J = 7.0, 10.0 Hz, 1H), 3.30 (dd, J = 7.5 Hz, 10.0 Hz, 1H). ^{13}C NMR (125 MHz, $CDCl_3$): δ 141.2, 134.7, 128.8, 128.7, 128.3, 127.1, 126.3, 126.0, 55.1, 51.9, 50.4. IR (neat, cm^{-1}): 1455, 1387, 1367, 1300, 1285, 1265, 1155, 1126, 1017, 727. HRMS (ESI) ($[M+H]^+$) Calcd. For $C_{13}H_{15}N_2O_2S_2^+$: 295.0570, Found: 295.0570.

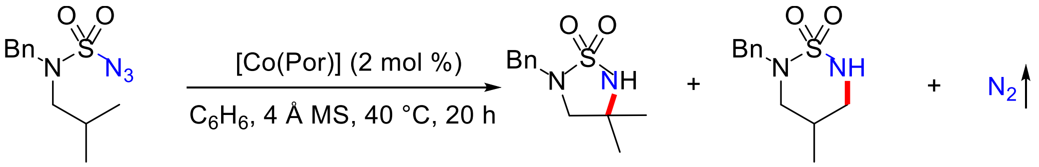

2g 53% **2g'** 37%

Products **2g** and **2g′** (inseparable) was obtained through General Procedure for C–H Amination of Sulfamoyl Azides. Yield: 90% overall yield (53% yield for **2g**, 37% yield for **2g′** based on the NMR ratio). Purified by chromatography on silica gel (4/1 Hexane/EtOAc), colorless oil, TLC R_f = 0.3 (4/1 Hexane/EtOAc). ^{1}H NMR of compound **2g** (500 MHz, $CDCl_3$): δ 7.37-7.30 (m, 5H), 4.24 (brs, 1H), 4.17 (s, 2H), 3.02 (s, 2H), 1.39 (s, 6H). ^{1}H NMR of compound **2g′** (500 MHz, $CDCl_3$): δ 7.37-7.30 (m, 5H), 4.44, 3.98 (AB q, J = 14.0 Hz, each 1H), 4.37-4.33 (m, 1H), 3.36-3.27 (m. 2H), 3.04-2.91 (m, 2H), 2.10-1.93 (m, 1H), 0.85 (d, J = 7.0 Hz, 3H). ^{13}C NMR of compounds **2g** and **2g′** (125 MHz, $CDCl_3$): δ 135.4, 135.2, 128.7, 128.6, 128.4, 128.1, 127.9, 59.9, 55.7, 54.8, 52.0, 51.3, 49.9, 29.1, 28.5, 14.8. IR of compounds **2g** and **2g′**(neat, cm^{-1}): 3259, 1307, 1157, 906 726, 697. HRMS of compounds **2g** and **2g′** (ESI) ($[M+H]^+$) Calcd. for $C_{11}H_{17}N_2O_2S^+$: 241.1005, Found: 241.1004.

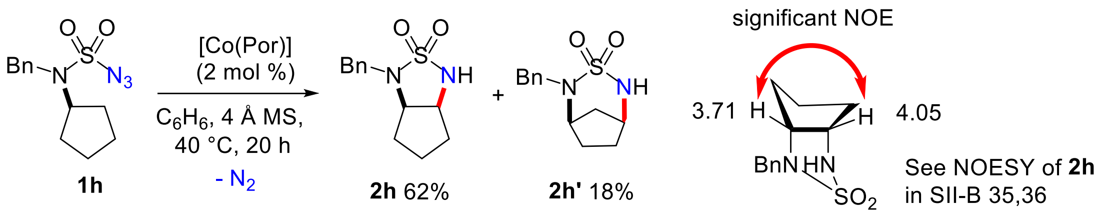

Product **2h** was obtained through General Procedure for C–H Amination of Sulfamoyl Azides. Yield: 62%. Purified by chromatography on silica gel (6/1 Hexane/EtOAc), colorless oil, TLC R_f = 0.25 (4/1 Hexane/EtOAc). ^{1}H NMR (500 MHz, $CDCl_3$): δ 7.40-7.29 (m, 5H), 4.36, 4.07 (AB q, *J* = 14.0 Hz, each 1H), 4.19 (d, *J* = 7.0 Hz, 1H), 4.05-4.00 (m, 1H), 3.71-3.67 (m, 1H), 1.96-1.88 (m, 1H), 1.86-1.75 (m, 1H), 1.72-1.66 (m, 1H), 1.56-1.43 (m, 3H). ^{13}C NMR (125 MHz, $CDCl_3$): δ 135.3, 129.0, 128.6, 128.0, 64.6, 56.1, 49.8, 33.3, 31.9, 23.2. IR (neat, cm^{-1}): 3249, 1295, 1161, 905, 726, 698. HRMS (ESI) ($[M+H]^+$) Calcd. for $C_{12}H_{17}N_2O_2S^+$: 253.1005, Found: 253.1005.

Product **2h′** was obtained through General Procedure for C–H Amination of Sulfamoyl Azides. Yield: 18%. Purified by chromatography on silica gel (4/1 Hexane/EtOAc), white solid, TLC R_f = 0.2 (4/1 Hexane/EtOAc). ^{1}H NMR (500 MHz, $CDCl_3$): δ 7.37-7.30 (m, 5H), 4.58, 3.78 (AB q, *J* = 14.0 Hz, each 1H), 4.37 (s, 1H), 3.98 (brs, 1H), 3.61 (t, *J* = 4.5 Hz, 1H), 2.61-2.52 (m, 2H), 2.07 (d, *J* = 12.5 Hz, 1H), 1.96-1.87 (m, 1H), 1.68-1.59 (m, 1H), 1.55-1.51 (m, 1H). ^{13}C NMR (125 MHz, $CDCl_3$): δ 135.7, 128.8, 128.6, 127.8, 60.1, 58.5, 50.2, 38.8, 27.5, 26.5. IR (neat, cm^{-1}): 3247, 2919, 1336, 1289, 1151, 1070, 732, 698. HRMS (ESI) ($[M+H]^+$) Calcd. for $C_{12}H_{17}N_2O_2S^+$: 253.1005, Found: 253.1000.

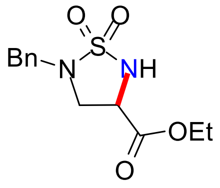

2i

Ethyl 5-benzyl-1,2,5-thiadiazolidine-3-carboxylate 1,1-dioxide (**2i**) was obtained in 89% yield with 5 mol% [Co(**P1**)] through General Procedure for C–H Amination of Sulfamoyl Azides. Purified by chromatography on silica gel (gradient elution: 4/1→2/1 Hexane/EtOAc), white solid, TLC R_f = 0.41 (1/1 Hexane/EtOAc). ^{1}H NMR (250 MHz, $CDCl_3$): δ 7.35-7.28 (m, 5H), 5.20 (s, br), 4.32-4.14 (m, 3H), 4.29, 3.96 (AB q, *J* = 13.8 Hz, each 1H), 3.43 (d, *J* = 1.8 Hz, 1H), 3.41 (s, 1H), 1.24 (t, *J* = 7.0 Hz, 3H). ^{13}C NMR (62.9 MHz, $CDCl_3$): δ 169.7, 134.4, 128.8, 128.5, 128.3, 63.1, 52.8, 50.3, 49.7, 14.0. IR (neat, cm^{-1}): 2922, 1739, 1458, 1225, 1167, 780, 737, 695. HRMS (ESI) ($[M+Na]^+$) Calcd. for $C_{12}H_{16}N_2O_4SNa^+$: 307.0723, Found: 307.0723.

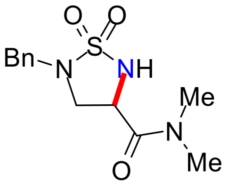

2j

5-Benzyl-*N*,*N*-dimethyl-1,2,5-thiadiazolidine-3-carboxamide 1,1-dioxide (**2j**) was obtained in 50% yield with 5 mol% [Co(**P1**)] through General Procedure for C–H Amination of Sulfamoyl Azides. Purified by chromatography on silica gel (gradient elution: 2/1→1/1 Hexane/EtOAc), white solid, TLC R_f = 0.2 (1/1 Hexane/EtOAc). ^{1}H NMR (400 MHz, $CDCl_3$): δ 7.37-7.31 (m, 5H), 5.71 (d, *J* = 7.6 Hz, 1H), 4.47-4.42 (m, 1H), 4.32, 4.05 (AB q, *J* = 13.6 Hz, each 1H), 3.39 (dd, *J* = 8.4, 9.6 Hz, 1H), 3.17 (dd, *J* = 4.8, 9.6 Hz, 1H), 3.00 (s, 3H), 2.91 (s, 3H). ^{13}C NMR (100 MHz, $CDCl_3$): δ 167.7, 134.9, 128.8, 128.6, 128.2, 50.9, 50.8, 36.3, 36.2, 29.7. IR (neat, cm^{-1}): 1650, 1455, 1370, 1327, 1260, 1168, 1094, 732, 698. HRMS (ESI) ($[M+Na]^+$) Calcd. for $C_{12}H_{17}N_3NaO_3S^+$: 306.0883, Found: 306.0890.

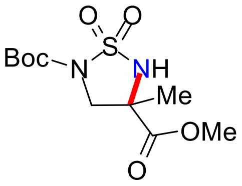

2k

2-Tert-butyl 4-methyl 4-methyl-1,2,5-thiadiazolidine-2,4-dicarboxylate 1,1-dioxide (**2k**) was obtained in 99% yield through General Procedure for C–H Amination of Sulfamoyl Azides. Purified by chromatography on silica gel (10:1 DCM/EtOAc), colorless liquid, TLC R_f = 0.70 (4/1 DCM/EtOAc); ^{1}H NMR (400 MHz, $CDCl_3$): δ 5.54 (s, 1H), 4.13 (d, *J* = 10.4 Hz, 1H), 3.84 (s, 3H), 3.75 (d, *J* = 10.4 Hz, 1H), 1.65 (s, 3H), 1.50 (s, 9H). ^{13}C NMR (100 MHz, $CDCl_3$): δ 171.4, 149.1, 85.0, 58.6, 54.1, 53.9, 27.9, 23.9. IR (neat, cm^{-1}): 2923, 2853, 1727, 1460, 1375, 1321, 1262, 1138, 1066, 808. HRMS (ESI) ($[M+Na]^+$) Calcd. for $C_{10}H_{18}N_2O_6SNa^+$: 317.0778 , Found: 317.0770.

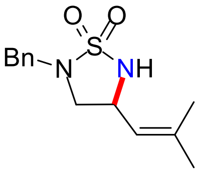

2l

2-Benzyl-4-(2-methylprop-1-en-1-yl)-1,2,5-thiadiazolidine 1,1-dioxide (**2l**) was obtained in 92% yield through General Procedure for C–H Amination of Sulfamoyl Azides. Purified by chromatography on silica gel (gradient elution: 4:1→2:1 Hexane/EtOAc), colorless liquid, TLC R_f = 0.50 (2/1 Hexane/EtOAc). ^{1}H NMR (400 MHz, $CDCl_3$): δ 7.36-7.29 (m, 5H), 5.14 (d, *J* = 8.8 Hz, 1H), 4.49-4.41 (m, 1H), 4.23, 4.08 (AB q, *J* = 13.6 Hz, each 1H), 4.22 (brs, 1H), 3.26 (dd, *J* = 7.2, 9.2 Hz, 1H), 2.98-2.92 (m, 1H), 1.69 (s, 3H), 1.64 (s, 3H). ^{13}C NMR (62.9 MHz, $CDCl_3$): δ 139.9, 135.2, 128.7, 128.6, 128.1, 121.4, 53.9, 50.6, 50.4, 25.6, 18.3. IR (neat, cm^{-1}): 1317,

1291, 1157, 1075, 1008, 891, 756, 698, 678. HRMS (ESI) ([M+Na]$^+$) Calcd. for $C_{13}H_{18}N_2O_2SNa^+$: 289.0981, Found: 289.0974.

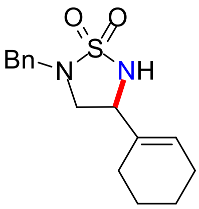

2m

2-Benzyl-4-(cyclohex-1-en-1-yl)-1,2,5-thiadiazolidine 1,1-dioxide (**2m**) was obtained in 98% yield through General Procedure for C–H Amination of Sulfamoyl Azides. Purified by chromatography on silica gel (gradient elution: 4:1→2:1 Hexane/EtOAc), white solid, TLC R_f = 0.50 (2:1 Hexane/EtOAc). ^{1}H NMR (250 MHz, $CDCl_3$): δ 7.36-7.30 (m, 5H), 5.74-5.71 (m, 1H), 4.30, 3.95 (AB q, J = 13.5 Hz, each 1H), 4.29-4.26 (m, 1H), 4.14 (q, J = 7.5 Hz, 1H), 3.27 (dd, J = 7.0, 9.5 Hz, 1H), 2.99 (dd, J = 8.3, 9.3 Hz, 1H), 2.04-1.80 (m, 4H), 1.63-1.50 (m, 4H). ^{13}C NMR (100 MHz, $CDCl_3$): δ 135.1, 133.9, 128.7, 128.6, 128.0, 126.1, 57.7, 51.7, 50.4, 24.9, 23.7, 22.2, 22.1. IR (neat, cm^{-1}): 1323, 1276, 1152, 909, 755, 698, 684. HRMS (ESI) ([M+H]$^+$) Calcd. for $C_{15}H_{21}N_2O_2S^+$: 293.1318, Found: 293.1321.

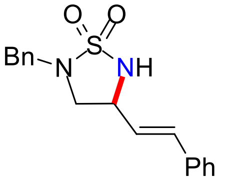

2n

(E)-2-Benzyl-4-styryl-1,2,5-thiadiazolidine 1,1-dioxide (**2n**) was obtained in 98% yield through General Procedure for C–H Amination of Sulfamoyl Azides. Purified by chromatography on silica gel (4/1 Hexane/EtOAc), white solid, TLC R_f = 0.3 (4/1 Hexane/EtOAc). ^{1}H NMR (500 MHz, $CDCl_3$): δ 7.41-7.27 (m, 10H), 6.63 (d, J = 16.0 Hz, 1H), 6.14 (dd, J = 8.0, 15.5 Hz, 1H), 4.69 (d, J = 6.0 Hz, 1H), 4.45-4.38 (m, 1H), 4.34, 4.08 (AB q, J = 14.0 Hz, each 1H), 3.45 (dd, J = 7.0, 9.5 Hz, 1H), 3.10 (dd, J = 8.0, 9.5 Hz, 1H). ^{13}C NMR (125 MHz, $CDCl_3$): δ 135.3, 134.9, 134.2, 128.8, 128.7, 128.7, 128.5, 128.2, 126.6, 125.4, 54.8, 53.2, 50.5. IR (neat, cm^{-1}): 3257, 1495, 1455, 1303, 1265, 1161, 967, 732, 693. HRMS (ESI) ([M+H]$^+$) Calcd. for $C_{17}H_{19}N_2O_2S^+$: 315.1162, Found: 315.1156.

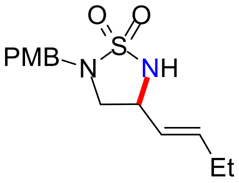

2o

(E)-4-(But-1-en-1-yl)-2-(4-methoxybenzyl)-1,2,5-thiadiazolidine 1,1-dioxide (**2o**) was obtained in 97% yield through General Procedure for C–H Amination of Sulfamoyl Azides. Purified by chromatography on silica gel (4/1 Hexane/EtOAc),

white solid, TLC R_f = 0.4 (4/1 Hexane/EtOAc). ^{1}H NMR (500 MHz, $CDCl_3$): δ 7.28 (d, J = 8.5 Hz, 2H), 6.88 (d, J = 8.5 Hz, 2H), 5.77 (dt, J = 15.0, 6.5 Hz, 1H), 5.40 (dd, J = 15.0, 7.5 Hz, 1H), 4.46 (d, J = 5.5 Hz, 1H), 4.21, 3.99 (AB q, J = 13.5 Hz, each 1H), 4.19-4.13 (m, 1H), 3.80 (s, 3H), 3.30 (dd, J = 7.5, 9.5 Hz, 1H), 2.96 (t, J = 7.5 Hz, 1H), 2.06-1.99 (m, 2H), 0.96 (t, J = 7.5 Hz, 3H). ^{13}C NMR (125 MHz, $CDCl_3$): δ 159.4, 137.9, 130.0, 126.9, 125.5, 114.1, 55.3, 54.7, 53.3, 49.8, 25.1, 13.0. IR (neat, cm^{-1}): 1613, 1513, 1302, 1248, 1163, 1031, 970, 734, 702. HRMS (ESI) ([M+Na]$^+$) Calcd. for $C_{14}H_{20}N_2O_3SNa^+$: 319.1087, Found: 319.1077.

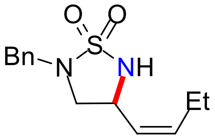

2p

(Z)-2-benzyl-4-(but-1-en-1-yl)-1,2,5-thiadiazolidine 1,1-dioxide (**2p**) was obtained in 80% yield through General Procedure for C–H Amination of Sulfamoyl Azides. Purified by chromatography on silica gel (gradient elution: 4/1→2/1 Hexane/EtOAc), colorless liquid, TLC R_f = 0.56 (2/1 Hexane/EtOAc). ^{1}H NMR (250 MHz, $CDCl_3$): δ 7.36-7.29 (m, 5H), 5.62 (dt, J = 10.8, 7.5 Hz, 1H), 5.36 (ddt, J = 10.5, 8.8, 1.5 Hz, 1H), 4.60-4.47 (m, 1H), 4.39 (d, J = 6.3 Hz, 1H), 4.25, 4.06 (AB q, J = 13.8 Hz, each 1H), 3.29 (dd, J = 6.8, 9.5 Hz, 1H), 2.96 (dd, J = 7.8, 9.3 Hz, 1H), 2.15-1.93 (m, 2H), 0.93 (t, J = 7.5 Hz, 3H). ^{13}C NMR (62.9 MHz, $CDCl_3$): 138.1, 135.1, 128.7, 128.6, 128.1, 125.4, 53.7, 50.4, 49.4, 21.0, 14.1. IR (neat, cm^{-1}): 1456, 1292, 1164, 891, 774, 728, 697. HRMS (ESI) ([M+NH_4]$^+$) Calcd. for $C_{13}H_{22}N_3O_2S^+$: 284.1427, Found: 284.1427.

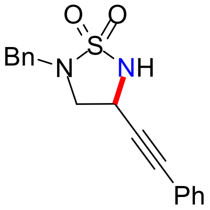

2q

2-Benzyl-4-(phenylethynyl)-1,2,5-thiadiazolidine 1,1-dioxide (**2q**) was obtained in 98% yield through General Procedure for C–H Amination of Sulfamoyl Azides. Purified by chromatography on silica gel (4/1 Hexane/EtOAc), white solid, TLC R_f = 0.35 (4/1 Hexane/EtOAc). ^{1}H NMR (500 MHz, $CDCl_3$): δ 7.42-7.29 (m, 10H), 4.76 (d, J = 6.5 Hz, 1H), 4.67 (q, J = 7.0 Hz, 1H), 4.32, 4.20 (AB q, J = 14.0 Hz, each 1H), 3.55 (dd, J = 7.0, 9.5 Hz, 1H), 3.41 (dd, J = 7.5, 9.5 Hz, 1H). ^{13}C NMR (125 MHz, $CDCl_3$): δ 134.7, 131.8, 129.1, 128.8, 128.6, 128.4, 128.3, 121.3, 86.3, 84.0, 53.8, 50.5, 44.4. IR (neat, cm^{-1}): 1491, 1334, 1303, 1166, 906, 727, 691. HRMS (ESI) ([M+H]$^+$) Calcd. for $C_{17}H_{17}N_2O_2S^+$: 313.1005, Found: 313.0997.

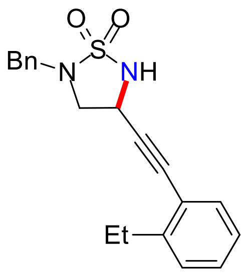

2r

2-benzyl-4-((2-ethylphenyl)ethynyl)-1,2,5-thiadiazolidine 1,1-dioxide (**2r**) was obtained in 98% yield through General Procedure for C–H Amination of Sulfamoyl Azides. Purified by chromatography on silica gel (4/1 Hexane/EtOAc), white solid, TLC R_f = 0.45 (4/1 Hexane/EtOAc). ^{1}H NMR (500 MHz, $CDCl_3$): δ 7.43-7.27 (m, 7H), 7.22 (d, J = 8.0 Hz, 1H), 7.15 (t, J = 7.5 Hz, 1H), 4.75-4.70 (m, 2H), 4.31, 4.24 (AB q, J = 14.0 Hz, each 1H), 3.59-3.55 (m, 1H), 3.45-3.41 (m, 1H), 2.75 (q, J = 7.5 Hz, 2H) 1.22 (t, J = 7.5 Hz, 3H). ^{13}C NMR (125 MHz, $CDCl_3$): δ 146.6, 134.8, 132.5, 129.4, 128.8, 128.6, 128.3, 128.0, 125.7, 120.3, 87.3, 85.0, 54.0, 50.5, 44.6, 27.5, 14.8. IR (neat, cm^{-1}): 1485, 1455, 1333, 1265, 1166, 759, 732, 698. HRMS (ESI) ([M+Na]$^+$) Calcd. for $C_{19}H_{20}N_2NaO_2S^+$: 363.1138, Found: 363.1153.

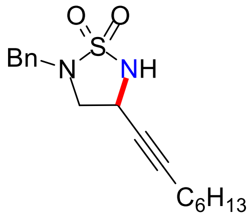

2s

2-Benzyl-4-(oct-1-yn-1-yl)-1,2,5-thiadiazolidine 1,1-dioxide (**2s**) was obtained in 90% yield through General Procedure for C–H Amination of Sulfamoyl Azides. Purified by chromatography on silica gel (4/1 Hexane/EtOAc), white solid, TLC R_f = 0.35 (4/1 Hexane/EtOAc). ^{1}H NMR (500 MHz, $CDCl_3$): δ 7.38-7.31 (m, 5H), 4.44-4.35 (m, 2H), 4.25, 4.17 (AB q, J = 14.0 Hz, each 1H), 3.41 (dd, J = 6.5, 9.5 Hz, 1H), 3.26 (dd, J = 7.0, 9.5 Hz, 1H), 2.18 (dt, J = 2.0, 7.0 Hz, 2H), 1.50-1.43 (m, 2H), 1.37-1.23 (m, 6H), 0.88 (t, J = 7.0 Hz, 3H). ^{13}C NMR (125 MHz, $CDCl_3$): δ 134.9, 128.8, 128.6, 128.2, 87.8, 75.2, 54.3, 50.4, 44.2, 31.2, 28.5, 28.2, 22.5, 18.6, 14.0. IR (neat, cm^{-1}): 1456, 1332, 1301, 1166, 907, 728. HRMS (ESI) ([M+H]$^+$) Calcd. for $C_{17}H_{25}N_2O_2S^+$: 321.1631, Found: 321.1631.

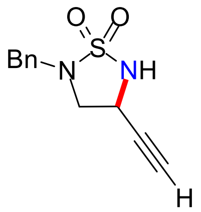

2t

2-Benzyl-4-ethynyl-1,2,5-thiadiazolidine 1,1-dioxide (**2t**) was obtained in 89% yield with 5 mol% [Co(**P1**)] through General Procedure for C–H Amination of Sulfamoyl Azides. Purified by chromatography on silica gel (gradient elution:

4/1→2/1 Hexane/EtOAc), white solid, TLC R_f = 0.28 (2:1 Hexane/EtOAc). ^{1}H NMR (500 MHz, $CDCl_3$): δ 7.38-7.32 (m, 5H), 4,58 (d, J = 4.0 Hz, 1H), 4.42 (dq, J = 2.0, 6.5 Hz, 1H), 4.27, 4.17 (AB q, J = 13.5 Hz, each 1H), 3.47 (dd, J = 7.0, 9.5 Hz, 1H), 3.33 (dd, J = 6.5, 9.5 Hz, 1H), 2.50 (d, J = 2.5 Hz, 1H). ^{13}C NMR (125 MHz, $CDCl_3$): δ 134.6, 128.8, 128.6, 128.3, 79.2, 74.7, 53.4, 50.6, 43.4. IR (neat, cm^{-1}): 1455, 1391, 1329, 1300, 1165, 907, 756, 728, 697. HRMS (ESI) ($[M+Na]^+$) Calcd. For $C_{11}H_{12}N_2NaO_2S^+$: 259.0512, Found: 259.0533.

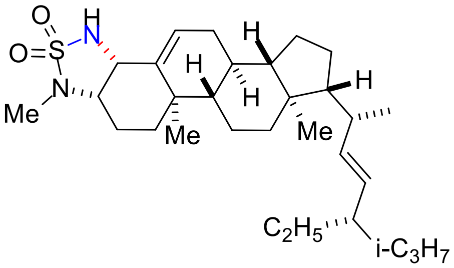

2u

An oven dried Schlenk tube was charged with catalyst (0.01 mmol) and 4Å MS (250 mg), then evacuated and back filled with nitrogen. The Teflon screw cap was replaced with a rubber septum and then an approximately 2.5 ml portion of benzene was added, then azide (265 mg, 0.5 mmol), followed by the remaining benzene (total 5 mL). The Schlenk tube was then purged with nitrogen for 2 minutes and the rubber septum was replaced with a Teflon screw cap. The Schlenk tube was then placed in an oil bath at 40˚ C. After 24 h, catalyst (0.01 mmol) was added to the reaction mixture under the N_2 atmosphere. After another 48 h, the reaction mixture was purified by flash column chromatography. The fractions containing product were collected and concentrated by rotary evaporation to afford the target compound **2u** (181 mg, 72%) . Purified by chromatography on silica gel (20/1 DCM/EtOAc), white solid, TLC R_f = 0.7 (20/1 DCM/EtOAc). ^{1}H NMR (500 MHz, $CDCl_3$): δ 5.73-5.71 (m, 1H), 5.14 (dd, J = 15.0, 8.5 Hz, 1H), 5.02 (dd, J = 15.0, 8.5 Hz, 1H), 4.30 (t, J = 5.5 Hz, 1H), 4.09 (d, J = 5.0 Hz, 1H), 3.37 (td, J = 5.5, 11.0 Hz, 1H), 2.77 (s, 3H), 2.13-1.99 (m, 4H), 1.90-1.87 (m, 2H), 1.76-1.67 (m, 1H), 1.63- 1.37 (m, 8H), 1.33-1.23 (m, 1H), 1.21-0.96 (m, 6H), 1.18 (s, 3H), 1.02 (d, J = 6.5 Hz, 3H), 0.94-0.88 (m, 1H), 0.84 (d, J = 6.0 Hz, 3H), 0.80 (t, J = 7.5 Hz, 6H), 0.70 (s, 3H). ^{13}C NMR (100 MHz, $CDCl_3$): δ 138.1, 137.3, 131.6, 129.4, 62.1, 59.8, 57.0, 55.9, 51.2, 49.5, 42.2, 40.4, 39.5, 36.4, 34.3, 32.1, 31.9, 31.5, 29.1, 28.9, 25.4, 24.2, 21.2, 21.1, 20.9, 20.6, 20.5, 19.0, 12.2, 12.1. IR (neat, cm^{-1}): 3232, 2955, 2868, 1454, 1381, 1304, 1265, 1148, 978, 914, 737. HRMS (ESI) ($[M+H]^+$) Calcd. for $C_{30}H_{51}N_2O_2S^+$: 503.3666, Found: 503.3673.

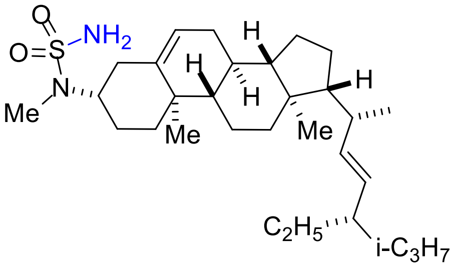

2u′

Yield: 22%. Purified by chromatography on silica gel (20/1 DCM/EtOAc), white solid, TLC R_f = 0.35 (16/1 DCM/EtOAc). ^{1}H NMR (500 MHz, CDCl$_3$): δ 5.37-5.35 (m, 1H), 5.15 (dd, J = 15.0, 8.5 Hz, 1H), 5.01 (dd, J = 15.0, 8.5 Hz, 1H), 4.36 (s, 2H), 3.74-3.66 (m, 1H), 2.82 (s, 3H), 2.46 (t, J = 13.0 Hz, 1H), 2.15-2.10 (m, 1H), 2.07-1.88 (m, 4H), 1.75-1.66 (m, 3H), 1.56-1.36 (m, 7H), 1.29-1.12 (m, 6H), 1.09-0.89 (m, 3H), 1.02 (d, J = 7.0 Hz, 3H), 0.99 (s, 3H), 0.85 (d, J = 6.5 Hz, 3H), 0.80 (t, J = 7.5 Hz, 6H), 0.69 (s, 3H). ^{13}C NMR (100 MHz, CDCl$_3$): δ 140.3, 138.3, 129.3, 122.2, 58.2, 56.8, 55.9, 51.2, 50.1, 42.2, 40.5, 39.6, 38.0, 36.6, 35.7, 31.9, 29.2, 28.9, 25.7, 25.4, 24.3, 21.2, 21.1, 21.0, 19.4, 19.0, 12.2, 12.0. IR (neat, cm^{-1}): 3259, 2958, 2933, 2869, 1463, 1344, 1318, 1300, 1265, 1153, 971, 952, 736. HRMS (ESI) ([M+Na]$^+$) Calcd. for $C_{30}H_{52}N_2NaO_2S^+$: 527.3642, Found: 527.3643.

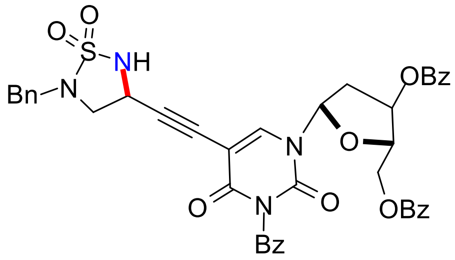

2v (dr ≈ 3/2)

Product **2v** was obtained through General Procedure for C–H Amination of Sulfamoyl Azides. Yield: 95%. Purified by chromatography on silica gel (4/1 Hexane/EtOAc), white solid, TLC R_f = 0.5 (1/1 Hexane/EtOAc). ^{1}H NMR (500 MHz, CDCl$_3$): δ 8.05-7.97 (m, 5H), 7.89 (d, J = 8.5 Hz, 2H), 7.66 (t, J = 7.5 Hz, 1H), 7.62-7.56 (m, 2H), 7.52-7.44 (m, 6H), 7.38-7.28 (m, 5H), 6.39-6.31 (m, 1H), 5.63 (d, J = 6.5 Hz, 1H), 4.77-4.74 (m, 2.4H), 4.69 (d, J = 6.5 Hz, 0.6H), 4.62 (d, J = 2.0 Hz, 1H), 4.48-4.39 (m, 1H), 4.26 (dd, J = 13.5, 2.0 Hz, 1H), 4.09 (d, J = 13.5 Hz, 1H), 3.37 (dd, J = 7.0, 10.0 Hz, 0.6H), 3.32 (dd, J = 7.0, 10.0 Hz, 0.4H), 3.25-3.17 (m, 1H), 2.84 (dd, J = 14.5, 5.5 Hz, 1H), 2.43-2.35 (m, 1H). ^{13}C NMR (100 MHz, CDCl$_3$): δ 167.3, 166.0, 165.9, 159.9, 148.0, 142.4, 135.4, 134.7, 133.8, 130.9, 130.5, 129.7, 129.6, 129.5, 129.3, 129.1, 129.0, 128.9, 128.8, 128.6, 128.4, 128.3, 99.1, 99.1, 89.2, 86.6, 83.5, 74.8, 64.3, 53.4, 50.7, 44.2, 38.8. IR (neat, cm^{-1}): 1754, 1712, 1671, 1450, 1315, 1266, 1167, 1095, 1070, 907, 727, 712. HRMS (ESI) ([M+Na]$^+$) Calcd. For $C_{41}H_{34}N_4NaO_{10}S^+$: 797.1888, Found: 797.1842.

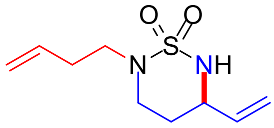

2w

2-(But-3-en-1-yl)-5-vinyl-1,2,6-thiadiazinane 1,1-dioxide (**2w**) was obtained in 44% yield through General Procedure for C–H Amination of Sulfamoyl Azides. Purified by chromatography on silica gel (gradient elution: 4/1→1/1 Hexanes/EtOAc), white solid, TLC R_f = 0.44 (1/1 Hexanes/EtOAc). ^{1}H NMR (400 MHz, CDCl$_3$): δ 5.83-5.70 (m, 2H), 5.27-5.01 (m, 4H), 4.22-4.14 (m, 1H), 3.96 (d, J = 9.2 Hz, 1H), 3.48 (dt, J = 3.2, 12.8 Hz, 1H), 3.29-3.17 (m, 2H), 3.07-3.00 (m, 1H), 2.35-2.28 (m, 2H), 1.77-1.60

(m, 2H). ^{13}C NMR (100 MHz, $CDCl_3$): δ 136.0, 134.8, 116.9, 116.5, 57.5, 48.6, 48.2, 32.3, 28.6. IR (neat, cm^{-1}): 1324, 1302, 1145, 1076, 918, 861, 771. HRMS (ESI) ($[M+H]^+$) Calcd. for $C_9H_{17}N_2O_2S^+$: 217.1005, Found: 217.1002.

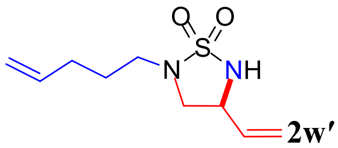

2w′

2-(Pent-4-en-1-yl)-4-vinyl-1,2,5-thiadiazolidine 1,1-dioxide (**2w′**) was obtained in 35% yield through General Procedure for C–H Amination of Sulfamoyl Azides. Purified by chromatography on silica gel (gradient elution: 4/1→1/1 Hexanes/EtOAc), colorless liquid, TLC R_f = 0.41 (1/1 Hexanes/EtOAc). ^{1}H NMR (400 MHz, $CDCl_3$): δ 5.88-5.71 (m, 2H), 5.36 (d, J = 17.2 Hz, 1H), 5.25 (d, J = 10.4 Hz, 1H), 5.03 (dd, J = 1.6, 17.2 Hz, 1H), 4.98 (d, J = 10.0 Hz, 1H), 4.52 (d, J = 5.6 Hz, 1H), 4.23 (q, J = 6.8 Hz, 1H), 3.51 (dd, J = 6.8, 9.2 Hz, 1H), 3.10-3.02 (m, 2H), 2.96-2.88 (m, 1H), 2.15-2.09 (m, 2H), 1.74-1.65 (m, 2H). ^{13}C NMR (100 MHz, $CDCl_3$): δ 137.2, 134.8, 119.1, 115.6, 54.8, 53.8, 46.3, 30.8, 26.9. IR (neat, cm^{-1}): 1294, 1155, 1066, 992, 916, 740. HRMS (ESI) ($[M+H]^+$) Calcd. for $C_9H_{17}N_2O_2S^+$: 217.1005, Found: 217.1000.

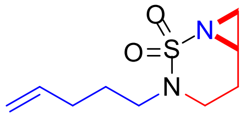

2w″

3-(Pent-4-en-1-yl)-2-thia-1,3-diazabicyclo[4.1.0]heptane 2,2-dioxide (**2w″**) was obtained in 20% yield through General Procedure for C–H Amination of Sulfamoyl Azides. Purified by chromatography on silica gel (gradient elution: 4/1→1/1 Hexanes/EtOAc), liquid, TLC R_f = 0.16 (1/1 Hexanes/EtOAc). ^{1}H NMR (400 MHz, $CDCl_3$): δ 5.78-5.66 (m, 1H), 5.00-4.91 (m, 2H), 3.35-3.28 (m, 1H), 3.24-3.03 (m, 3H), 2.96-2.91 (m, 1H), 2.53 (d, J = 4.0 Hz, 1H), 2.42 (d, J = 4.8 Hz, 1H), 2.40-2.32 (m, 1H), 2.07-1.99 (m, 3H), 1.64-1.56 (m, 2H). ^{13}C NMR (100 MHz, $CDCl_3$): δ 137.3, 115.4, 49.0, 43.8, 40.2, 32.9, 30.6, 27.1, 17.3. IR (neat, cm^{-1}): 1330, 1147, 1066, 1050, 913, 711. HRMS (ESI) ($[M+H]^+$) Calcd. for $C_9H_{17}N_2O_2S^+$: 217.1005, Found: 217.1000.

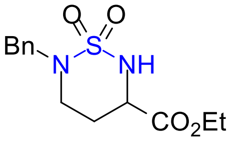

3

Ethyl 6-benzyl-1,2,6-thiadiazinane-3-carboxylate 1,1-dioxide **3** was synthesized according to the reference.[2b] Purified by chromatography on silica gel (gradient elution: 4:1→2:1 Hexanes/EtOAc), white solid, TLC R_f = 0.17 (4:1 Hexanes/EtOAc). ^{1}H NMR (250 MHz, $CDCl_3$): δ 7.34-7.25 (m, 5H), 4.71 (d, J = 8.8 Hz, 1H), 4.44, 4.00 (AB q, J = 13.8 Hz, each 1H), 4.39-4.32 (m, 1H), 4.23 (q, J = 7.0 Hz, 2H), 3.40 (dt, J

= 3.3, 13.3 Hz, 1H), 3.19-3.09 (m, 1H), 1.94-1.67 (m, 2H), 1.27 (t, J = 7.0 Hz, 3H). ^{13}C NMR (62.9 MHz, $CDCl_3$): δ 169.5, 135.1, 128.7, 128.1, 62.2, 57.4, 51.6, 47.0, 25.9, 14.1. IR (neat, cm^{-1}): 1742, 1331, 1306, 1206, 1165, 1144, 1114, 1036, 861, 776, 747, 694. HRMS (ESI) ($[M+Na]^+$) Calcd. for $C_{13}H_{18}N_2O_4SNa^+$: 321.0880, Found: 321.0875.

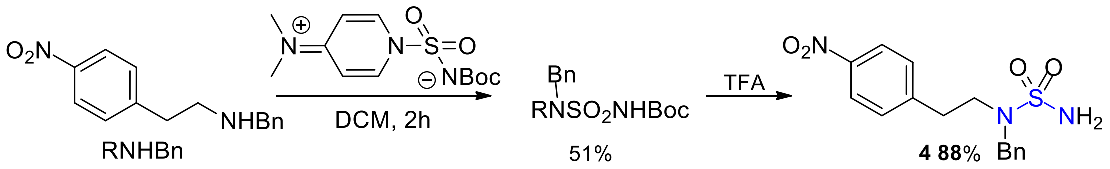

To a solution of N-benzyl-2-(4-nitrophenyl)ethan-1-amine (40 mg, 0.156 mmol) in DCM (0.5 mL) was added N-(*tert*-butoxycarbonyl)-N-[4-(dimethylazaniumylidene) -1,4-dihydropyridin-1-ylsulfonyl]-azanide[16] (55 mg, 0.187 mmol). The reaction mixture was stirred for 30 minutes followed by a short plug of silica gel to give the desired *tert*-butyl (N-benzyl-N-(4-nitrophenethyl) sulfamoyl) carbamate as white solid (35 mg, 51% yield). ^{1}H NMR (500 MHz, $CDCl_3$): δ 8.09 (d, J = 8.5 Hz, 2H), 7.38-7.32 (m, 5H), 7.17 (d, J = 8.5 Hz, 2H), 7.10 (brs, 1H), 4.48 (s, 2H), 3.59 (t, J = 7.5 Hz, 2H), 2.84 (t, J = 7.5 Hz, 2H), 1.50 (s, 9H).

tert-Butyl (N-benzyl-N-(4-nitrophenethyl) sulfamoyl) carbamate (33 mg, 0.083 mmol) was dissolved into DCM (0.5 mL). Then TFA (1 mL) was added to this solution and the reaction mixture was stirred for overnight. The solvent was removed. The residue was purified by chromatography on silica gel (3/1 Hexane/EtOAc) to form desired N-benzyl-N-(4-nitrophenethyl) sulfamoyl amide (**4**), white solid (22 mg, 88% yield), TLC R_f = 0.5 (1/1 Hexane/EtOAc). ^{1}H NMR (500 MHz, $CDCl_3$): δ 8.09 (d, J = 8.5 Hz, 2H), 7.38-7.30 (m, 5H), 7.21 (d, J = 8.5 Hz, 2H), 4.40 (brs, 2H), 4.36 (s, 2H), 3.40 (t, J = 7.5 Hz, 2H), 2.92 (t, J = 7.5 Hz, 2H). ^{13}C NMR (125 MHz, $CDCl_3$): δ 146.7, 146.2, 135.5, 129.7, 128.9, 128.5, 128.3, 123.7, 52.8, 49.1, 34.7. IR (neat, cm^{-1}): 3340, 3269, 1733, 1518, 1348, 1340, 1266, 1244, 1147, 898, 734, 698. HRMS (ESI) ($[M+Na]^+$) Calcd. for $C_{15}H_{17}N_3NaO_4S^+$: 358.0832, Found: 358.0849.

[16] Toupet, L.; Barragan, V.; Dewynter, G.; Montero, J.-L. *Org. Lett.* **2001**, *3*, 2241.

Scheme S2

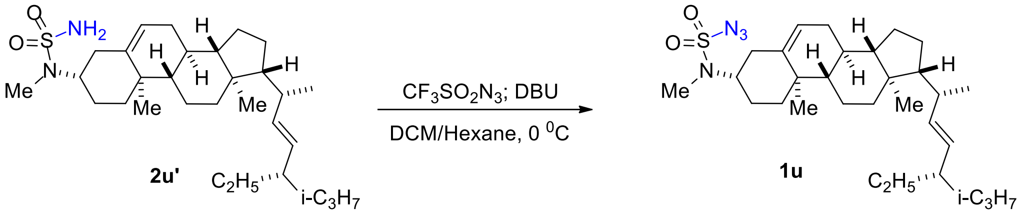

To a solution of $CF_3SO_2N_3$ (0.1 M solution in Hexanes) (2.2 mL, 0.22 mmol) at 0˚C, a mixture of stigmasterol-based sulfamoyl amine (55 mg, 0.11 mmol) and DBU (33 µL, 0.22 mmol) in DCM (3 mL) was added dropwisely via syringe. The reaction mixture was stirred at 0˚C for 15 min until the TLC shows the full conversion of starting material. Then the majority of the solvent was removed under reduced pressure at room temperature. Purification of this mixture by chromatography on silica gel (16/1 Hexane/EtOAc) led to desired product as the white solid (51 mg, 87%). TLC R_f = 0.75 (8/1 Hexane/EtOAc). The ^{1}H NMR was identical to the compound **1u** obtained through General Method for the Synthesis of Sulfamoyl Azides.

Scheme S3

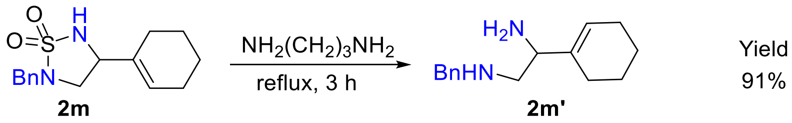

An oven dried Schlenk tube was charged with 0.2 mmol of 2-benzyl-4-(cyclohex-1-en-1-yl)-1,2,5-thiadiazolidine 1,1-dioxide (**2m**) in 0.3 mL of dry 1,3-diaminopropane. The Teflon screw cap was replaced with a rubber septum. The Schlenk tube was then purged with nitrogen for 2 minutes and the rubber septum was replaced with a Teflon screw cap. The mixture was refluxed for 3 h. After cooling to room temperature, the mixture was diluted with 5 mL of CH_2Cl_2, and extracted with 5 mL of water. After extraction of the water layer with 4 x 5 mL of CH_2Cl_2, the combined organic layers were dried over Na_2SO_4. The product was obtained as a colorless oil in high yield after removal of the solvent under reduced pressure. ^{1}H NMR (400 MHz, $CDCl_3$): δ 7.26-7.18 (m, 5H), 5.55 (brs, 1H), 3.73 (s, 2H), 3.24 (t, J = 6.0 Hz, 1H), 2.61 (dd, J = 4.4, 7.6 Hz, 1H), 2.46 (dd, J = 7.6, 11.6 Hz, 1H), 1.94 (brs, 3H), 1.78 (brs, 1H), 1.55-1.48 (m, 7H). ^{13}C NMR (62.9 MHz, $CDCl_3$): 140.5, 139.7, 128.3, 128.0, 126.8, 122.3, 57.2, 53.9, 53.6, 25.0, 24.6, 22.8, 22.7. IR (neat, cm^{-1}): 2987, 1452, 1406, 1066, 1056, 735, 698. HRMS (ESI) ([M+H]$^+$) Calcd. for $C_{15}H_{23}N_2^+$: 231.1856, Found: 231.1854.

X-ray Crystallograhy

The X-ray diffraction data for compounds **2c** and **4** were measured on a Bruker D8 Venture PHOTON 100 CMOS system equipped with a Cu K_{α} INCOATEC Imus micro-focus source (λ = 1.54178 Å). The X-ray diffraction data for compounds **2i** and **3** were collected using Bruker-AXS SMART-APEXII CCD diffractometer using Kα radiation (λ = 1.54178 Å). Indexing was performed using *APEX2* [1] (Difference Vectors method). Data integration and reduction were performed using SaintPlus 6.01 [2]. Absorption correction was performed by multi-scan method implemented in SADABS [3]. Space groups were determined using XPREP implemented in APEX2 [1]. Structures were solved using SHELXS-97 (direct methods) and refined using SHELXL-2013 [7] (full-matrix least-squares on F^2) contained in APEX2 [1,7], WinGX v1.70.01 [4,5,6,7] and OLEX2 [7,8]. All non-hydrogen atoms were refined anisotropically.

Compound **2c:** Hydrogen atoms of –NH groups have been found from difference Fourier map and were freely refined. The remaining hydrogen atoms were placed in geometrically calculated positions and included in the refinement process using riding model with isotropic thermal parameters: Uiso(H) = 1.2Ueq(-CH). There are two molecules of opposite chirality in an asymmetric unit. Crystal data and refinement conditions are shown in Table 1.

Compound **2i:** All hydrogen atoms were found in the difference Fourier map and were freely refined. Crystal data and refinement conditions are shown in Table 2.

Compound **3:** hydrogen atom of -NH group (H2A) has been found from difference Fourier map and was freely refined. The remaining hydrogen atoms were placed in geometrically calculated positions and included in the refinement process using riding model with isotropic thermal parameters: Uiso(H) = 1.2Ueq(-CH,-CH_2) and Uiso(H) = 1.5Ueq(-CH_3). Crystal data and refinement conditions are shown in Table 3.

Compound **4:** Hydrogen atom of –NH_2 group has been found from difference Fourier map and was freely refined. The remaining hydrogen atoms were placed in geometrically calculated positions and included in the refinement process using riding model with isotropic thermal parameters: Uiso(H) = 1.2Ueq(-CH). Crystal data and refinement conditions are shown in Table 4.

[1] Bruker (2013). *APEX2* (Version 2013.6-2). Bruker AXS Inc., Madison, Wisconsin, USA.

[2] Bruker (2013). SAINT-V8.32A. Data Reduction Software.

[3] Sheldrick, G. M. (1996). *SADABS. Program for Empirical Absorption Correction.* University of Gottingen, Germany.

[4] Farrugia L.*J. Appl. Cryst.* (1999). 32, 837±838

[5] Sheldrick, G.M. (1997) SHELXL-97. Program for the Refinement of Crystal

[6] Sheldrick, G.M. (1990) *Acta Cryst.* A46, 467-473

[7] Sheldrick, G. M. (2008). *Acta Cryst.* A64, 112-122.

[8] Dolomanov, O.V.; Bourhis, L.J.; Gildea, R.J.; Howard, J.A.K.; Puschmann, H., OLEX2: A complete structure solution, refinement and analysis program (2009). *J. Appl. Cryst.*, 42, 339-341.

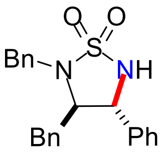

2c

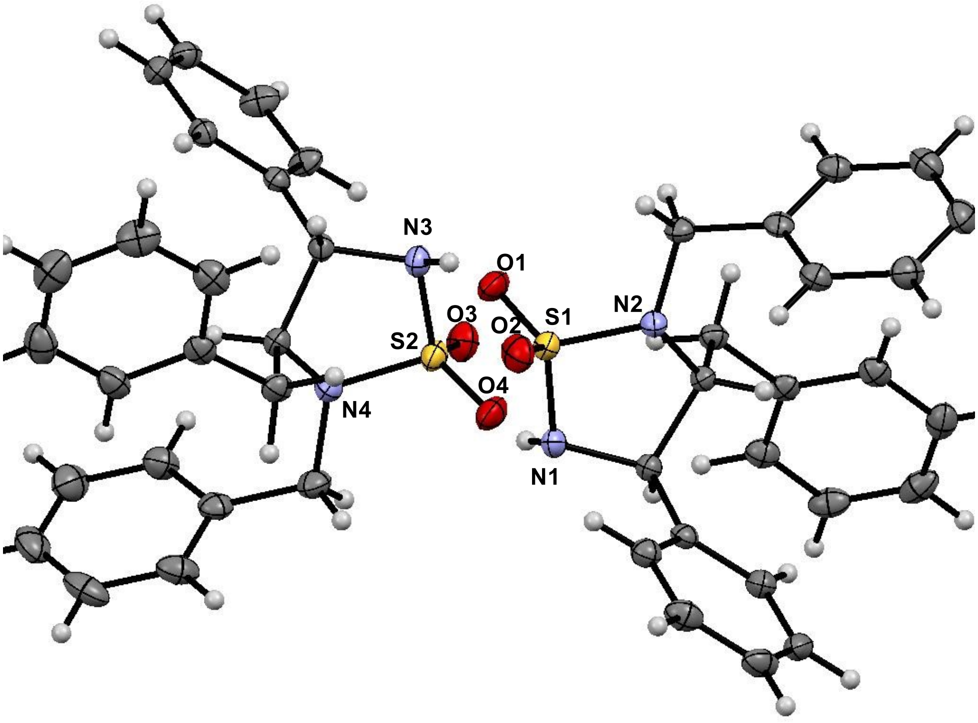

**Table 1. Crystal data and structure refinement for compound 2c**	
Identification code	2c
Empirical formula	$C_{22}H_{22}N_2O_2S$
Formula weight	378.47
Temperature/K	100.0
Crystal system	monoclinic
Space group	$P2_1/c$
a/Å	10.1061(3)
b/Å	18.1581(5)
c/Å	20.5423(6)
α/°	90
β/°	90.2280(10)
γ/°	90
Volume/Å^3	3769.64(19)
Z	8
ρ_{calc}g/cm^3	1.334
μ/mm^{-1}	1.680

F(000)	1600.0
Crystal size/mm^3	0.12 × 0.03 × 0.02
Radiation	CuKα (λ = 1.54178)
2Θ range for data collection/°	6.496 to 138.82
Index ranges	$-12 \leq h \leq 12, -21 \leq k \leq 22, -24 \leq l \leq 24$
Reflections collected	49569
Independent reflections	6932 [R_{int} = 0.0432, R_{sigma} = 0.0223]
Data/restraints/parameters	6932/0/495
Goodness-of-fit on F^2	1.058
Final R indexes [I>=2σ (I)]	R_1 = 0.0345, wR_2 = 0.0821
Final R indexes [all data]	R_1 = 0.0424, wR_2 = 0.0880
Largest diff. peak/hole / e Å^{-3}	0.40/-0.28

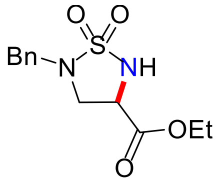

2i

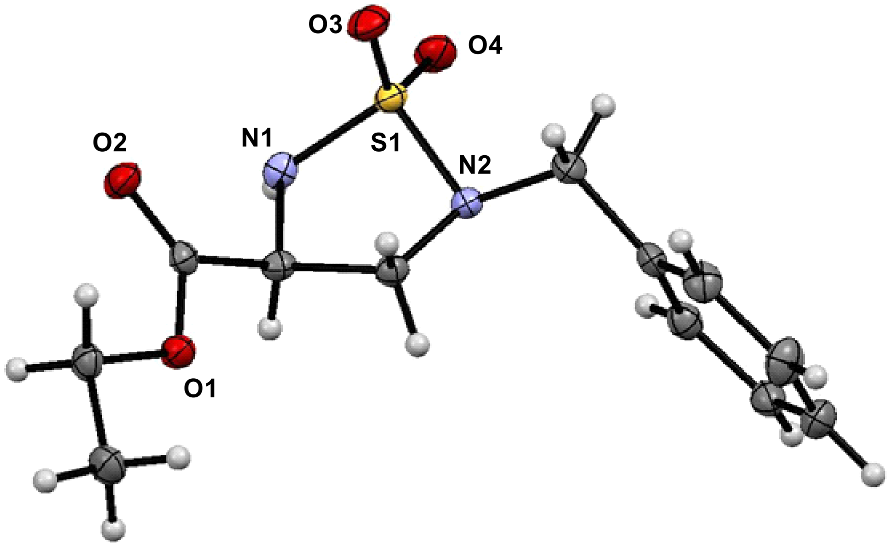

**Table 2. Crystal data and structure refinement for compound 2i**	
Identification code	**2i**
Empirical formula	$C_{12} H_{16} N_2 O_4 S$
Formula weight	284.33
Temperature	100(2) K
Wavelength	1.54178 A
Crystal system, space group	Orthorhombic, P212121

Unit cell dimensions	a = 5.7221(1) A alpha = 90 deg.
	b = 9.3754(2) A beta = 90 deg.
	c = 24.6691(5) A gamma = 90 deg.
Volume	1323.42(5) A^3
Z, Calculated density	4, 1.427 Mg/m^3
Absorption coefficient	2.304 mm^-1
F(000)	600
Crystal size	0.30 x 0.10 x 0.05 mm
Theta range for data collection	3.58 to 67.34 deg.
Limiting indices	-6<=h<=6, -11<=k<=11, -29<=l<=28
Reflections collected / unique	11376 / 2324 [R(int) = 0.0405]
Completeness to theta = 67.34	98.2 %
Absorption correction	Semi-empirical from equivalents
Max. and min. transmission	0.8935 and 0.5449
Refinement method	Full-matrix least-squares on F^2
Data / restraints / parameters	2324 / 0 / 236
Goodness-of-fit on F^2	1.071
Final R indices [I>2sigma(I)]	R1 = 0.0260, wR2 = 0.0653
R indices (all data)	R1 = 0.0272, wR2 = 0.0659
Absolute structure parameter	0.003(15)
Largest diff. peak and hole	0.194 and -0.359 e.A^-3

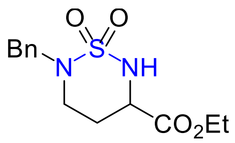

3

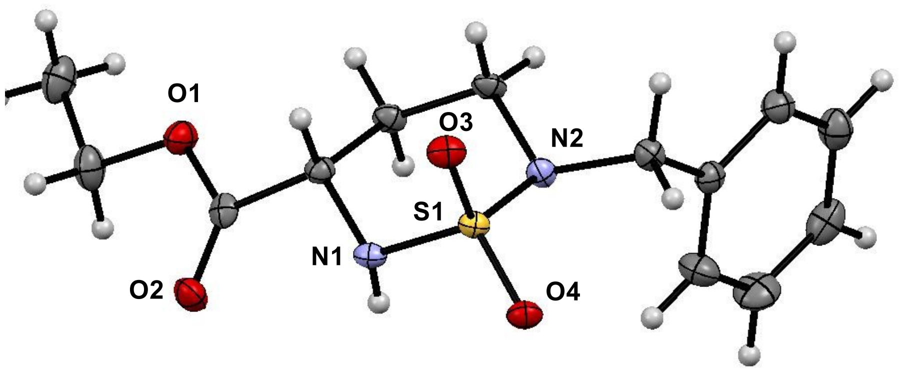

**Table 3. Crystal data and structure refinement for compound 3**	
Identification code	**3**
Empirical formula	$C_{13}H_{18}N_2O_4S$
Formula weight	298.35
Temperature/K	100.15
Crystal system	orthorhombic
Space group	$P2_12_12_1$
a/Å	6.1655(2)
b/Å	9.5773(3)
c/Å	24.0447(8)
α/°	90
β/°	90
γ/°	90
Volume/Å^3	1419.81(8)
Z	4
ρ_{calc}mg/mm^3	1.396
m/mm^{-1}	2.173
F(000)	632.0
Crystal size/mm^3	0.2 × 0.2 × 0.03
Radiation	CuKα (λ = 1.54178)
2Θ range for data collection	7.352 to 137.74°
Index ranges	$-6 \leq h \leq 5$, $-11 \leq k \leq 11$, $-28 \leq l \leq 27$
Reflections collected	9770
Independent reflections	2515 [R_{int} = 0.0478, R_{sigma} = 0.0420]
Data/restraints/parameters	2515/0/186
Goodness-of-fit on F^2	1.031
Final R indexes [I>=2σ (I)]	R_1 = 0.0304, wR_2 = 0.0712
Final R indexes [all data]	R_1 = 0.0332, wR_2 = 0.0723
Largest diff. peak/hole / e Å^{-3}	0.19/-0.34
Flack parameter	0.032(12)

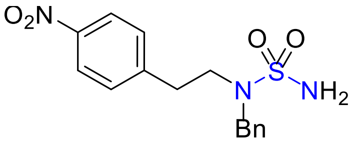

4

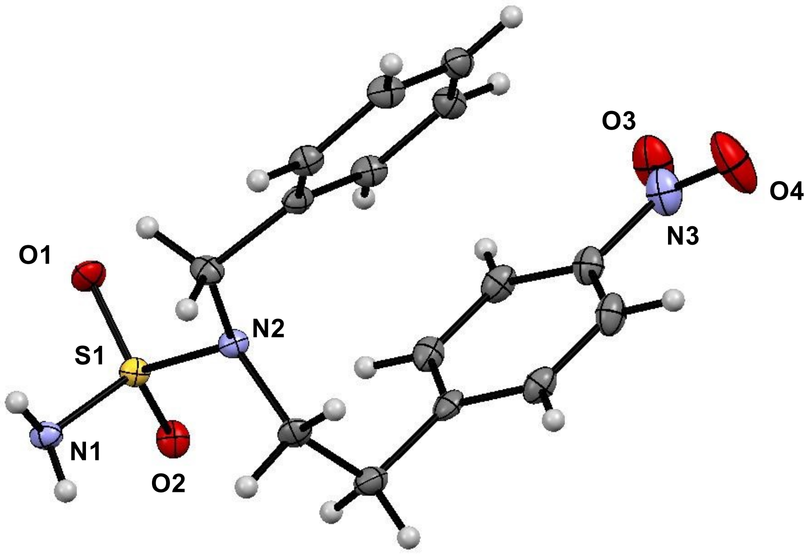

**Table 4. Crystal data and structure refinement for compound 4**	
Identification code	**4**
Empirical formula	$C_{15}H_{17}N_3O_4S$
Formula weight	335.38
Temperature/K	100.0
Crystal system	orthorhombic
Space group	$P2_12_12_1$
a/Å	6.0617(2)
b/Å	7.1812(2)
c/Å	35.5229(10)
α/°	90
β/°	90
γ/°	90
Volume/Å^3	1546.32(8)
Z	4
ρ_{calc}g/cm^3	1.441
μ/mm^{-1}	2.086
F(000)	704.0

Crystal size/mm^3	0.25 × 0.02 × 0.02
Radiation	CuKα (λ = 1.54178)
2Θ range for data collection/°	9.96 to 138.65
Index ranges	-7 ≤ h ≤ 7, -8 ≤ k ≤ 8, -42 ≤ l ≤ 42
Reflections collected	19967
Independent reflections	2863 [R_{int} = 0.0655, R_{sigma} = 0.0368]
Data/restraints/parameters	2863/0/216
Goodness-of-fit on F^2	1.026
Final R indexes [I>=2σ (I)]	R_1 = 0.0323, wR_2 = 0.0833
Final R indexes [all data]	R_1 = 0.0349, wR_2 = 0.0849
Largest diff. peak/hole / e Å^{-3}	0.30/-0.31
Flack parameter	0.086(9)

DSC Spectrum for a Sulfamoyl Azide

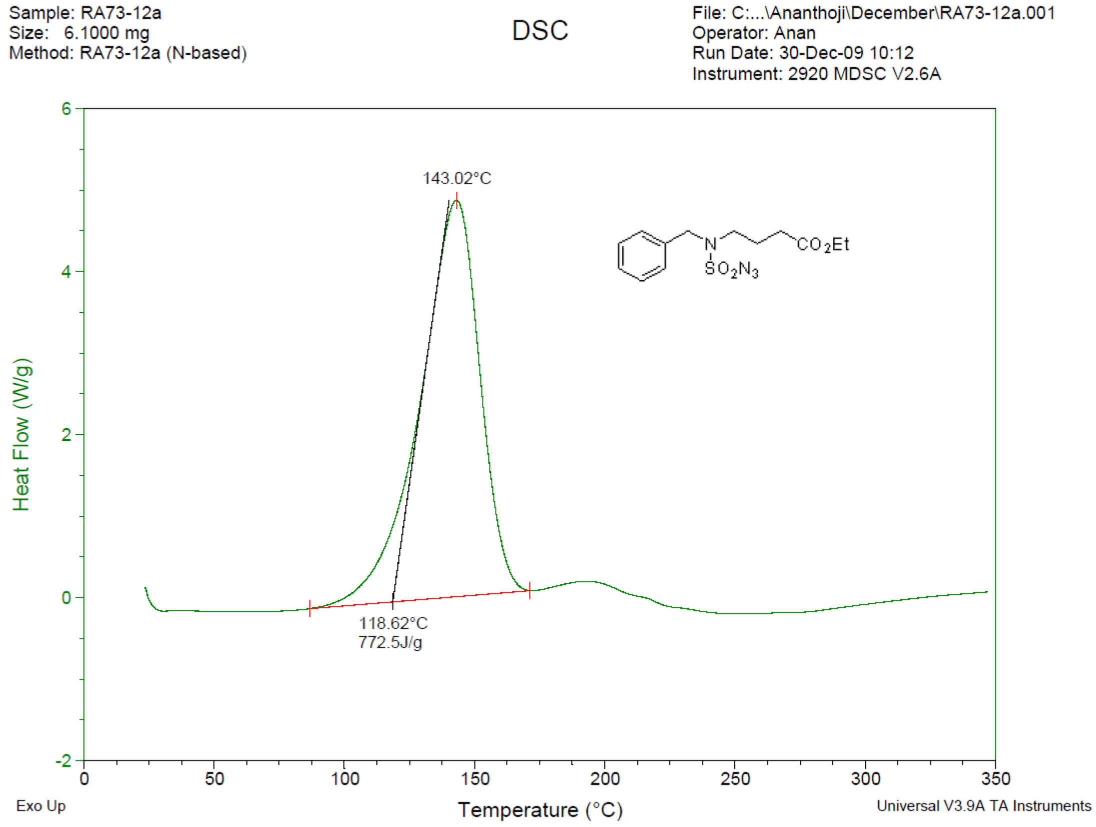

Supplement: Supplementary file 1 [file SC-007-C6SC02231F-s001.pdf]
