# Supplementary material for: Intramolecular 1,5-C(sp3)–H radical amination via Co(ii)-based metalloradical catalysis for five-membered cyclic sulfamides
Source: Chem Sci. 2016 Jul 28;7(12):6934–9. doi: 10.1039/c6sc02231f (PMC5271564; doi:10.1039/c6sc02231f)
Supplement: Supplementary file 2 [file SC-007-C6SC02231F-s002.pdf]

Electronic Supplementary Material (ESI) for Chemical Science.
This journal is © The Royal Society of Chemistry 2016

Supporting Information

Intramolecular 1,5-C(sp^3)–H Radical Amination via Co(II)-Based Metalloradical Catalysis for Five-Membered Cyclic Sulfamides

Hongjian Lu,[*#†‡] Kai Lang,[#§†] Huiling Jiang,[†] Lukasz Wojtas[†] and X. Peter Zhang[*§†]

[§]Department of Chemistry, Merkert Chemistry Center, Boston College, Chestnut Hill, Massachusetts 02467, United States

[†]Department of Chemistry, University of South Florida, Tampa, Florida 33620, United States

[‡]The Institute of Chemistry & Biomedical Sciences, Nanjing University, Nanjing, 210093, P. R. China

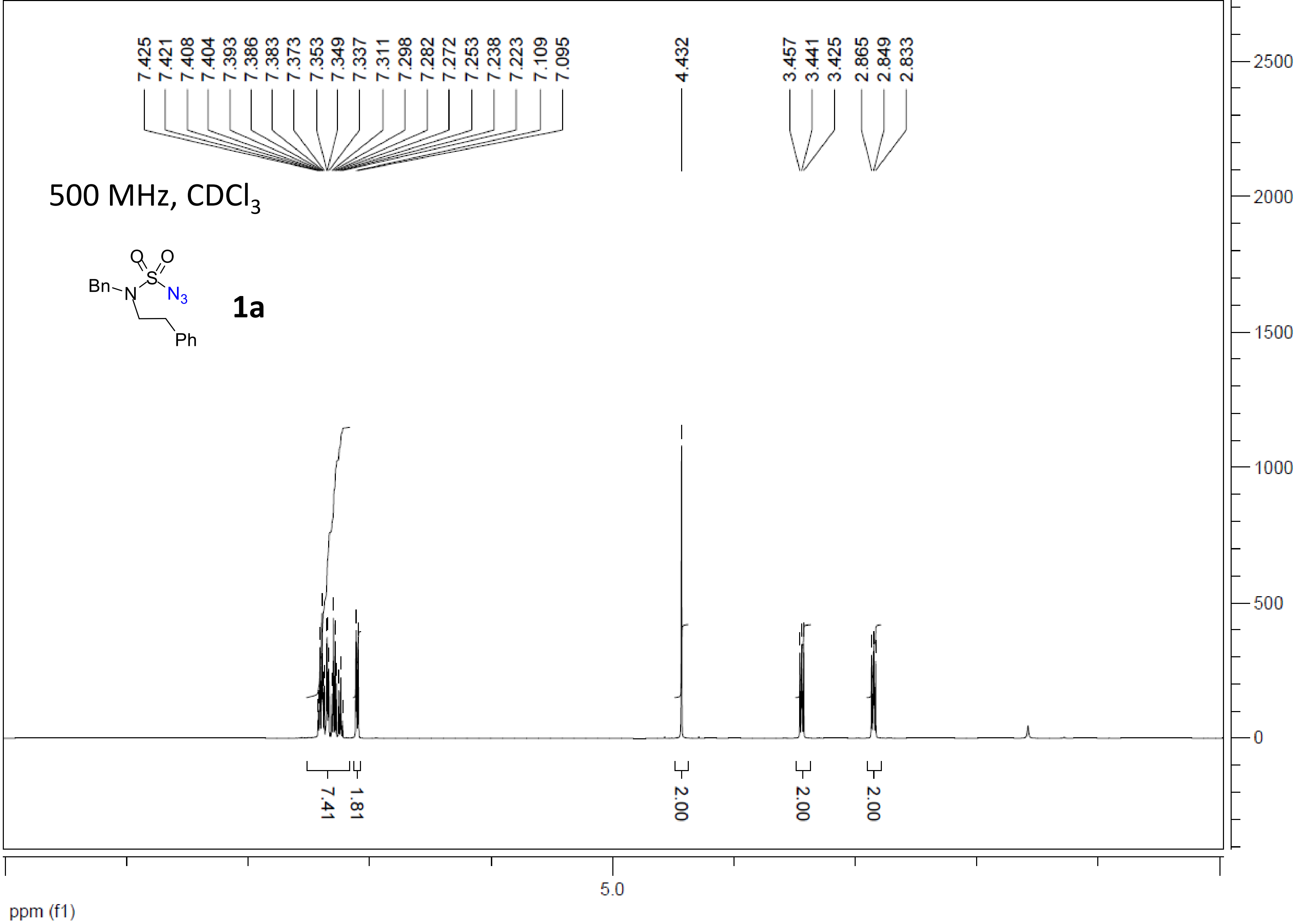
7.425 7.421 7.408 7.404 7.393 7.386 7.383 7.373 7.353 7.349 7.337 7.311 7.298 7.282 7.272 7.253 7.238 7.223 7.109 7.095
4.432
3.457 3.441 3.425 2.865 2.849 2.833
500 MHz, CDCl3
Bn
N
S
O
O
N3
Ph
1a
2500
2000
1500
1000
500
0
7.41
1.81
2.00
2.00
2.00
5.0
ppm (f1)

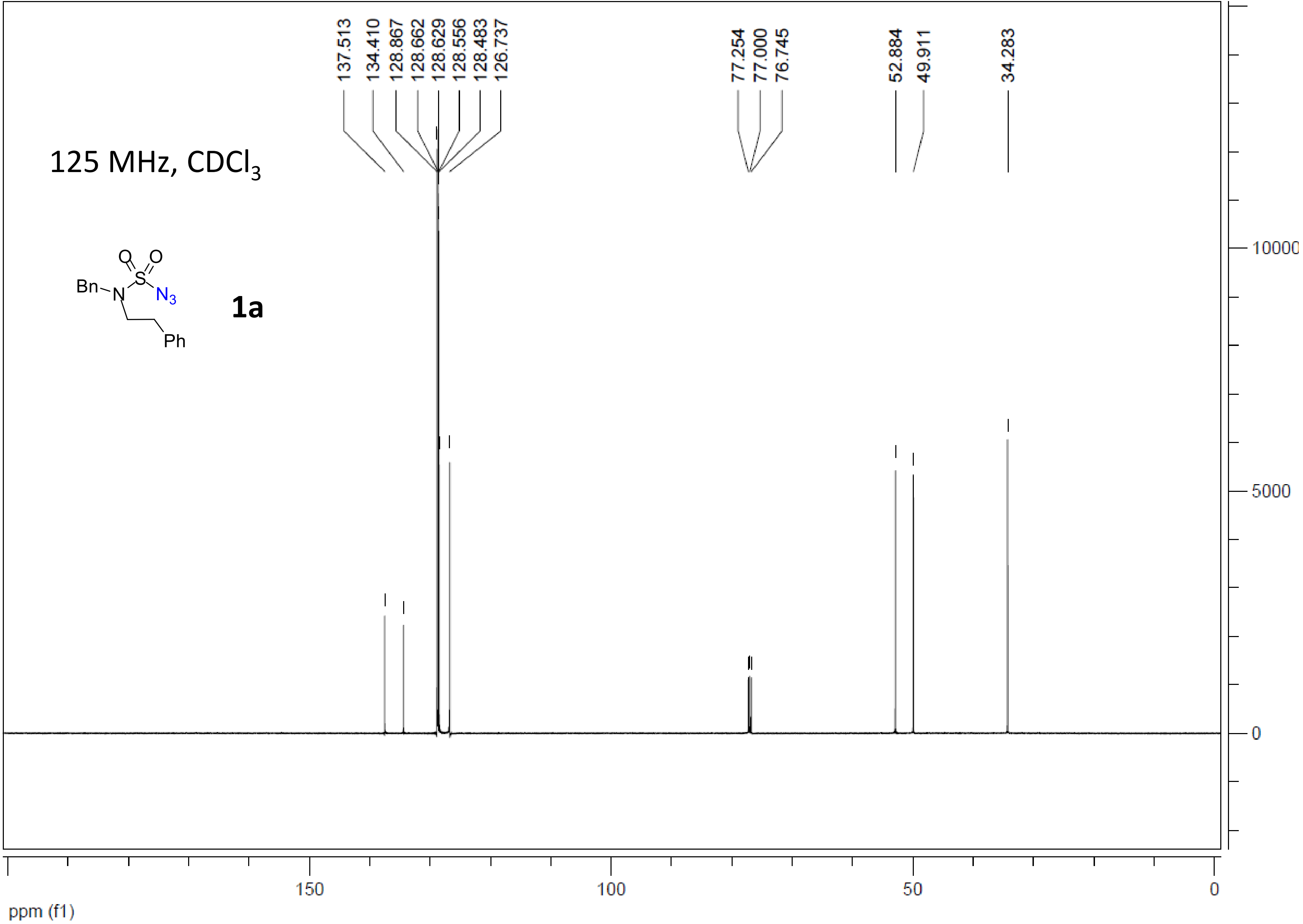
137.513
134.410
128.867
128.662
128.629
128.556
128.483
126.737
77.254
77.000
76.745
52.884
49.911
34.283
125 MHz, CDCl3
O O
S
Bn N N3
Ph
1a
10000
5000
0
150
100
50
0
ppm (f1)

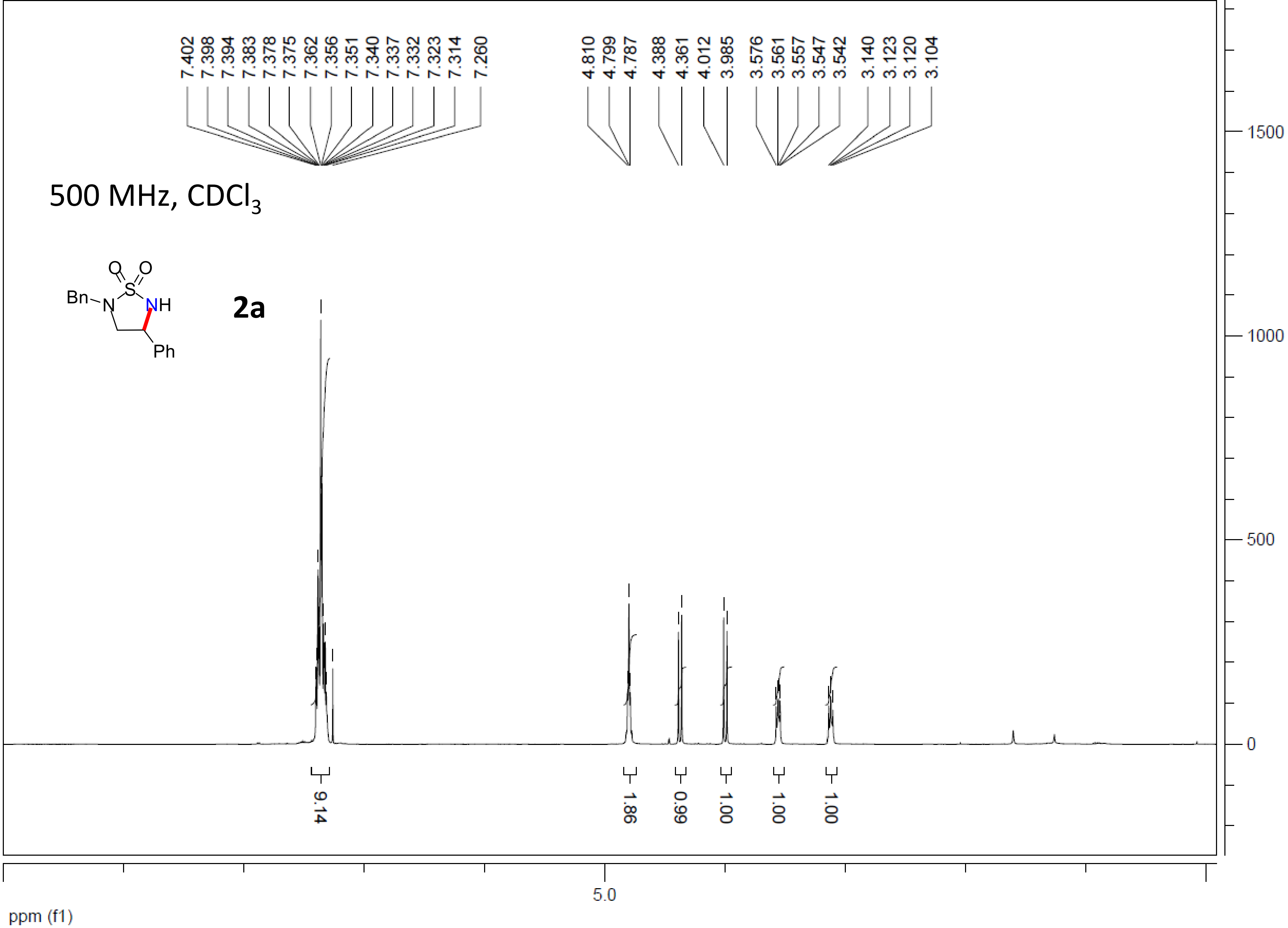
7.402 7.398 7.394 7.383 7.378 7.375 7.362 7.356 7.351 7.340 7.337 7.332 7.323 7.314 7.260
4.810 4.799 4.787 4.388 4.361 4.012 3.985 3.576 3.561 3.557 3.547 3.542 3.140 3.123 3.120 3.104
500 MHz, $CDCl_3$
Bn
N
S
O
O
NH
Ph
2a
9.14
1.86
0.99
1.00
1.00
1.00
1500
1000
500
0
5.0
ppm (f1)

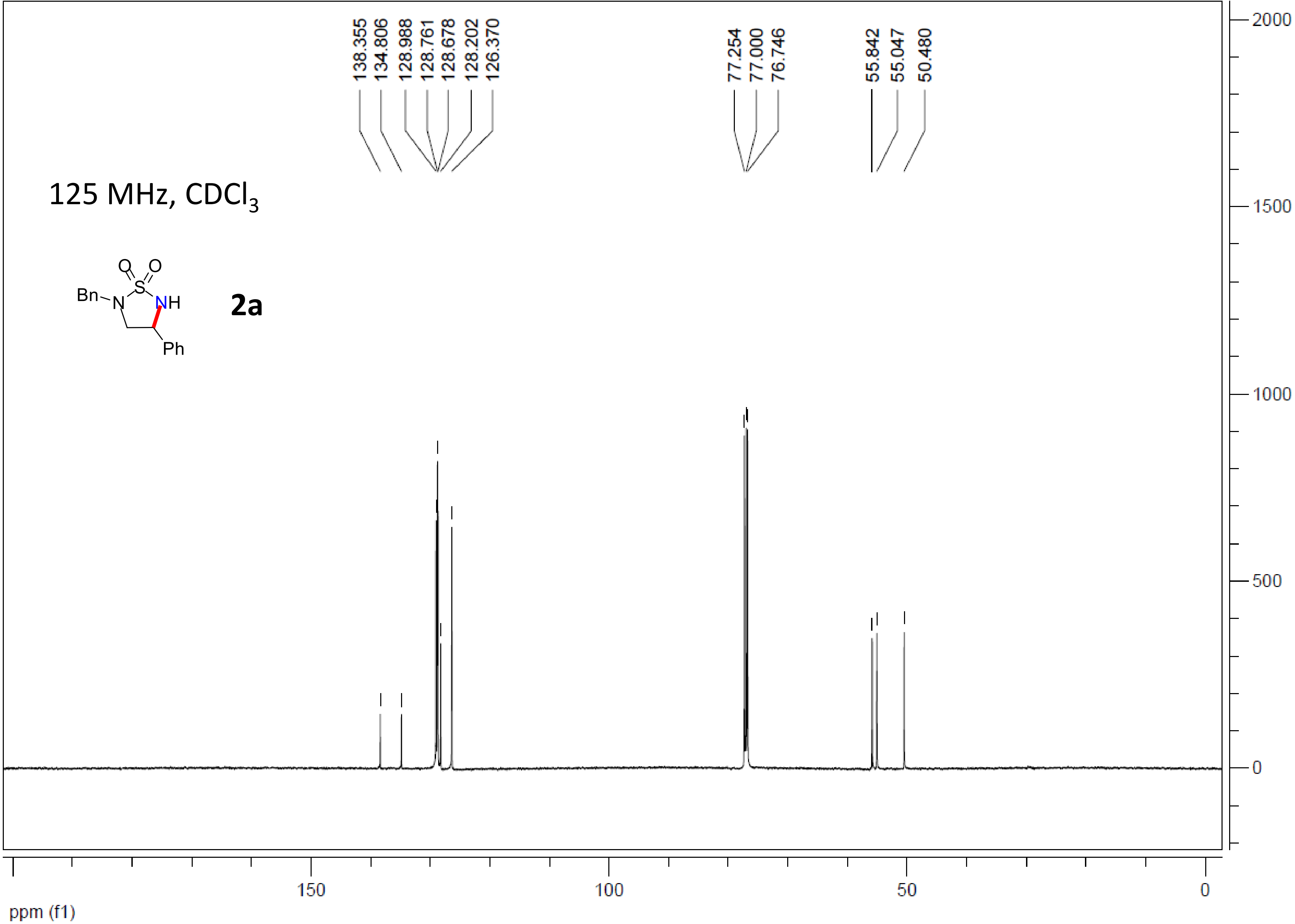
138.355
134.806
128.988
128.761
128.678
128.202
126.370
77.254
77.000
76.746
55.842
55.047
50.480
125 MHz, $CDCl_3$
Bn
N
S
O
O
NH
Ph
2a
2000
1500
1000
500
0
150
100
50
0
ppm (f1)

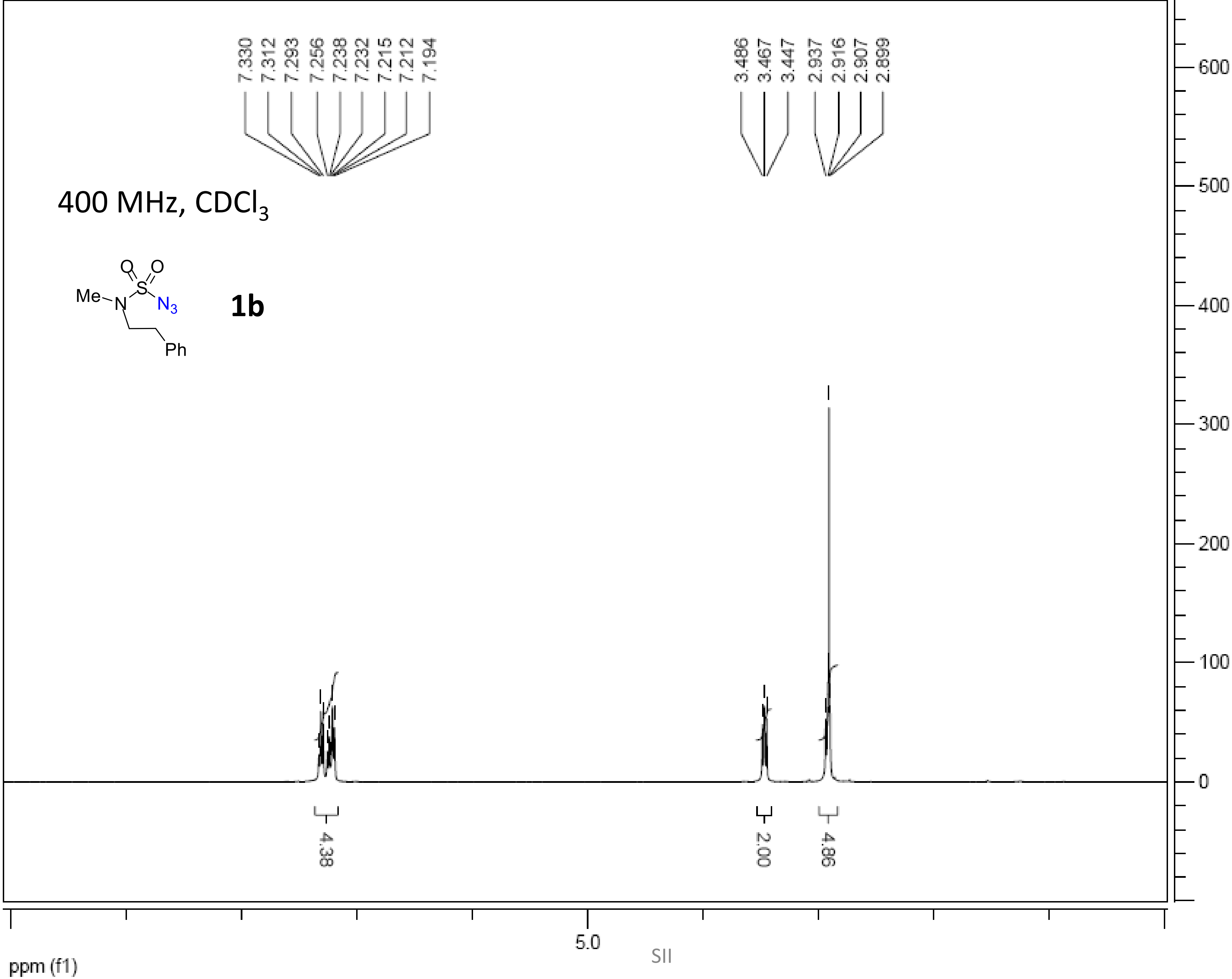
7.330
7.312
7.293
7.256
7.238
7.232
7.215
7.212
7.194
3.486
3.467
3.447
2.937
2.916
2.907
2.899
400 MHz, $CDCl_3$
Me
N
S
O
O
N_3
Ph
1b
600
500
400
300
200
100
0
4.38
2.00
4.86
5.0
ppm (f1)

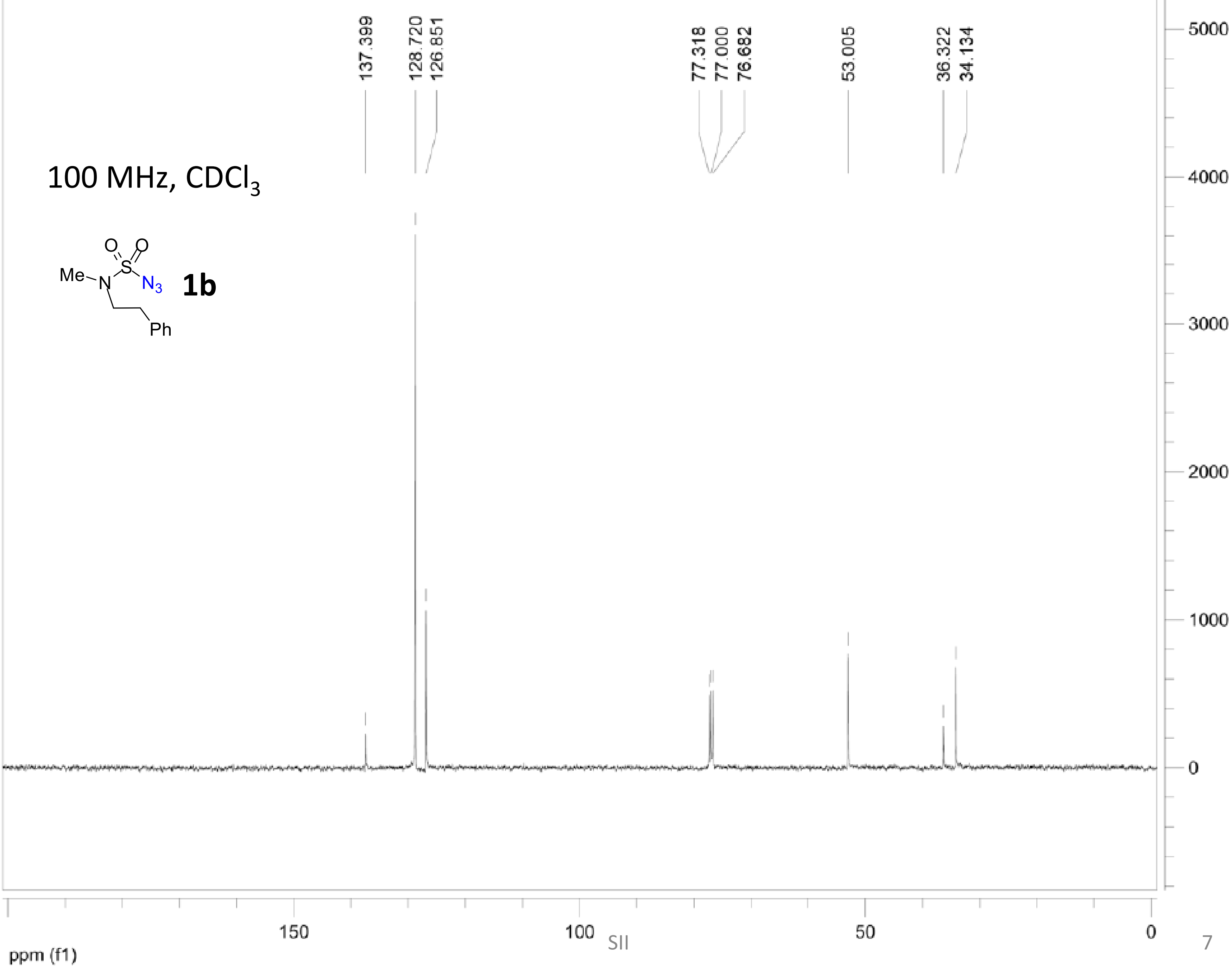
100 MHz, $CDCl_3$
1b
Me
N
S
O
O
N_3
Ph
137.399
128.720
126.851
77.318
77.000
76.682
53.005
36.322
34.134
5000
4000
3000
2000
1000
0
150
100
50
0
ppm (f1)

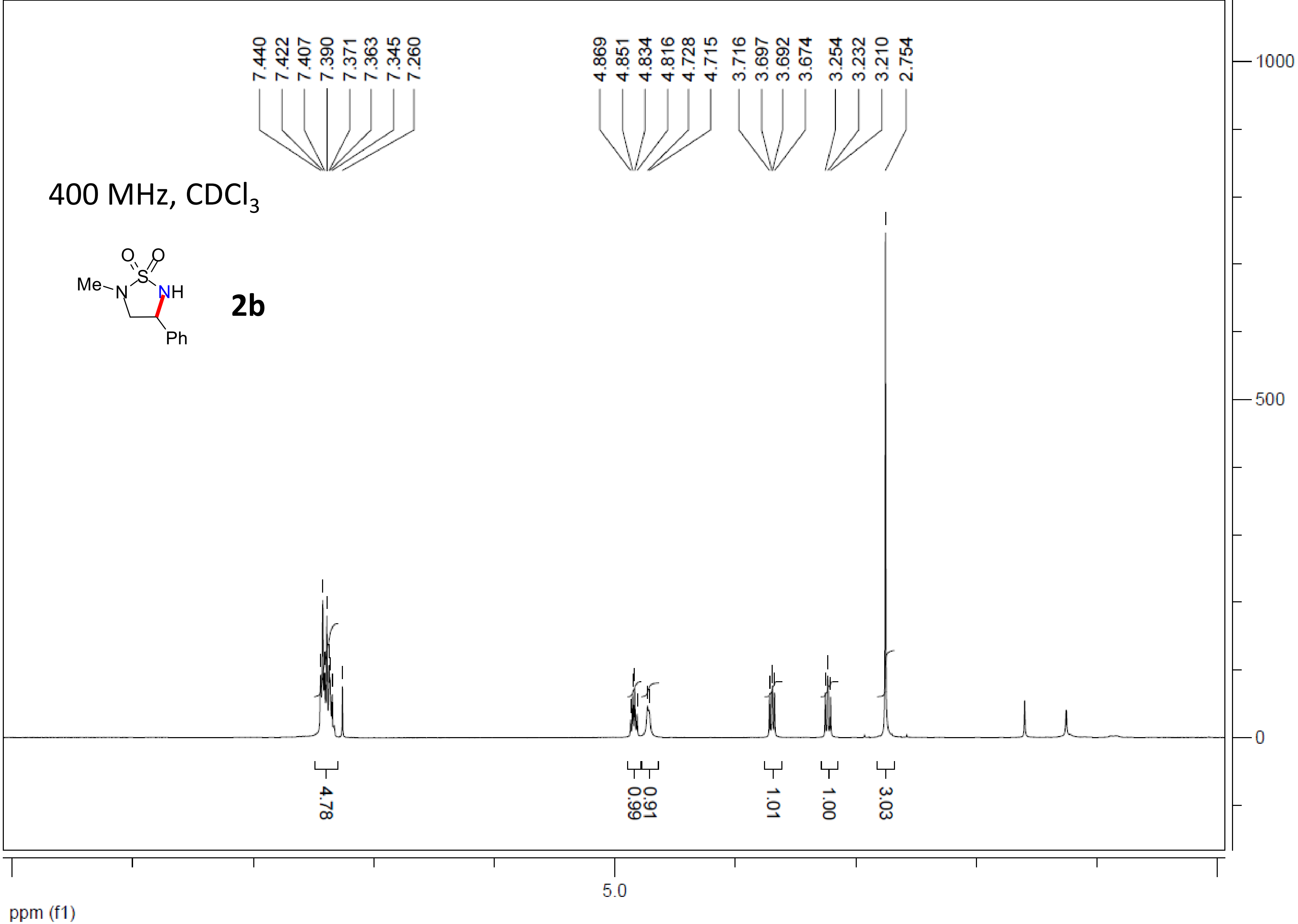
7.440
7.422
7.407
7.390
7.371
7.363
7.345
7.260
4.869
4.851
4.834
4.816
4.728
4.715
3.716
3.697
3.692
3.674
3.254
3.232
3.210
2.754
400 MHz, CDCl3
Me
N
S
O
O
NH
Ph
2b
1000
500
0
4.78
0.99
0.91
1.01
1.00
3.03
5.0
ppm (f1)

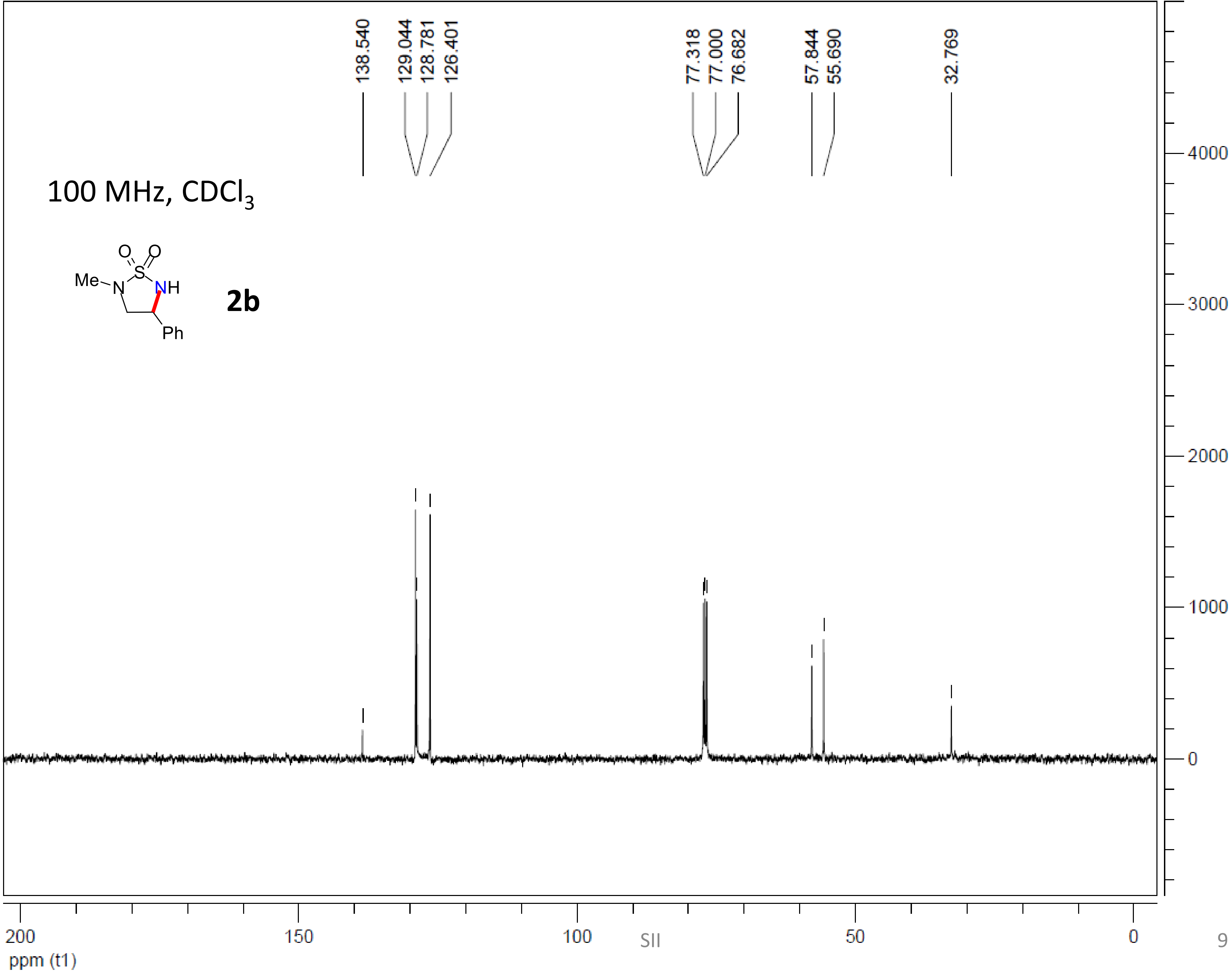
138.540
129.044
128.781
126.401
77.318
77.000
76.682
57.844
55.690
32.769
100 MHz, $CDCl_3$
O
O
Me
N
S
NH
Ph
2b
4000
3000
2000
1000
0
200
150
100
50
0
ppm (t1)

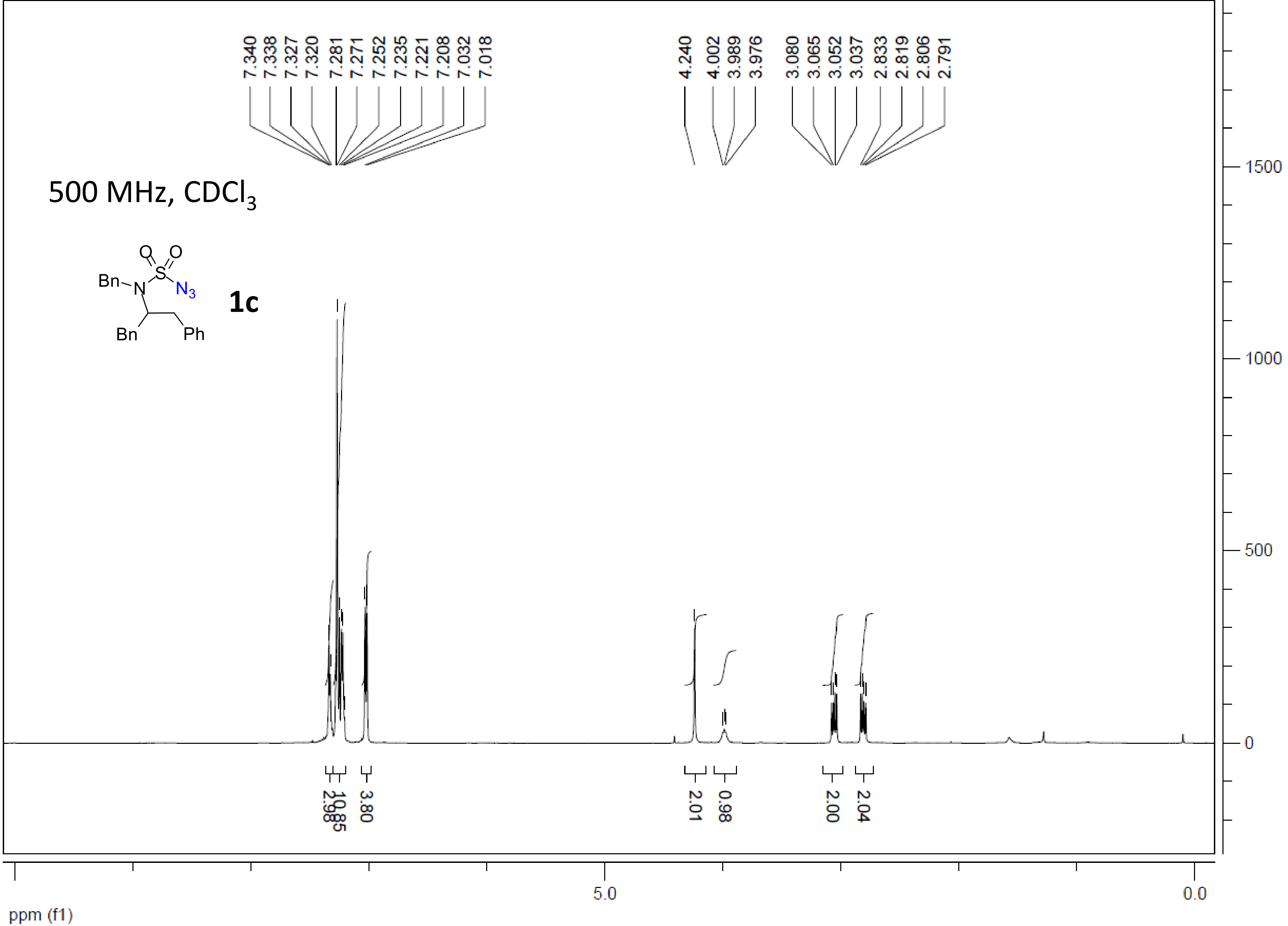
500 MHz, CDCl3
1c
7.340 7.338 7.327 7.320 7.281 7.271 7.252 7.235 7.221 7.208 7.032 7.018
4.240 4.002 3.989 3.976
3.080 3.065 3.052 3.037 2.833 2.819 2.806 2.791
2.98 10.85 3.80 2.01 0.98 2.00 2.04
1500
1000
500
0
5.0
0.0
ppm (f1)

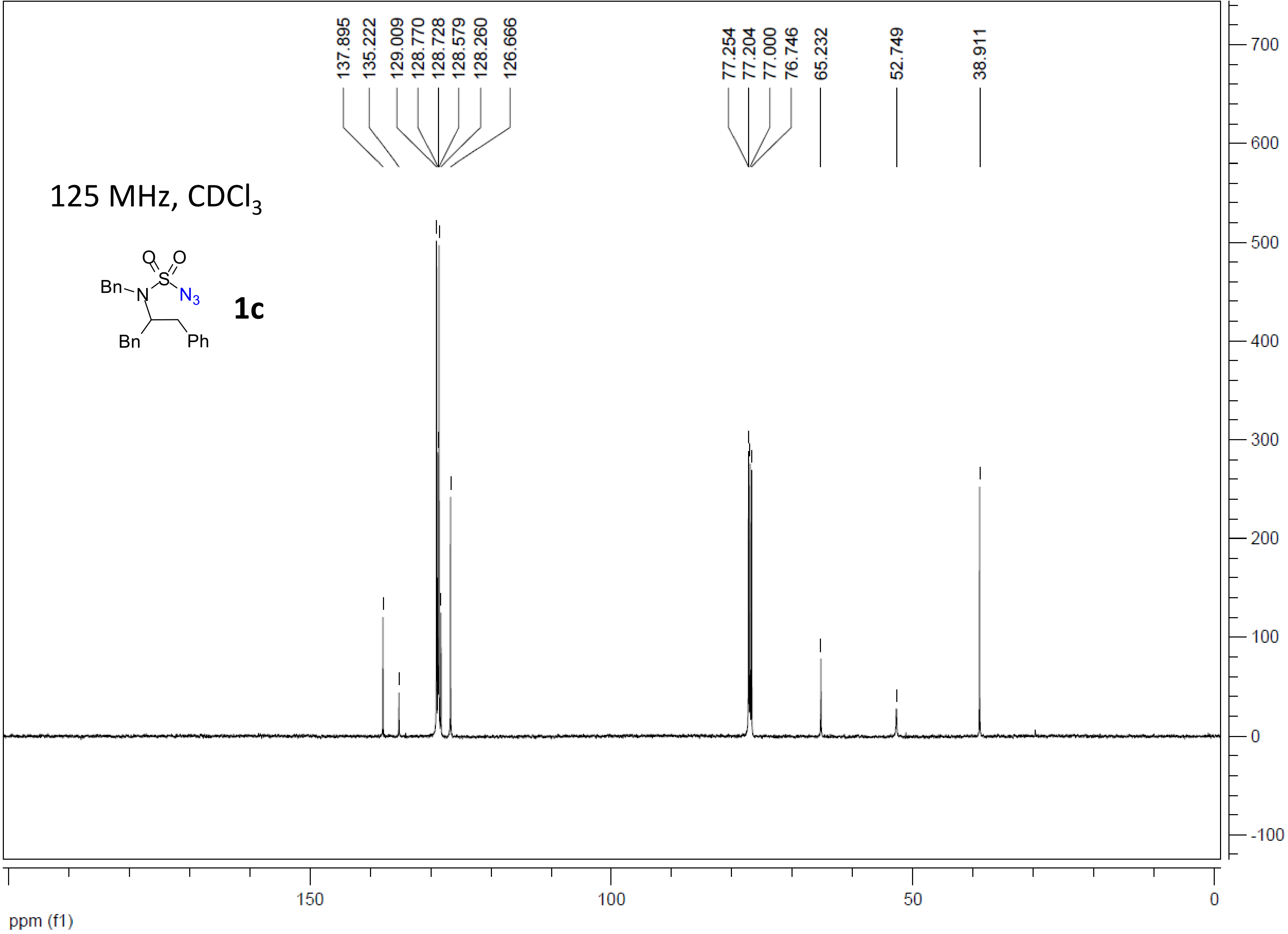

137.895
135.222
129.009
128.770
128.728
128.579
128.260
126.666
77.254
77.204
77.000
76.746
65.232
52.749
38.911
125 MHz, CDCl3
Bn
N
S
O
O
N3
Bn
Ph
1c
700
600
500
400
300
200
100
0
-100
150
100
50
0
ppm (f1)

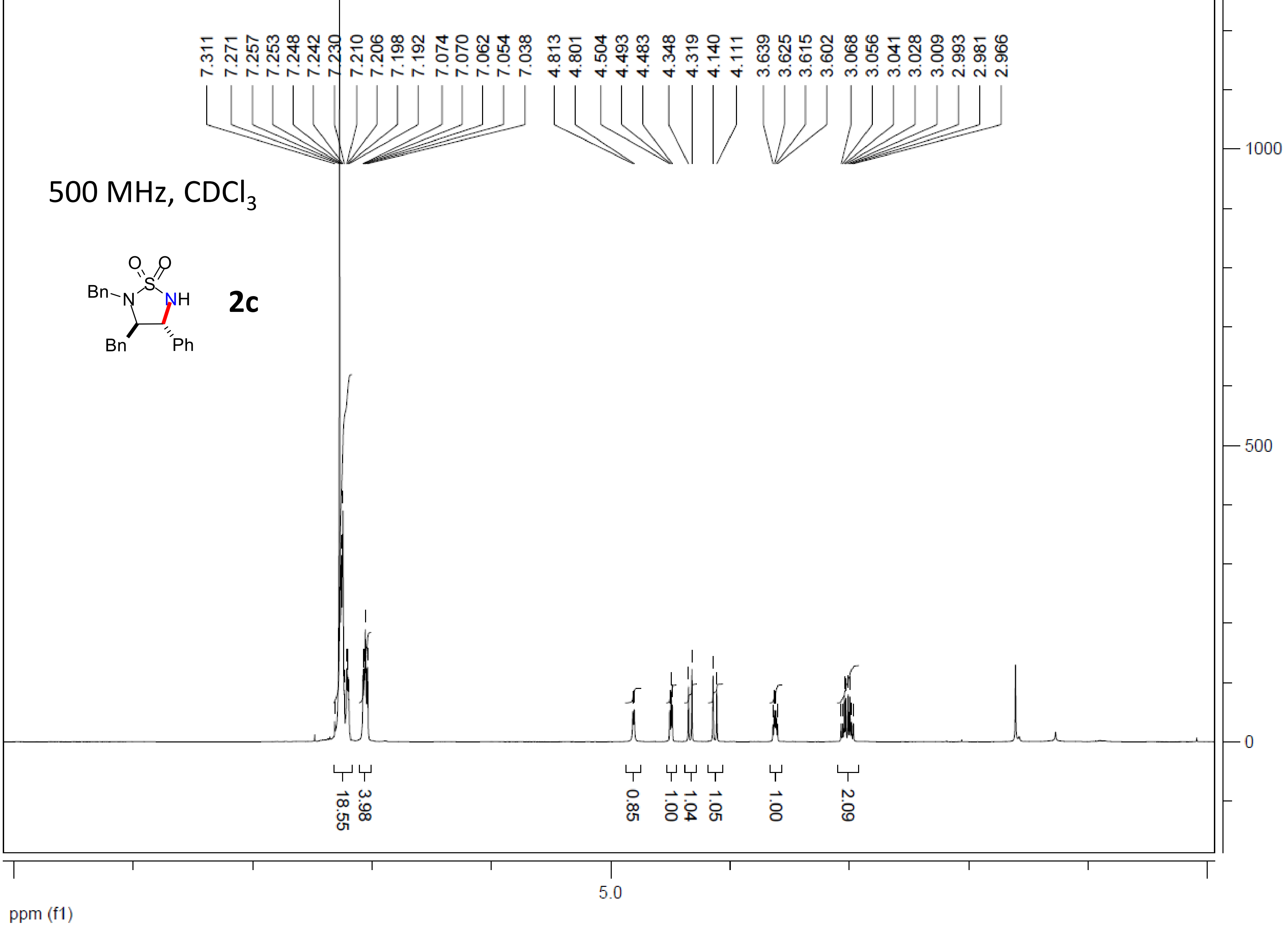
7.311 7.271 7.257 7.253 7.248 7.242 7.230 7.210 7.206 7.198 7.192 7.074 7.070 7.062 7.054 7.038 4.813 4.801 4.504 4.493 4.483 4.348 4.319 4.140 4.111 3.639 3.625 3.615 3.602 3.068 3.056 3.041 3.028 3.009 2.993 2.981 2.966
500 MHz, $CDCl_3$
Bn
N
S
O
O
NH
Bn
Ph
2c
1000
500
0
18.55
3.98
0.85
1.00
1.04
1.05
1.00
2.09
5.0
ppm (f1)

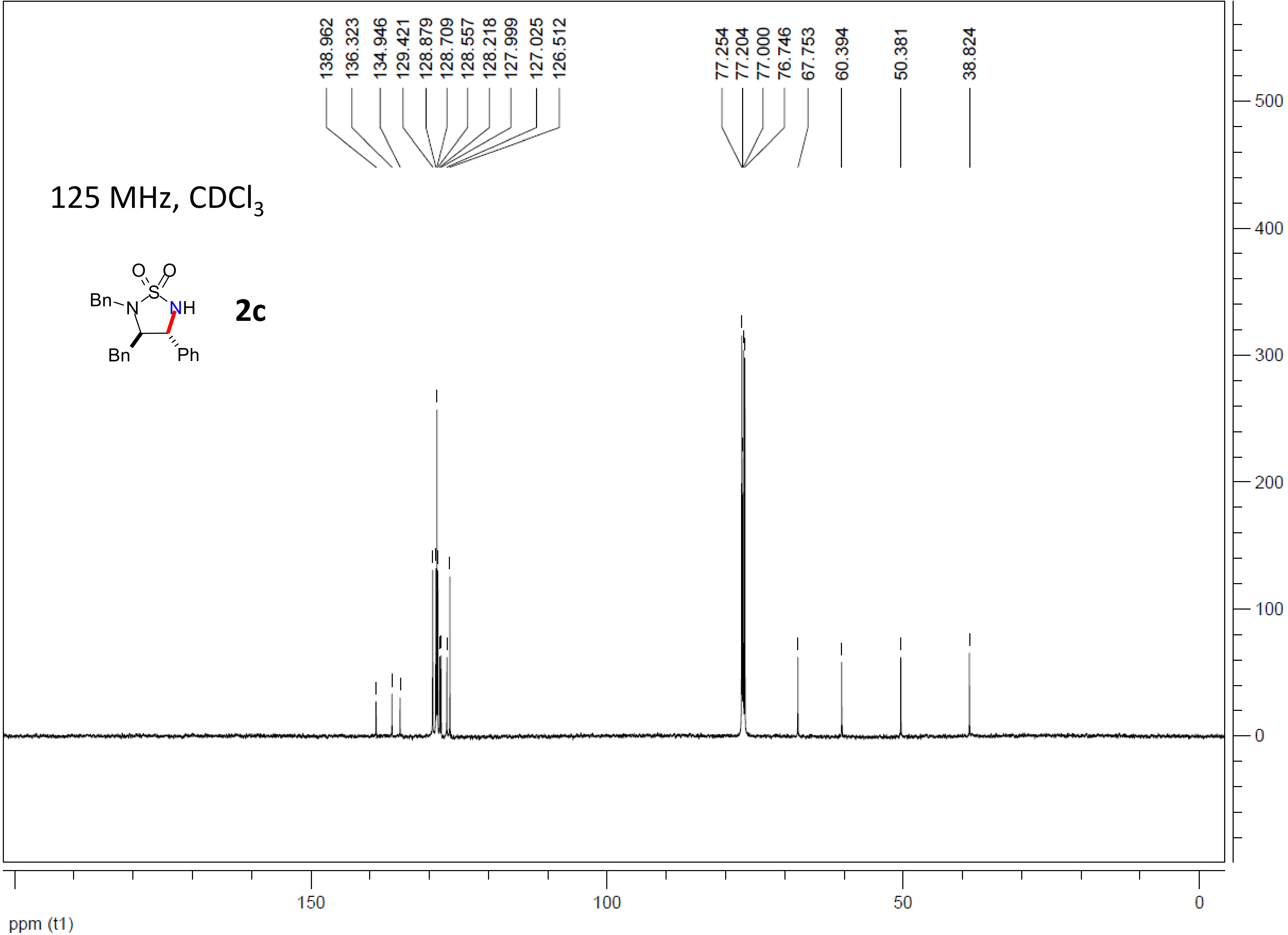
138.962
136.323
134.946
129.421
128.879
128.709
128.557
128.218
127.999
127.025
126.512
77.254
77.204
77.000
76.746
67.753
60.394
50.381
38.824
125 MHz, CDCl3
O O
S
Bn N NH
Bn Ph
2c
500
400
300
200
100
0
150
100
50
0
ppm (t1)

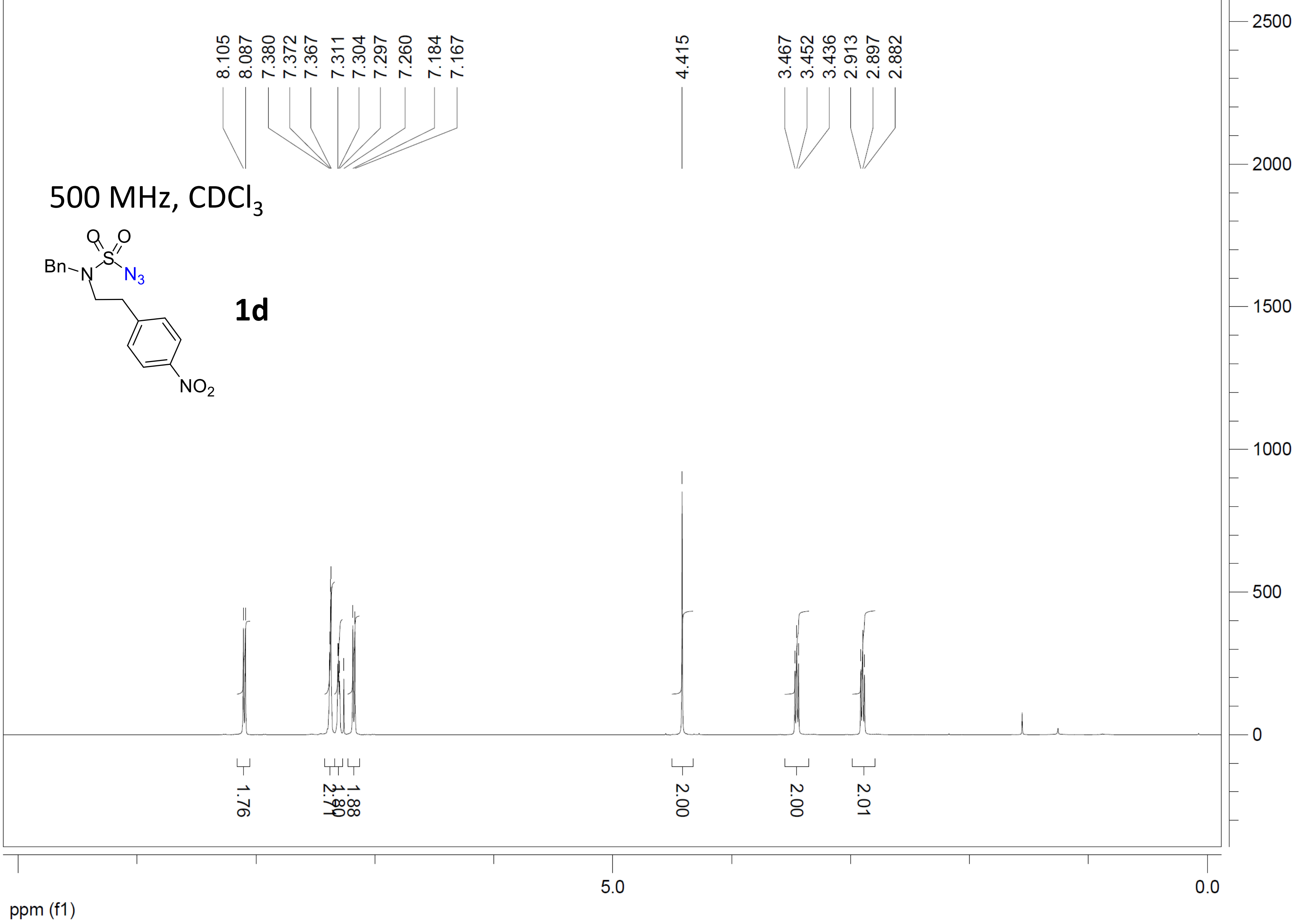
500 MHz, CDCl3
1d
Bn
N
S
O
O
N3
NO2
8.105
8.087
7.380
7.372
7.367
7.311
7.304
7.297
7.260
7.184
7.167
4.415
3.467
3.452
3.436
2.913
2.897
2.882
1.76
2.71
1.80
1.88
2.00
2.00
2.01
5.0
0.0
ppm (f1)
2500
2000
1500
1000
500
0

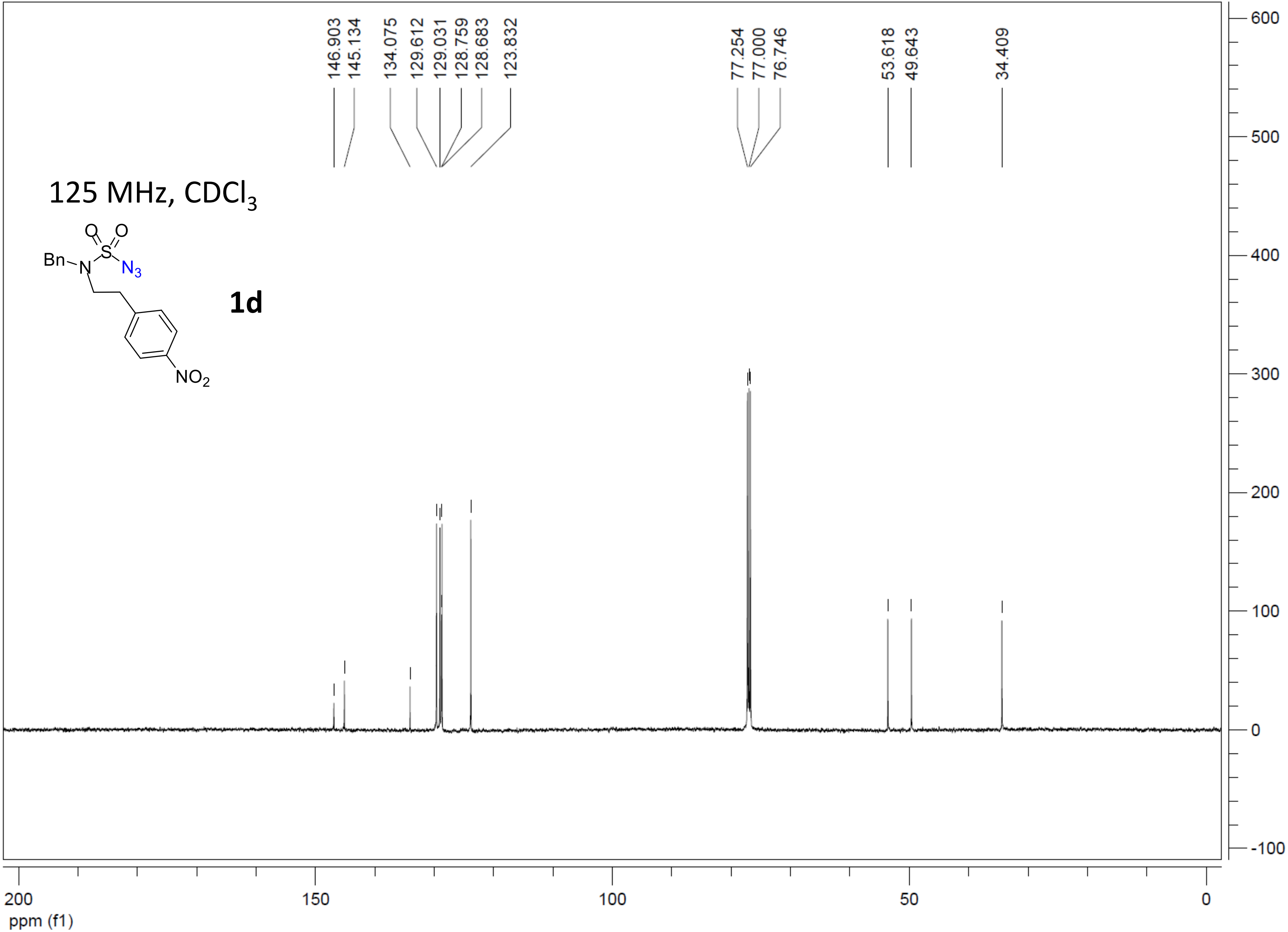

146.903
145.134
134.075
129.612
129.031
128.759
128.683
123.832
77.254
77.000
76.746
53.618
49.643
34.409
125 MHz, $CDCl_3$
Bn
N
S
O
O
N_3
NO_2
1d
600
500
400
300
200
100
0
-100
200
150
100
50
0
ppm (f1)

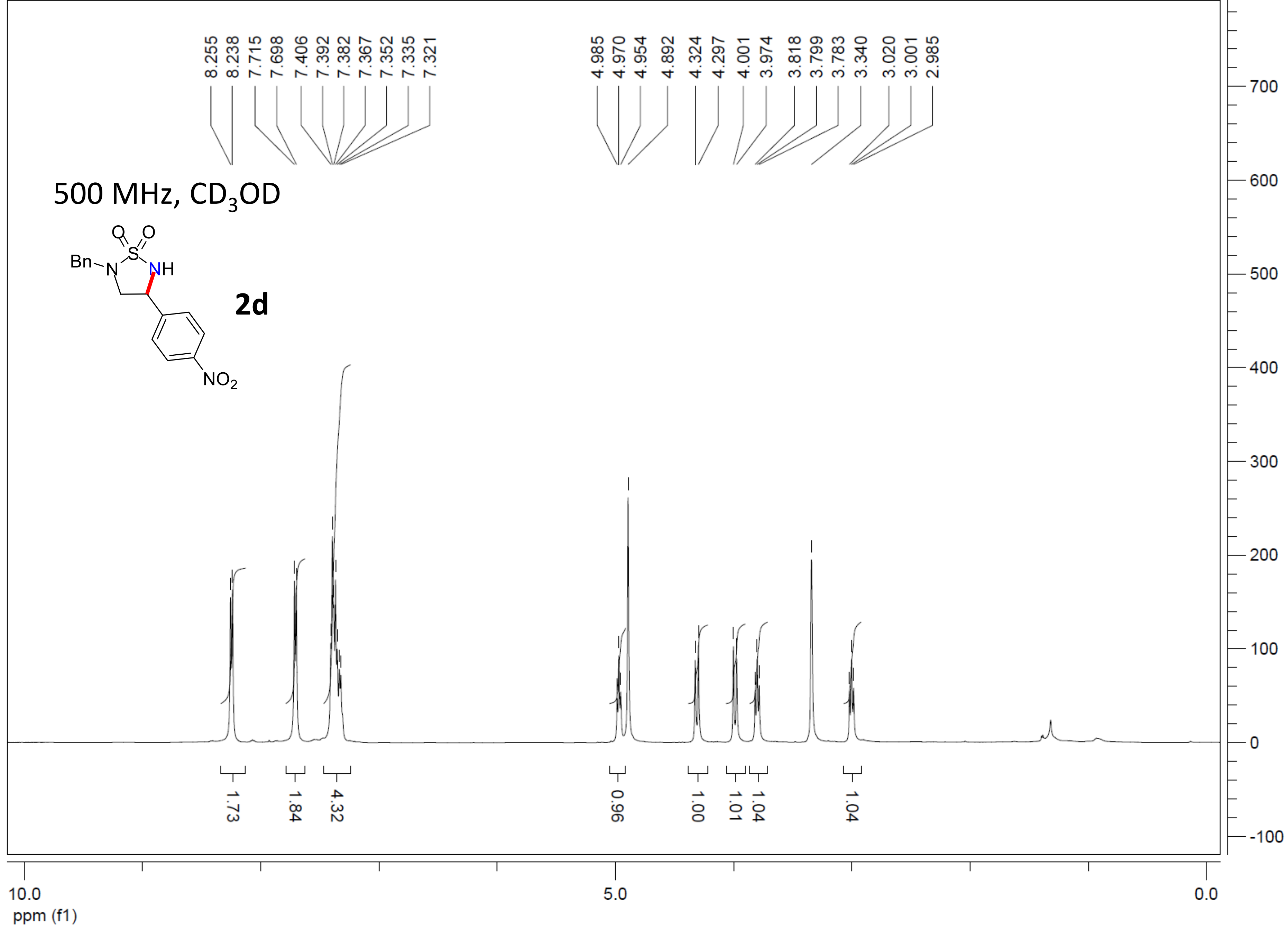

8.255
8.238
7.715
7.698
7.406
7.392
7.382
7.367
7.352
7.335
7.321
4.985
4.970
4.954
4.892
4.324
4.297
4.001
3.974
3.818
3.799
3.783
3.340
3.020
3.001
2.985
500 MHz, CD_3OD
Bn
N
S
O
O
NH
NO_2
2d
1.73
1.84
4.32
0.96
1.00
1.01
1.04
1.04
700
600
500
400
300
200
100
0
-100
10.0
5.0
0.0
ppm (f1)

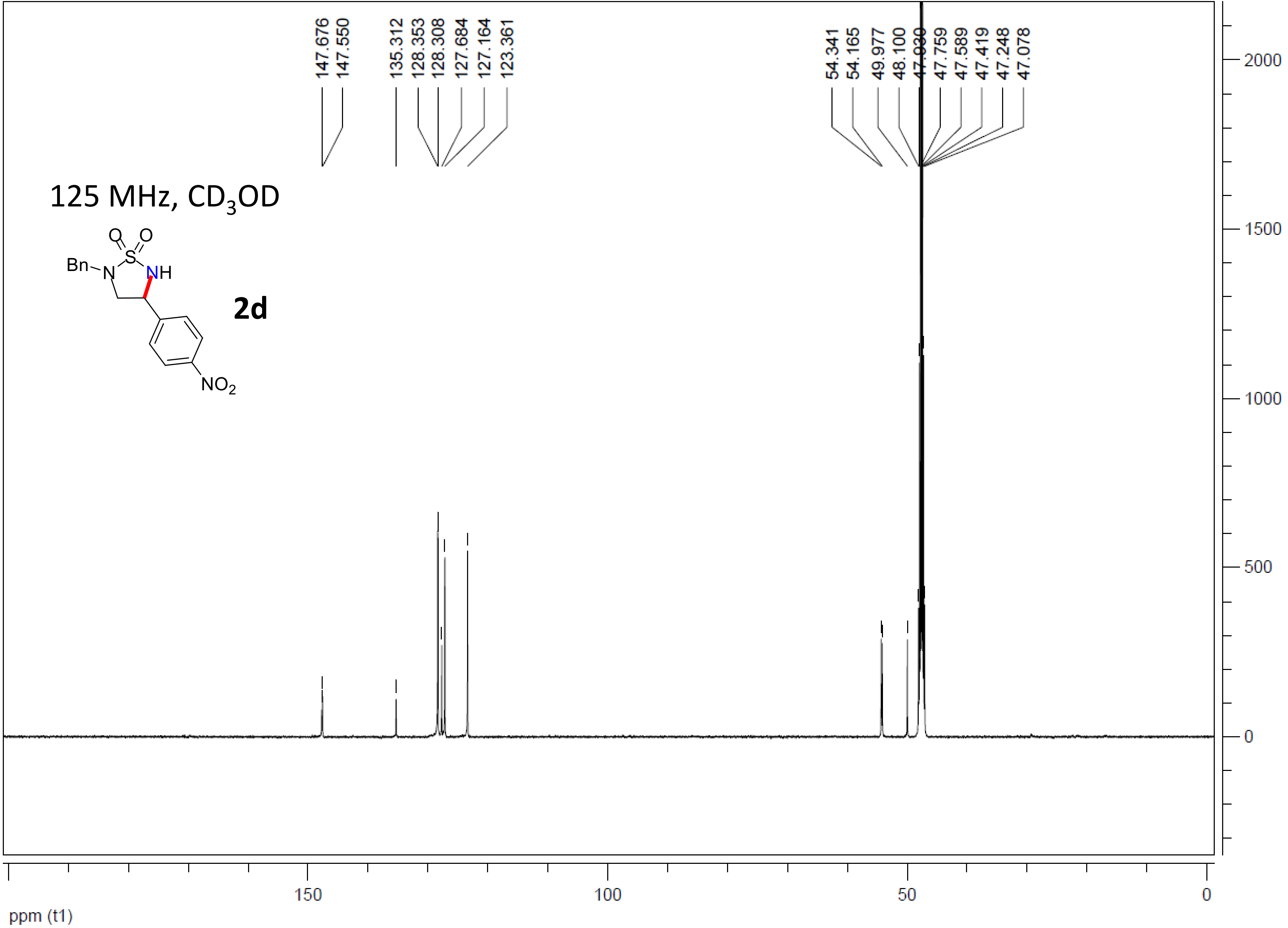
147.676
147.550
135.312
128.353
128.308
127.684
127.164
123.361
54.341
54.165
49.977
48.100
47.759
47.589
47.419
47.248
47.078
125 MHz, CD3OD
Bn
N
S
O
O
NH
NO2
2d
2000
1500
1000
500
0
150
100
50
0
ppm (t1)

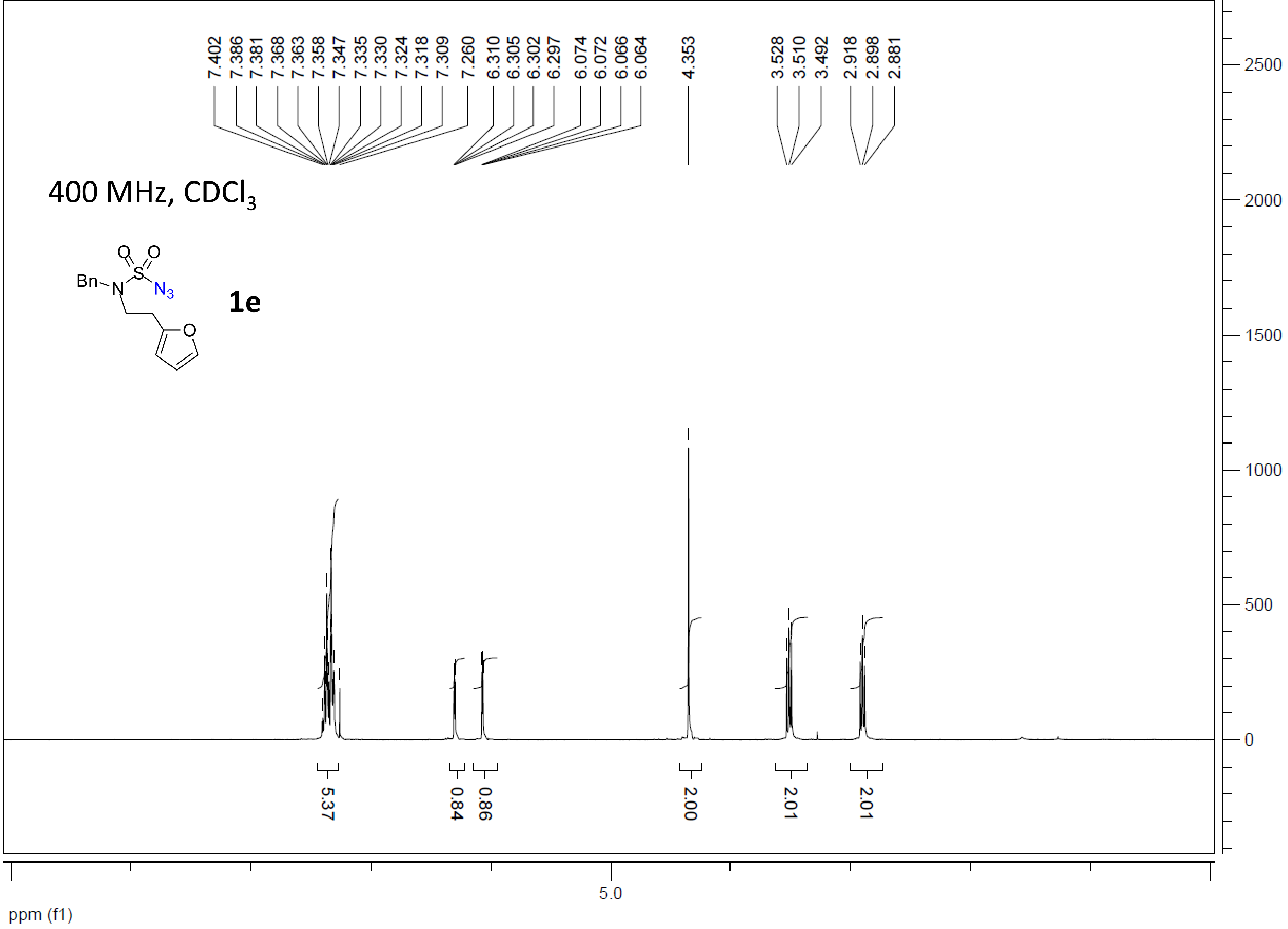
7.402 7.386 7.381 7.368 7.363 7.358 7.347 7.335 7.330 7.324 7.318 7.309 7.260 6.310 6.305 6.302 6.297 6.074 6.072 6.066 6.064 4.353 3.528 3.510 3.492 2.918 2.898 2.881
400 MHz, CDCl3
Bn
N
S
O
O
N3
O
1e
2500
2000
1500
1000
500
0
5.37
0.84
0.86
2.00
2.01
2.01
5.0
ppm (f1)

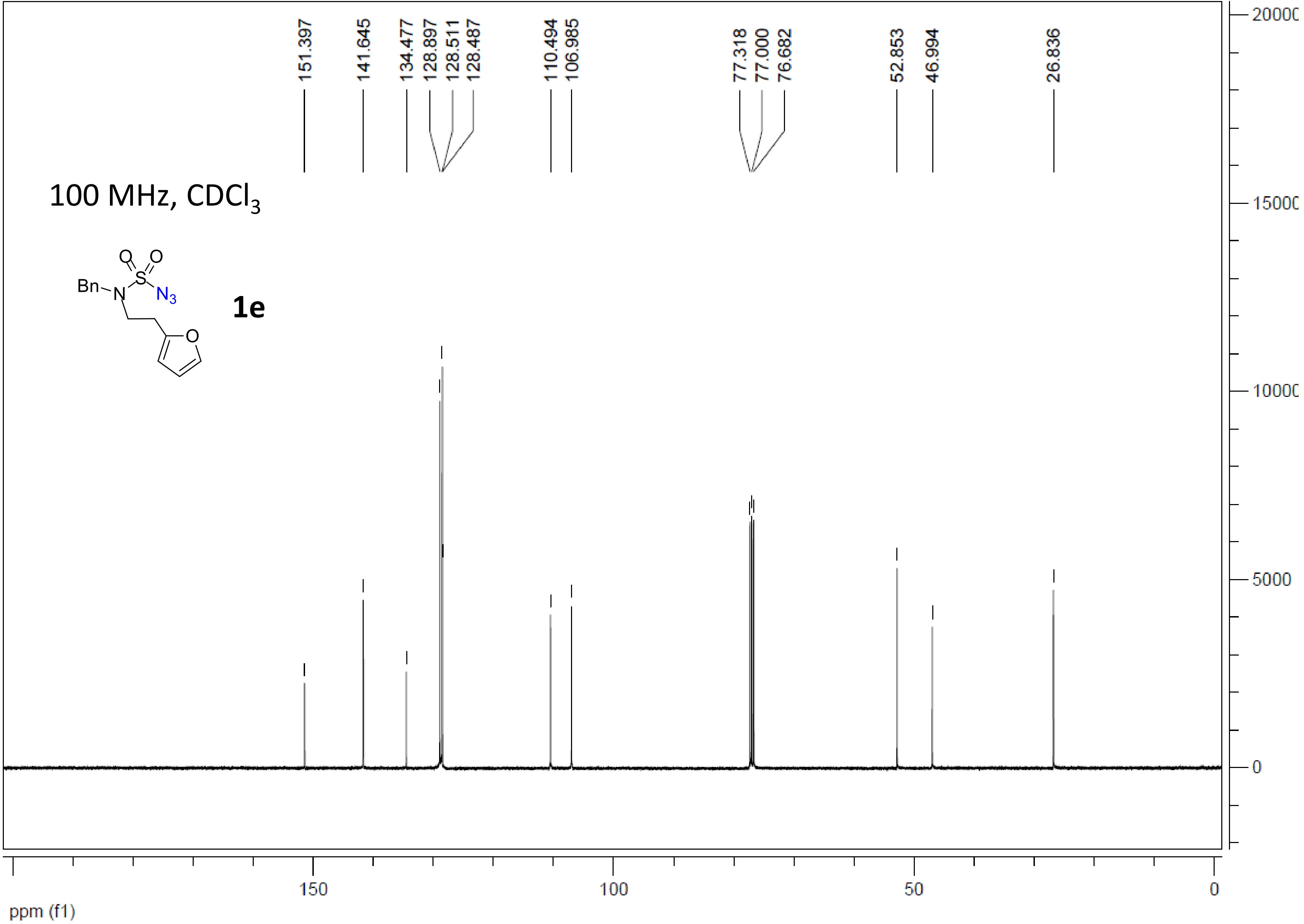
151.397
141.645
134.477
128.897
128.511
128.487
110.494
106.985
77.318
77.000
76.682
52.853
46.994
26.836
100 MHz, $CDCl_3$
Bn
N
S
O
O
N_3
O
1e
20000
15000
10000
5000
0
150
100
50
0
ppm (f1)

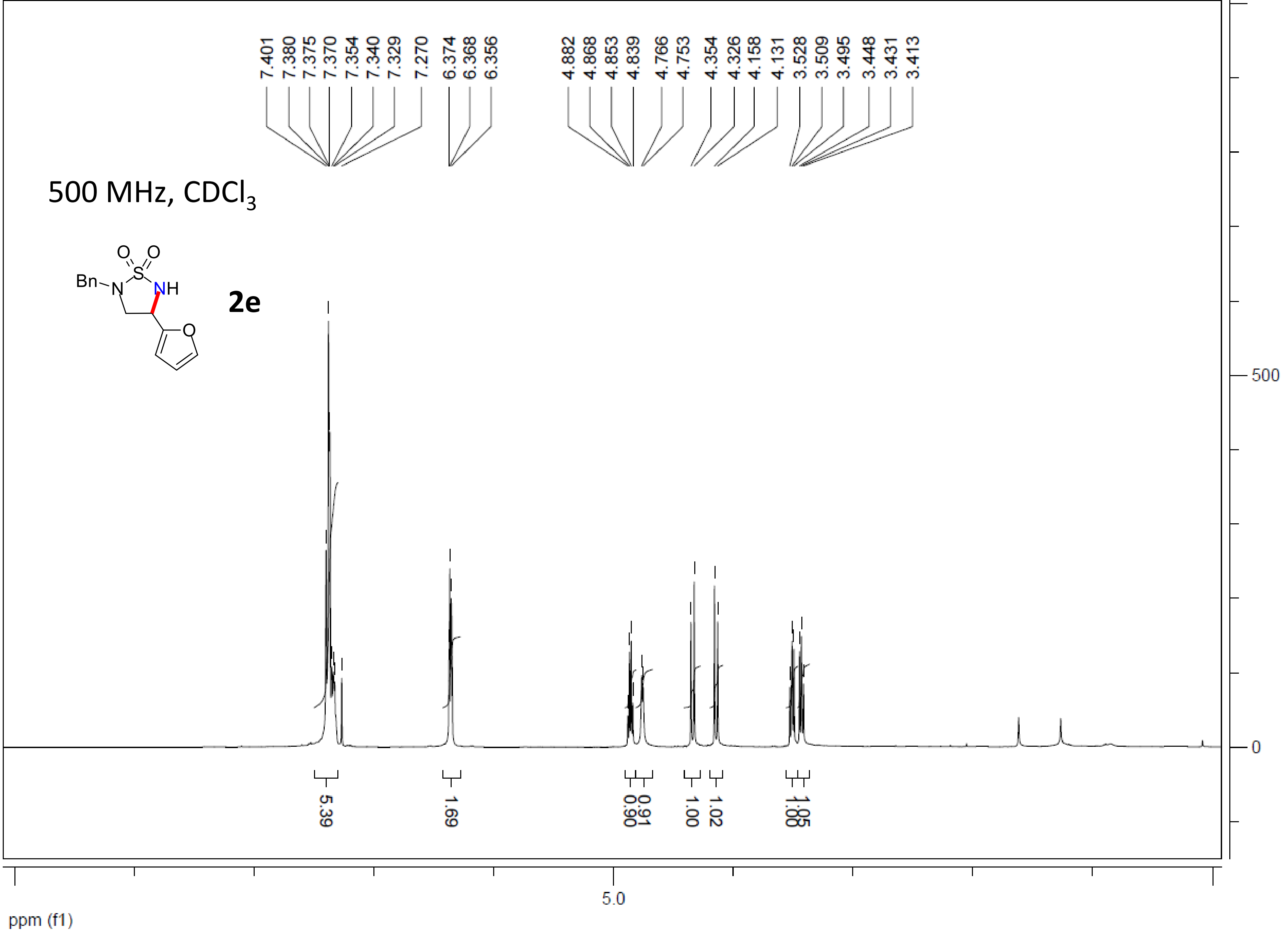
500 MHz, CDCl3
Bn
N
S
O
O
NH
O
2e
7.401
7.380
7.375
7.370
7.354
7.340
7.329
7.270
6.374
6.368
6.356
4.882
4.868
4.853
4.839
4.766
4.753
4.354
4.326
4.158
4.131
3.528
3.509
3.495
3.448
3.431
3.413
500
0
5.39
1.69
0.90
0.91
1.00
1.02
1.05
5.0
ppm (f1)

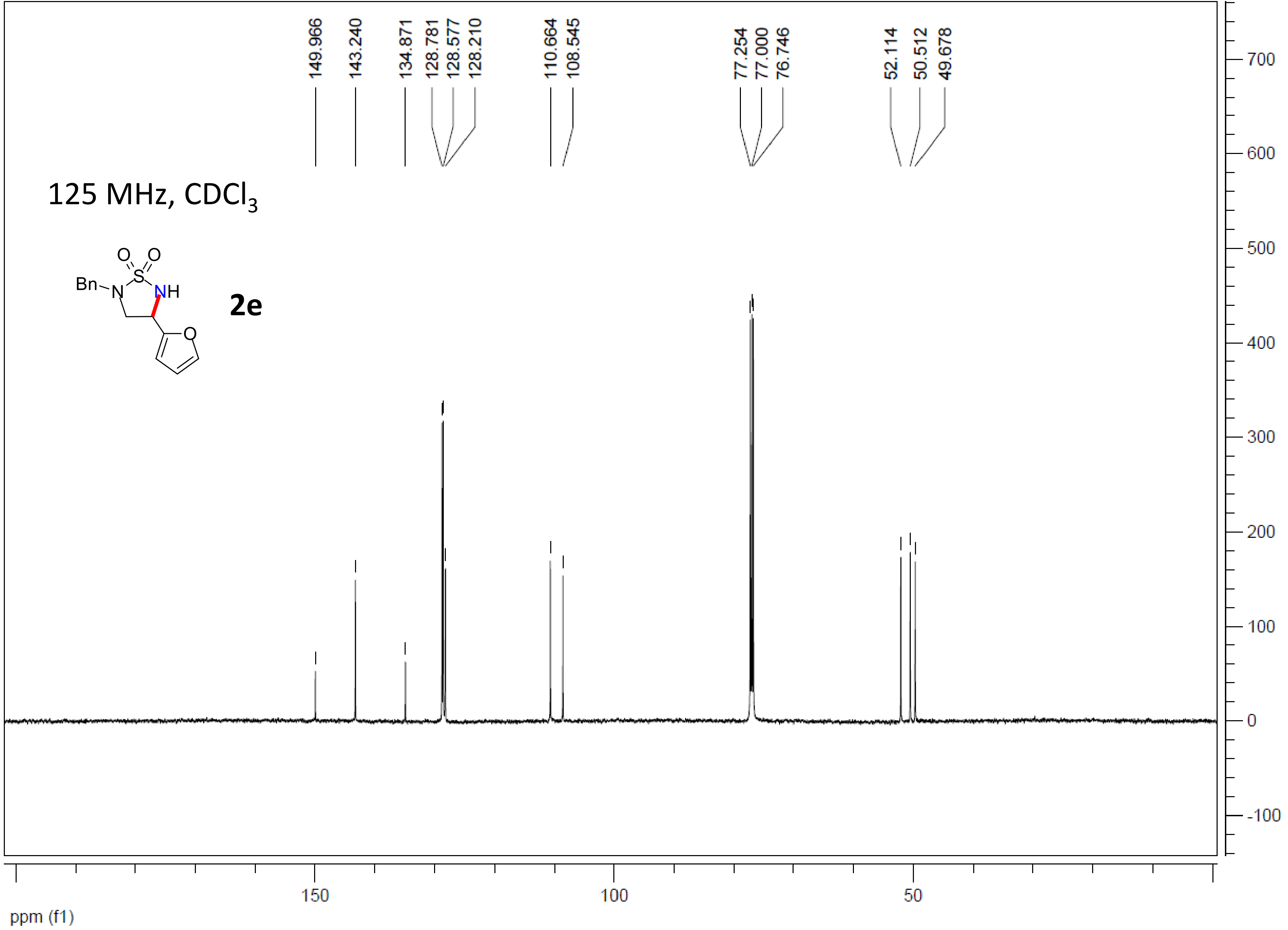

149.966
143.240
134.871
128.781
128.577
128.210
110.664
108.545
77.254
77.000
76.746
52.114
50.512
49.678
125 MHz, $CDCl_3$
Bn
N
S
O
O
NH
O
2e
700
600
500
400
300
200
100
0
-100
150
100
50
ppm (f1)

SII

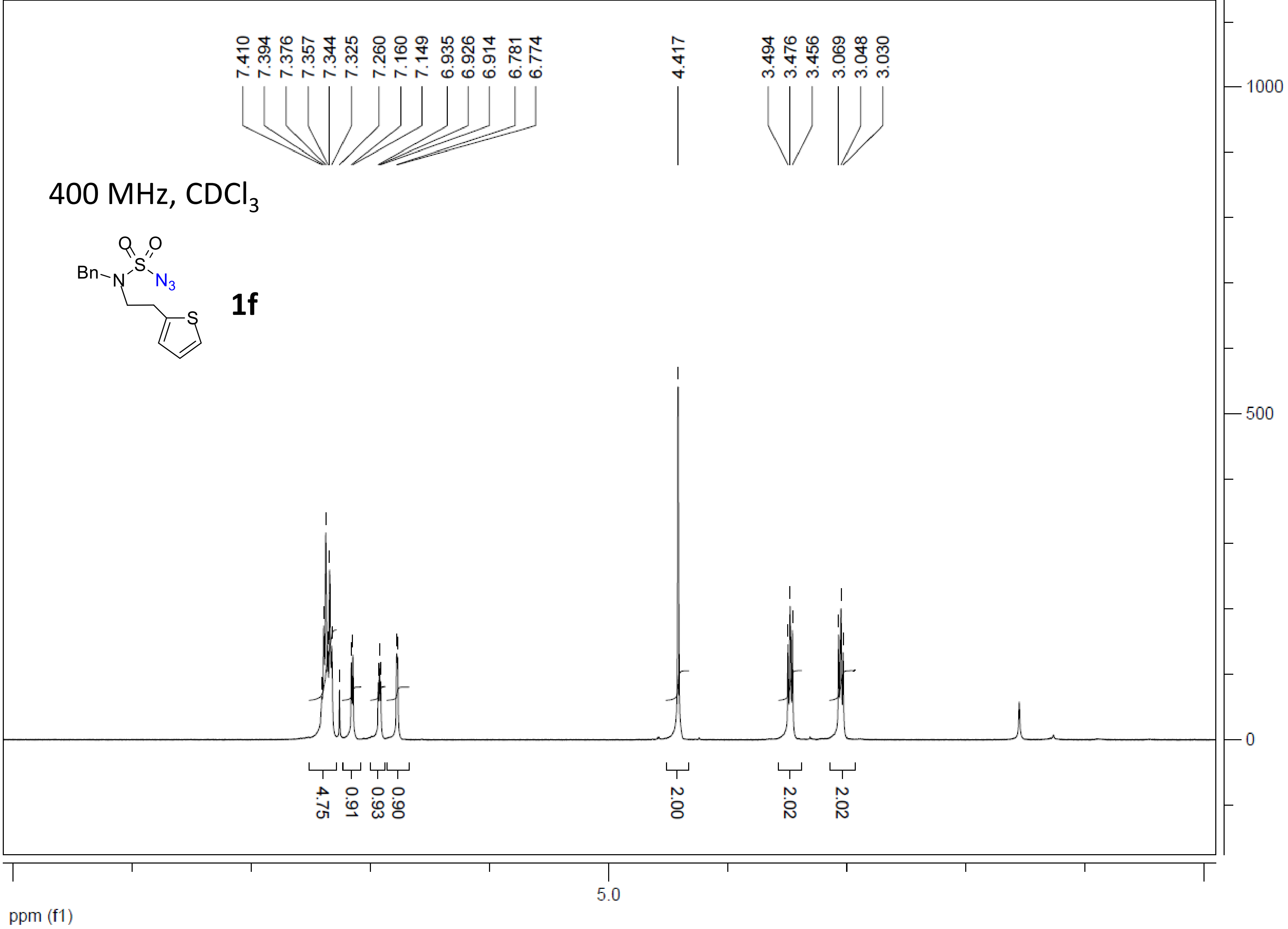
7.410 7.394 7.376 7.357 7.344 7.325 7.260 7.160 7.149 6.935 6.926 6.914 6.781 6.774
4.417
3.494 3.476 3.456 3.069 3.048 3.030
400 MHz, $CDCl_3$
Bn
N
S
O
O
N_3
S
1f
4.75 0.91 0.93 0.90
2.00
2.02 2.02
1000
500
0
5.0
ppm (f1)

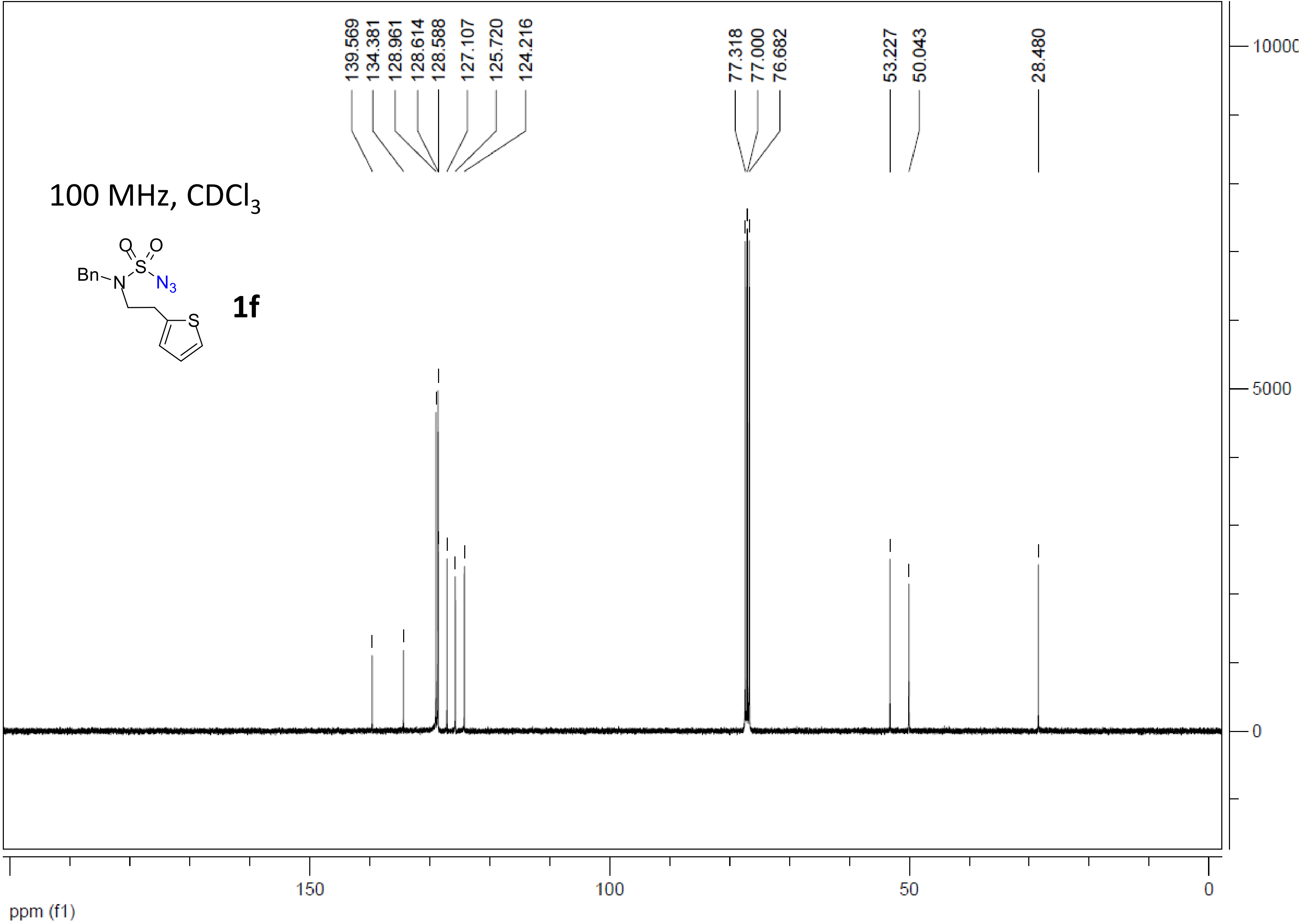
100 MHz, $CDCl_3$
Bn
N
S
O
O
N_3
S
1f
139.569
134.381
128.961
128.614
128.588
127.107
125.720
124.216
77.318
77.000
76.682
53.227
50.043
28.480
10000
5000
0
150
100
50
0
ppm (f1)

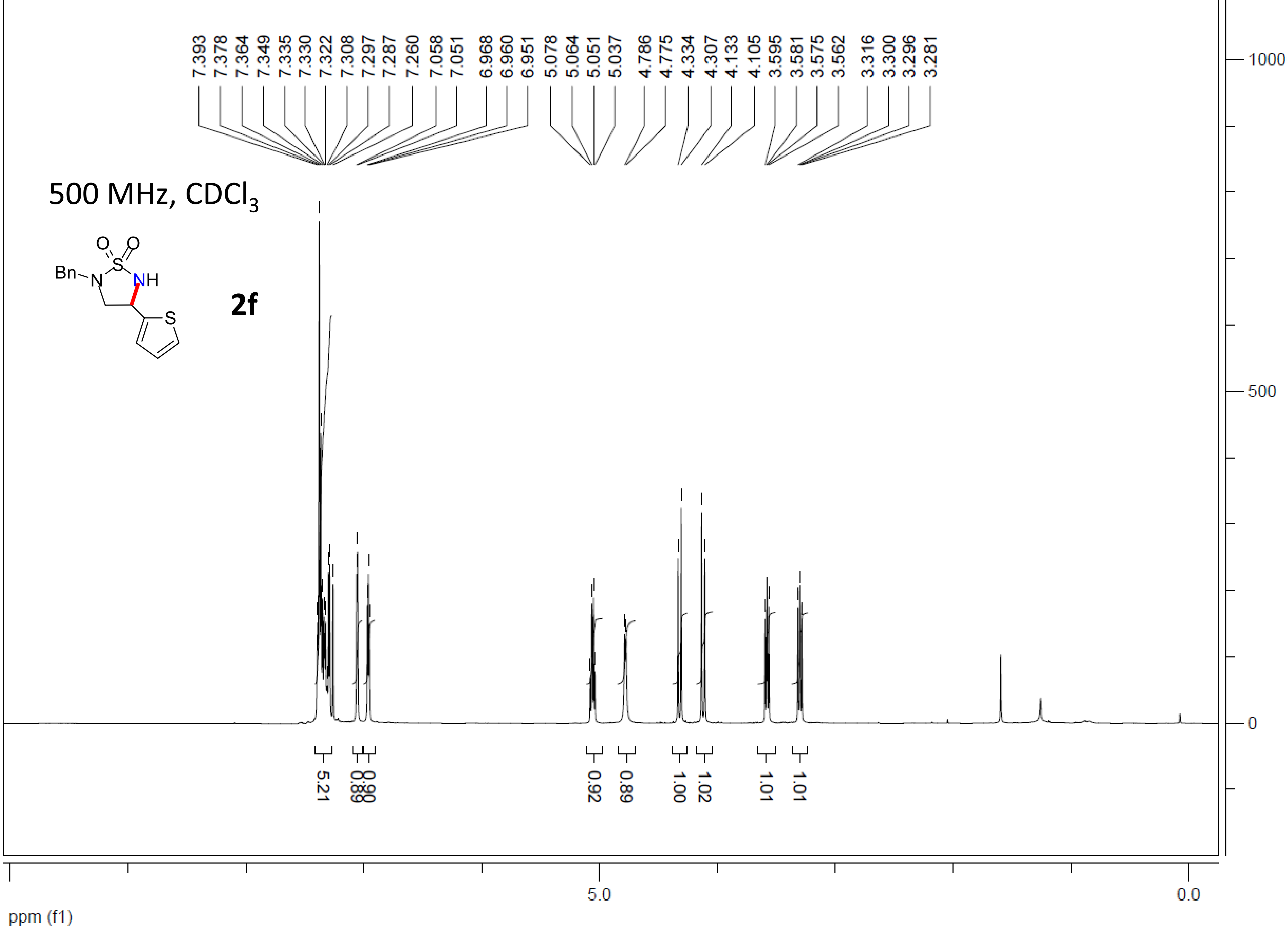
7.393 7.378 7.364 7.349 7.335 7.330 7.322 7.308 7.297 7.287 7.260 7.058 7.051 6.968 6.960 6.951 5.078 5.064 5.051 5.037 4.786 4.775 4.334 4.307 4.133 4.105 3.595 3.581 3.575 3.562 3.316 3.300 3.296 3.281
500 MHz, $CDCl_3$
Bn
N
S
O
O
NH
S
2f
1000
500
0
5.21
0.89
0.90
0.92
0.89
1.00
1.02
1.01
1.01
5.0
0.0
ppm (f1)

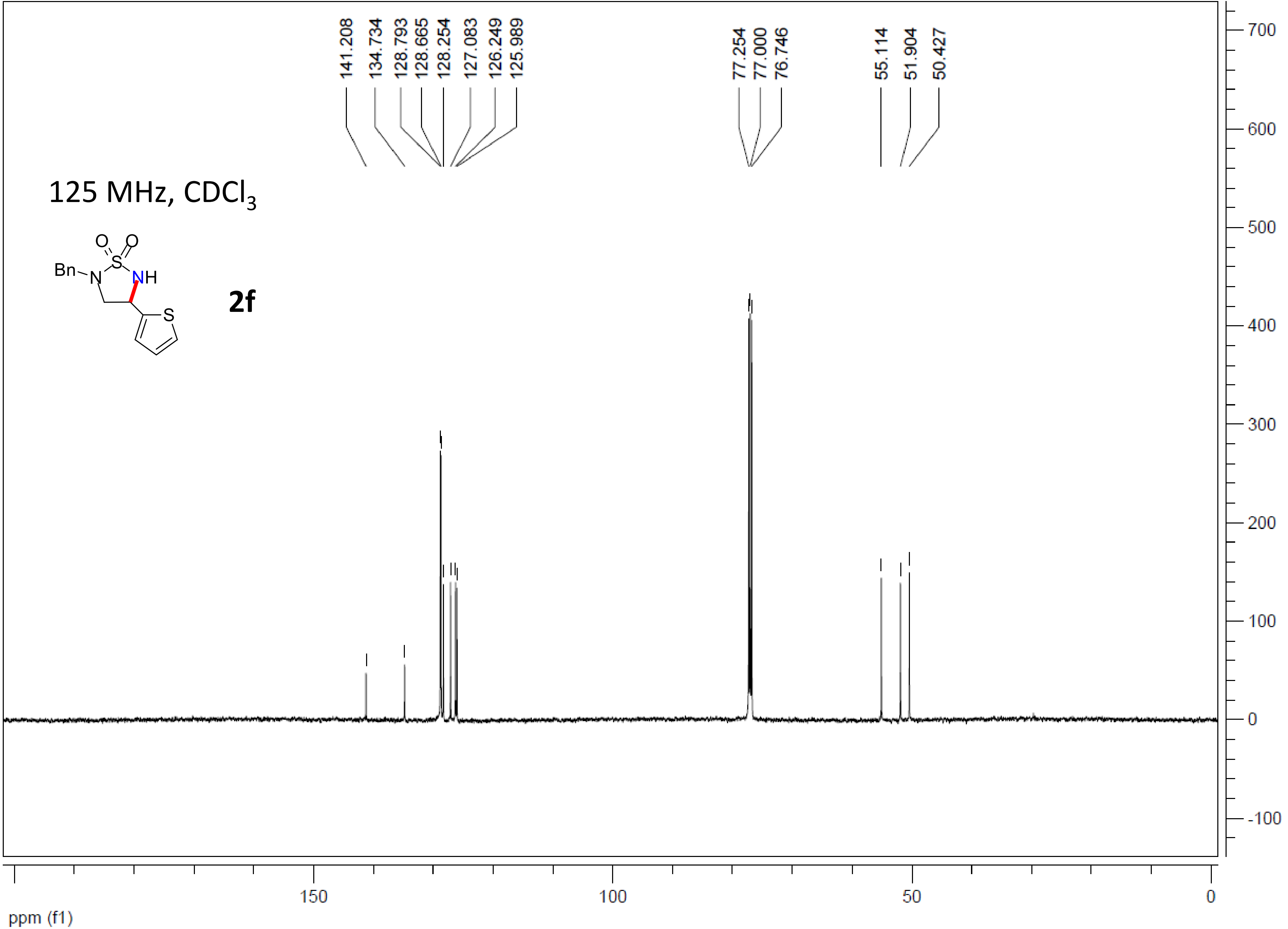
141.208
134.734
128.793
128.665
128.254
127.083
126.249
125.989
77.254
77.000
76.746
55.114
51.904
50.427
125 MHz, CDCl3
Bn
N
S
O
O
NH
S
2f
700
600
500
400
300
200
100
0
-100
150
100
50
0
ppm (f1)

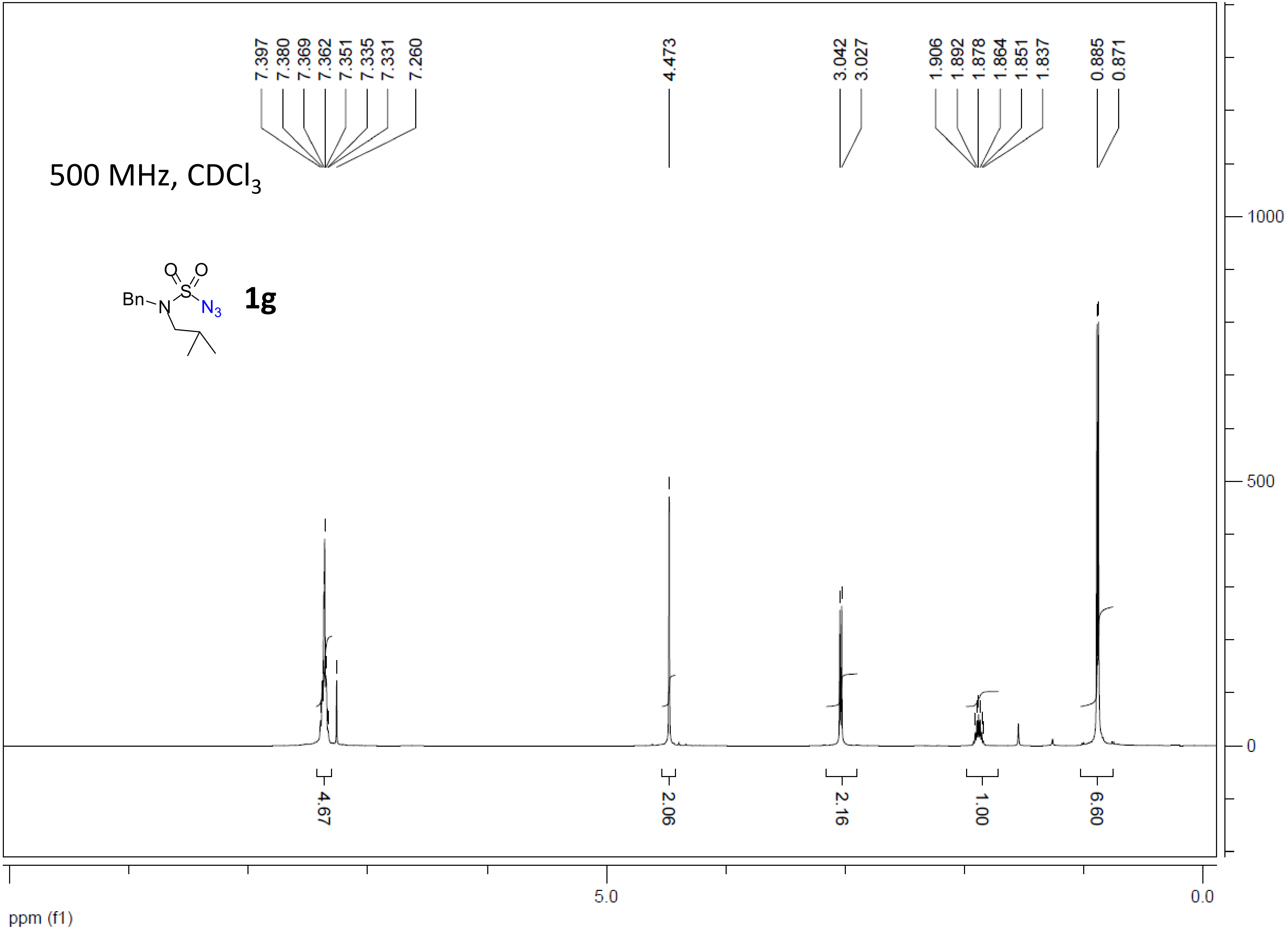
7.397
7.380
7.369
7.362
7.351
7.335
7.331
7.260
4.473
3.042
3.027
1.906
1.892
1.878
1.864
1.851
1.837
0.885
0.871
500 MHz, CDCl3
Bn
N
S
O
O
N3
1g
1000
500
0
4.67
2.06
2.16
1.00
6.60
5.0
0.0
ppm (f1)

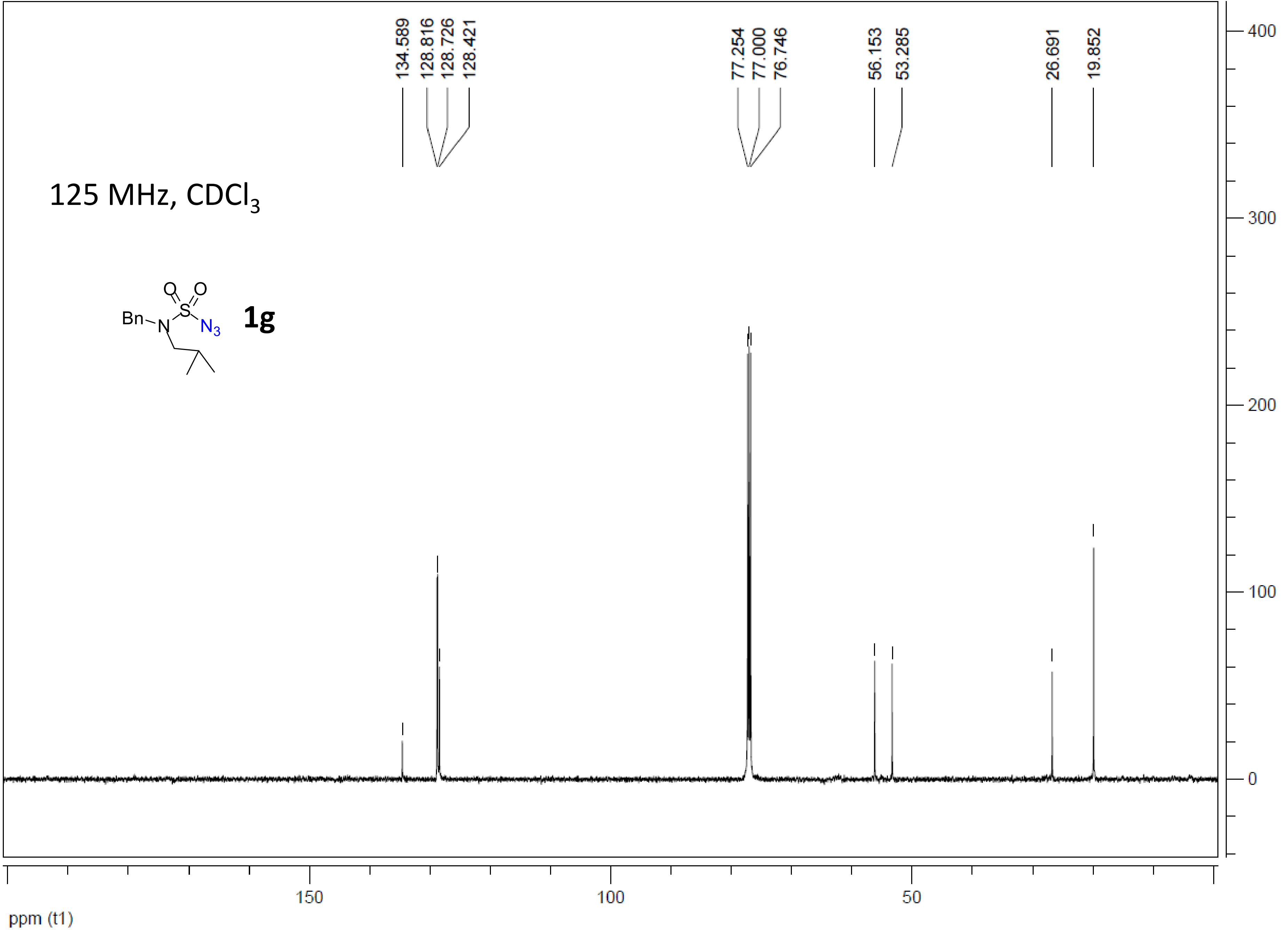
134.589
128.816
128.726
128.421
77.254
77.000
76.746
56.153
53.285
26.691
19.852
125 MHz, $CDCl_3$
Bn
N
S
O
O
N_3
1g
400
300
200
100
0
150
100
50
ppm (t1)

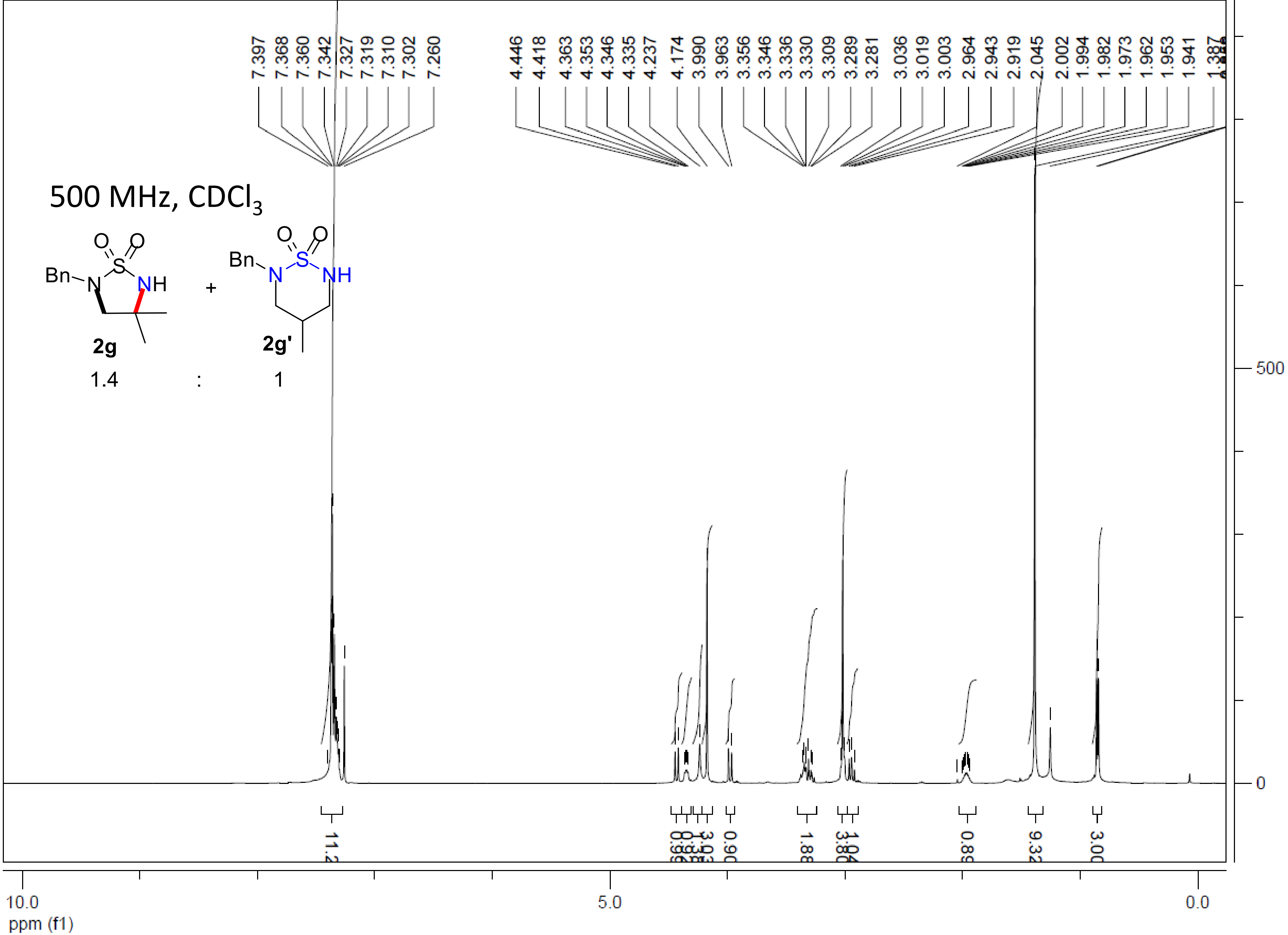
500 MHz, CDCl3
Bn
N
S
O
O
NH
2g
1.4
:
1
+
2g'
7.397
7.368
7.360
7.342
7.327
7.319
7.310
7.302
7.260
4.446
4.418
4.363
4.353
4.346
4.335
4.237
4.174
3.990
3.963
3.356
3.346
3.336
3.330
3.309
3.289
3.281
3.036
3.019
3.003
2.964
2.943
2.919
2.045
2.002
1.994
1.982
1.973
1.962
1.953
1.941
1.387
11.2
0.90
1.88
3.80
1.04
0.89
9.32
3.00
500
0
10.0
5.0
0.0
ppm (f1)

SII

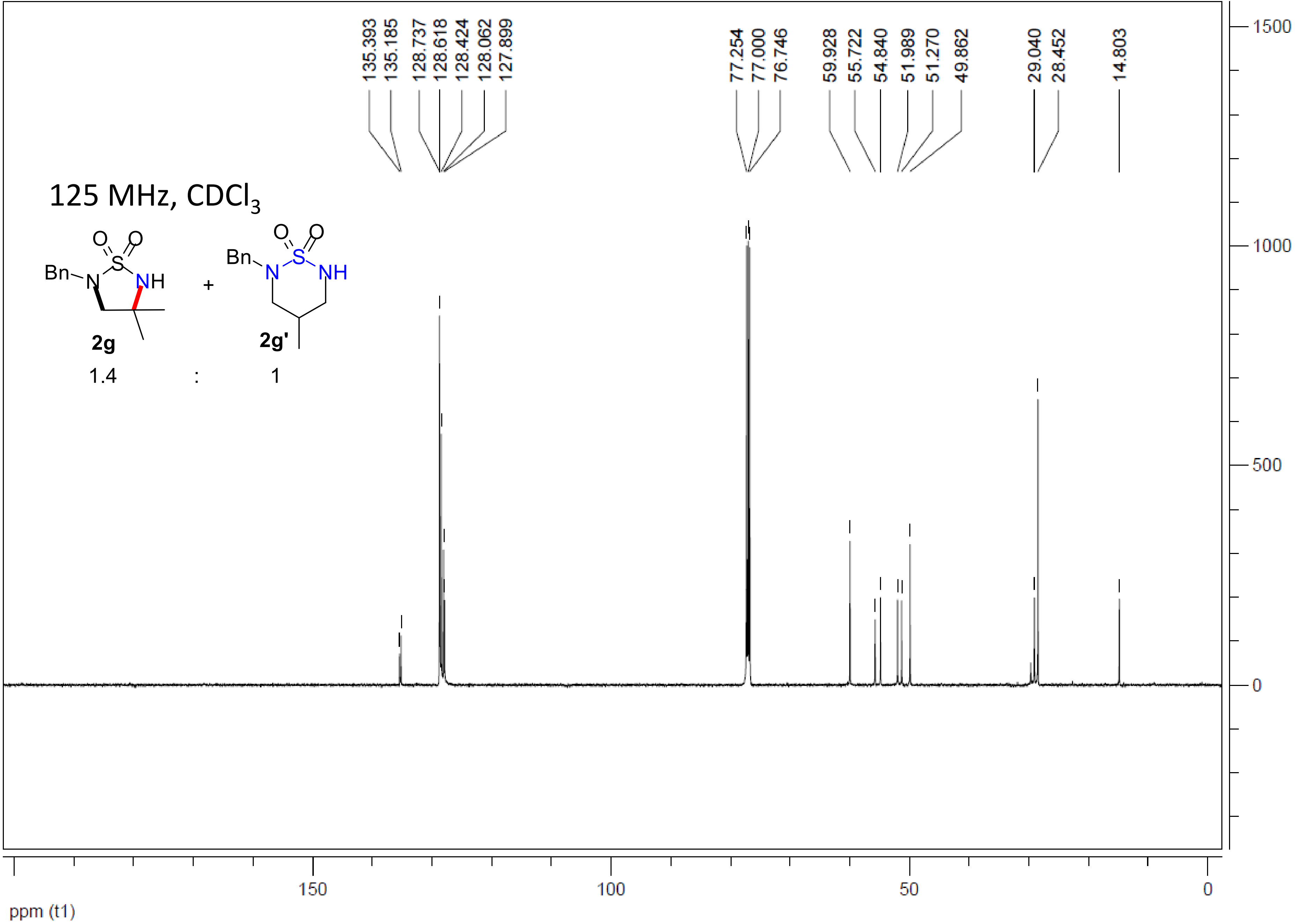
135.393
135.185
128.737
128.618
128.424
128.062
127.899
77.254
77.000
76.746
59.928
55.722
54.840
51.989
51.270
49.862
29.040
28.452
14.803
125 MHz, CDCl3
Bn
N
S
O
O
NH
2g
1.4
:
1
+
2g'
1500
1000
500
0
150
100
50
0
ppm (t1)

SII

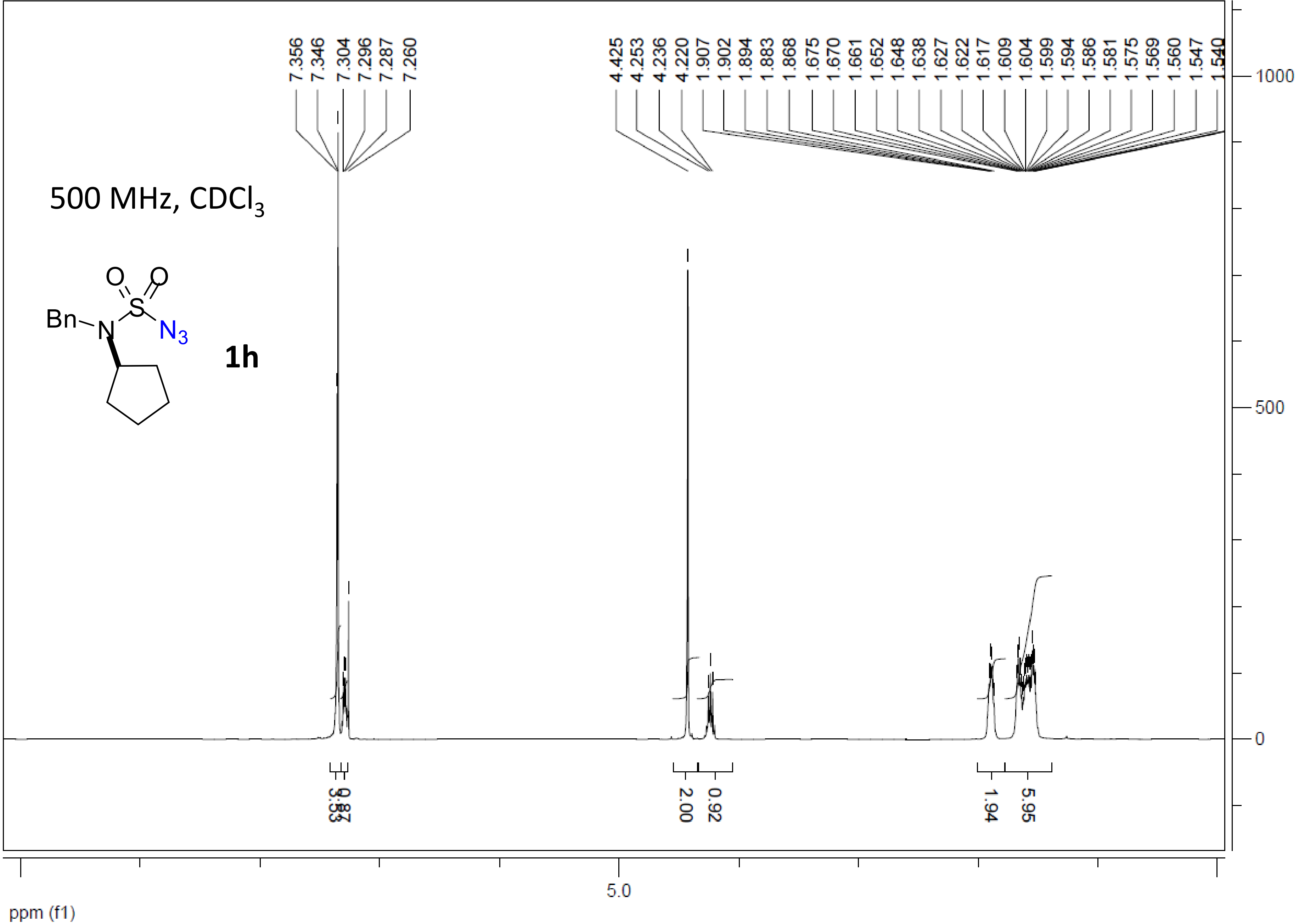

7.356
7.346
7.304
7.296
7.287
7.260
4.425
4.253
4.236
4.220
1.907
1.902
1.894
1.883
1.868
1.675
1.670
1.661
1.652
1.648
1.638
1.627
1.622
1.617
1.609
1.604
1.599
1.594
1.586
1.581
1.575
1.569
1.560
1.547
1.540
500 MHz, $CDCl_3$
O
O
S
Bn
N
N_3
1h
1000
500
0
2.00
0.92
1.94
5.95
5.0
ppm (f1)

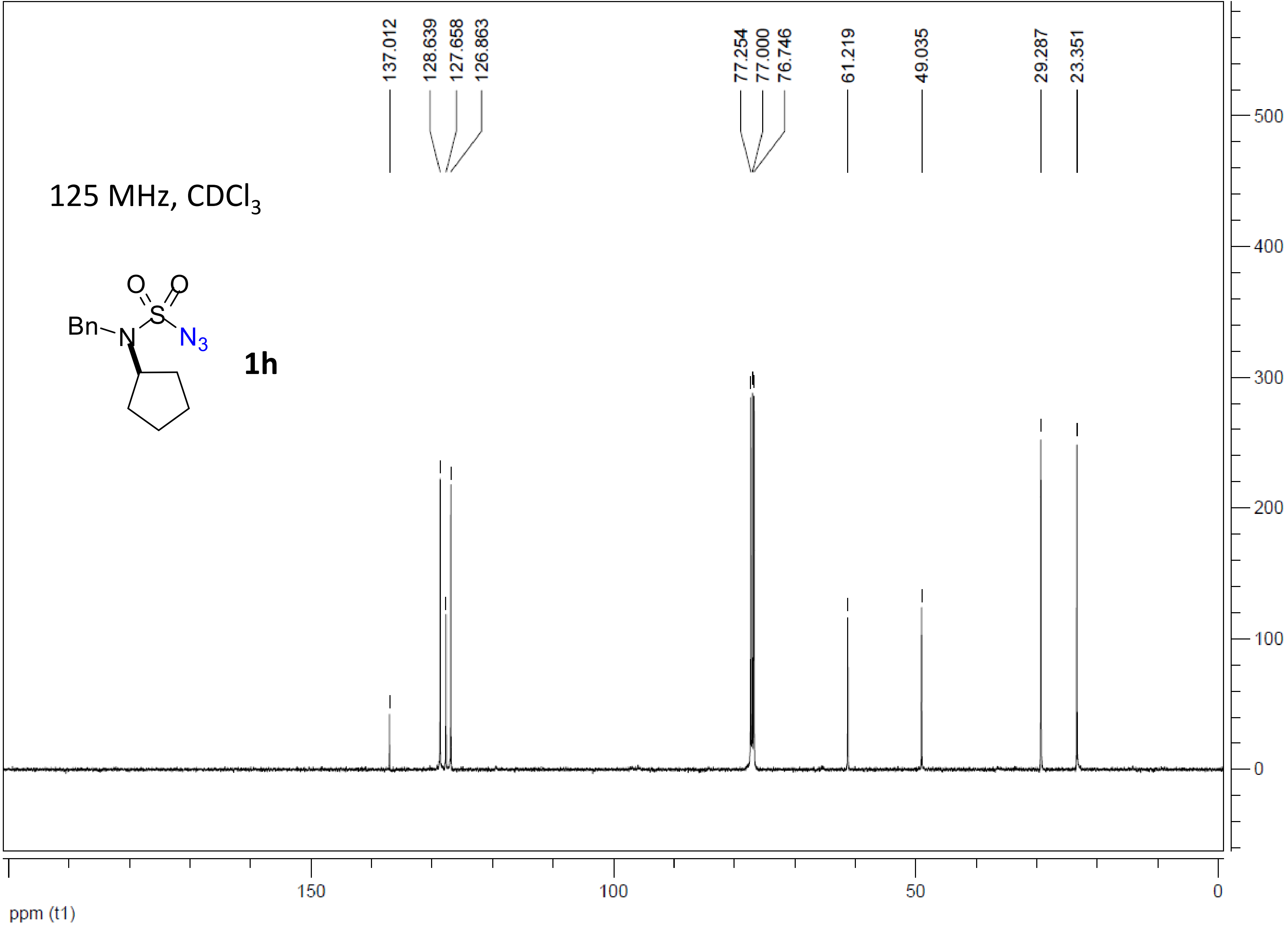
137.012
128.639
127.658
126.863
77.254
77.000
76.746
61.219
49.035
29.287
23.351
125 MHz, CDCl3
Bn
N
S
O
O
N3
1h
500
400
300
200
100
0
150
100
50
0
ppm (t1)

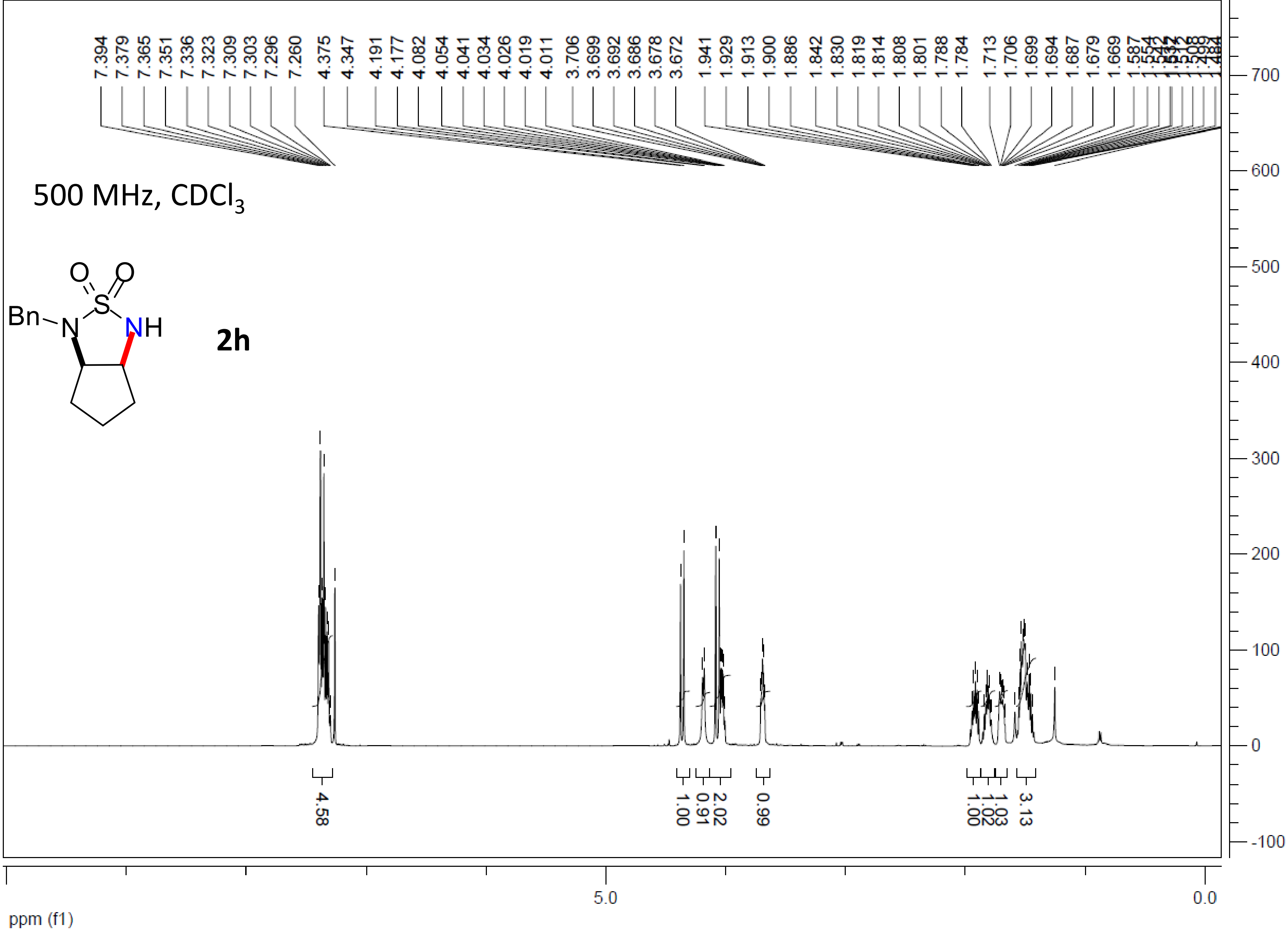
7.394 7.379 7.365 7.351 7.336 7.323 7.309 7.303 7.296 7.260 4.375 4.347 4.191 4.177 4.082 4.054 4.041 4.034 4.026 4.019 4.011 3.706 3.699 3.692 3.686 3.678 3.672 1.941 1.929 1.913 1.900 1.886 1.842 1.830 1.819 1.814 1.808 1.801 1.788 1.784 1.713 1.706 1.699 1.694 1.687 1.679 1.669 1.587 1.554
500 MHz, CDCl3
Bn
N
S
O
O
NH
2h
4.58
1.00
0.91
2.02
0.99
1.00
1.02
1.03
3.13
700
600
500
400
300
200
100
0
-100
5.0
0.0
ppm (f1)

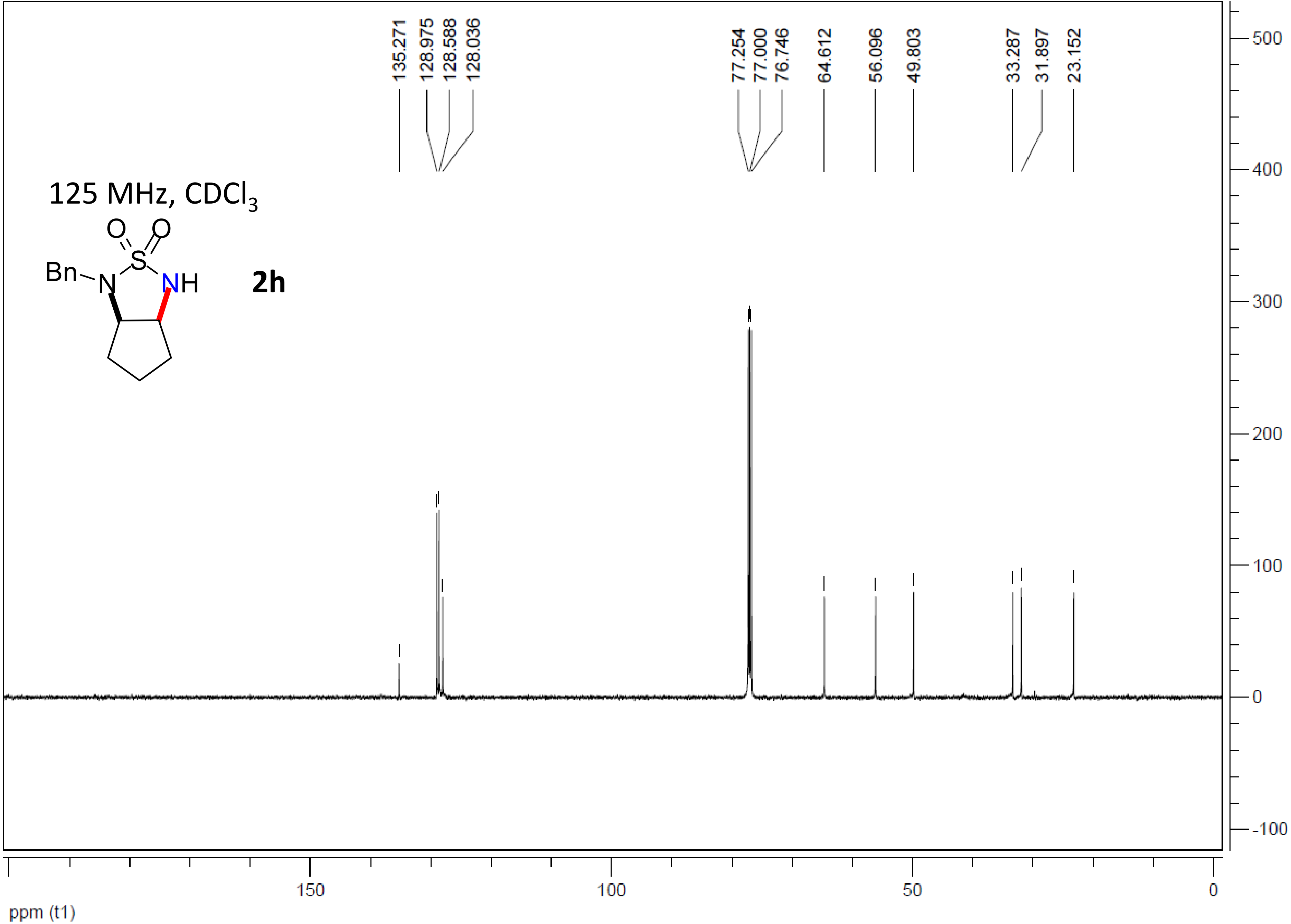
135.271
128.975
128.588
128.036
77.254
77.000
76.746
64.612
56.096
49.803
33.287
31.897
23.152
125 MHz, $CDCl_3$
Bn
N
S
O
O
NH
2h
500
400
300
200
100
0
-100
150
100
50
0
ppm (t1)

500 MHz, $CDCl_3$

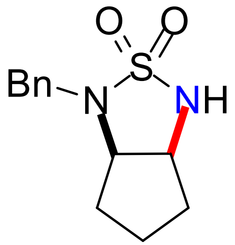

2h

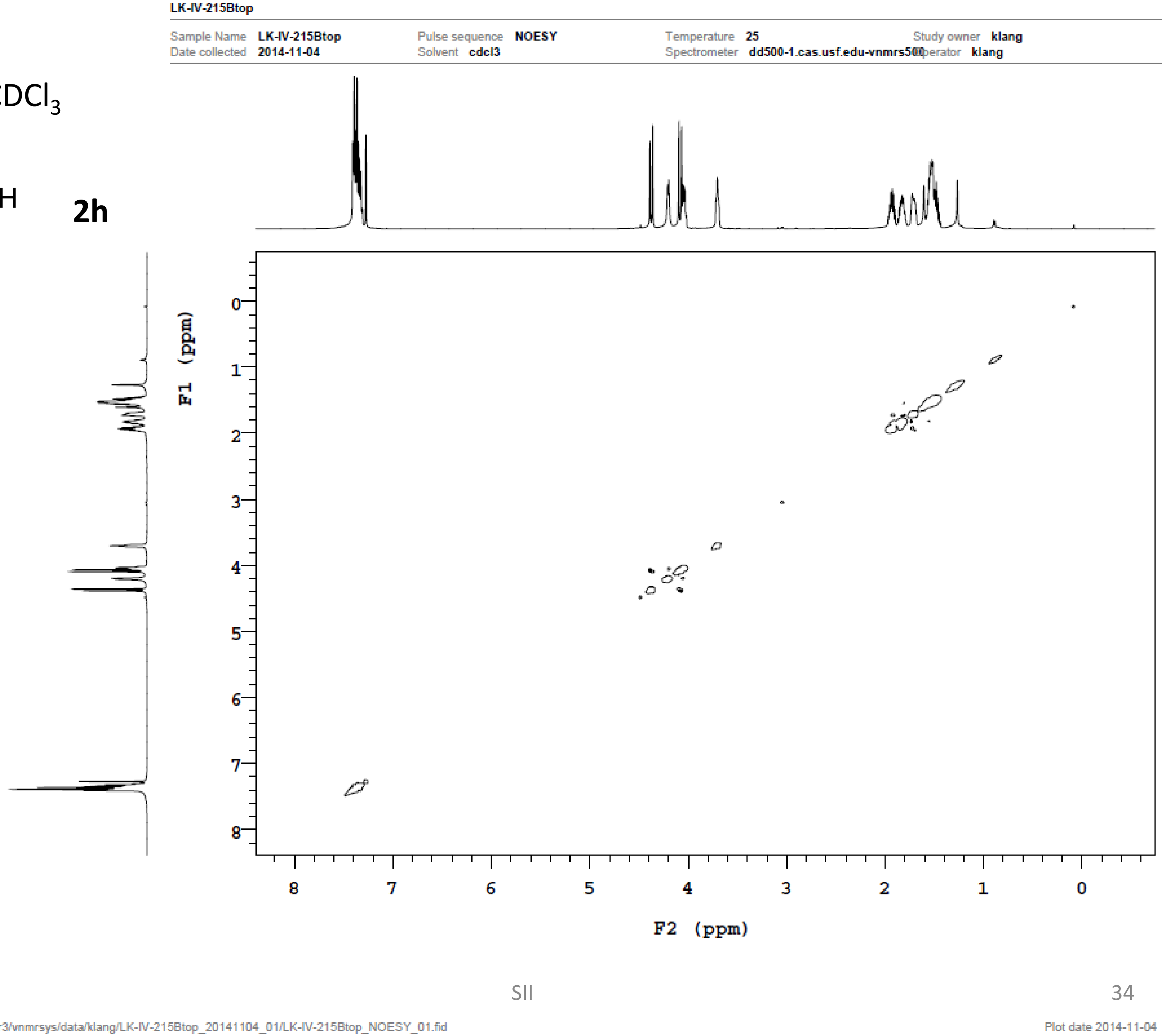

500 MHz, $CDCl_3$

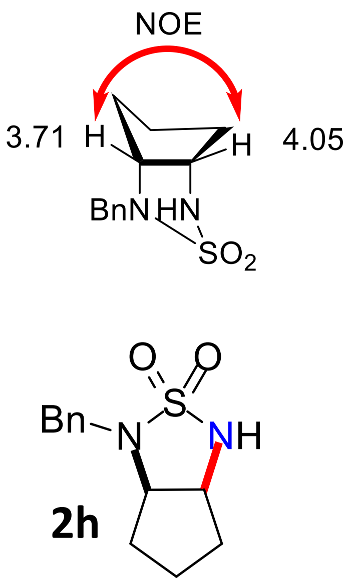

2h

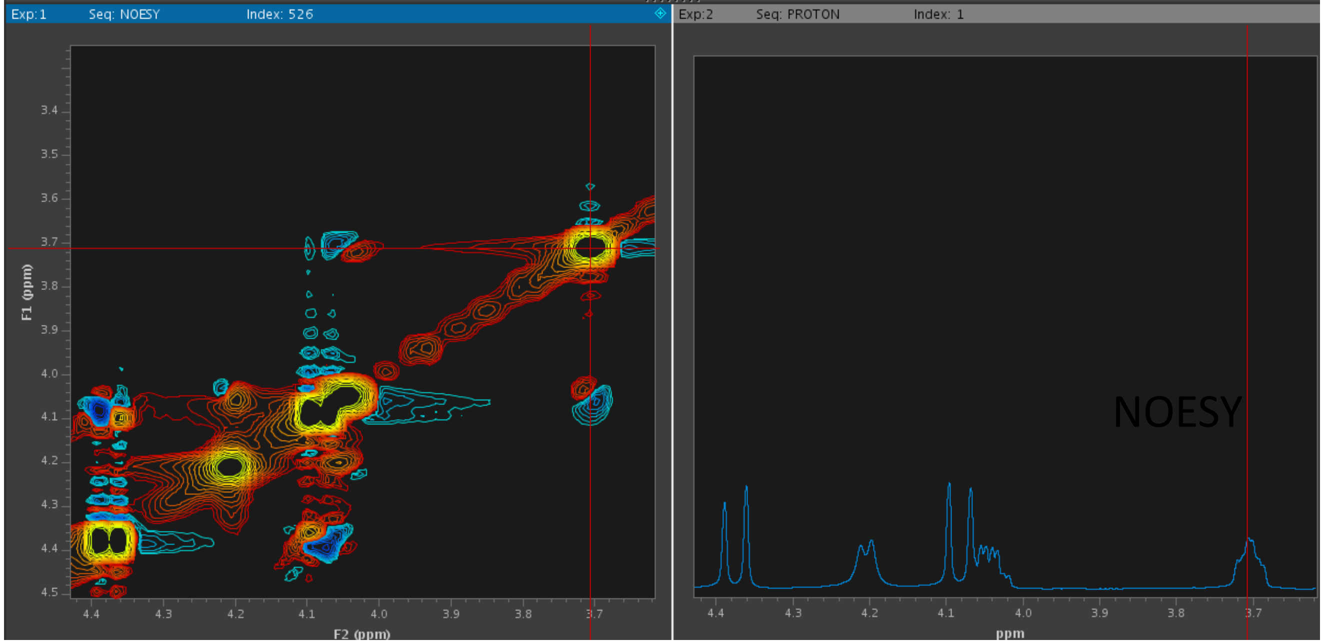

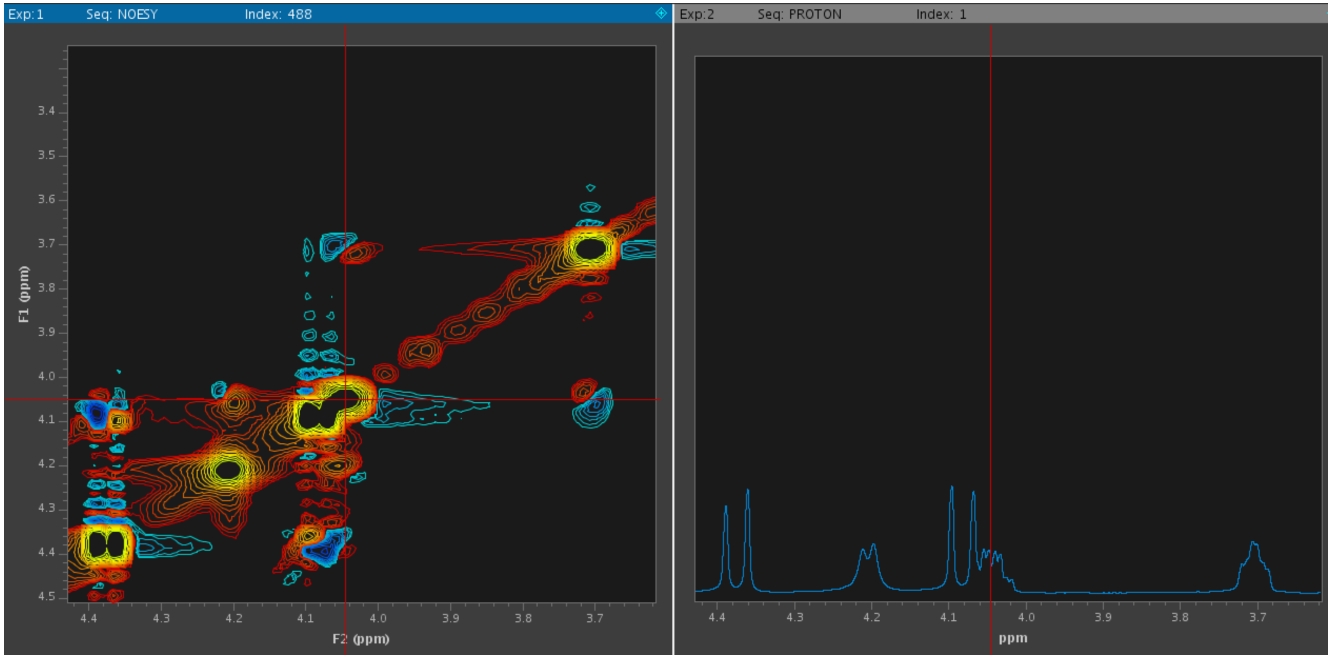

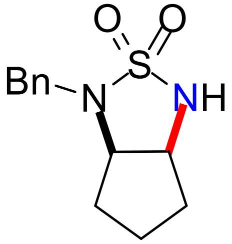
O
O
Bn
N
S
NH

2h

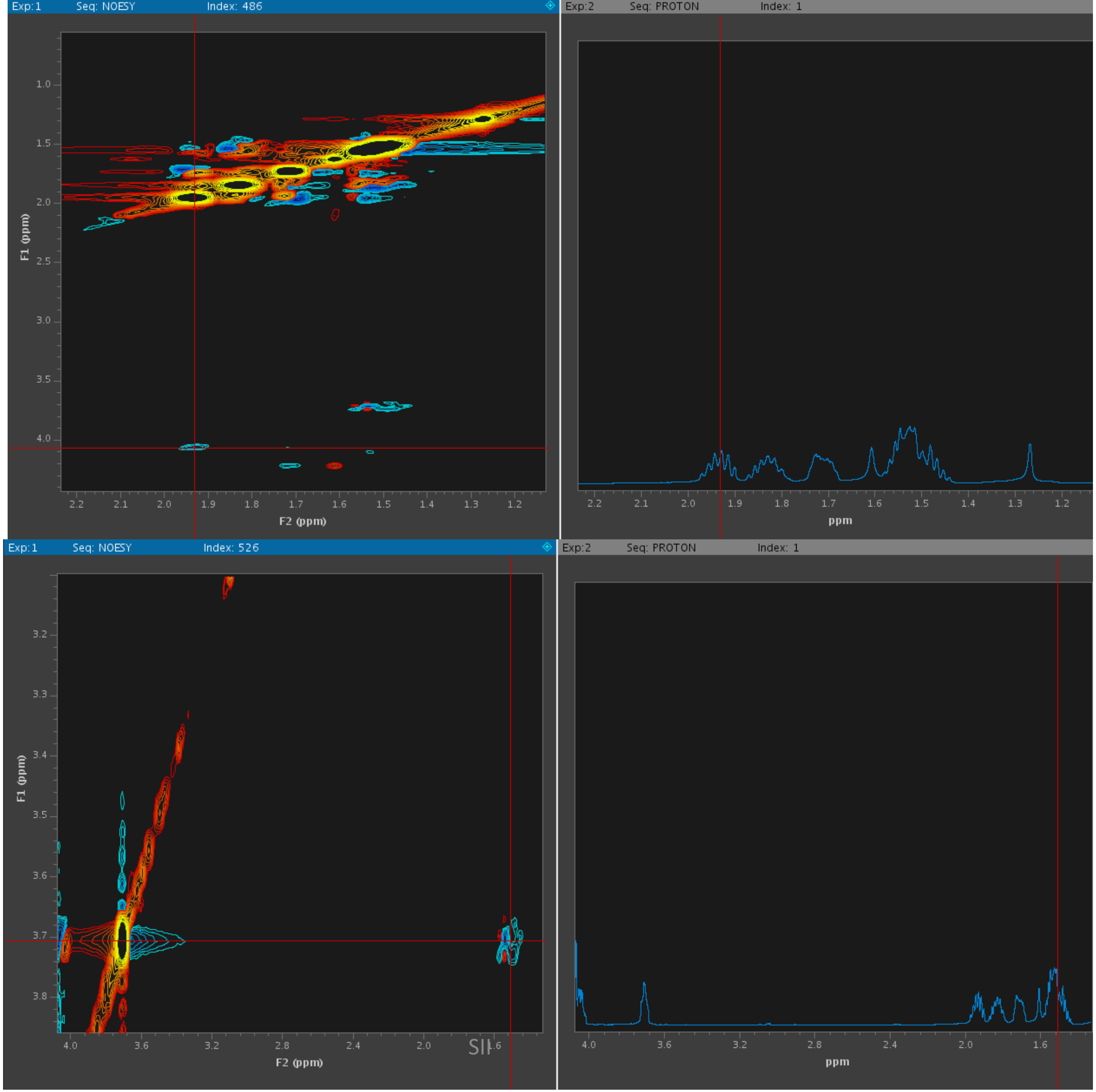
Exp: 1 Seq: NOESY Index: 486
Exp: 2 Seq: PROTON Index: 1
F1 (ppm)
F2 (ppm)
ppm
Exp: 1 Seq: NOESY Index: 526
Exp: 2 Seq: PROTON Index: 1
F1 (ppm)
F2 (ppm)
ppm

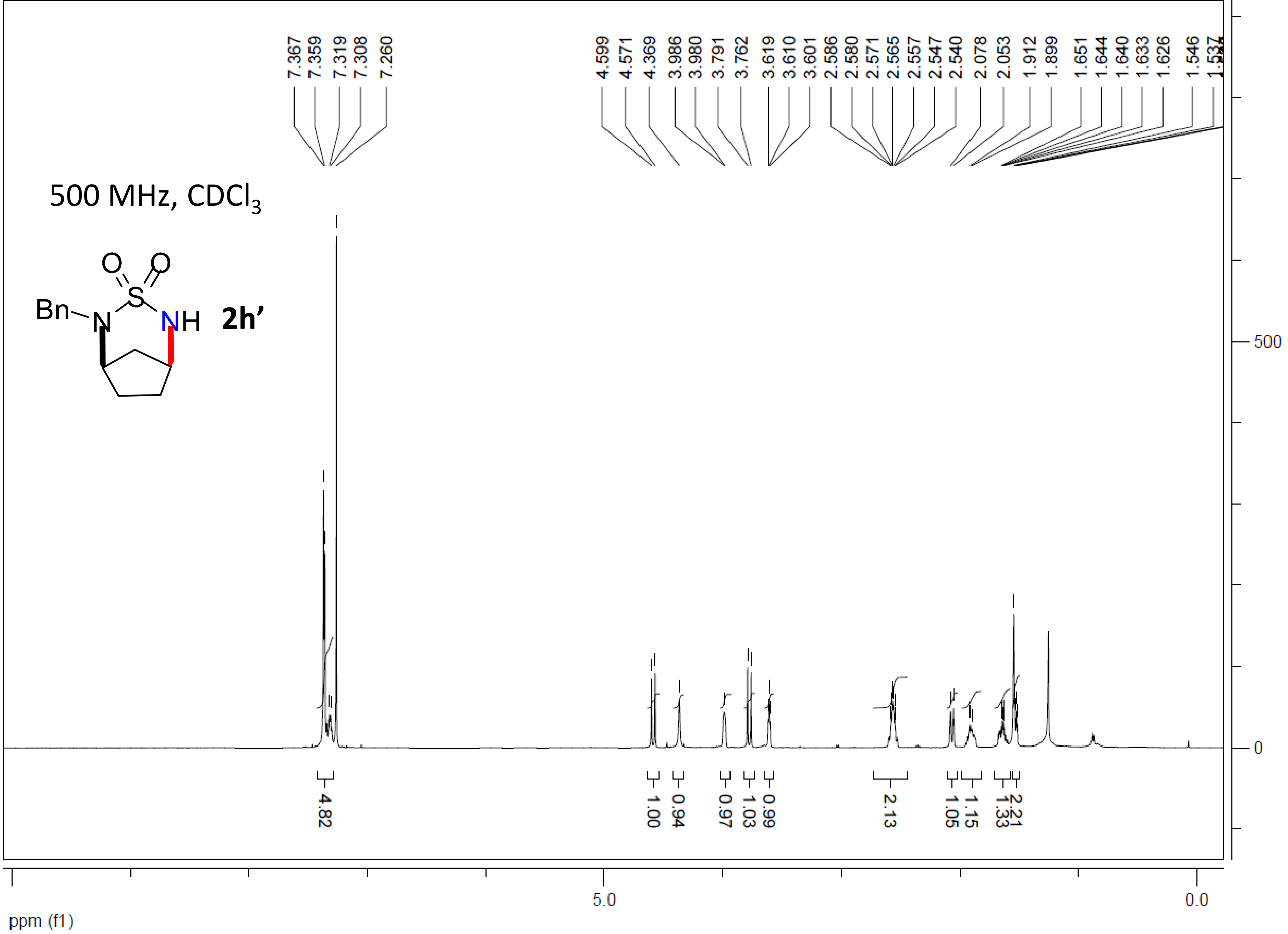
500 MHz, CDCl3
Bn
N
S
O
O
NH
2h'
7.367
7.359
7.319
7.308
7.260
4.599
4.571
4.369
3.986
3.980
3.791
3.762
3.619
3.610
3.601
2.586
2.580
2.571
2.565
2.557
2.547
2.540
2.078
2.053
1.912
1.899
1.651
1.644
1.640
1.633
1.626
1.546
1.537
4.82
1.00
0.94
0.97
1.03
0.99
2.13
1.05
1.15
1.33
2.21
500
0
5.0
0.0
ppm (f1)

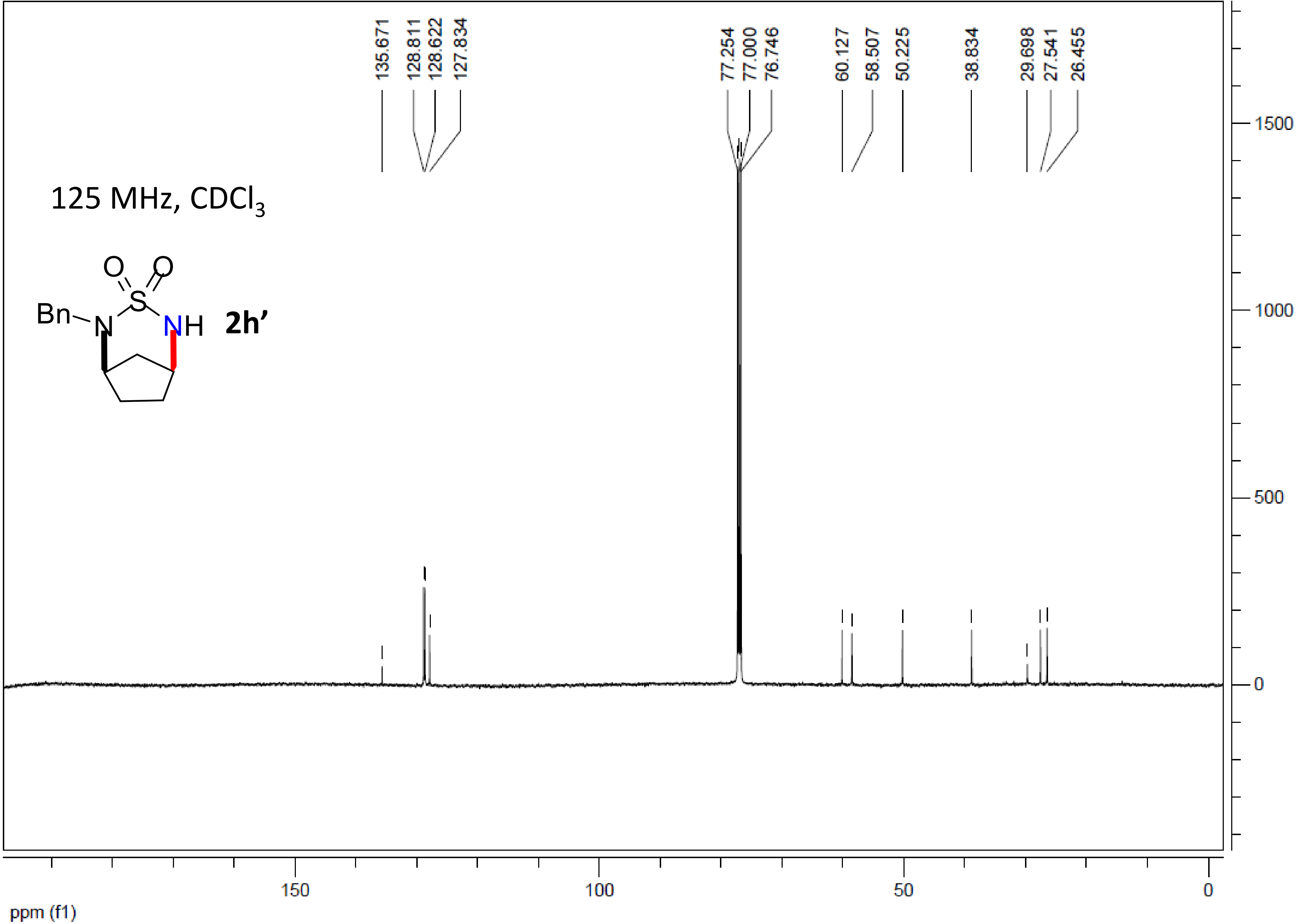
135.671
128.811
128.622
127.834
77.254
77.000
76.746
60.127
58.507
50.225
38.834
29.698
27.541
26.455
125 MHz, CDCl3
Bn
N
S
O
O
NH
2h’
1500
1000
500
0
150
100
50
0
ppm (f1)

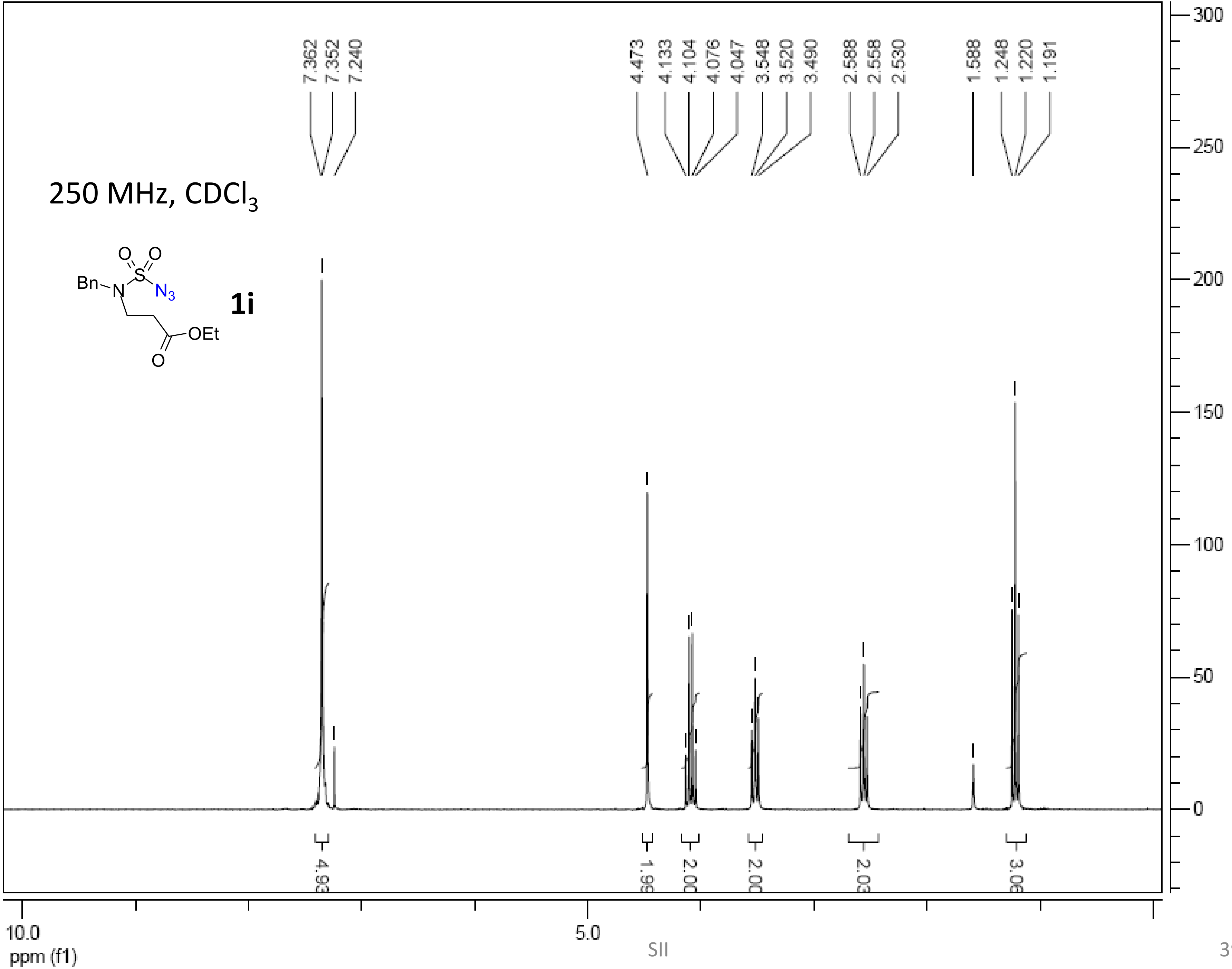
250 MHz, $CDCl_3$
Bn
N
S
O O
N_3
OEt
O
1i
7.362 7.352 7.240
4.473 4.133 4.104 4.076 4.047 3.548 3.520 3.490
2.588 2.558 2.530
1.588 1.248 1.220 1.191
4.93 1.99 2.00 2.00 2.03 3.06
300 250 200 150 100 50 0
10.0
5.0
ppm (f1)

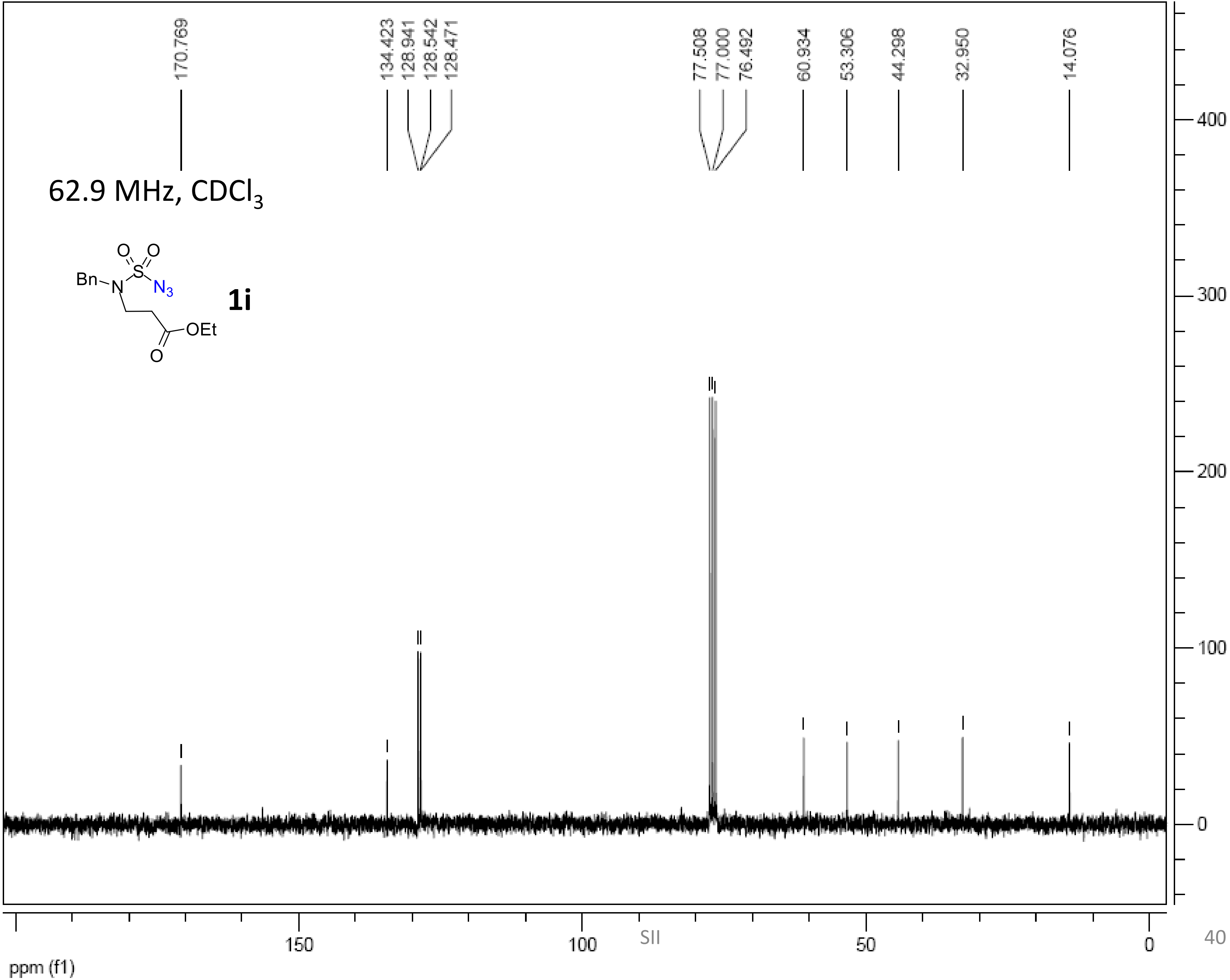
170.769
134.423
128.941
128.542
128.471
77.508
77.000
76.492
60.934
53.306
44.298
32.950
14.076
62.9 MHz, $CDCl_3$
Bn
N
S
O
O
N_3
OEt
O
1i
400
300
200
100
0
150
100
50
0
ppm (f1)

SII

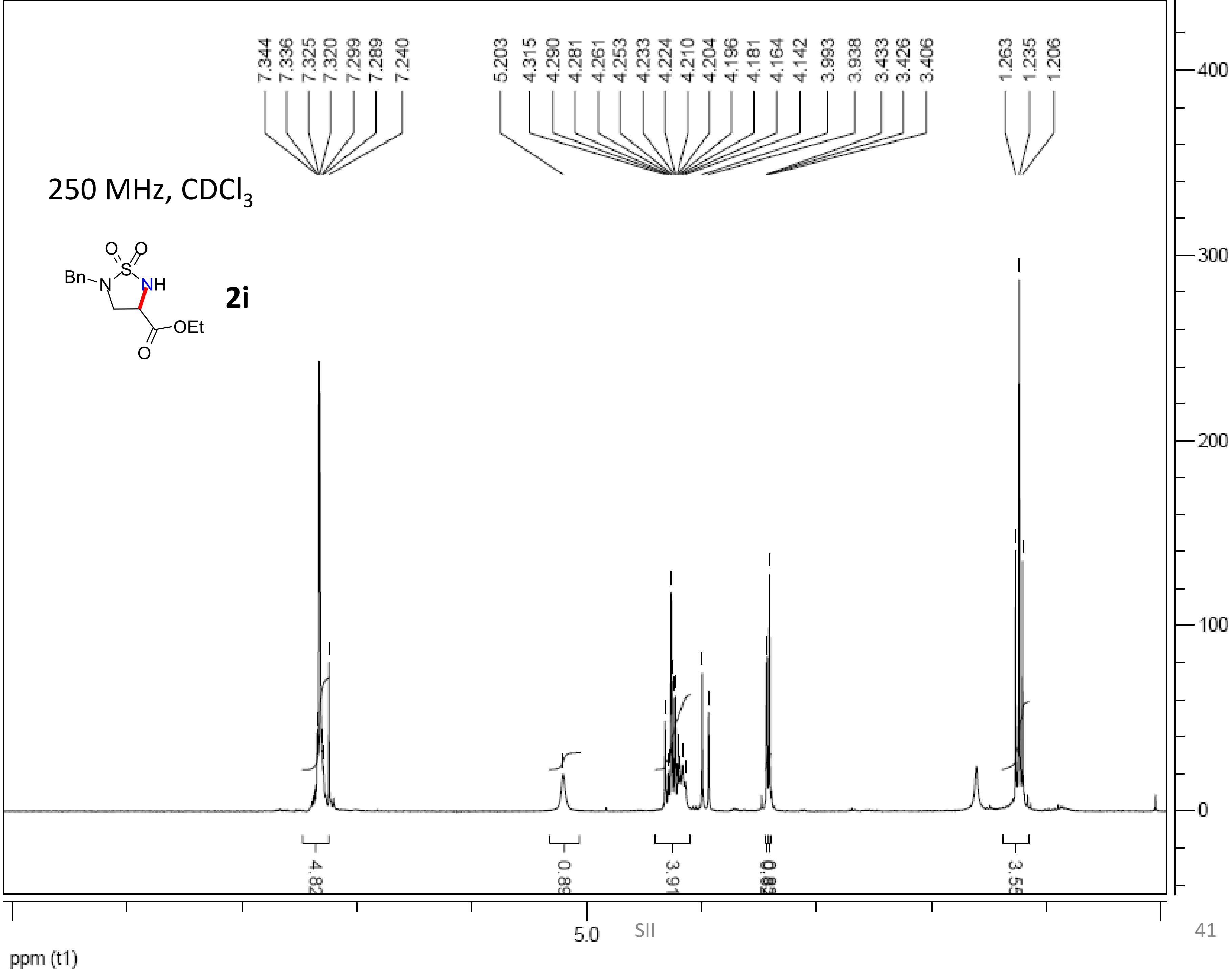

7.344 7.336 7.325 7.320 7.299 7.289 7.240
5.203 4.315 4.290 4.281 4.261 4.253 4.233 4.224 4.210 4.204 4.196 4.181 4.164 4.142 3.993 3.938 3.433 3.426 3.406
1.263 1.235 1.206
250 MHz, CDCl3
Bn
N
S
O
O
NH
OEt
O
2i
400
300
200
100
0
4.82
0.89
3.91
3.55
5.0
ppm (t1)

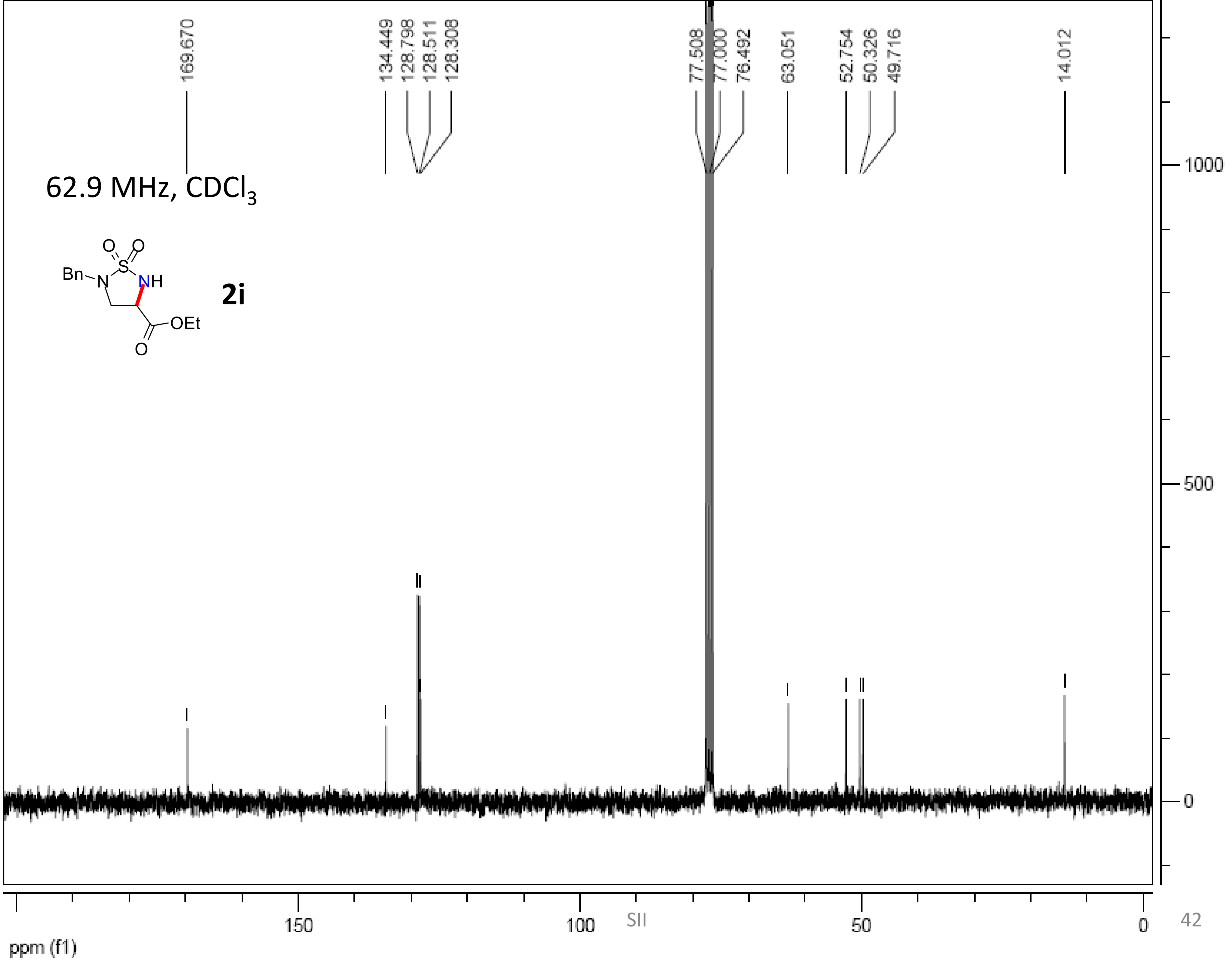
169.670
134.449
128.798
128.511
128.308
77.508
77.000
76.492
63.051
52.754
50.326
49.716
14.012
62.9 MHz, CDCl3
Bn
N
S
O
O
NH
OEt
O
2i
1000
500
0
150
100
50
0
ppm (f1)

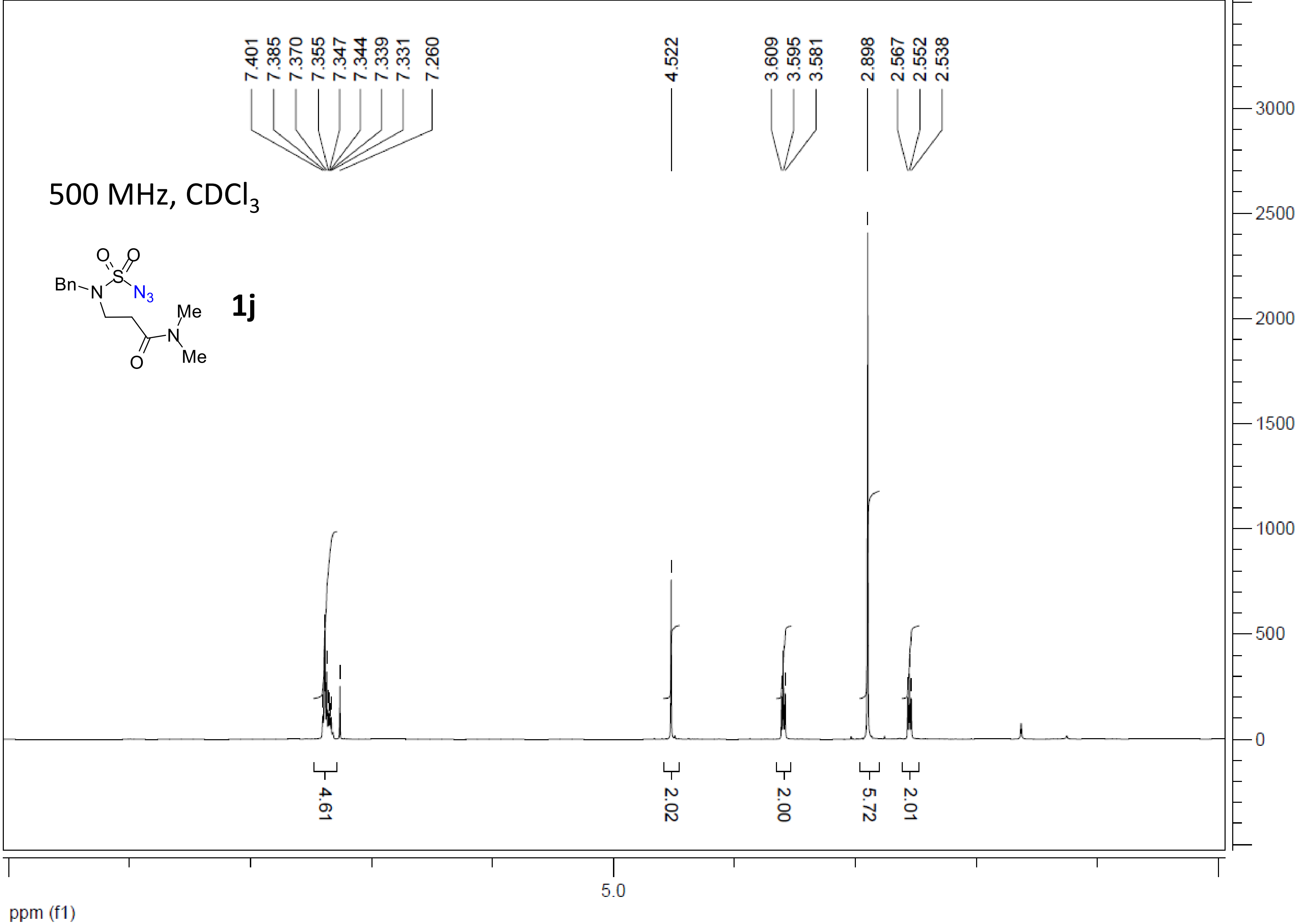

7.401
7.385
7.370
7.355
7.347
7.344
7.339
7.331
7.260
4.522
3.609
3.595
3.581
2.898
2.567
2.552
2.538
500 MHz, $CDCl_3$
Bn
N
S
O
O
N_3
Me
N
Me
O
1j
3000
2500
2000
1500
1000
500
0
4.61
2.02
2.00
5.72
2.01
5.0
ppm (f1)

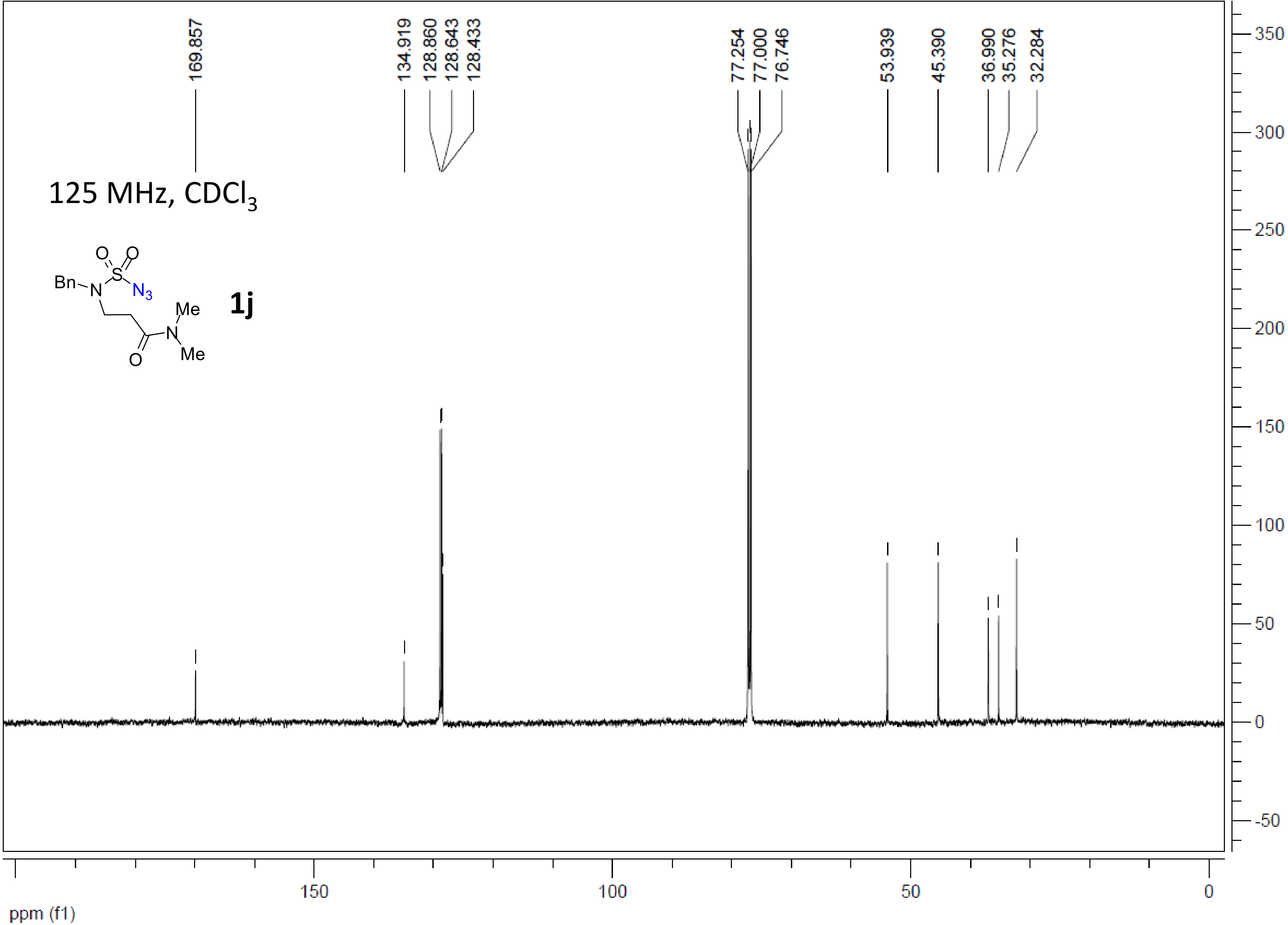
169.857
134.919
128.860
128.643
128.433
77.254
77.000
76.746
53.939
45.390
36.990
35.276
32.284
125 MHz, $CDCl_3$
Bn
N
S
O
O
N_3
Me
N
Me
O
1j
ppm (f1)

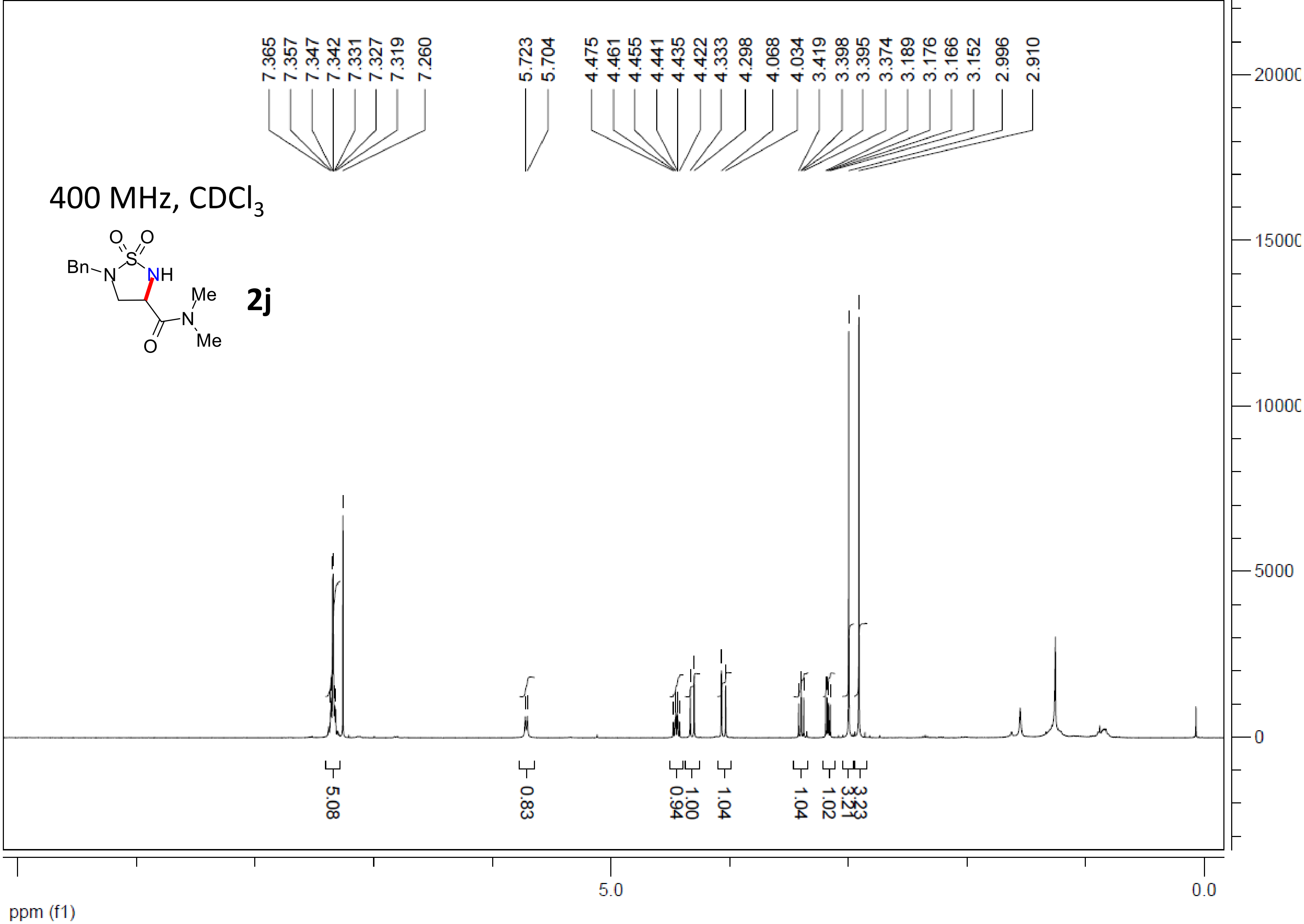
7.365
7.357
7.347
7.342
7.331
7.327
7.319
7.260
5.723
5.704
4.475
4.461
4.455
4.441
4.435
4.422
4.333
4.298
4.068
4.034
3.419
3.398
3.395
3.374
3.189
3.176
3.166
3.152
2.996
2.910
400 MHz, $CDCl_3$
5.08
0.83
0.94
1.00
1.04
1.04
1.02
3.21
3.23
5.0
0.0
ppm (f1)
20000
15000
10000
5000
0
Bn
N
S
O
O
NH
Me
N
Me
O

2j

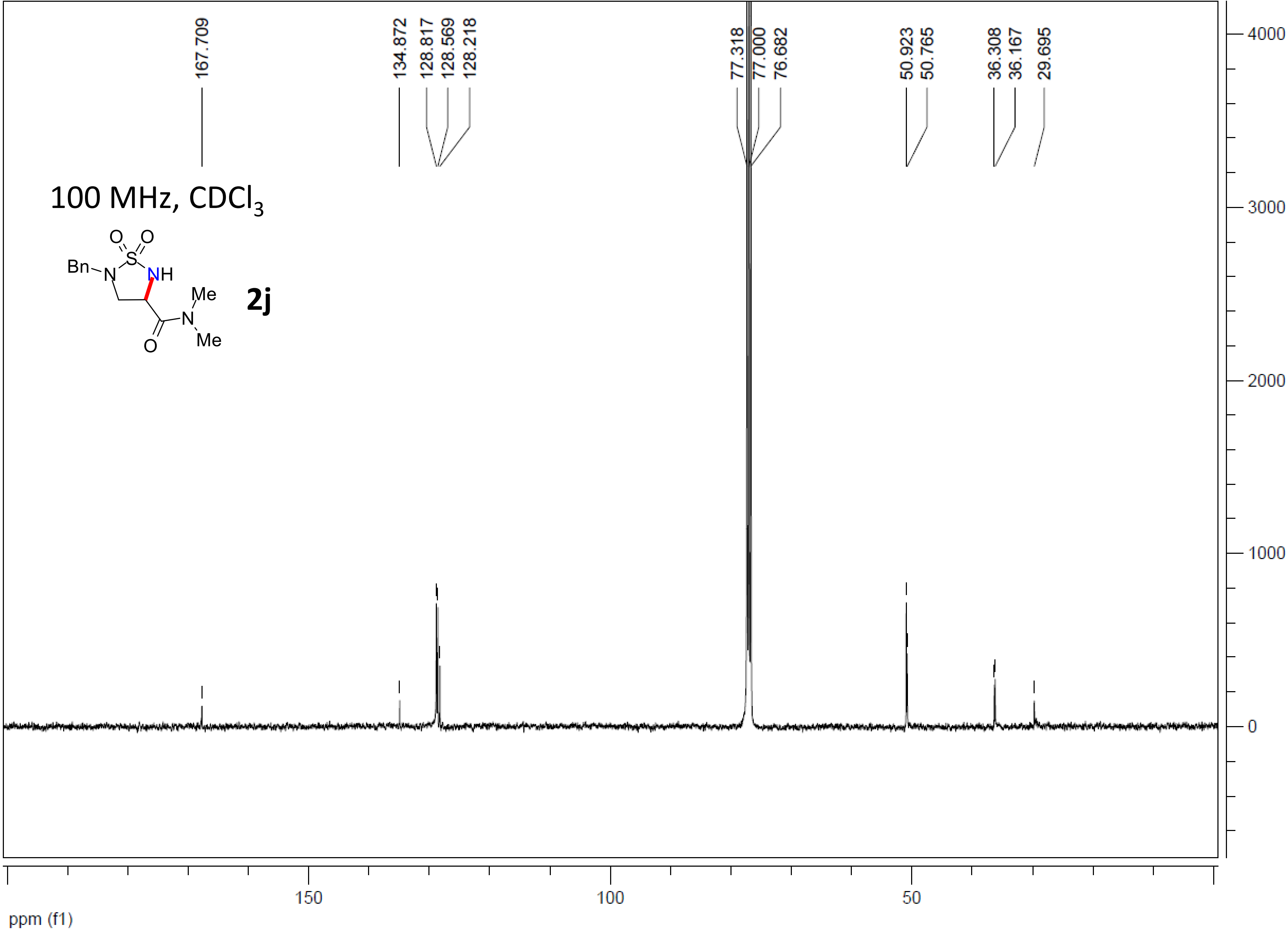
167.709
134.872
128.817
128.569
128.218
77.318
77.000
76.682
50.923
50.765
36.308
36.167
29.695
100 MHz, $CDCl_3$
O O
S
Bn
N
NH
Me
N
Me
O
2j
4000
3000
2000
1000
0
150
100
50
ppm (f1)

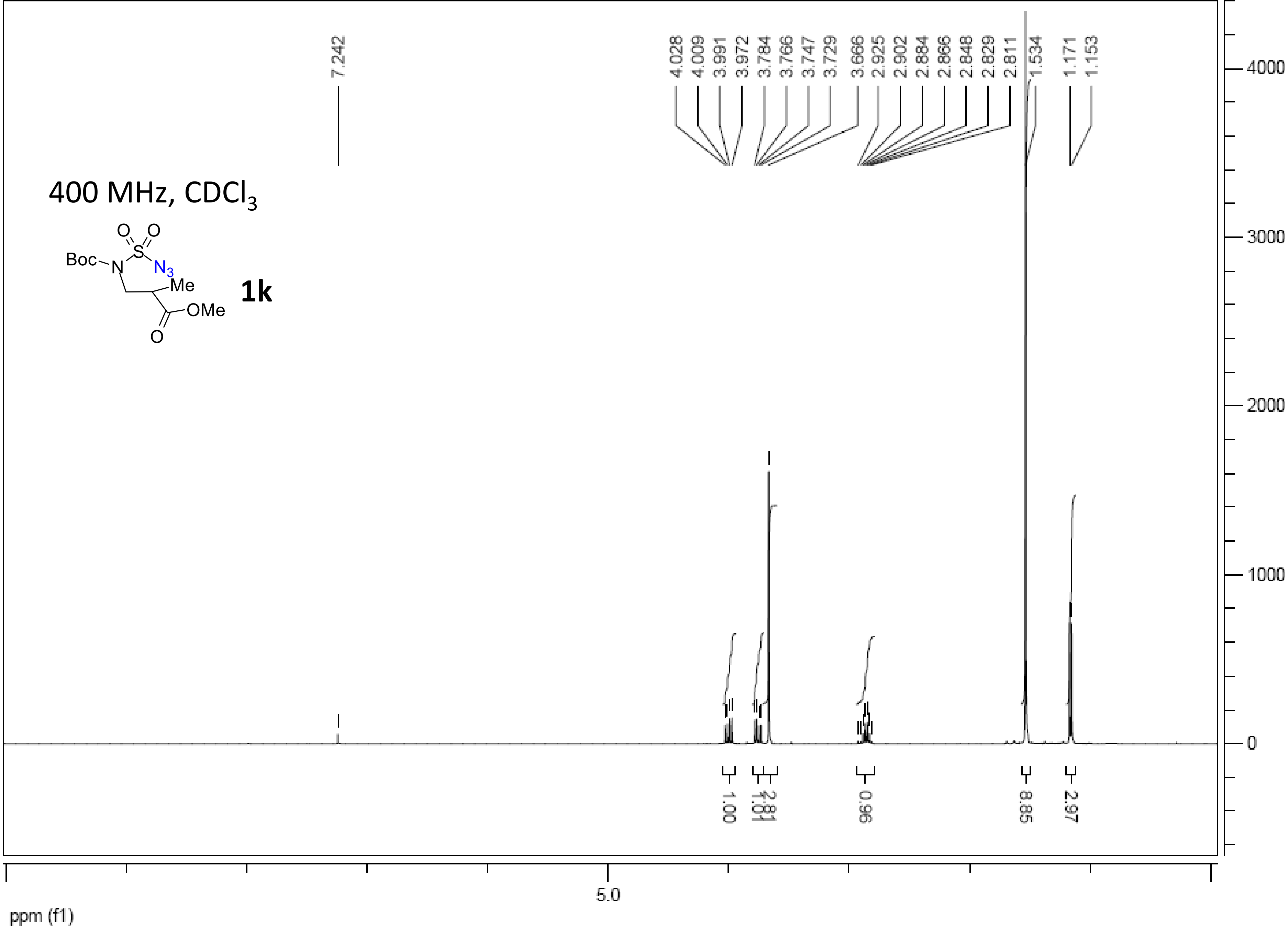

400 MHz, CDCl3
Boc
N
S
O
O
N3
Me
OMe
O
1k
7.242
4.028
4.009
3.991
3.972
3.784
3.766
3.747
3.729
3.666
2.925
2.902
2.884
2.866
2.848
2.829
2.811
1.534
1.171
1.153
1.00
1.01
2.81
0.96
8.85
2.97
4000
3000
2000
1000
0
5.0
ppm (f1)

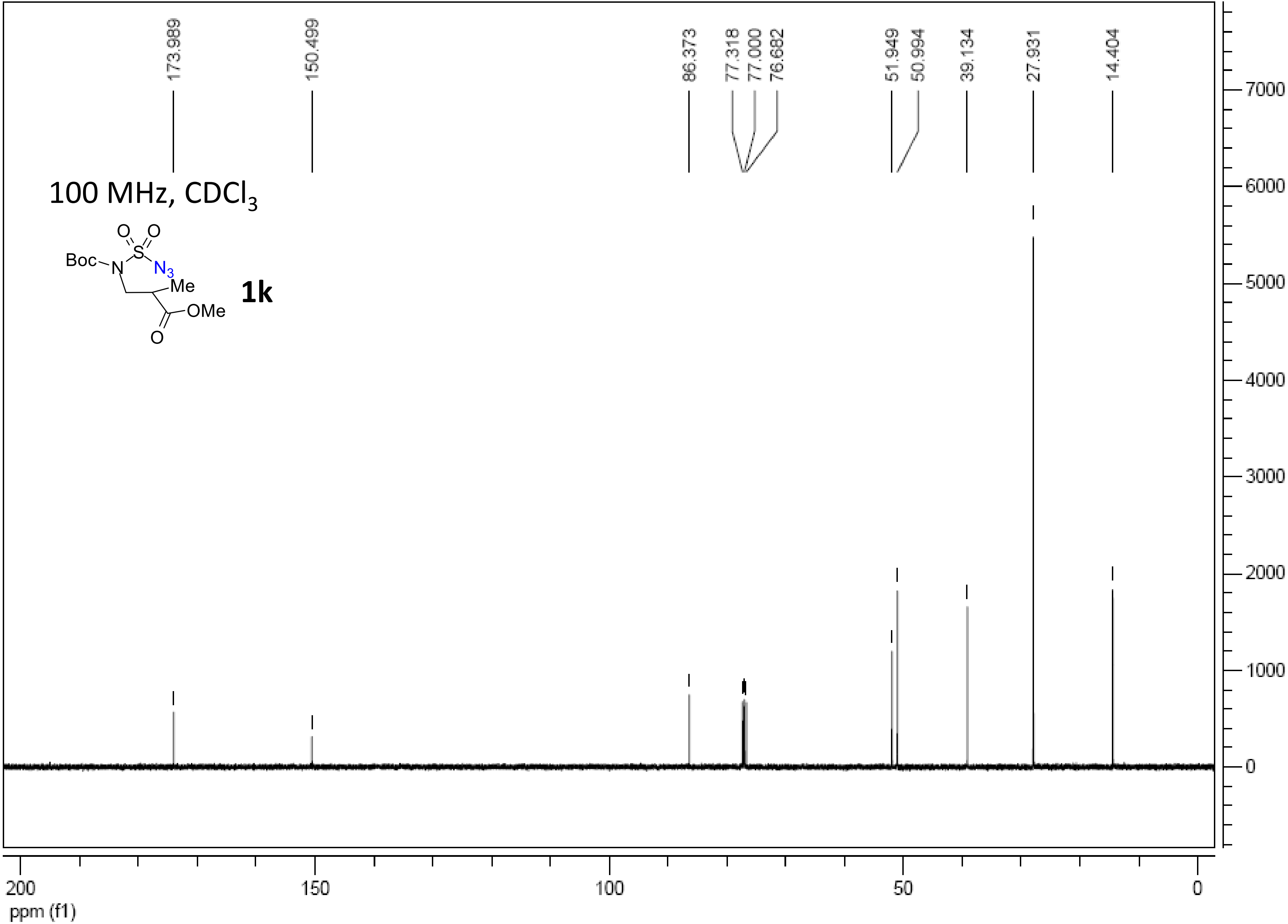

173.989
150.499
86.373
77.318
77.000
76.682
51.949
50.994
39.134
27.931
14.404
100 MHz, CDCl3
O
O
Boc
N
S
N3
Me
OMe
O
1k
7000
6000
5000
4000
3000
2000
1000
0
200
150
100
50
0
ppm (f1)

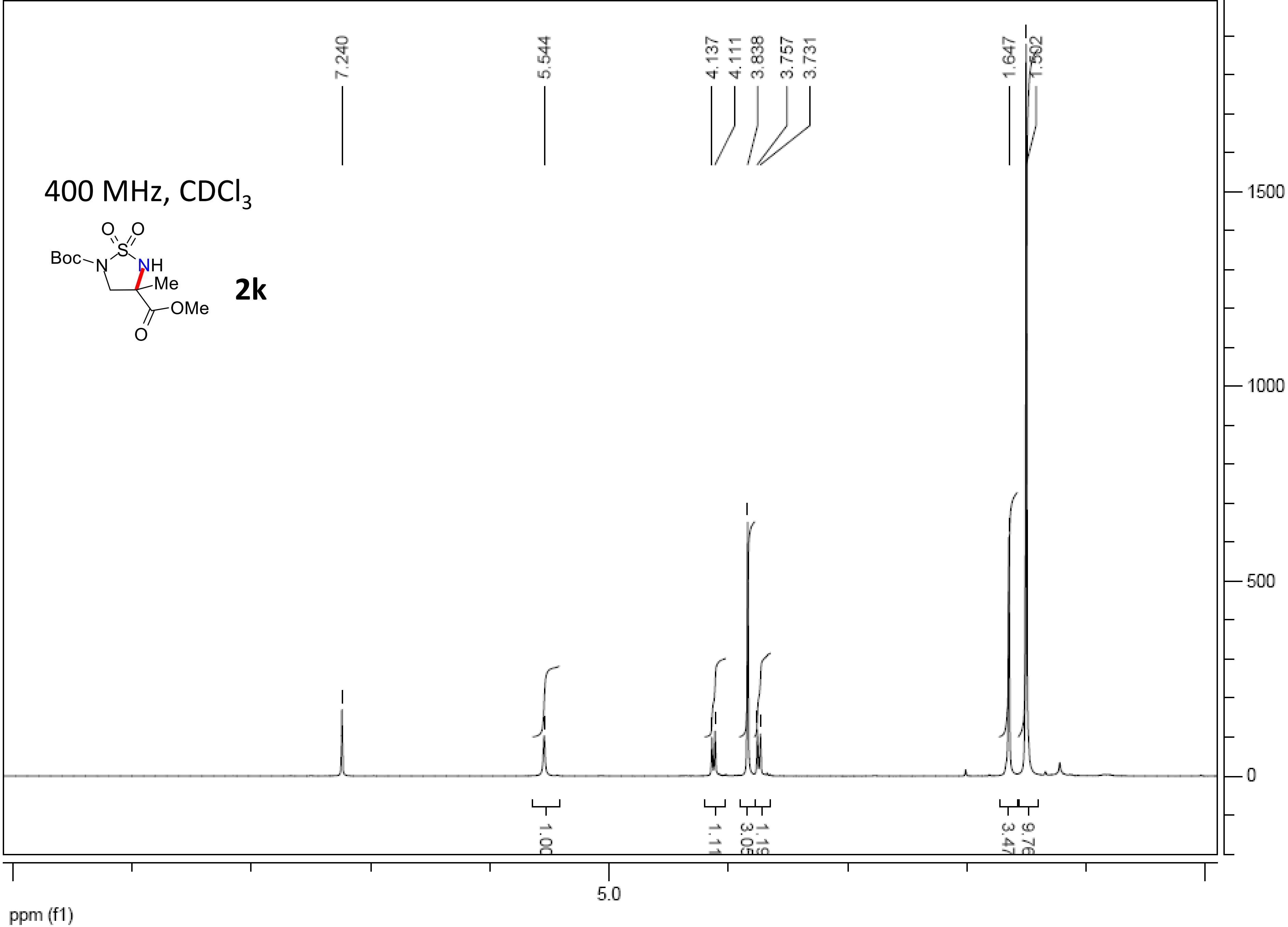

7.240
5.544
4.137
4.111
3.838
3.757
3.731
1.647
1.502
400 MHz, CDCl3
Boc
N
S
O
O
NH
Me
OMe
O
2k
1500
1000
500
0
1.00
1.11
3.05
1.19
3.47
9.76
5.0
ppm (f1)

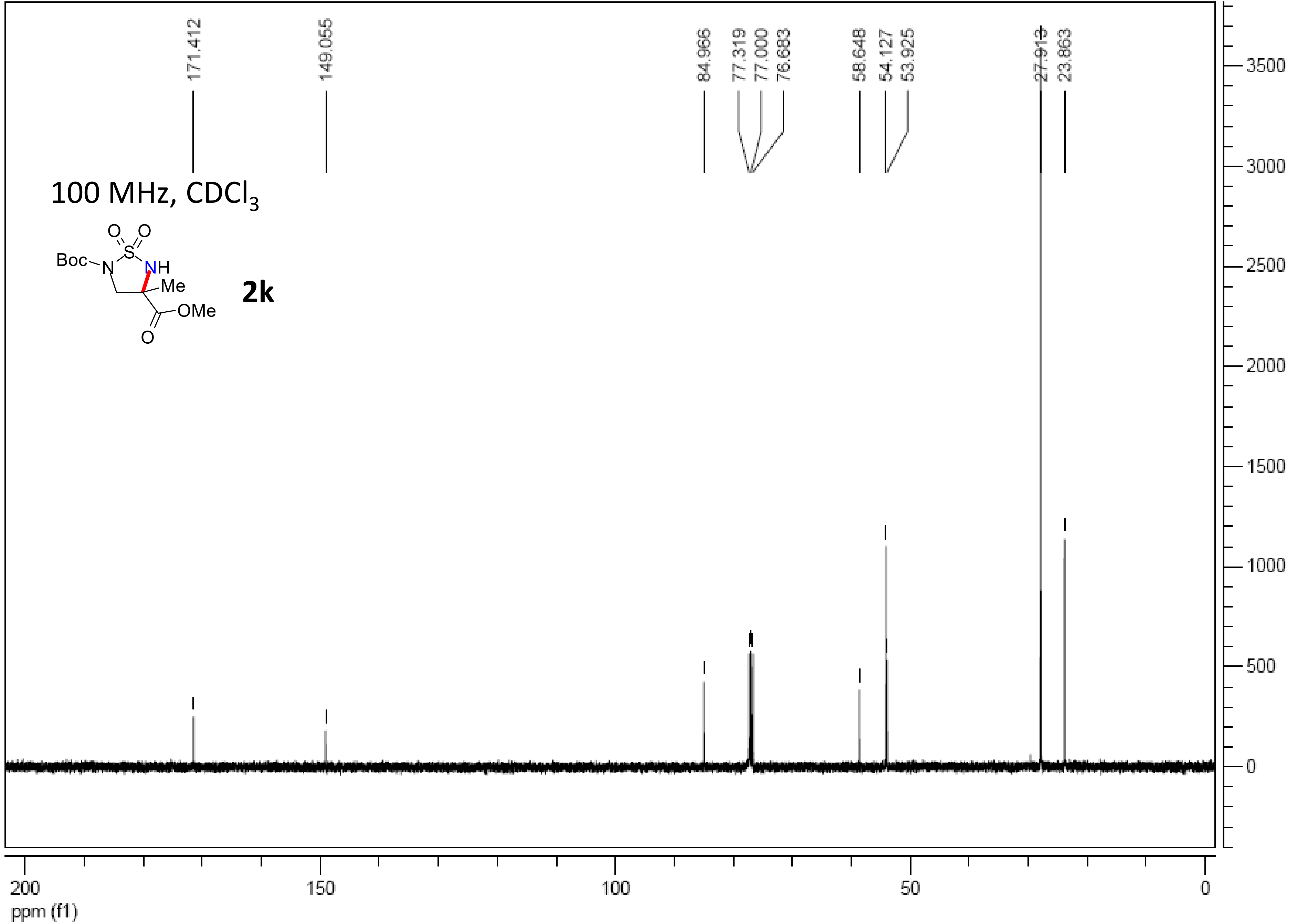
171.412
149.055
84.966
77.319
77.000
76.683
58.648
54.127
53.925
27.913
23.863
100 MHz, $CDCl_3$
O O
Boc
N
S
NH
Me
OMe
O
2k
3500
3000
2500
2000
1500
1000
500
0
200
150
100
50
0
ppm (f1)

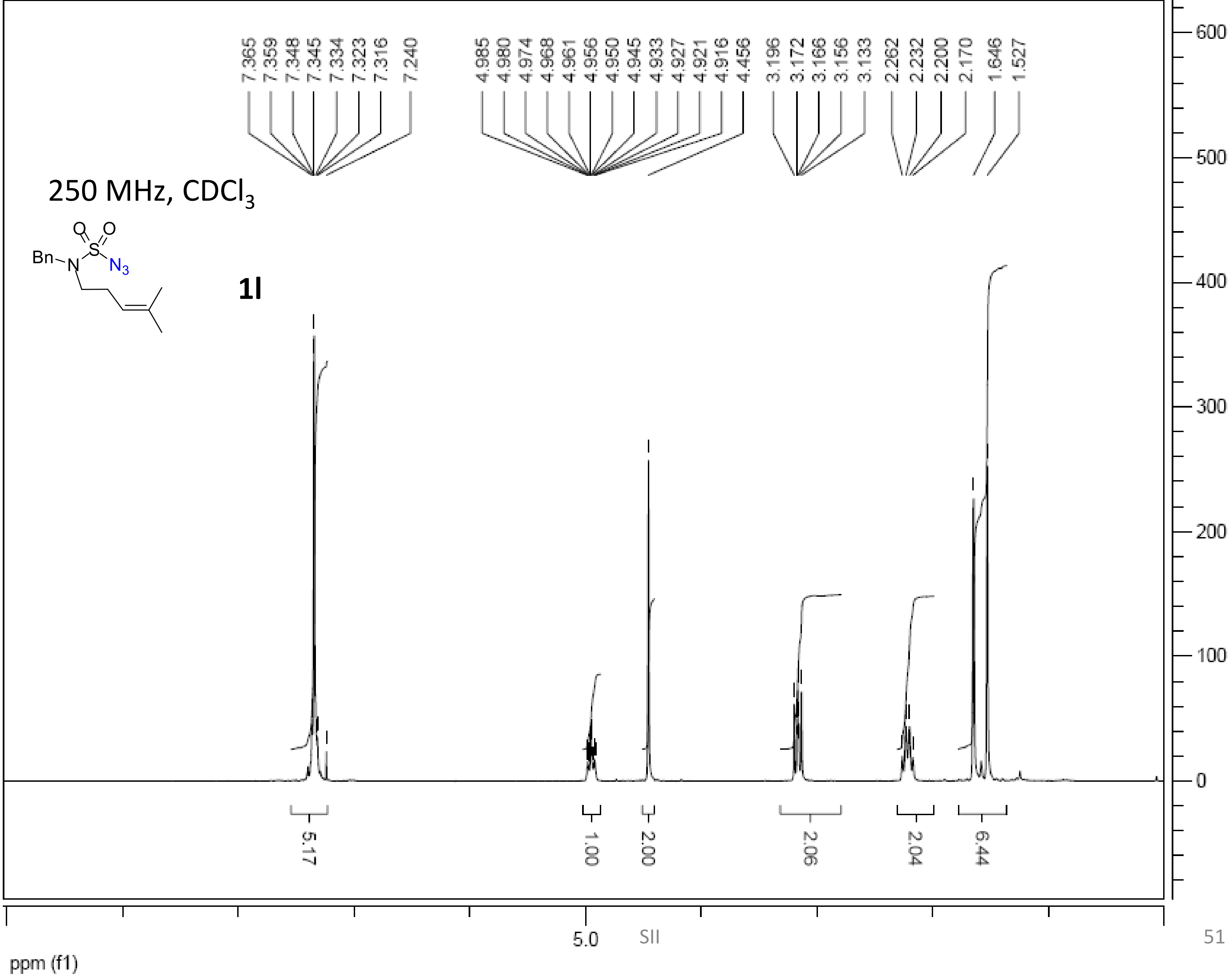
250 MHz, CDCl3
1l
Bn
N
S
O
O
N3
7.365 7.359 7.348 7.345 7.334 7.323 7.316 7.240
4.985 4.980 4.974 4.968 4.961 4.956 4.950 4.945 4.933 4.927 4.921 4.916 4.456
3.196 3.172 3.166 3.156 3.133
2.262 2.232 2.200 2.170 1.646 1.527
5.17
1.00
2.00
2.06
2.04
6.44
600
500
400
300
200
100
0
5.0
ppm (f1)

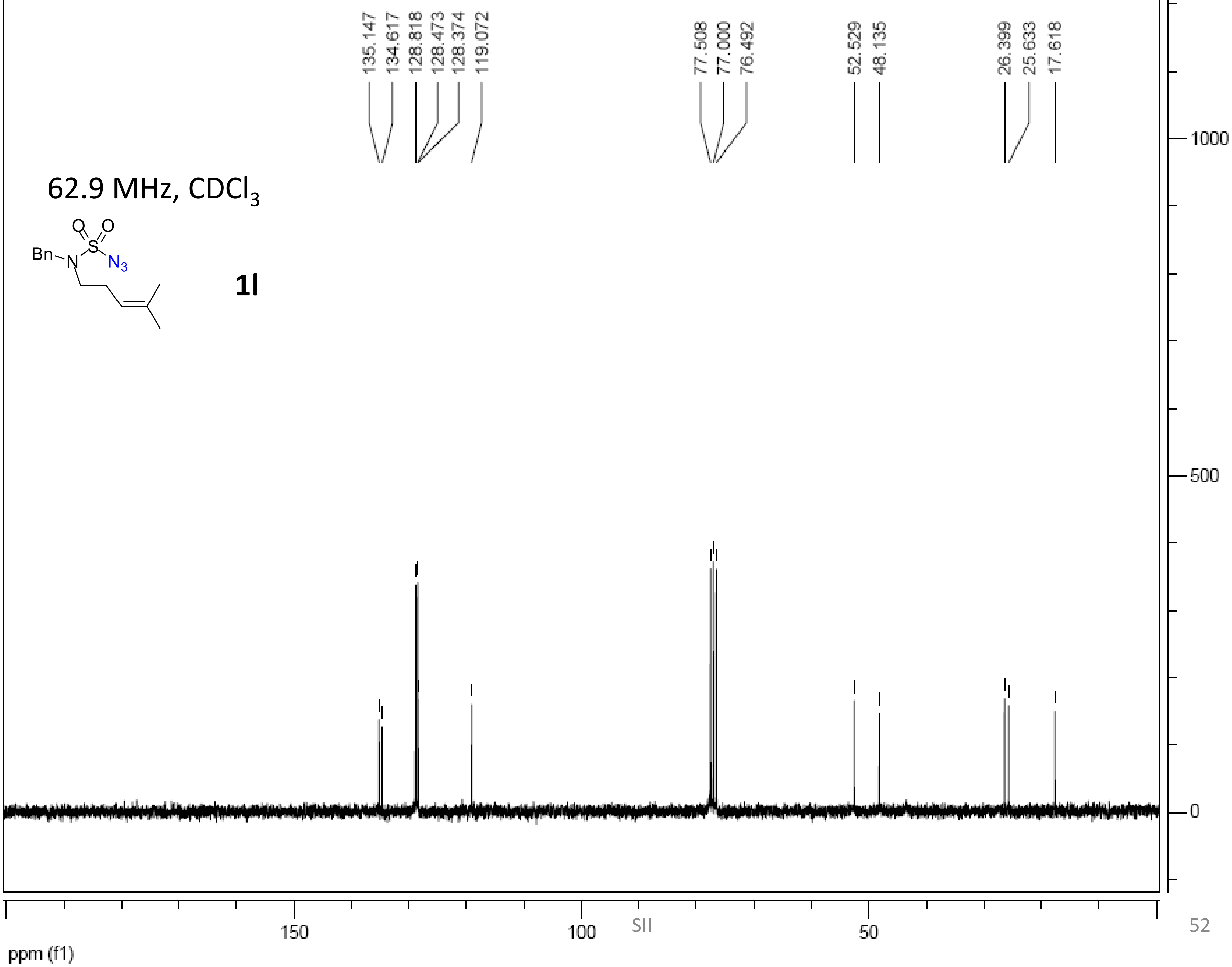
135.147
134.617
128.818
128.473
128.374
119.072
77.508
77.000
76.492
52.529
48.135
26.399
25.633
17.618
62.9 MHz, CDCl3
Bn
N
S
O
O
N3
1l
1000
500
0
150
100
50
ppm (f1)

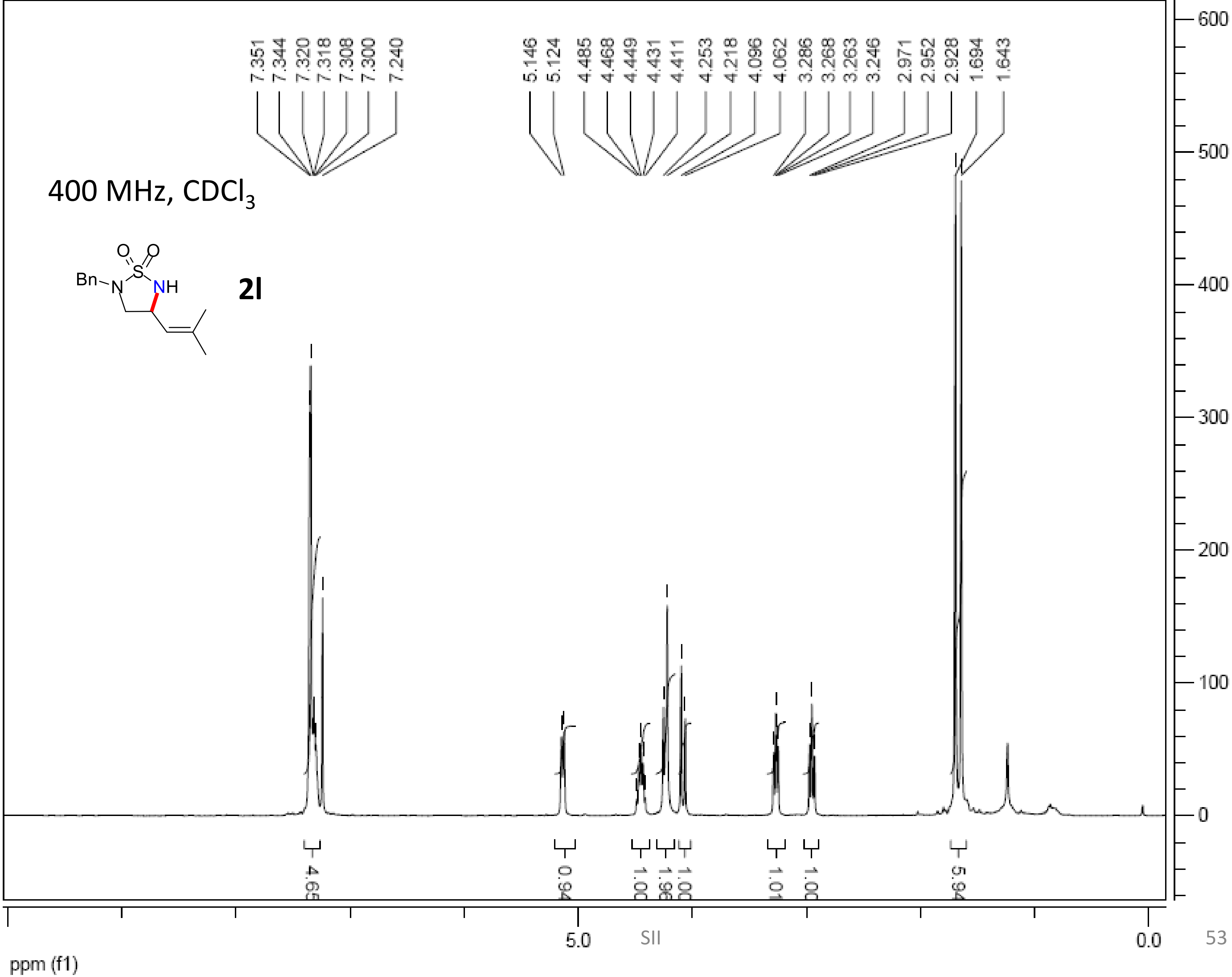
7.351 7.344 7.320 7.318 7.308 7.300 7.240
5.146 5.124 4.485 4.468 4.449 4.431 4.411 4.253 4.218 4.096 4.062 3.286 3.268 3.263 3.246 2.971 2.952 2.928 1.694 1.643
400 MHz, $CDCl_3$
Bn
N
S
O
O
NH
2l
4.65
0.94
1.00
1.96
1.00
1.01
1.00
5.94
600
500
400
300
200
100
0
5.0
0.0
ppm (f1)

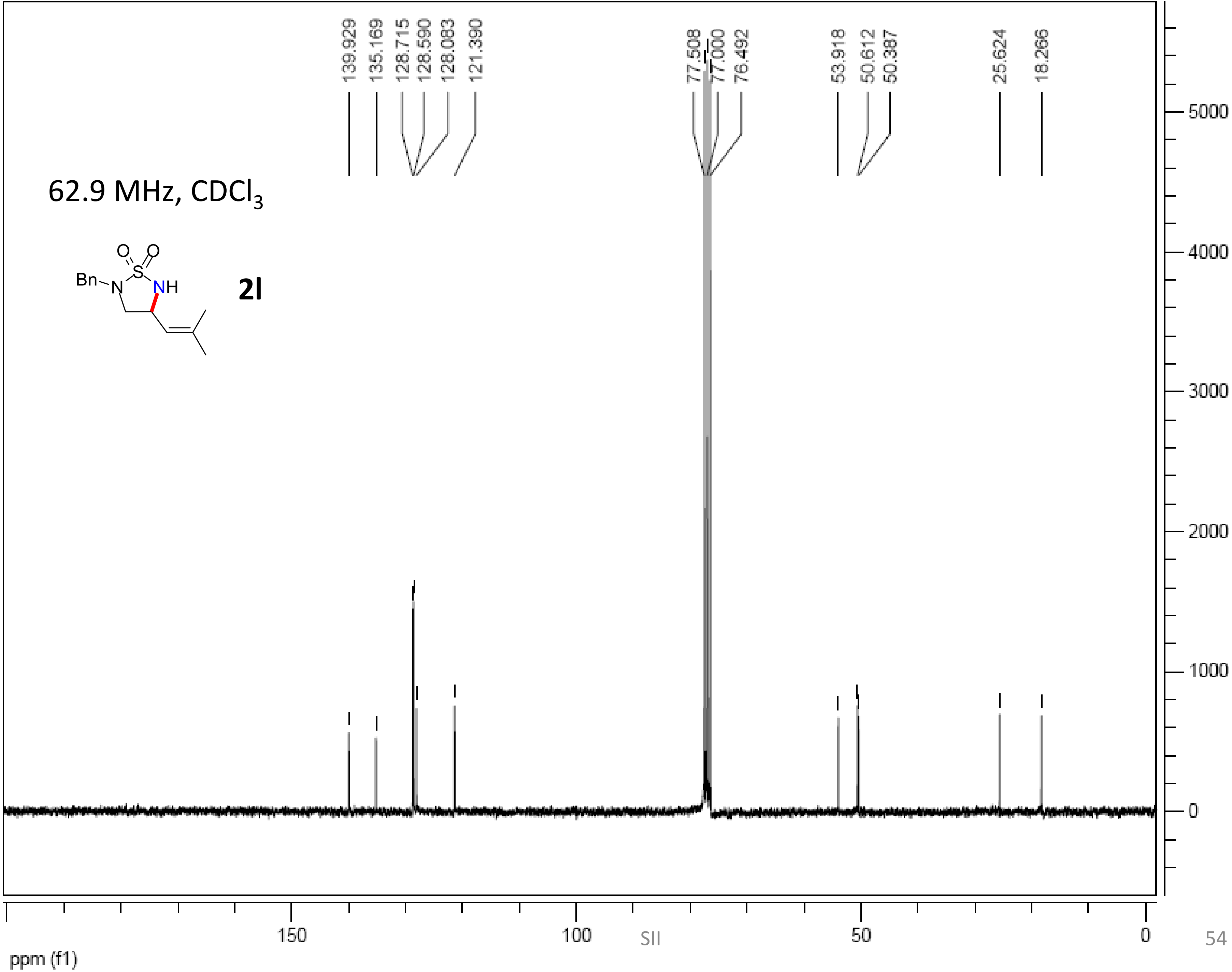
139.929
135.169
128.715
128.590
128.083
121.390
77.508
77.000
76.492
53.918
50.612
50.387
25.624
18.266
62.9 MHz, $CDCl_3$
O O
Bn
N
S
NH
2l
5000
4000
3000
2000
1000
0
150
100
50
0
ppm (f1)

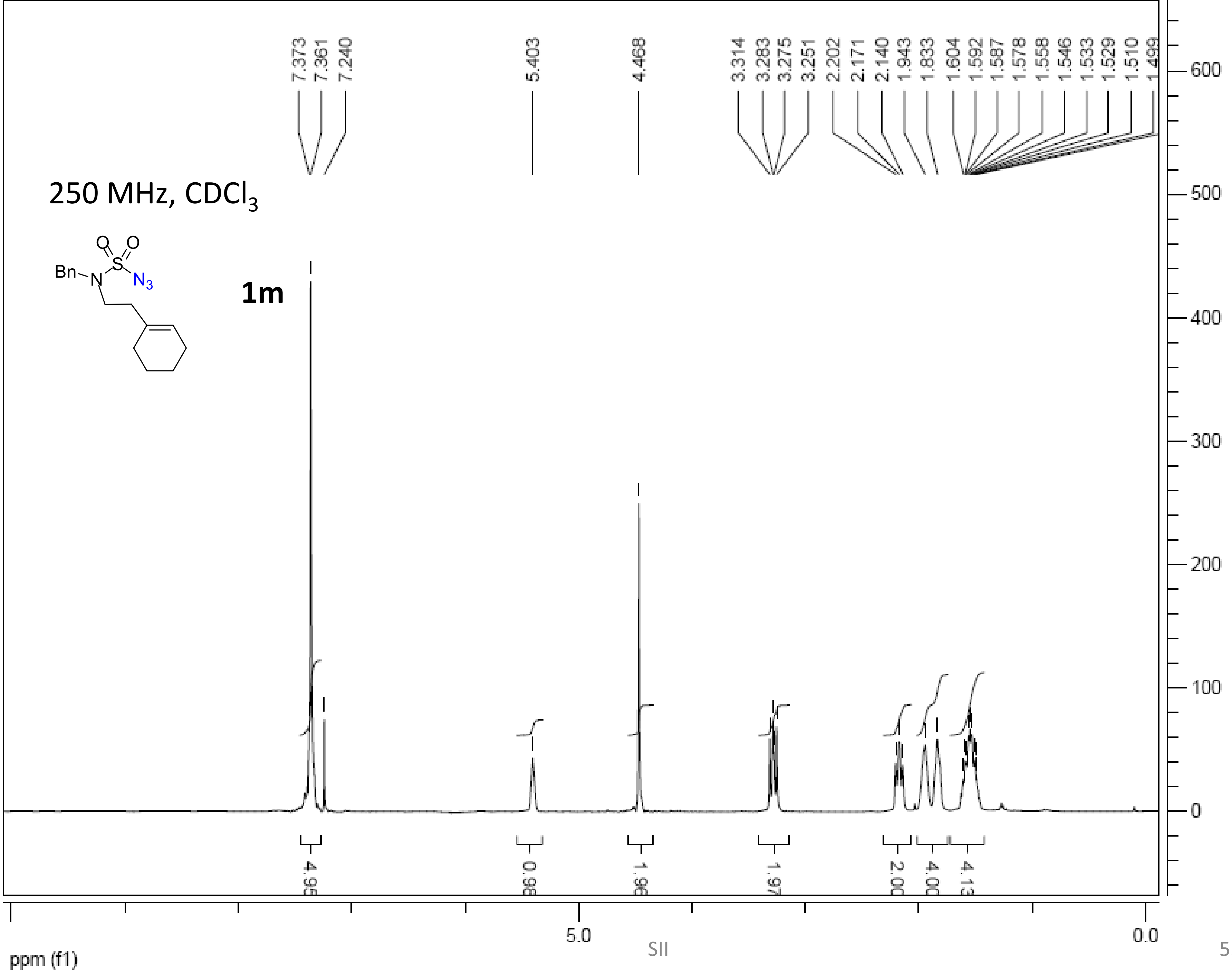
250 MHz, $CDCl_3$
1m
Bn
N
S
O
O
N_3
7.373
7.361
7.240
5.403
4.468
3.314
3.283
3.275
3.251
2.202
2.171
2.140
1.943
1.833
1.604
1.592
1.587
1.578
1.558
1.546
1.533
1.529
1.510
1.499
4.95
0.98
1.96
1.97
2.00
4.00
4.13
600
500
400
300
200
100
0
5.0
0.0
ppm (f1)

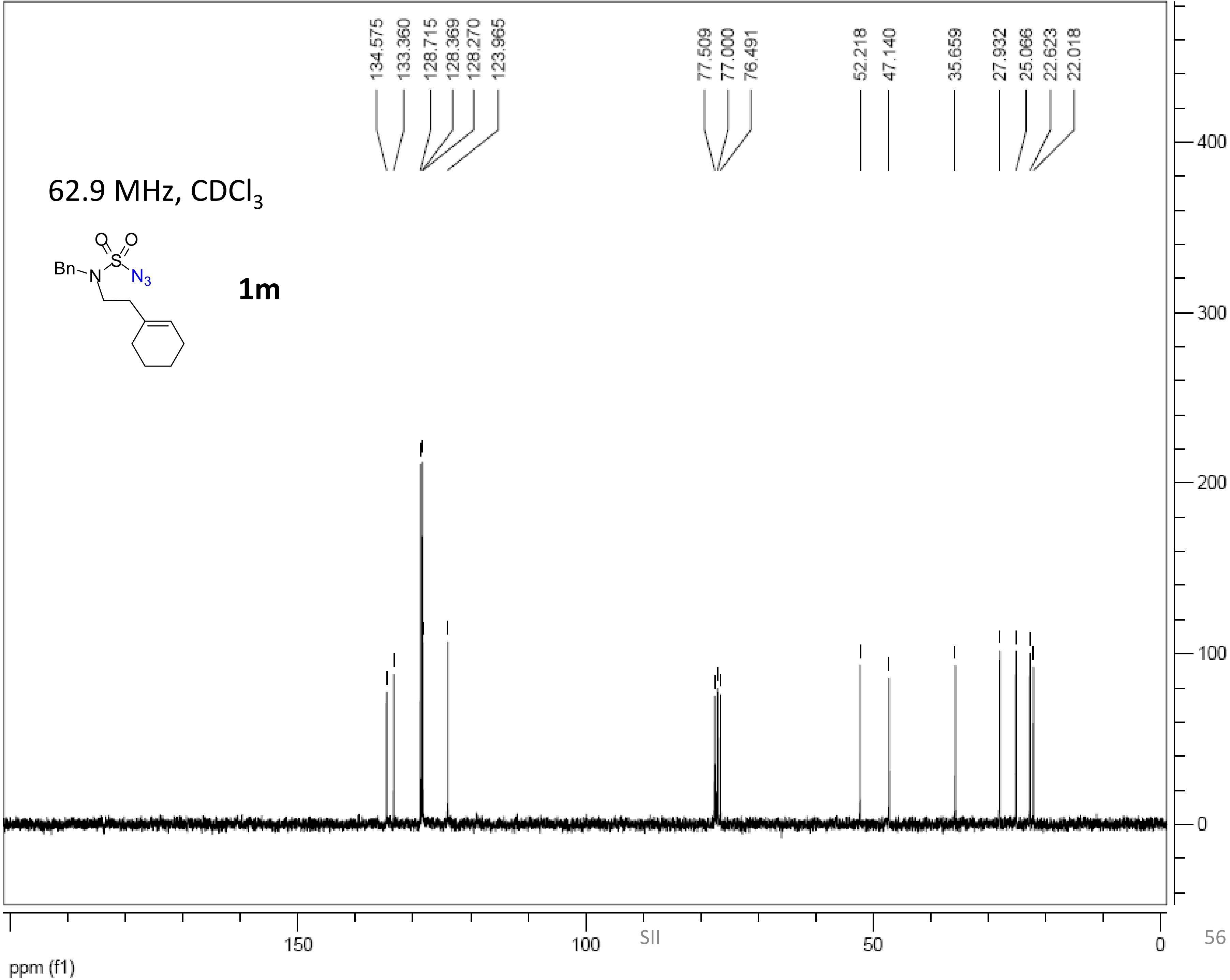
134.575
133.360
128.715
128.369
128.270
123.965
77.509
77.000
76.491
52.218
47.140
35.659
27.932
25.066
22.623
22.018
62.9 MHz, CDCl3
Bn
N
S
O
O
N3
1m
400
300
200
100
0
150
100
50
0
ppm (f1)

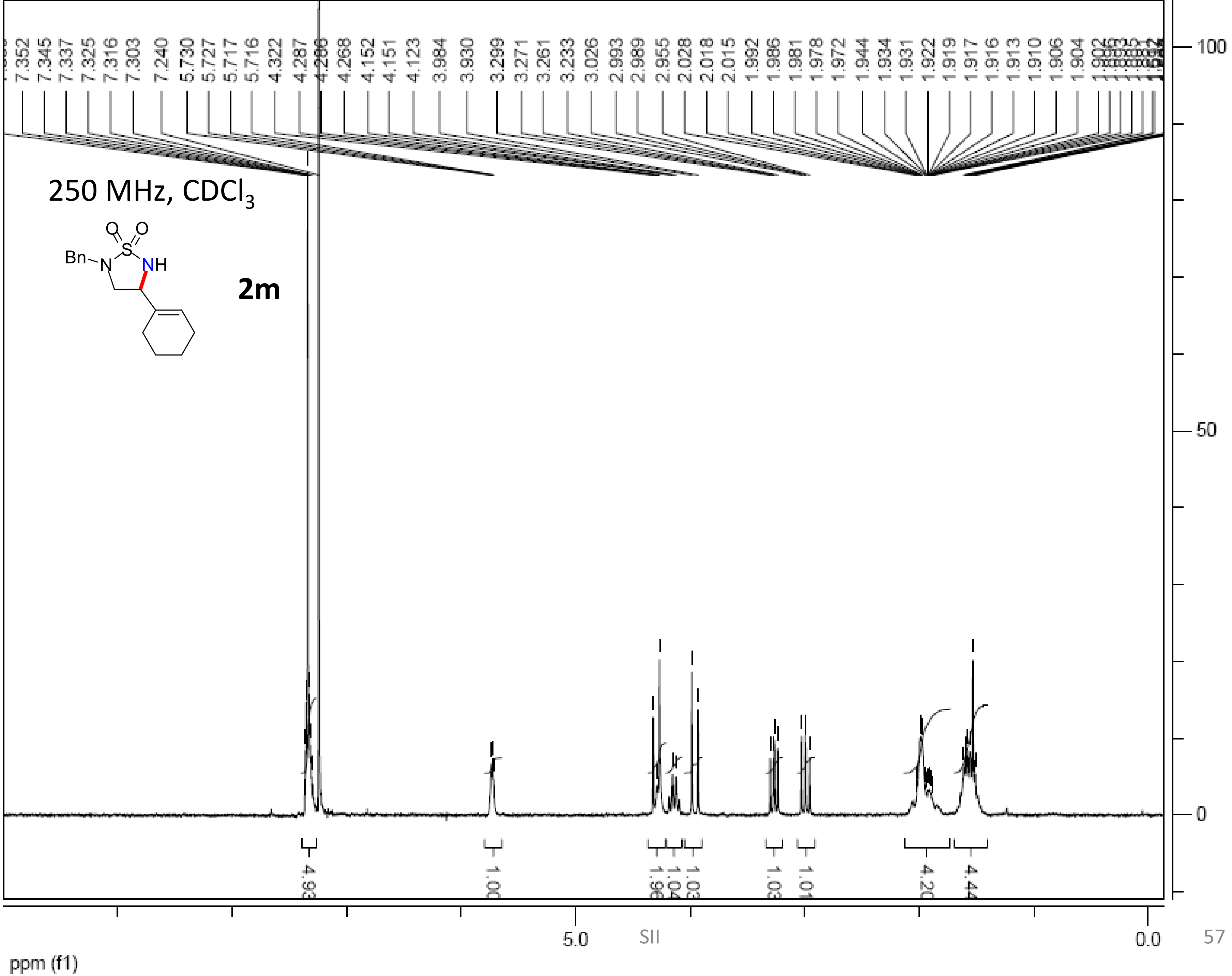

250 MHz, CDCl3
Bn
N
S
O
O
NH
2m
7.352
7.345
7.337
7.325
7.316
7.303
7.240
5.730
5.727
5.717
5.716
4.322
4.287
4.268
4.152
4.151
4.123
3.984
3.930
3.299
3.271
3.261
3.233
3.026
2.993
2.989
2.955
2.028
2.018
2.015
1.992
1.986
1.981
1.978
1.972
1.944
1.934
1.931
1.922
1.919
1.917
1.916
1.913
1.910
1.906
1.904
100
50
0
4.93
1.00
1.96
1.04
1.03
1.03
1.01
4.20
4.44
5.0
0.0
ppm (f1)

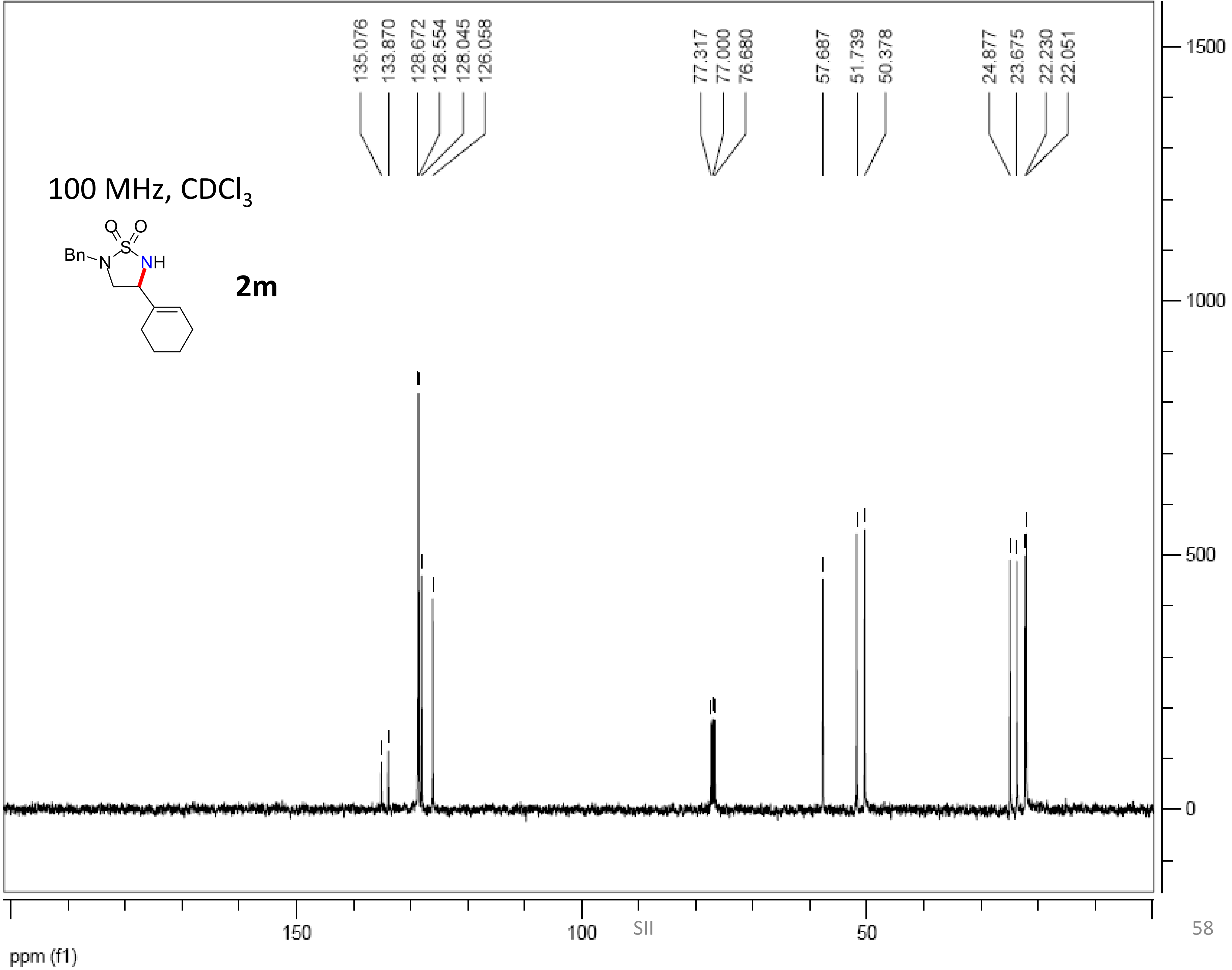

100 MHz, $CDCl_3$

2m

SII

400 MHz, $CDCl_3$

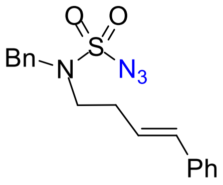

1n

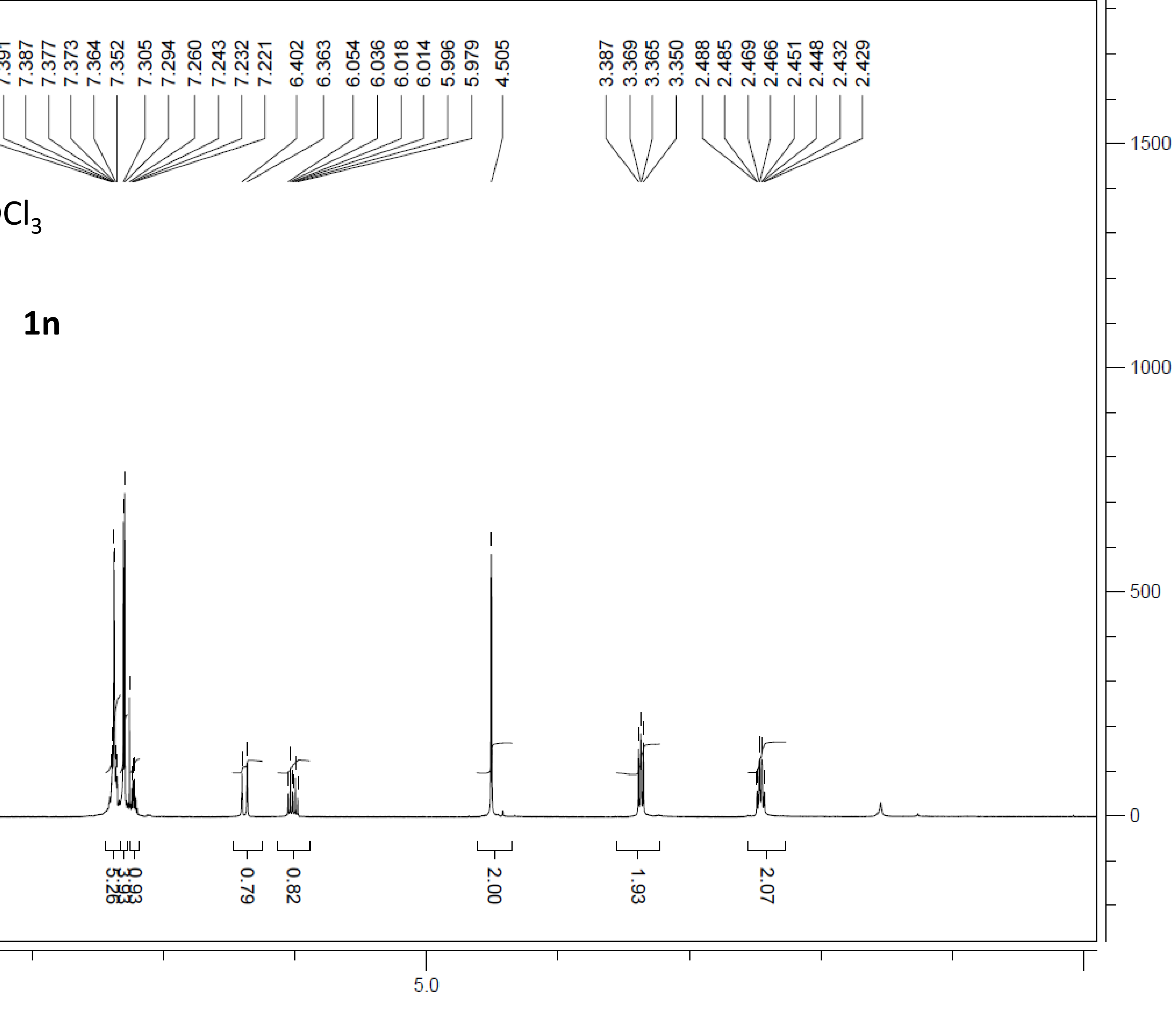

100 MHz, $CDCl_3$

1n

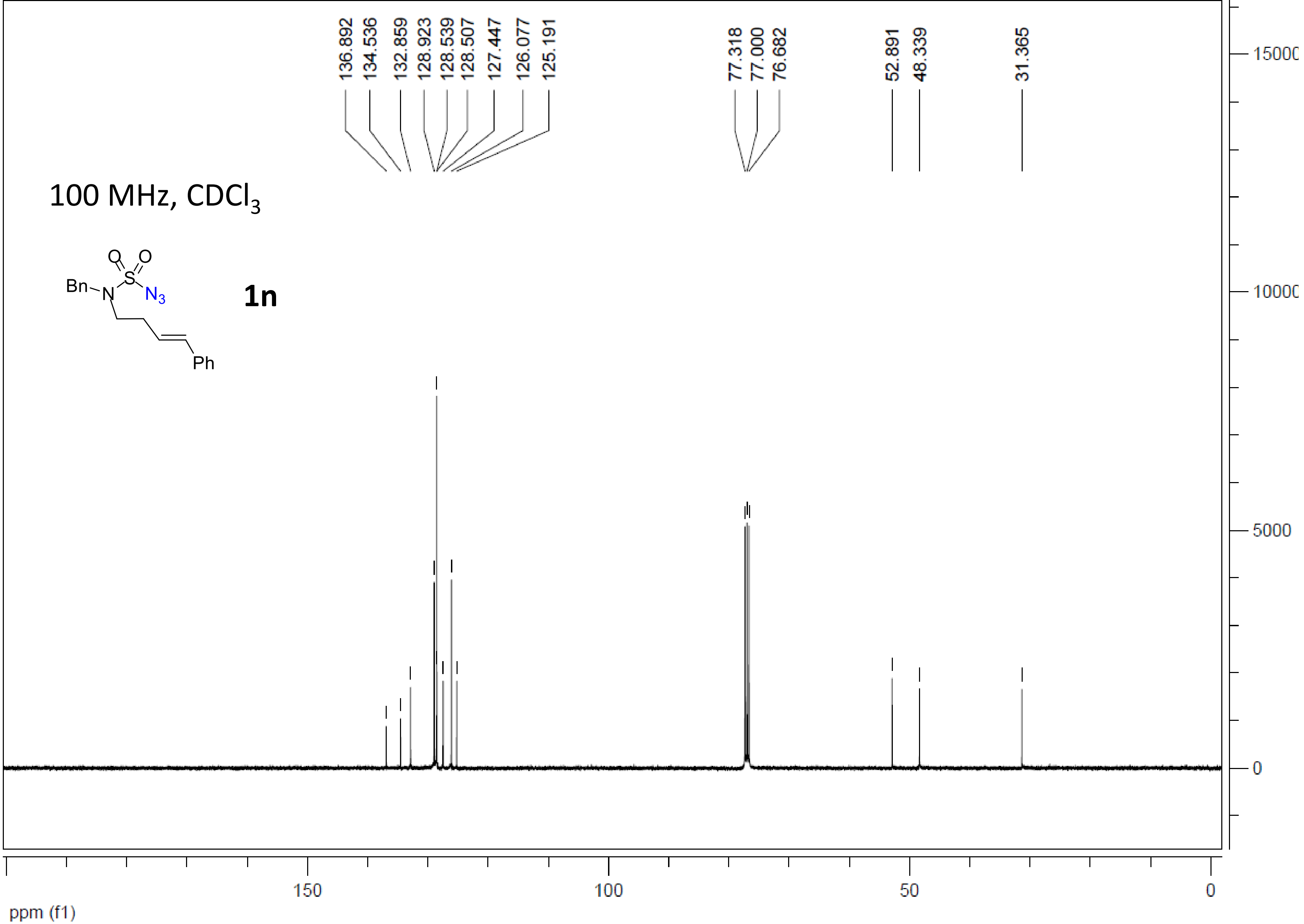

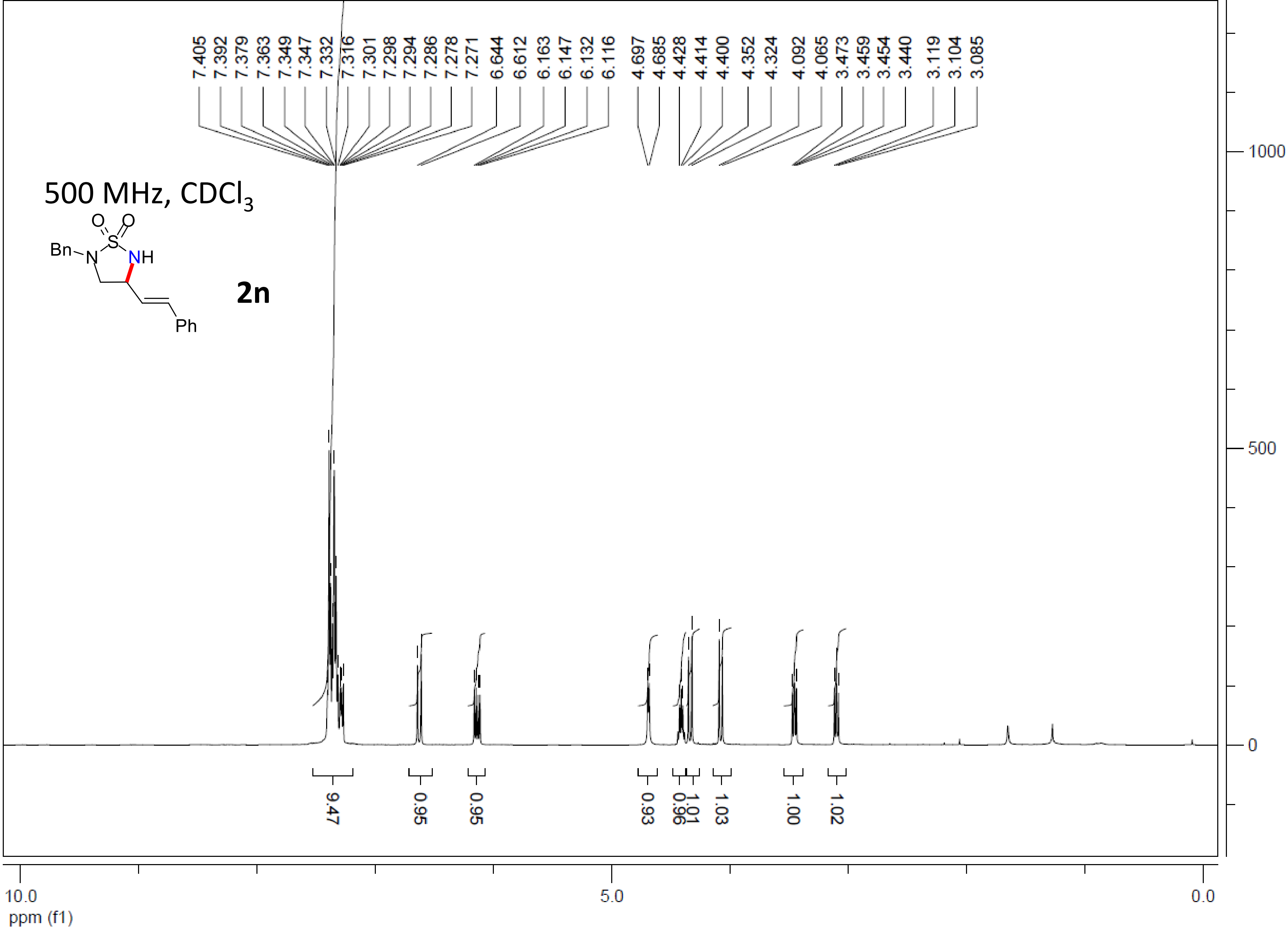
7.405 7.392 7.379 7.363 7.349 7.347 7.332 7.316 7.301 7.298 7.294 7.286 7.278 7.271 6.644 6.612 6.163 6.147 6.132 6.116 4.697 4.685 4.428 4.414 4.400 4.352 4.324 4.092 4.065 3.473 3.459 3.454 3.440 3.119 3.104 3.085
500 MHz, CDCl3
Bn
N
S
O
O
NH
Ph
2n
9.47
0.95
0.95
0.93
0.96
1.01
1.03
1.00
1.02
1000
500
0
10.0
5.0
0.0
ppm (f1)

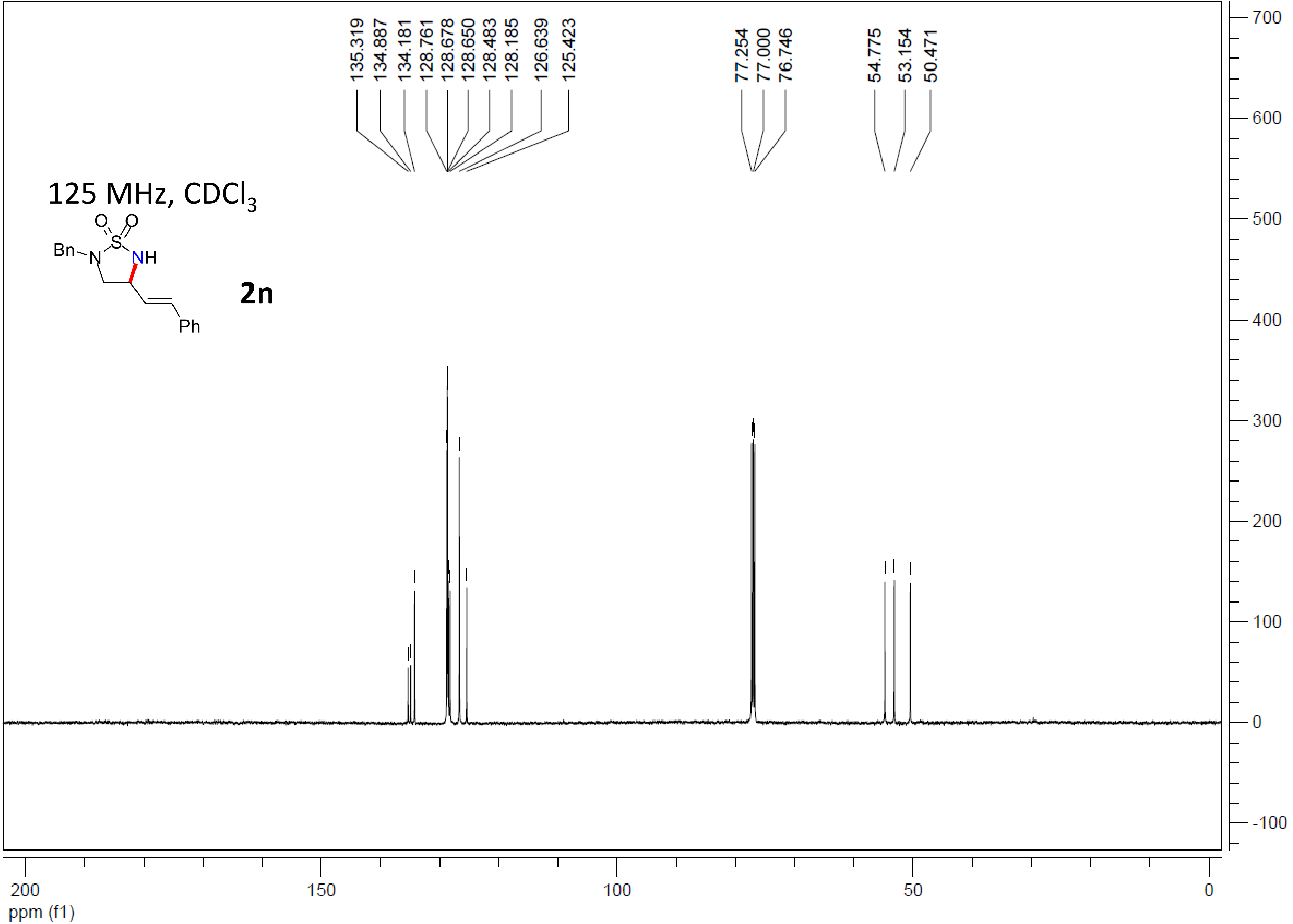

135.319
134.887
134.181
128.761
128.678
128.650
128.483
128.185
126.639
125.423
77.254
77.000
76.746
54.775
53.154
50.471
125 MHz, $CDCl_3$
O
O
S
Bn
N
NH
Ph
2n
700
600
500
400
300
200
100
0
-100
200
150
100
50
0
ppm (f1)

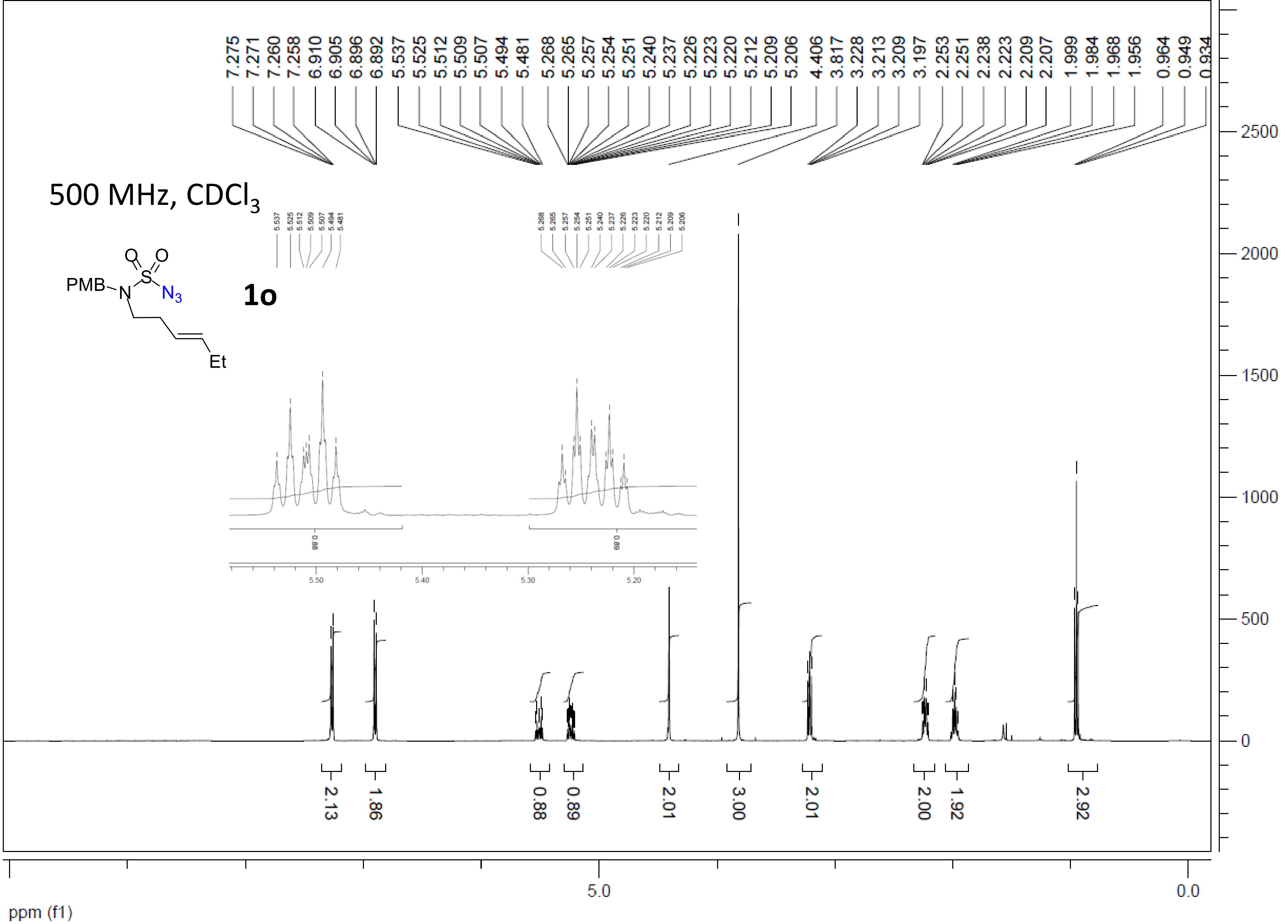
500 MHz, CDCl3
PMB
N
S
O
O
N3
Et
1o
7.275
7.271
7.260
7.258
6.910
6.905
6.896
6.892
5.537
5.525
5.512
5.509
5.507
5.494
5.481
5.268
5.265
5.257
5.254
5.251
5.240
5.237
5.226
5.223
5.220
5.212
5.209
5.206
4.406
3.817
3.228
3.213
3.209
3.197
2.253
2.251
2.238
2.223
2.209
2.207
1.999
1.984
1.968
1.956
0.964
0.949
0.934
2.13
1.86
0.88
0.89
2.01
3.00
2.01
2.00
1.92
2.92
5.0
0.0
ppm (f1)

125 MHz, $CDCl_3$

1o

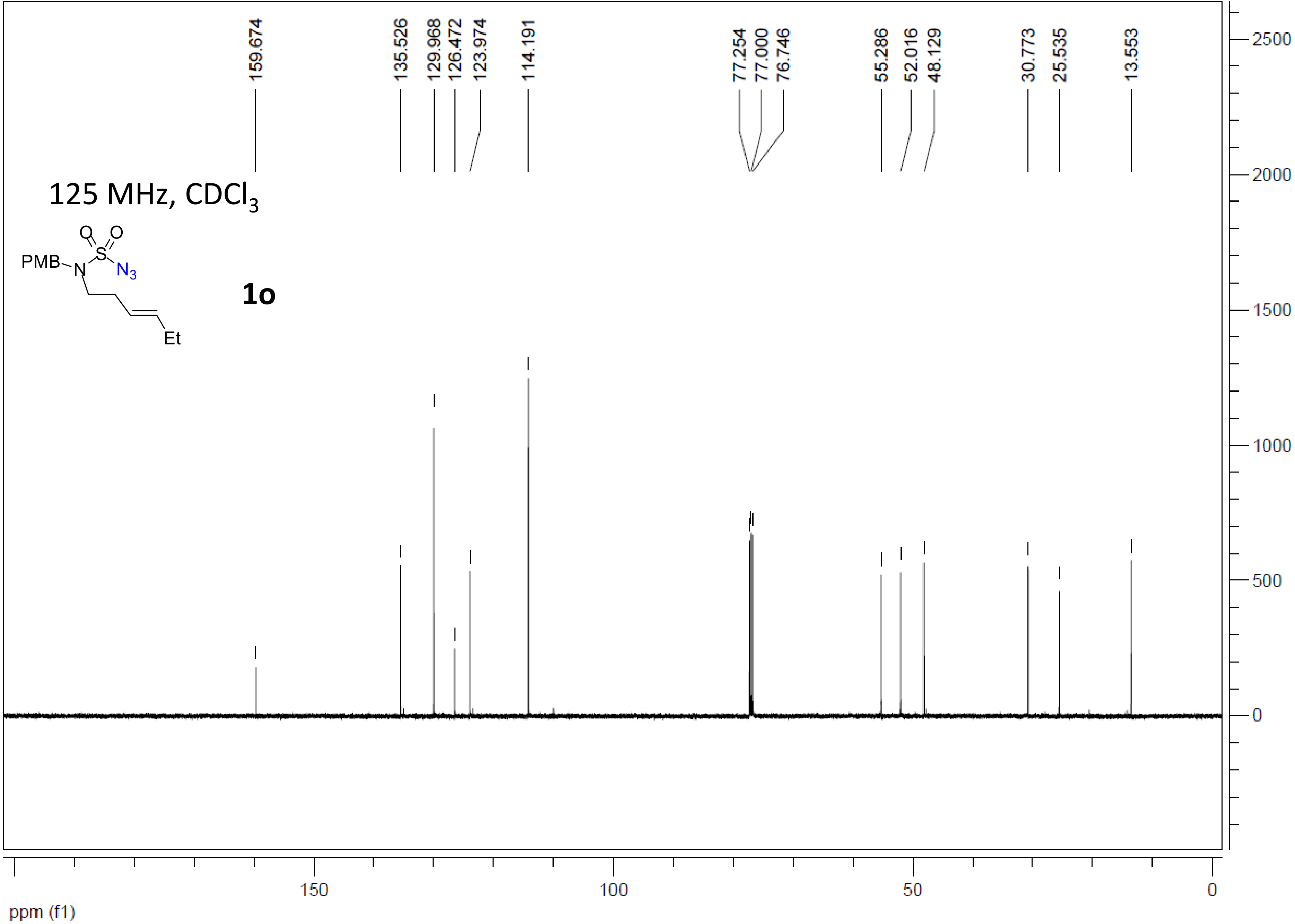

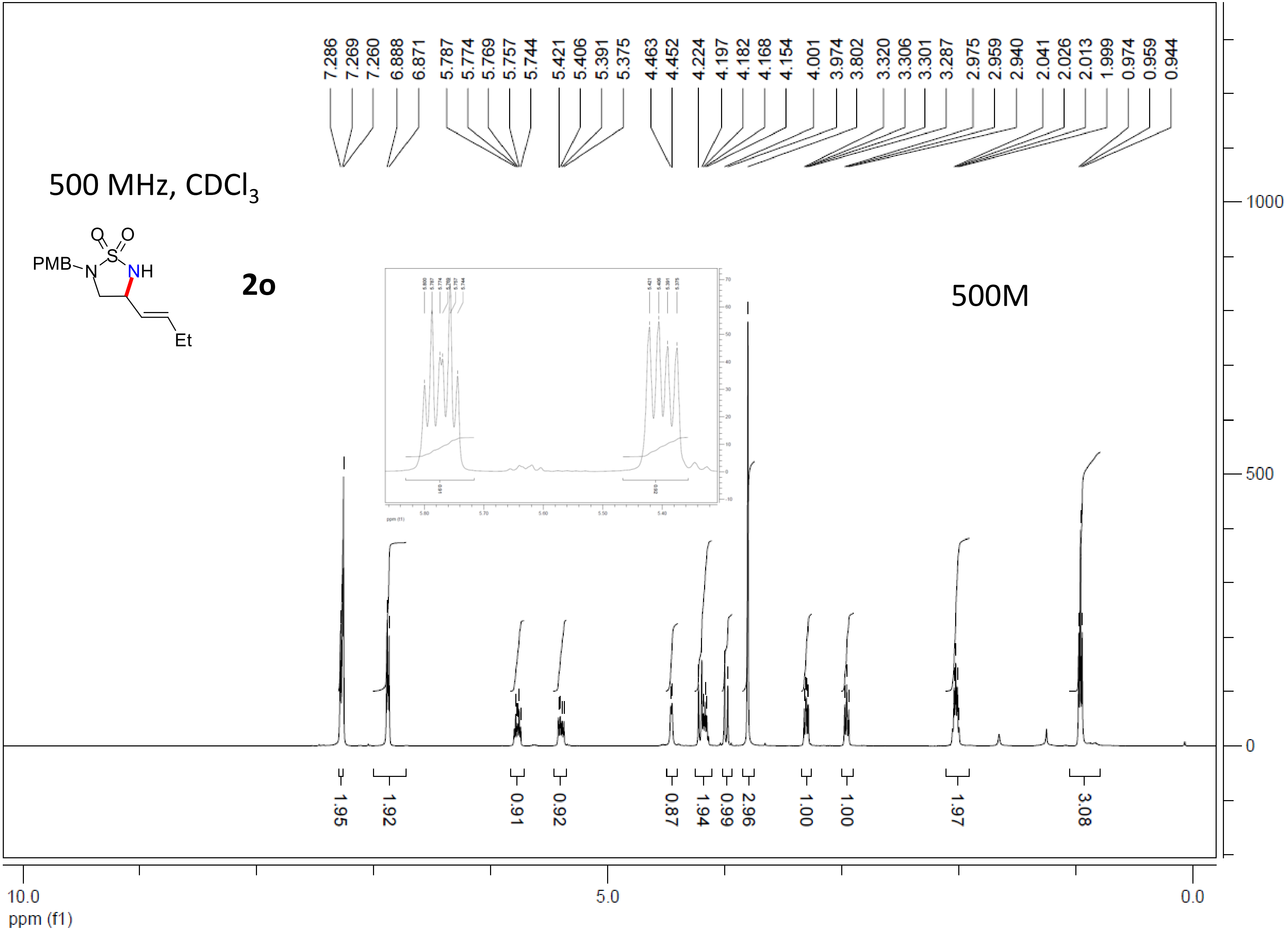

500 MHz, CDCl3
PMB
N
S
O
O
NH
Et
2o
500M
7.286
7.269
7.260
6.888
6.871
5.787
5.774
5.769
5.757
5.744
5.421
5.406
5.391
5.375
4.463
4.452
4.224
4.197
4.182
4.168
4.154
4.001
3.974
3.802
3.320
3.306
3.301
3.287
2.975
2.959
2.940
2.041
2.026
2.013
1.999
0.974
0.959
0.944
1.95
1.92
0.91
0.92
0.87
1.94
0.99
2.96
1.00
1.00
1.97
3.08
1000
500
0
10.0
5.0
0.0
ppm (f1)

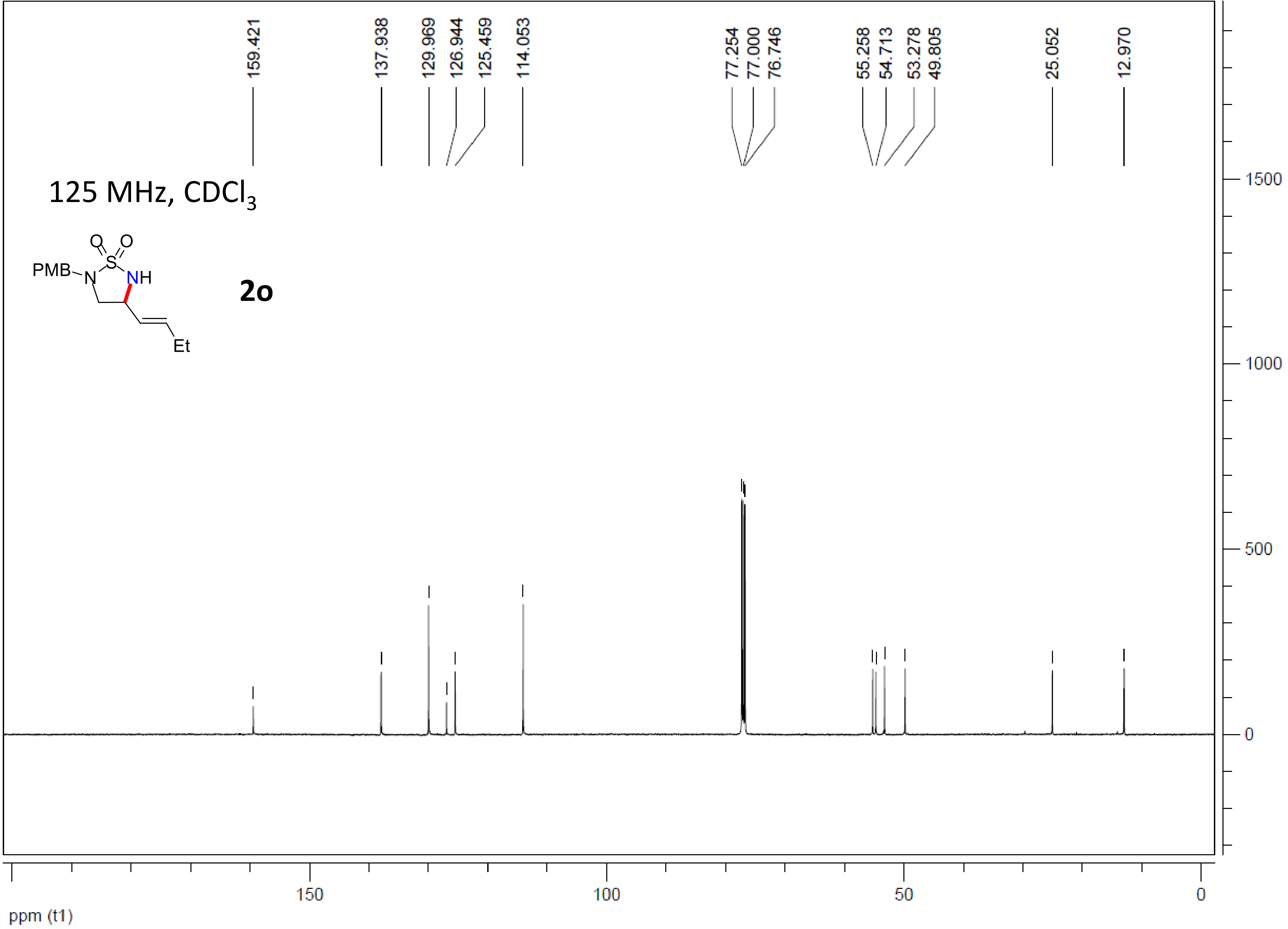
159.421
137.938
129.969
126.944
125.459
114.053
77.254
77.000
76.746
55.258
54.713
53.278
49.805
25.052
12.970
125 MHz, $CDCl_3$
PMB
N
S
O
O
NH
Et
2o
1500
1000
500
0
150
100
50
0
ppm (t1)

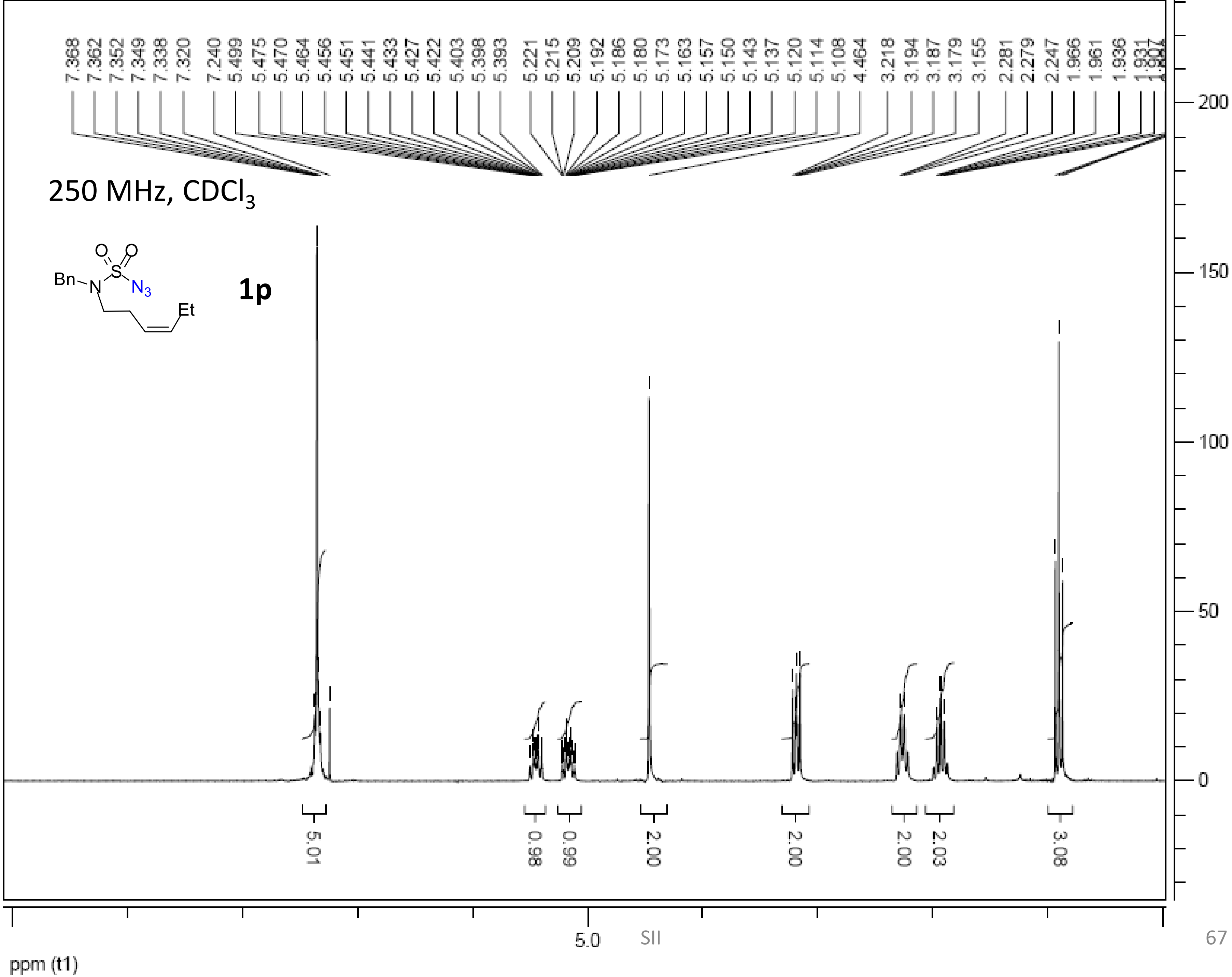

250 MHz, CDCl3
1p
Bn
N
S
O
O
N3
Et
7.368
7.362
7.352
7.349
7.338
7.320
7.240
5.499
5.475
5.470
5.464
5.456
5.451
5.441
5.433
5.427
5.422
5.403
5.398
5.393
5.221
5.215
5.209
5.192
5.186
5.180
5.173
5.163
5.157
5.150
5.143
5.137
5.120
5.114
5.108
4.464
3.218
3.194
3.187
3.179
3.155
2.281
2.279
2.247
1.966
1.961
1.936
1.931
200
150
100
50
0
5.01
0.98
0.99
2.00
2.00
2.00
2.03
3.08
5.0
ppm (t1)

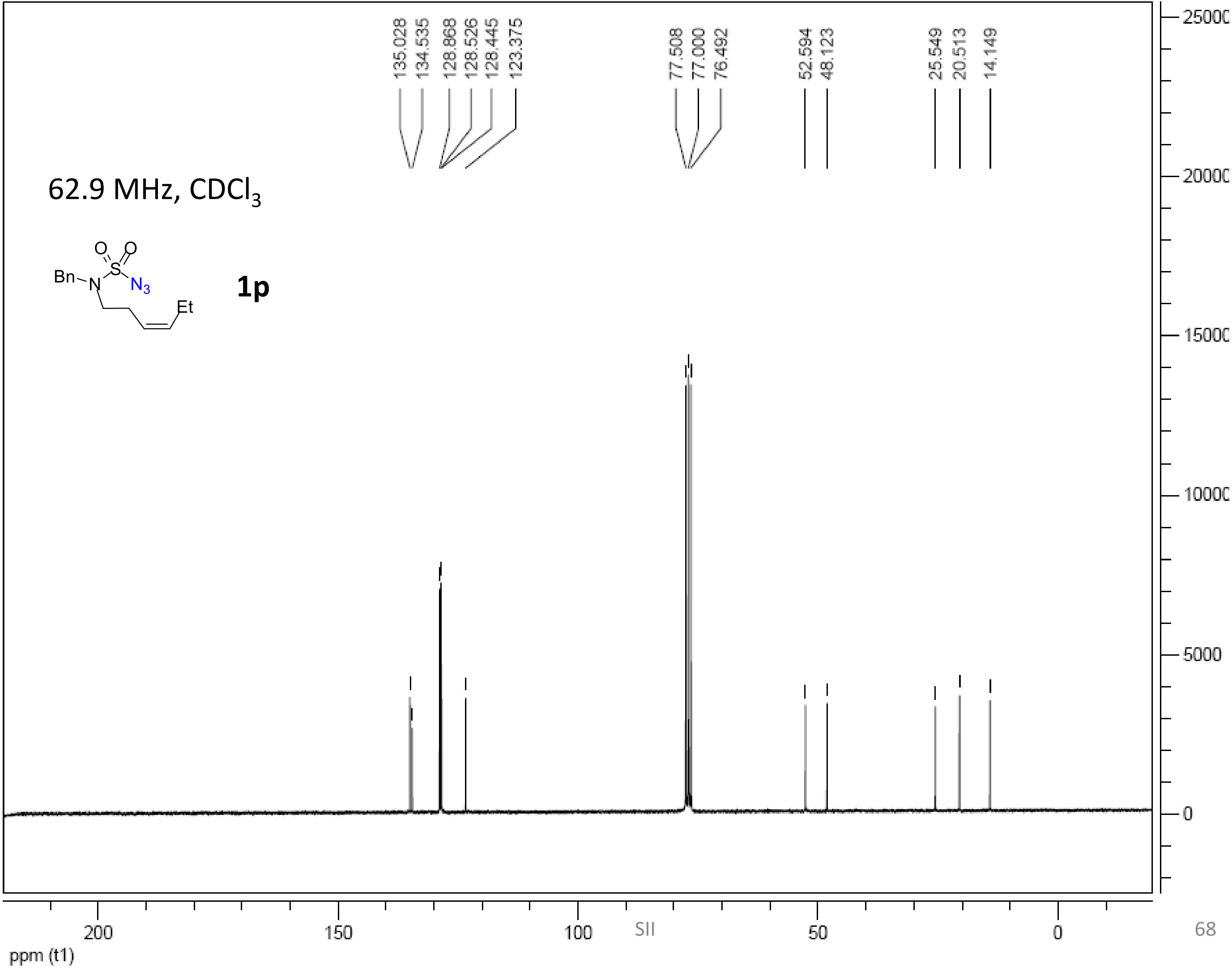
135.028
134.535
128.868
128.526
128.445
123.375
77.508
77.000
76.492
52.594
48.123
25.549
20.513
14.149
62.9 MHz, CDCl3
Bn
N
S
O
O
N3
Et
1p
25000
20000
15000
10000
5000
0
200
150
100
50
0
ppm (t1)

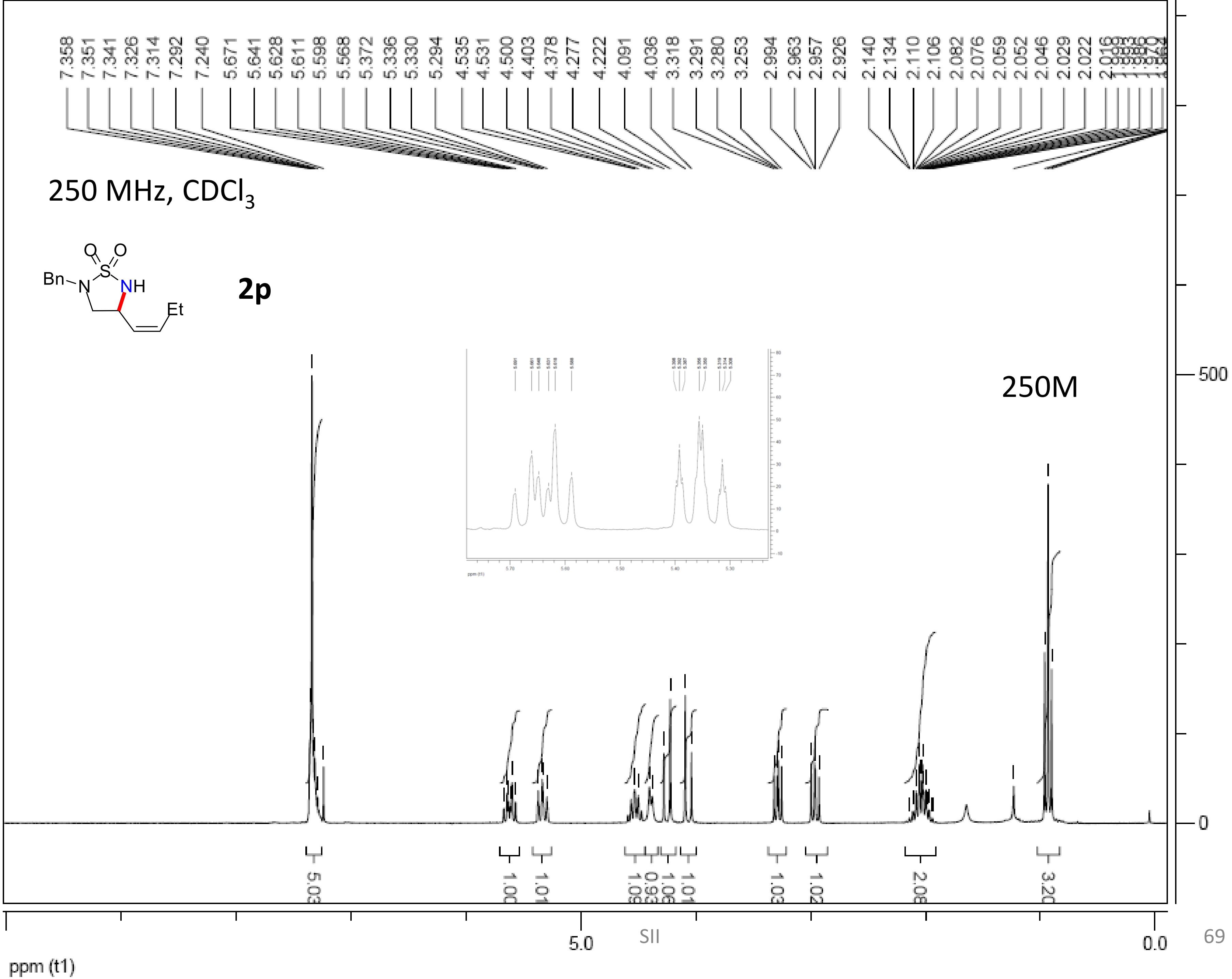

7.358 7.351 7.341 7.326 7.314 7.292 7.240 5.671 5.641 5.628 5.611 5.598 5.568 5.372 5.336 5.330 5.294 4.535 4.531 4.500 4.403 4.378 4.277 4.222 4.091 4.036 3.318 3.291 3.280 3.253 2.994 2.963 2.957 2.926 2.140 2.134 2.110 2.106 2.082 2.076 2.059 2.052 2.046 2.029 2.022 2.016
250 MHz, CDCl3
O O
Bn
N
S
NH
Et
2p
250M
500
0
5.03
1.00
1.01
1.09
0.93
1.06
1.01
1.03
1.02
2.08
3.20
5.0
0.0
ppm (t1)

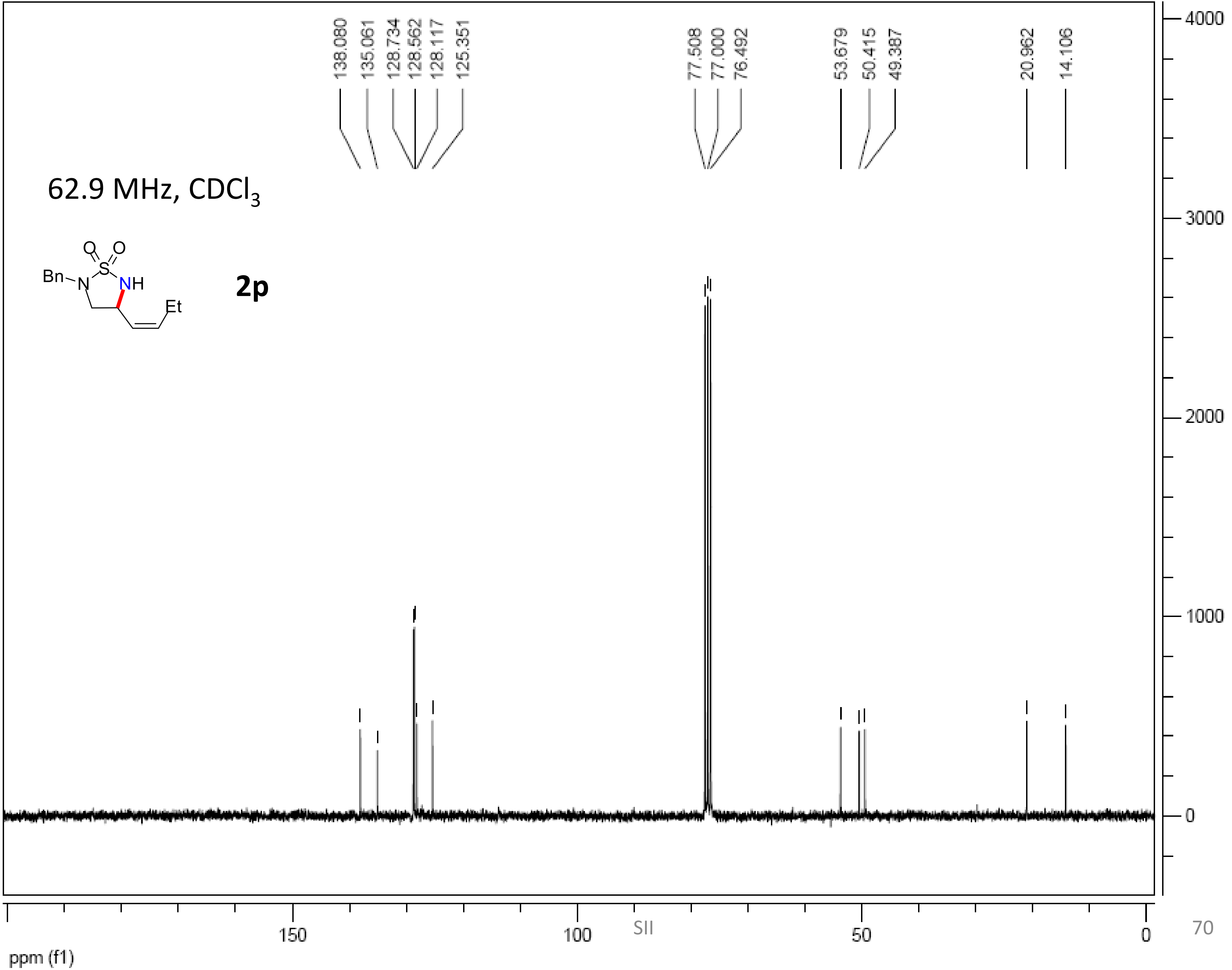

138.080
135.061
128.734
128.562
128.117
125.351
77.508
77.000
76.492
53.679
50.415
49.387
20.962
14.106
62.9 MHz, CDCl3
Bn
N
S
O
O
NH
Et
2p
4000
3000
2000
1000
0
150
100
50
0
ppm (f1)

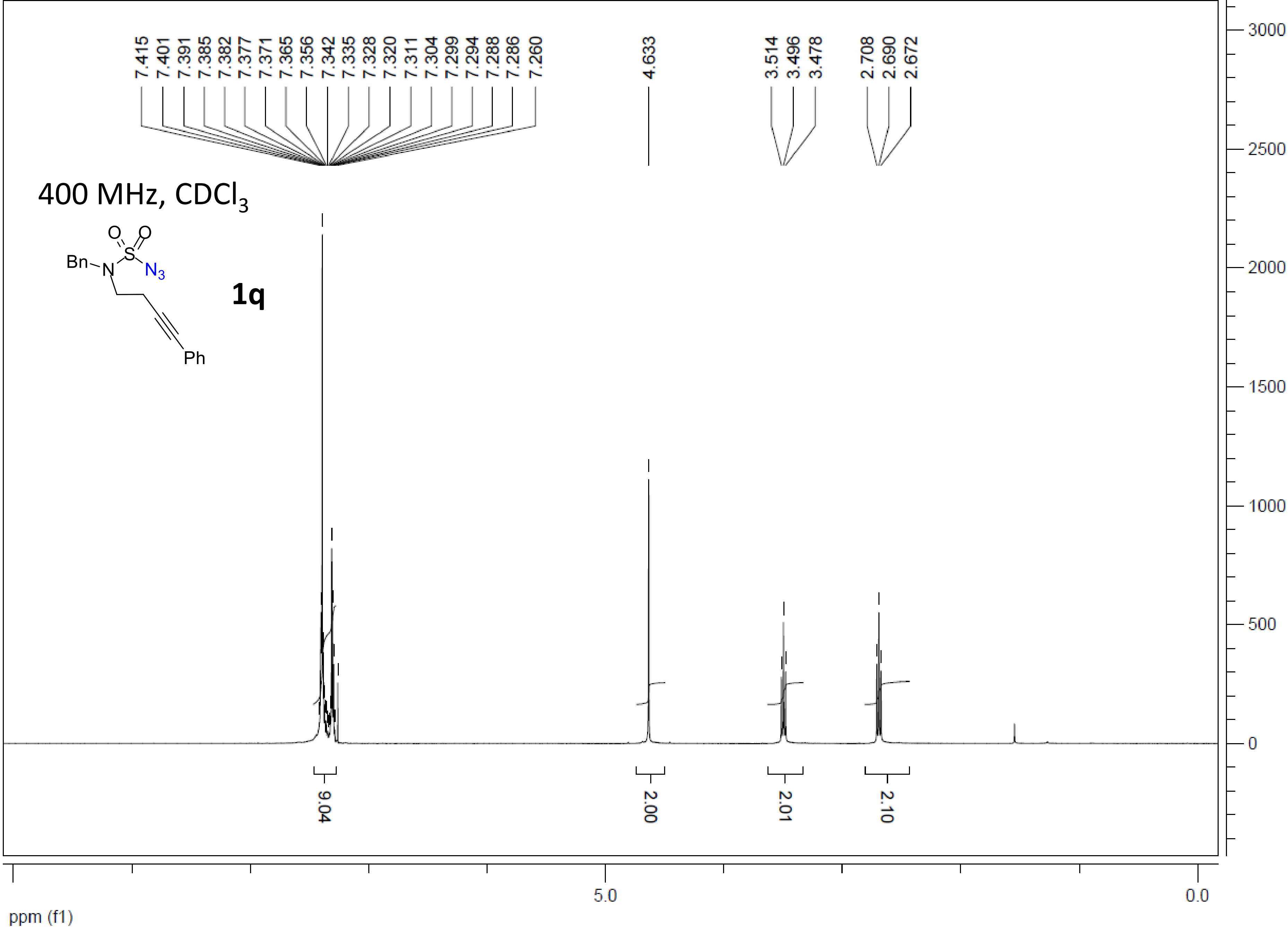

7.415 7.401 7.391 7.385 7.382 7.377 7.371 7.365 7.356 7.342 7.335 7.328 7.320 7.311 7.304 7.299 7.294 7.288 7.286 7.260
4.633
3.514 3.496 3.478
2.708 2.690 2.672
400 MHz, CDCl3
Bn
N
S
O
O
N3
Ph
1q
9.04
2.00
2.01
2.10
3000
2500
2000
1500
1000
500
0
5.0
0.0
ppm (f1)

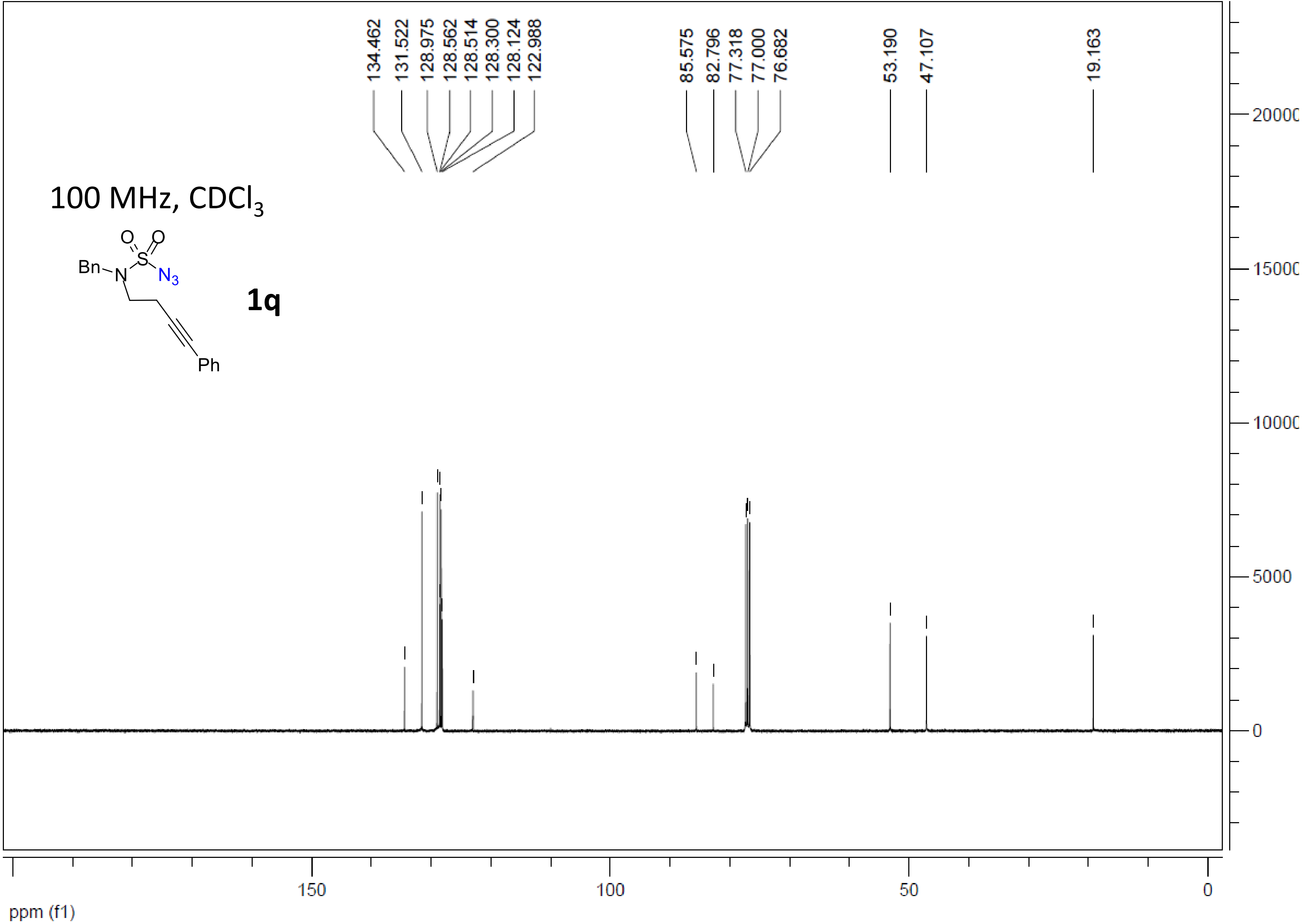
134.462
131.522
128.975
128.562
128.514
128.300
128.124
122.988
85.575
82.796
77.318
77.000
76.682
53.190
47.107
19.163
100 MHz, CDCl3
Bn
N
S
O
O
N3
Ph
1q
20000
15000
10000
5000
0
150
100
50
0
ppm (f1)

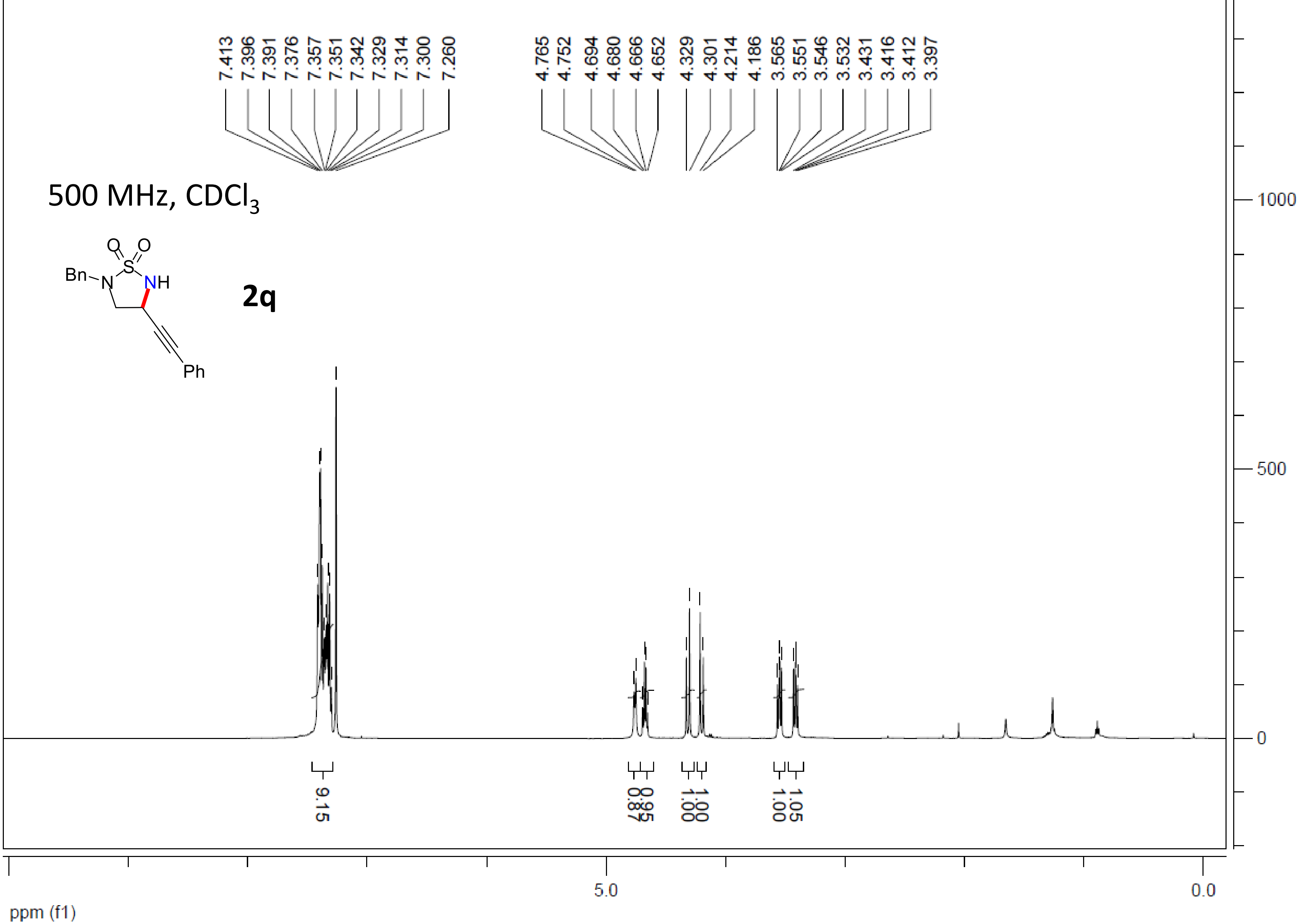
500 MHz, $CDCl_3$
2q
Bn
N
S
O
O
NH
Ph
7.413
7.396
7.391
7.376
7.357
7.351
7.342
7.329
7.314
7.300
7.260
4.765
4.752
4.694
4.680
4.666
4.652
4.329
4.301
4.214
4.186
3.565
3.551
3.546
3.532
3.431
3.416
3.412
3.397
9.15
0.87
0.95
1.00
1.00
1.00
1.05
1000
500
0
5.0
0.0
ppm (f1)

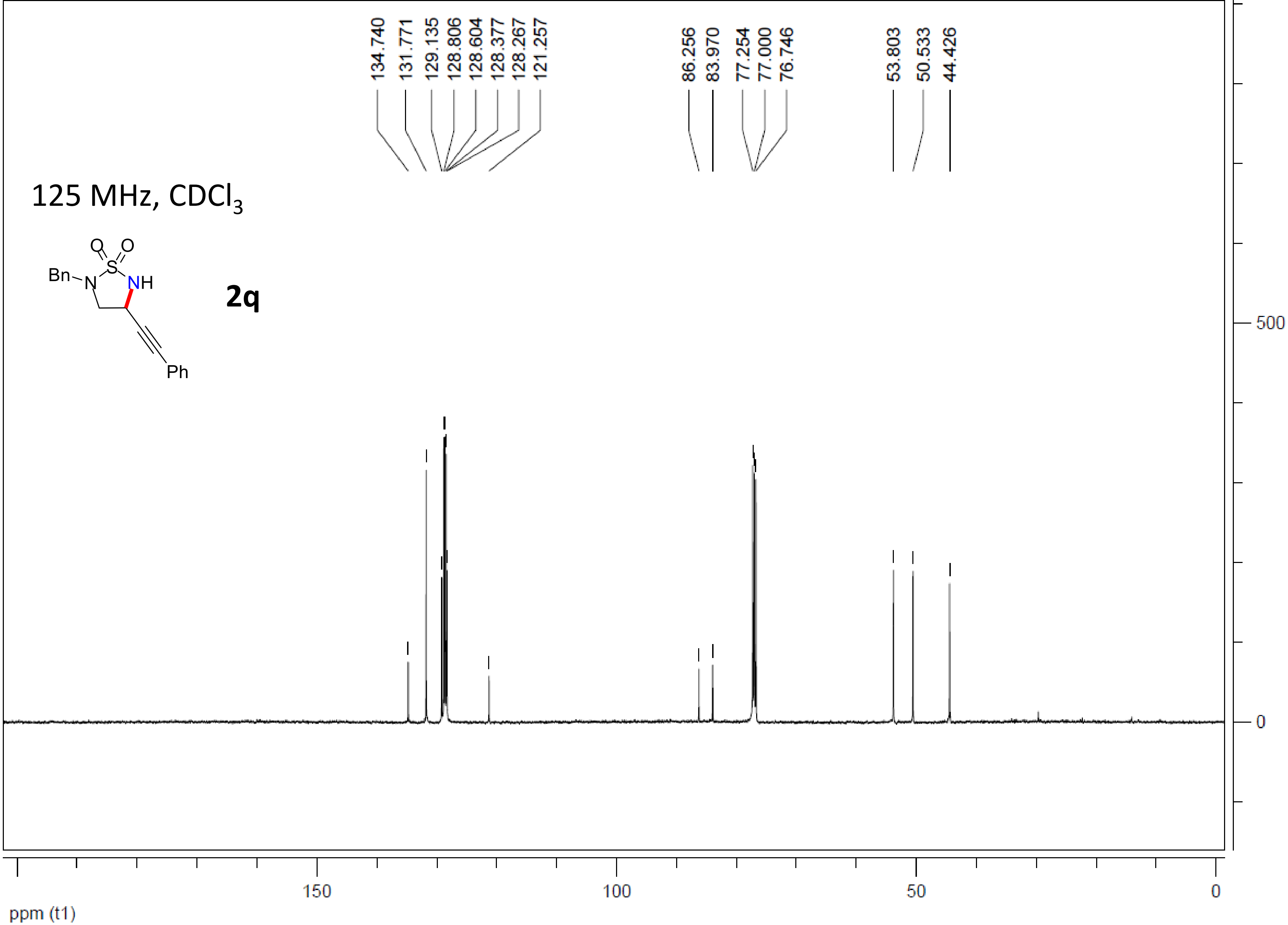
134.740
131.771
129.135
128.806
128.604
128.377
128.267
121.257
86.256
83.970
77.254
77.000
76.746
53.803
50.533
44.426
125 MHz, CDCl3
Bn
N
S
O
O
NH
Ph
2q
500
0
150
100
50
0
ppm (t1)

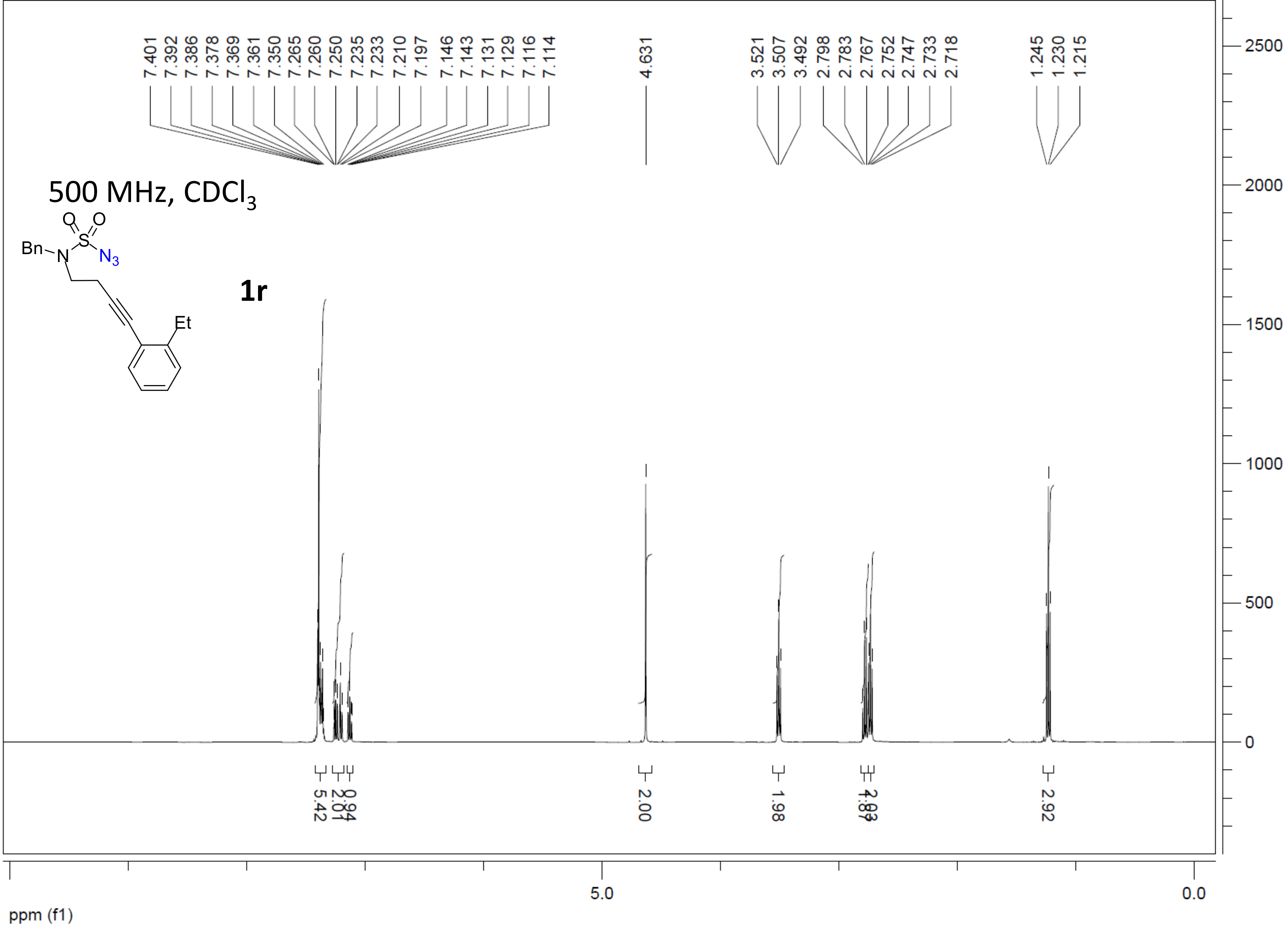
7.401 7.392 7.386 7.378 7.369 7.361 7.350 7.265 7.260 7.250 7.235 7.233 7.210 7.197 7.146 7.143 7.131 7.129 7.116 7.114
4.631
3.521 3.507 3.492 2.798 2.783 2.767 2.752 2.747 2.733 2.718
1.245 1.230 1.215
500 MHz, CDCl3
Bn
N
S
O
O
N3
Et
1r
5.42
2.00
1.98
2.92
2500
2000
1500
1000
500
0
5.0
0.0
ppm (f1)

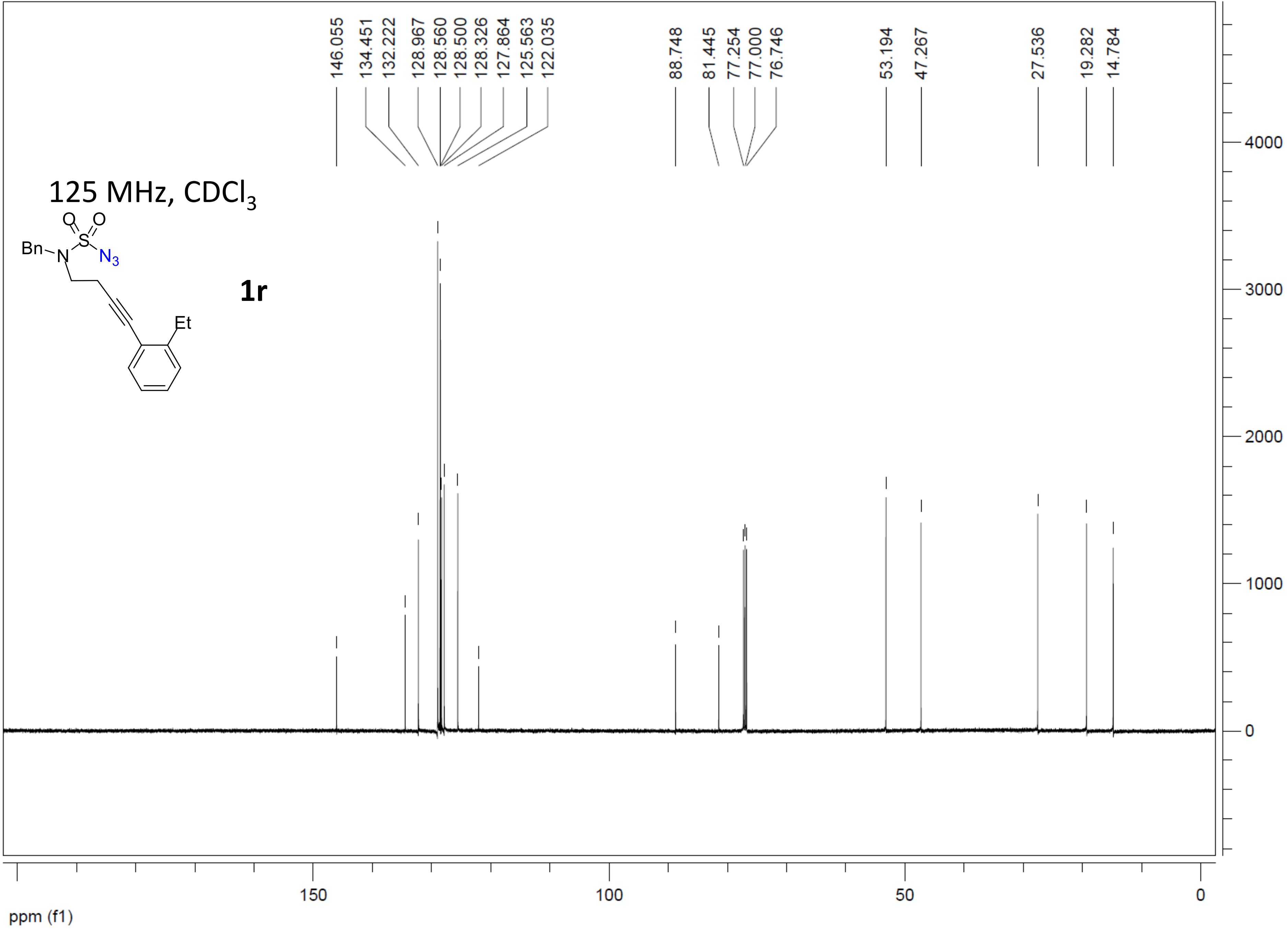
146.055
134.451
132.222
128.967
128.560
128.500
128.326
127.864
125.563
122.035
88.748
81.445
77.254
77.000
76.746
53.194
47.267
27.536
19.282
14.784
125 MHz, CDCl3
Bn
N
S
O
O
N3
Et
1r
4000
3000
2000
1000
0
150
100
50
0
ppm (f1)

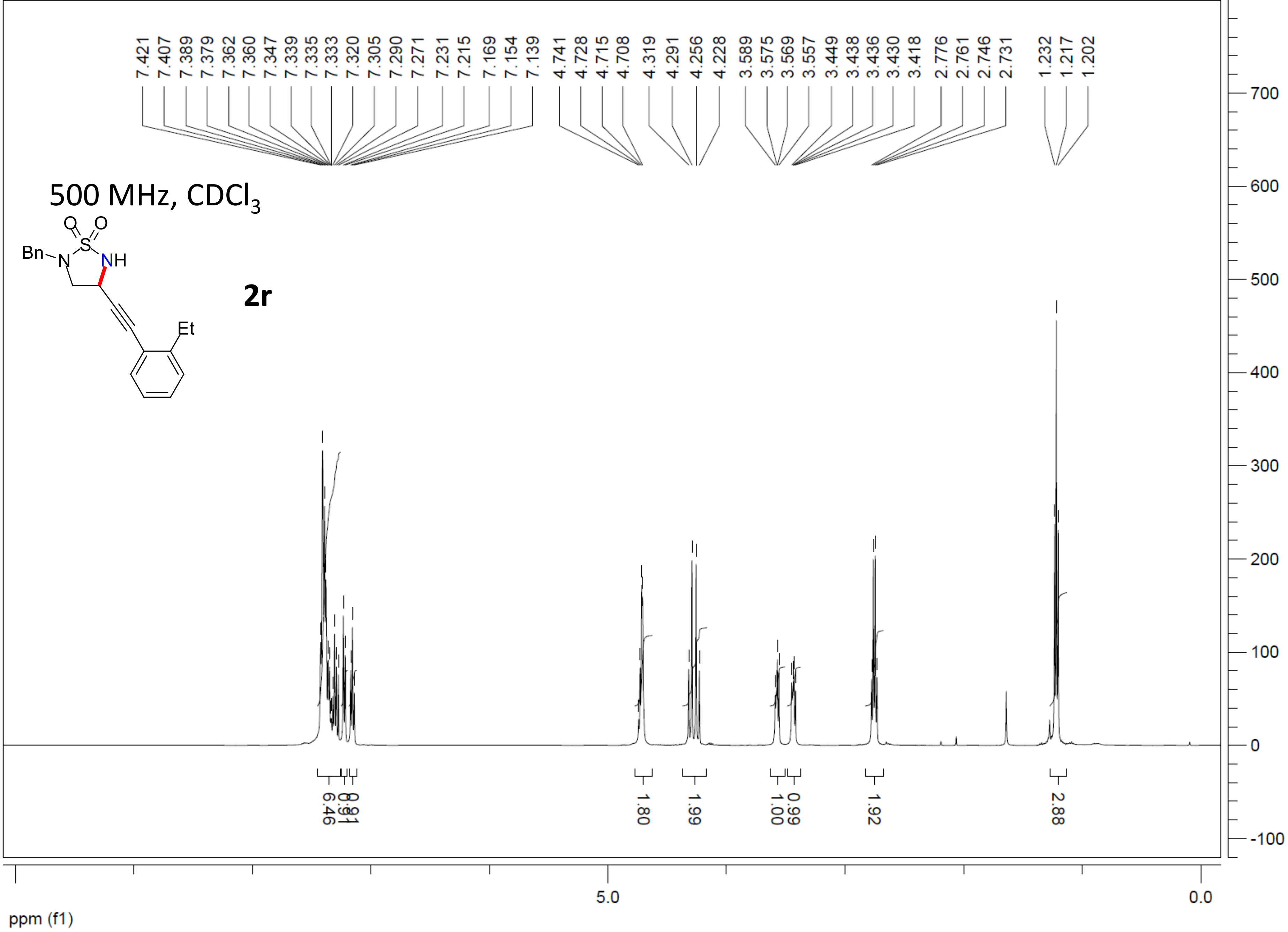
7.421 7.407 7.389 7.379 7.362 7.360 7.347 7.339 7.335 7.333 7.320 7.305 7.290 7.271 7.231 7.215 7.169 7.154 7.139
4.741 4.728 4.715 4.708
4.319 4.291 4.256 4.228
3.589 3.575 3.569 3.557 3.449 3.438 3.436 3.430 3.418
2.776 2.761 2.746 2.731
1.232 1.217 1.202
500 MHz, CDCl3
Bn
N
S
O
O
NH
Et
2r
6.46 0.91 0.91
1.80
1.99
1.00 0.99
1.92
2.88
700
600
500
400
300
200
100
0
-100
5.0
0.0
ppm (f1)

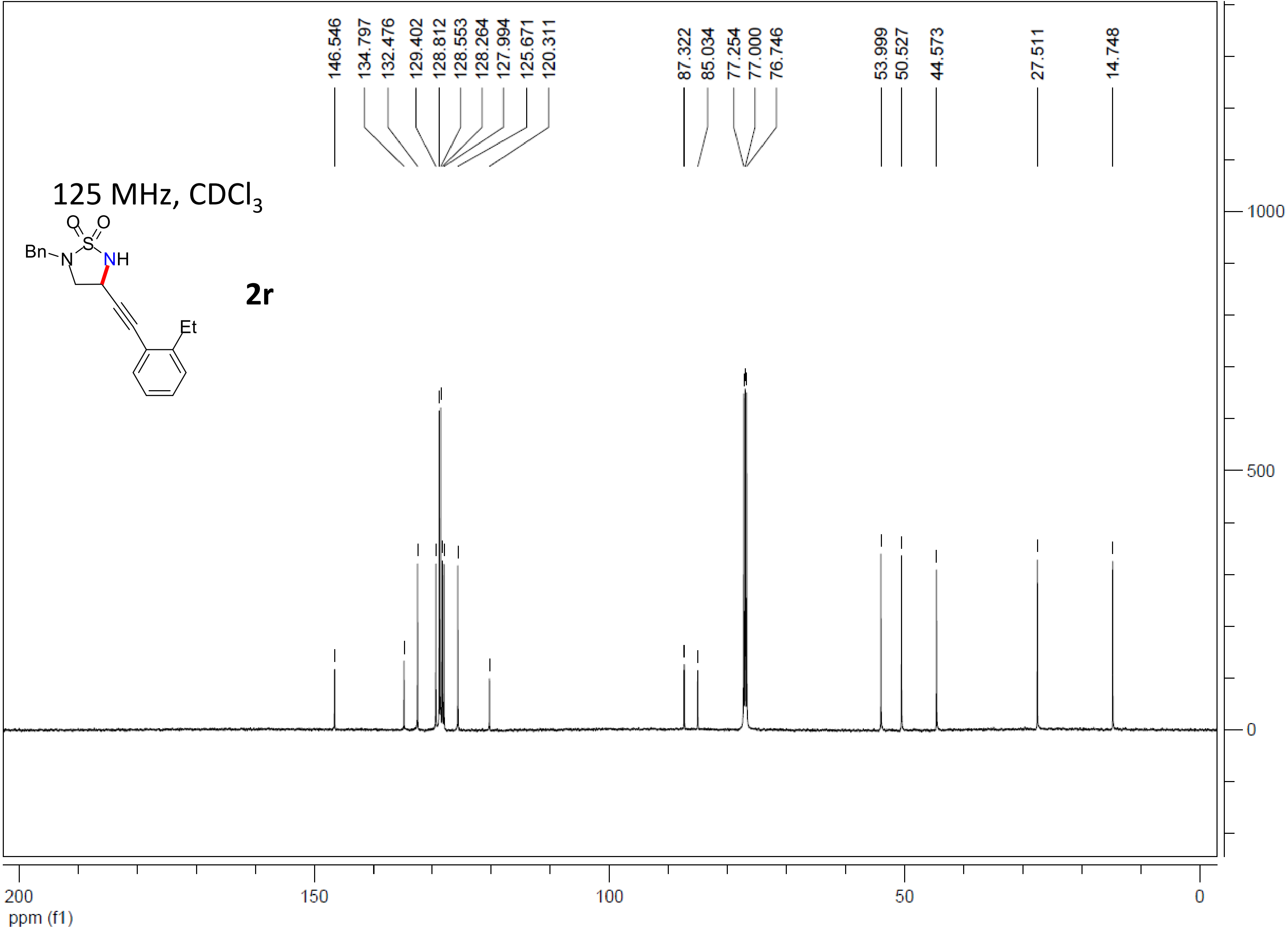
146.546
134.797
132.476
129.402
128.812
128.553
128.264
127.994
125.671
120.311
87.322
85.034
77.254
77.000
76.746
53.999
50.527
44.573
27.511
14.748
125 MHz, CDCl3
Bn
N
S
O
O
NH
Et
2r
1000
500
0
200
150
100
50
0
ppm (f1)

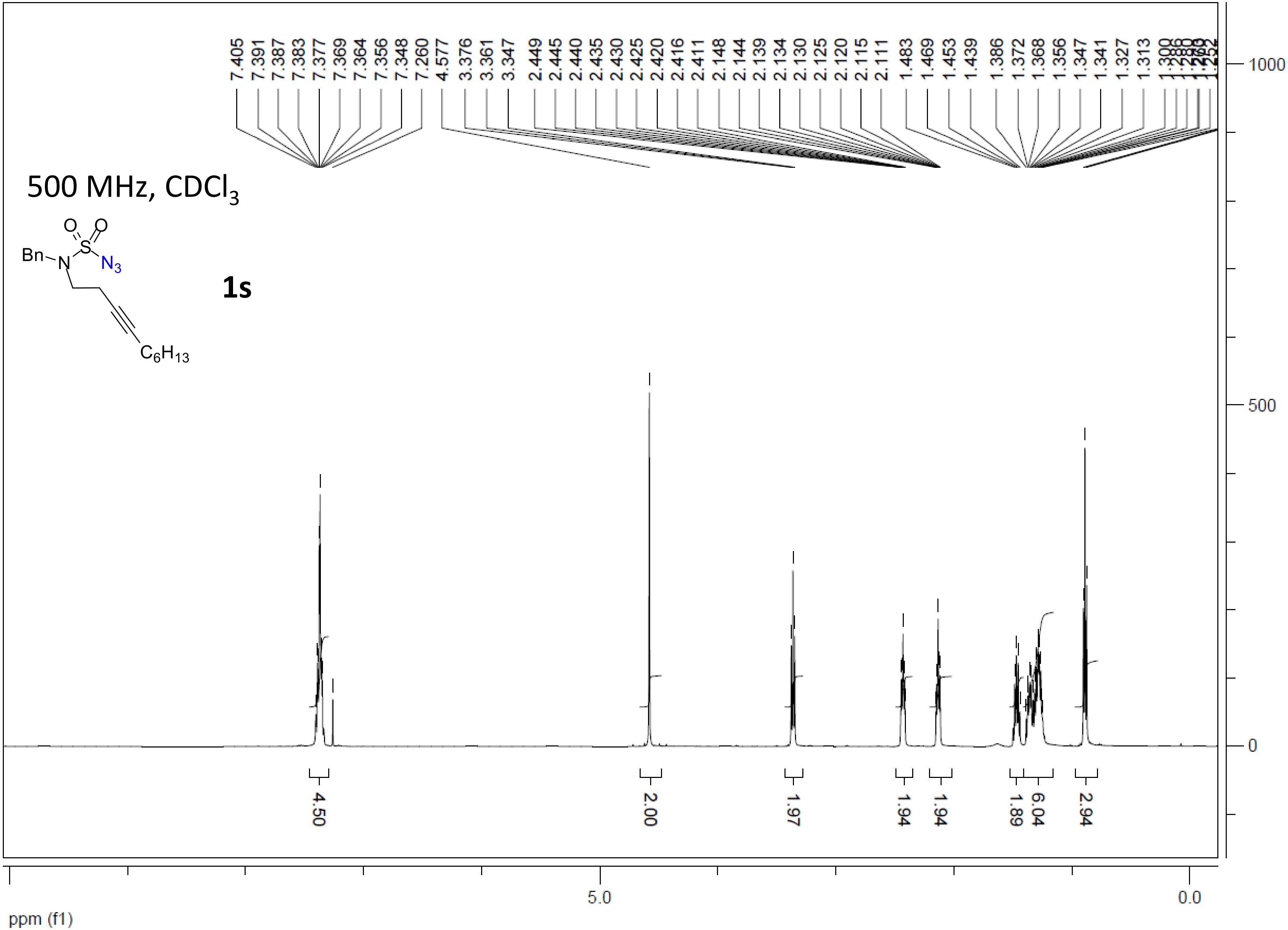
500 MHz, CDCl3
Bn
N
S
O
O
N3
C6H13
1s
7.405
7.391
7.387
7.383
7.377
7.369
7.364
7.356
7.348
7.260
4.577
3.376
3.361
3.347
2.449
2.445
2.440
2.435
2.430
2.425
2.420
2.416
2.411
2.148
2.144
2.139
2.134
2.130
2.125
2.120
2.115
2.111
1.483
1.469
1.453
1.439
1.386
1.372
1.368
1.356
1.347
1.341
1.327
1.313
4.50
2.00
1.97
1.94
1.94
1.89
6.04
2.94
1000
500
0
5.0
0.0
ppm (f1)

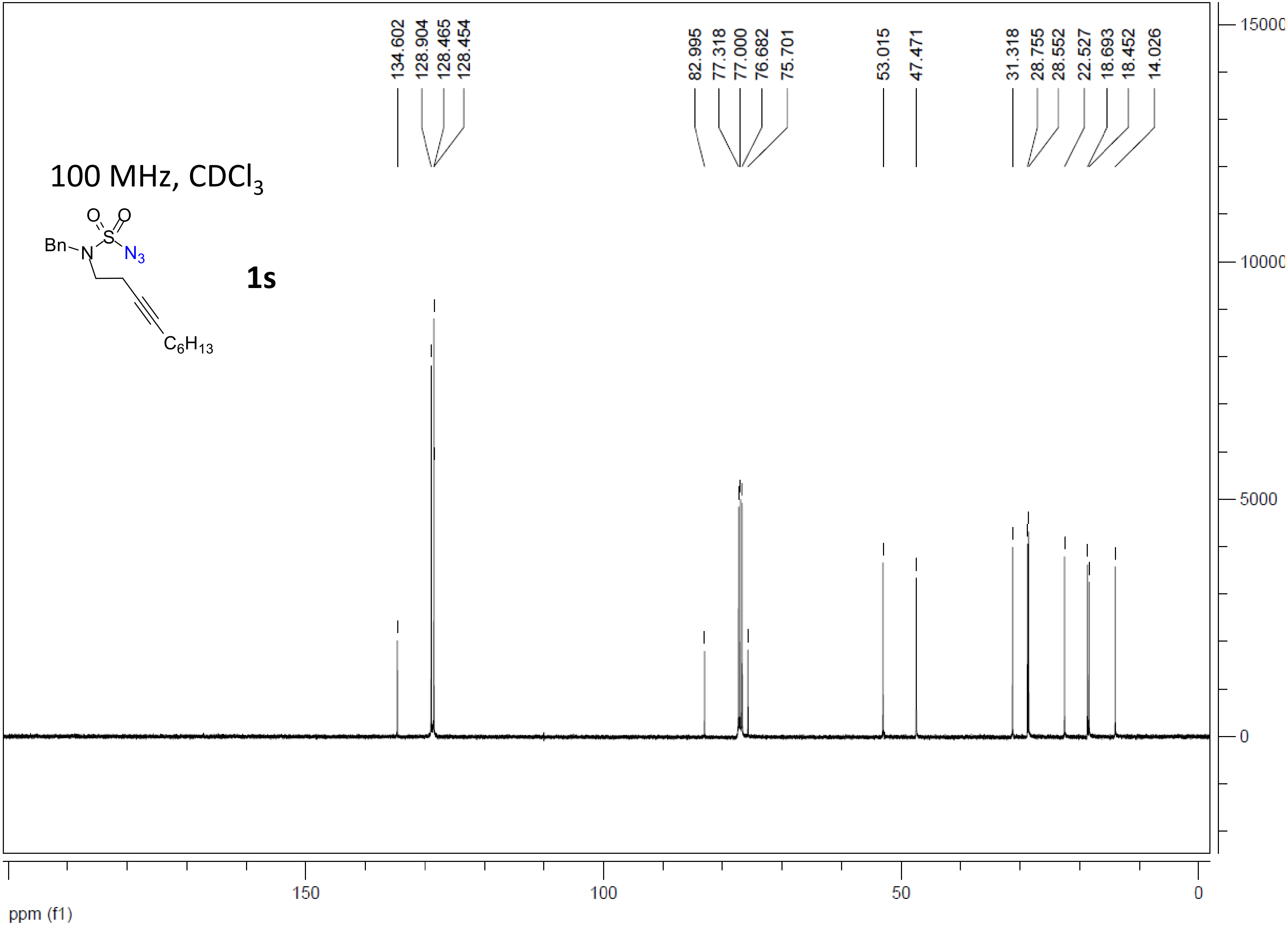
100 MHz, CDCl3
Bn
N
S
O
O
N3
C6H13
1s
134.602
128.904
128.465
128.454
82.995
77.318
77.000
76.682
75.701
53.015
47.471
31.318
28.755
28.552
22.527
18.693
18.452
14.026
15000
10000
5000
0
150
100
50
0
ppm (f1)

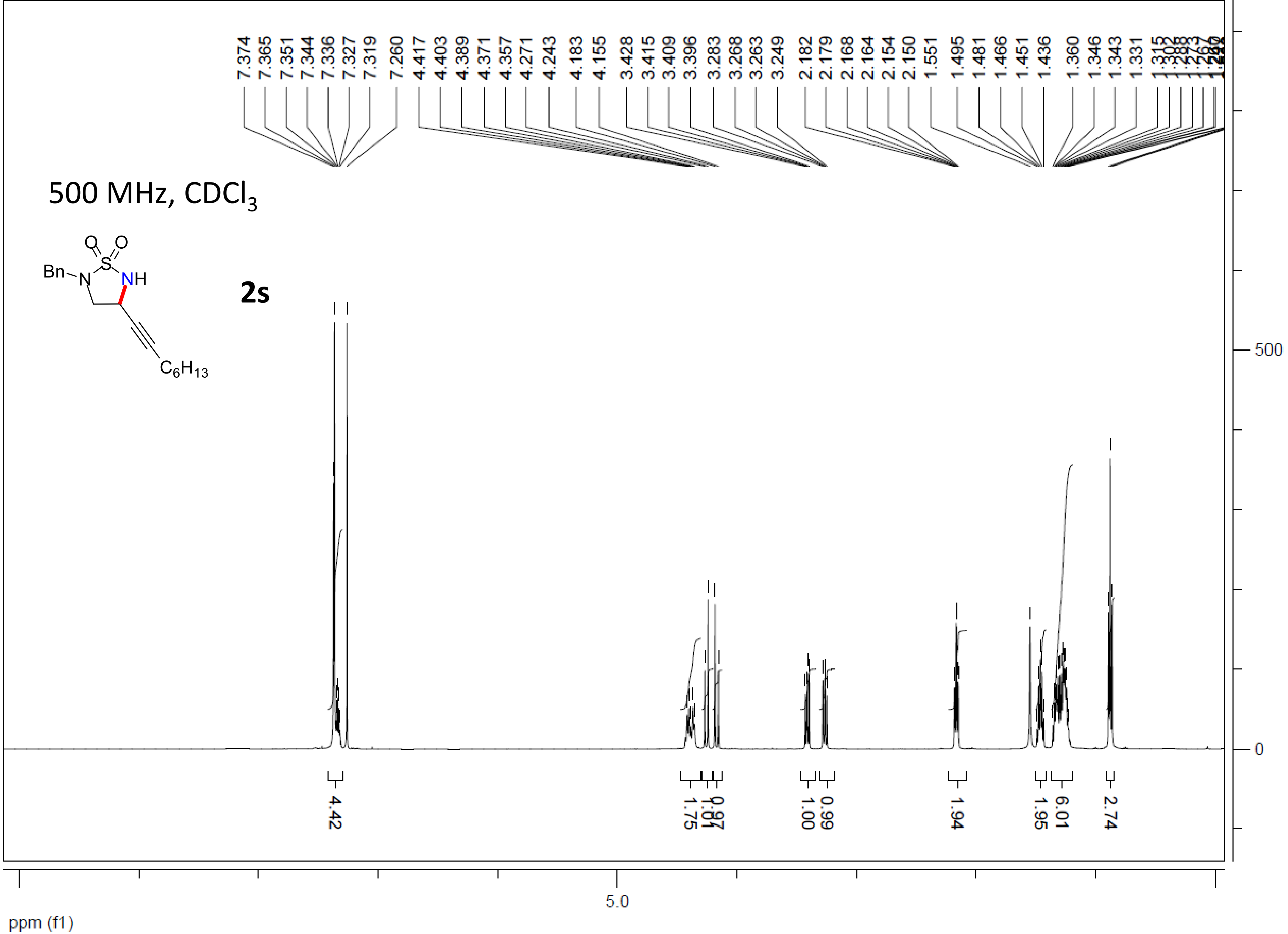
500 MHz, CDCl3
Bn
N
S
O
O
NH
C6H13
2s
7.374
7.365
7.351
7.344
7.336
7.327
7.319
7.260
4.417
4.403
4.389
4.371
4.357
4.271
4.243
4.183
4.155
3.428
3.415
3.409
3.396
3.283
3.268
3.263
3.249
2.182
2.179
2.168
2.164
2.154
2.150
1.551
1.495
1.481
1.466
1.451
1.436
1.360
1.346
1.343
1.331
1.315
4.42
1.75
1.00
0.99
1.94
1.95
6.01
2.74
500
0
5.0
ppm (f1)

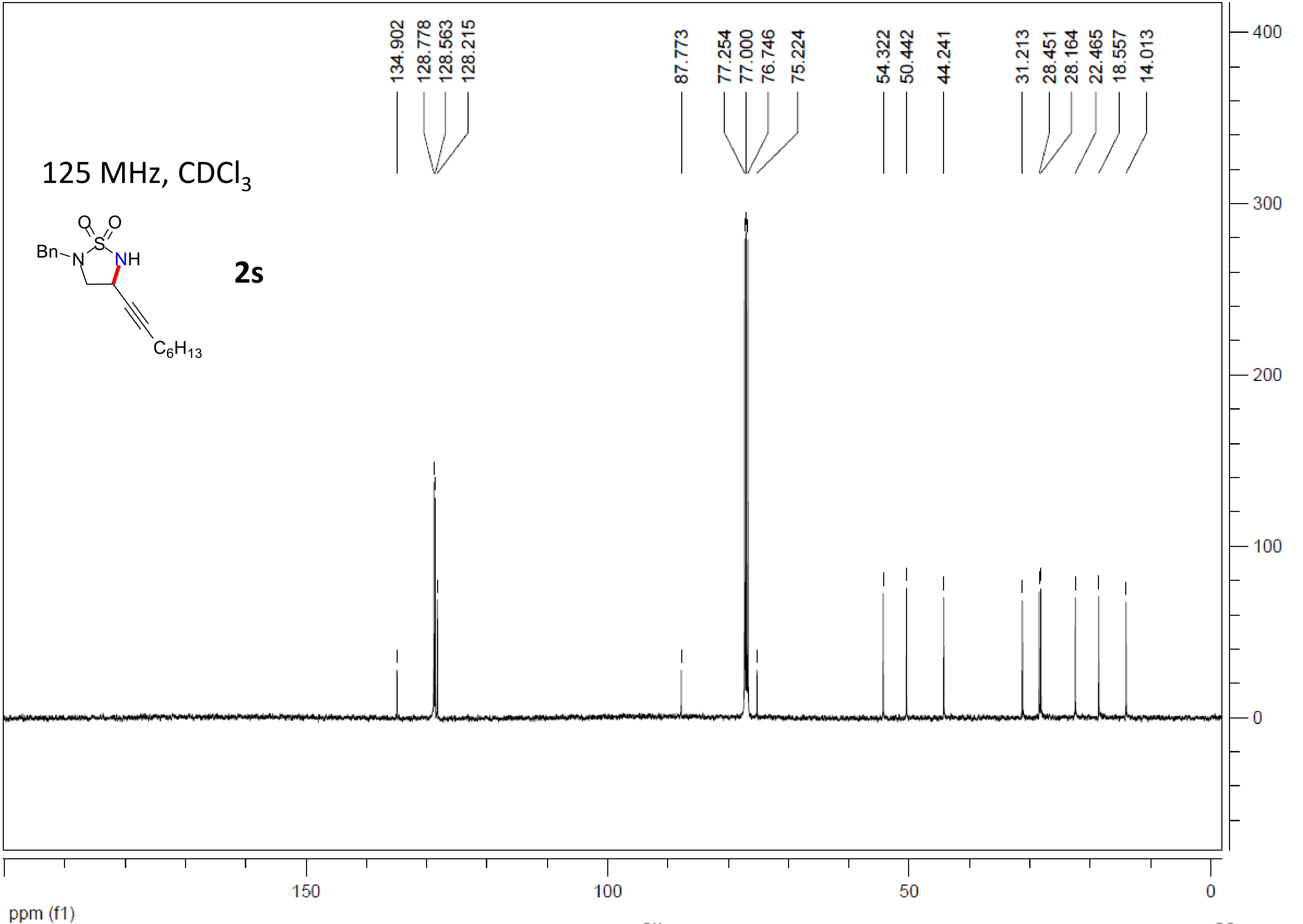
125 MHz, $CDCl_3$
2s
Bn
N
S
O
O
NH
C_6H_{13}
134.902
128.778
128.563
128.215
87.773
77.254
77.000
76.746
75.224
54.322
50.442
44.241
31.213
28.451
28.164
22.465
18.557
14.013
400
300
200
100
0
150
100
50
0
ppm (f1)

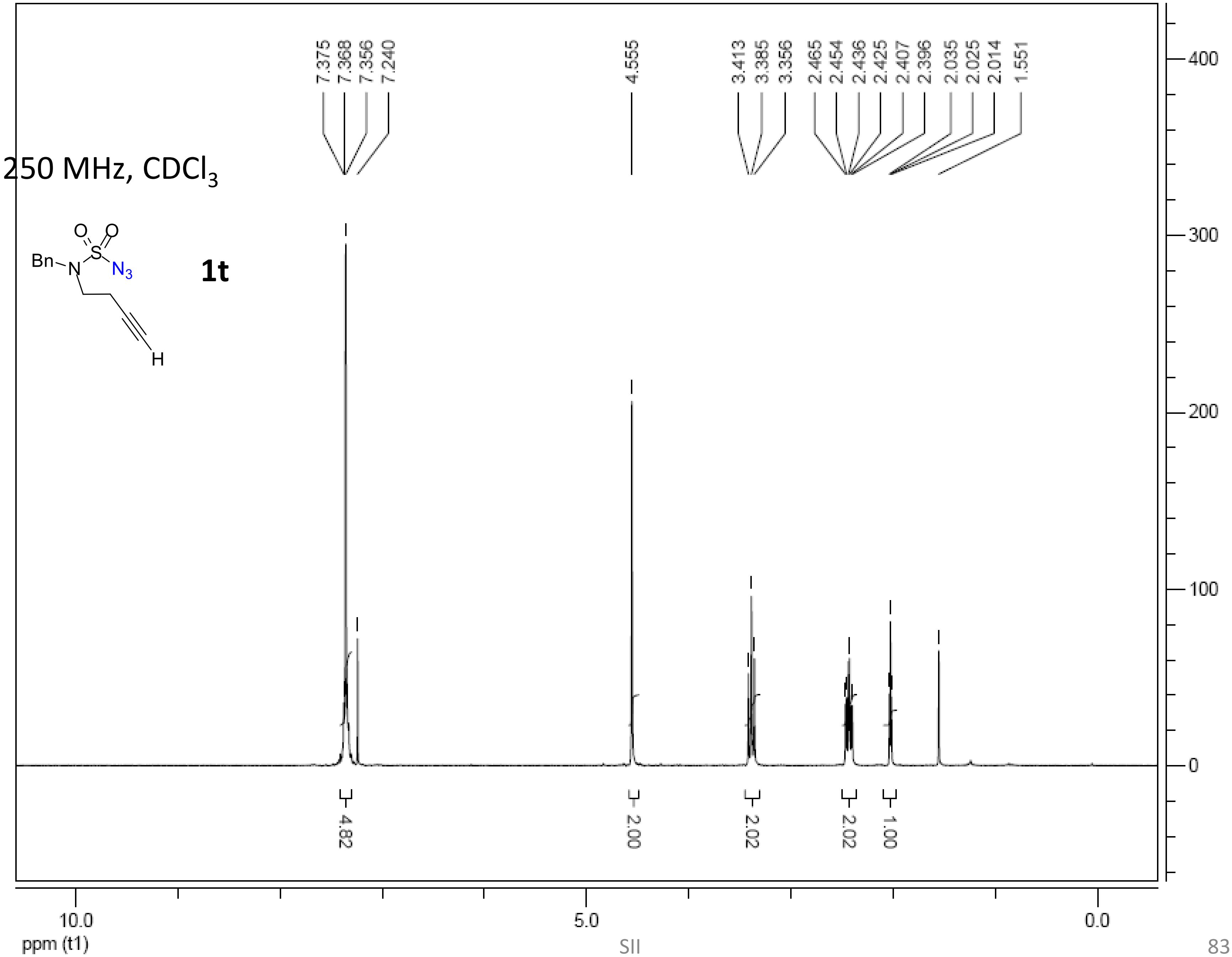
250 MHz, $CDCl_3$
1t
Bn
N
S
O
O
N_3
H
7.375 7.368 7.356 7.240
4.555
3.413 3.385 3.356 2.465 2.454 2.436 2.425 2.407 2.396 2.035 2.025 2.014 1.551
4.82
2.00
2.02
2.02
1.00
400
300
200
100
0
10.0
5.0
0.0
ppm (t1)

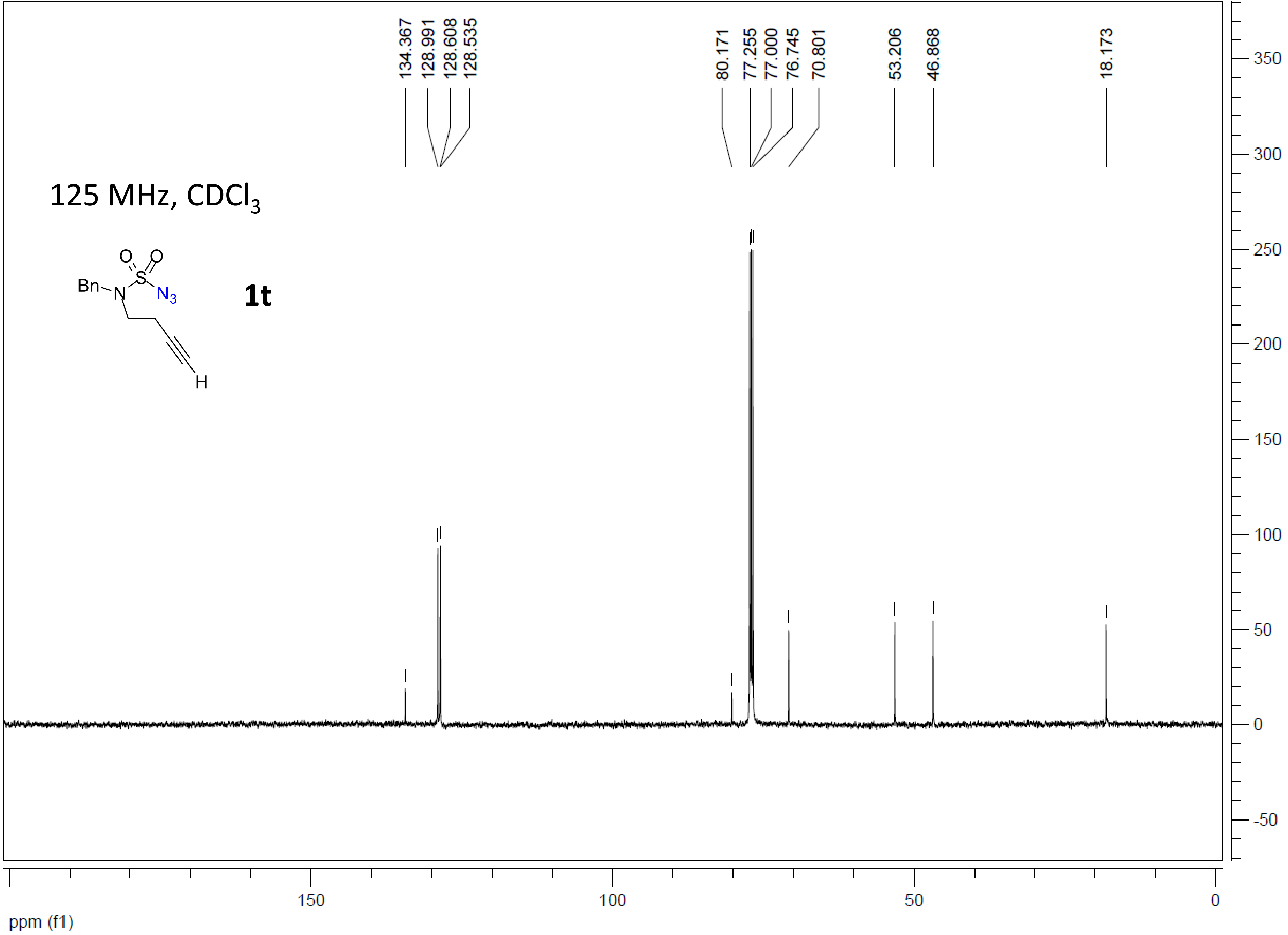
134.367
128.991
128.608
128.535
80.171
77.255
77.000
76.745
70.801
53.206
46.868
18.173
125 MHz, CDCl3
Bn
N
S
O
O
N3
H
1t
350
300
250
200
150
100
50
0
-50
150
100
50
0
ppm (f1)

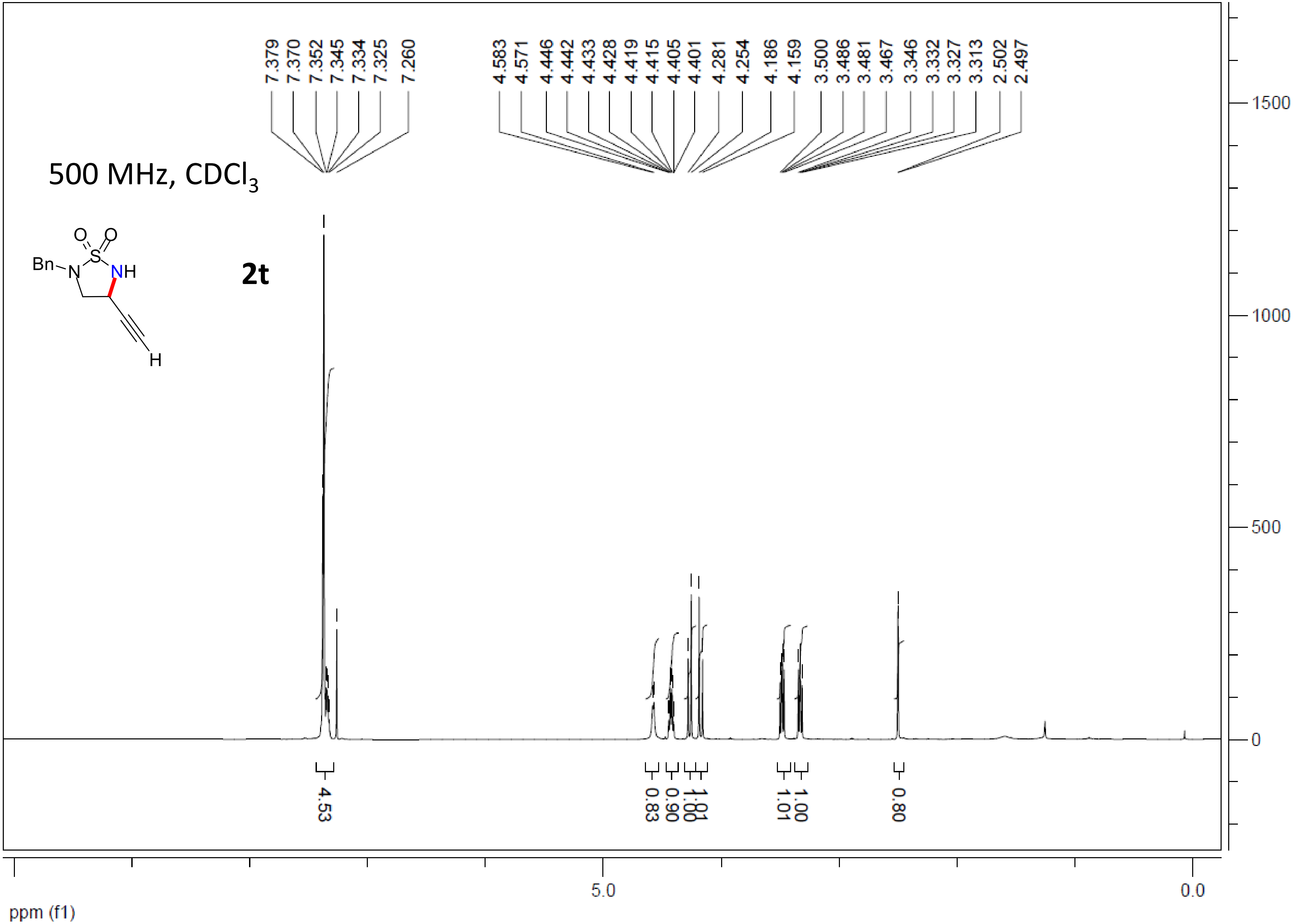
500 MHz, CDCl3
2t
Bn
N
S
O
O
NH
H
7.379
7.370
7.352
7.345
7.334
7.325
7.260
4.583
4.571
4.446
4.442
4.433
4.428
4.419
4.415
4.405
4.401
4.281
4.254
4.186
4.159
3.500
3.486
3.481
3.467
3.346
3.332
3.327
3.313
2.502
2.497
4.53
0.83
0.90
1.80
1.01
1.01
1.00
0.80
1500
1000
500
0
5.0
0.0
ppm (f1)

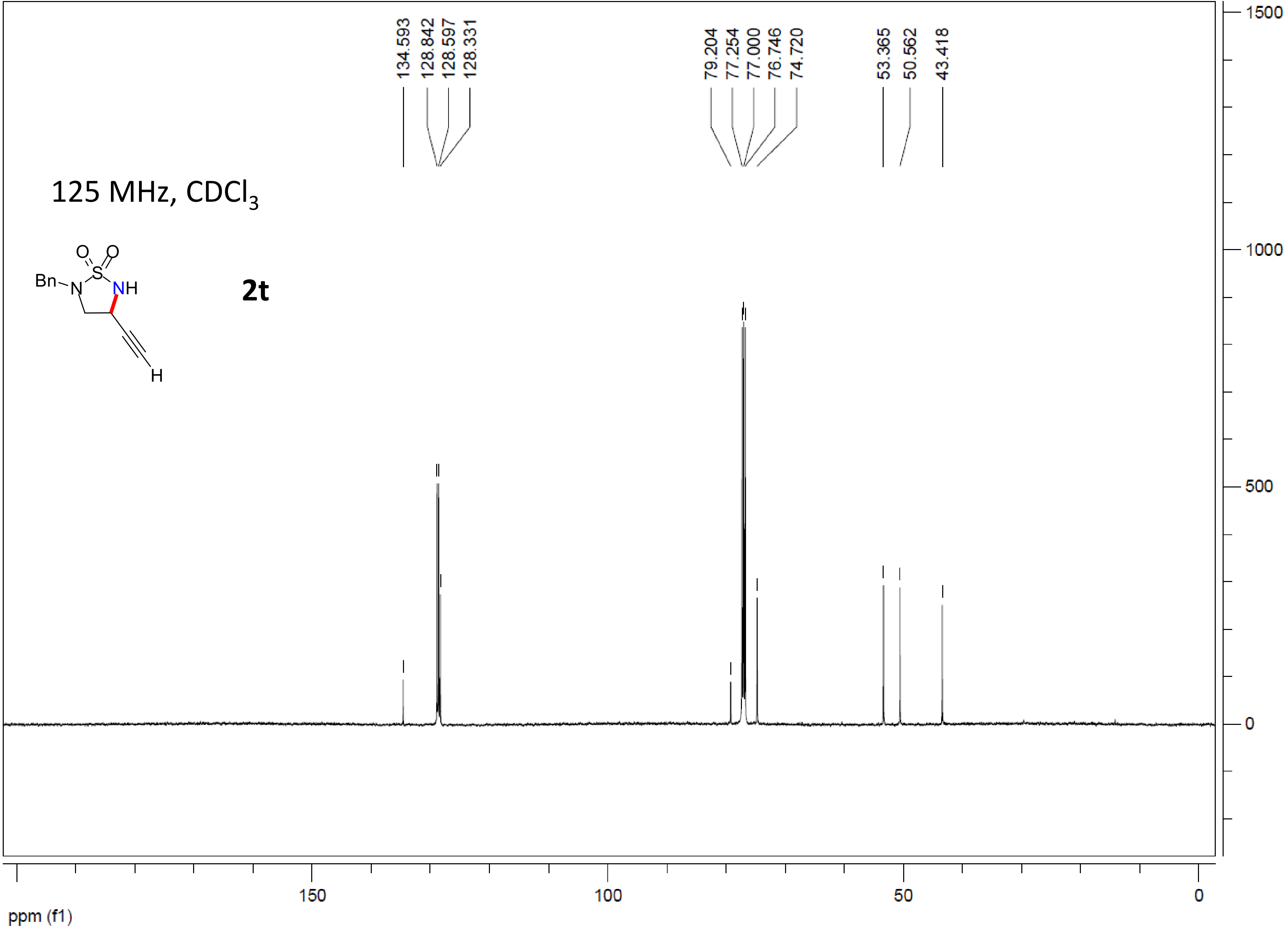
134.593
128.842
128.597
128.331
79.204
77.254
77.000
76.746
74.720
53.365
50.562
43.418
125 MHz, CDCl3
Bn
N
S
O
O
NH
H
2t
1500
1000
500
0
150
100
50
0
ppm (f1)

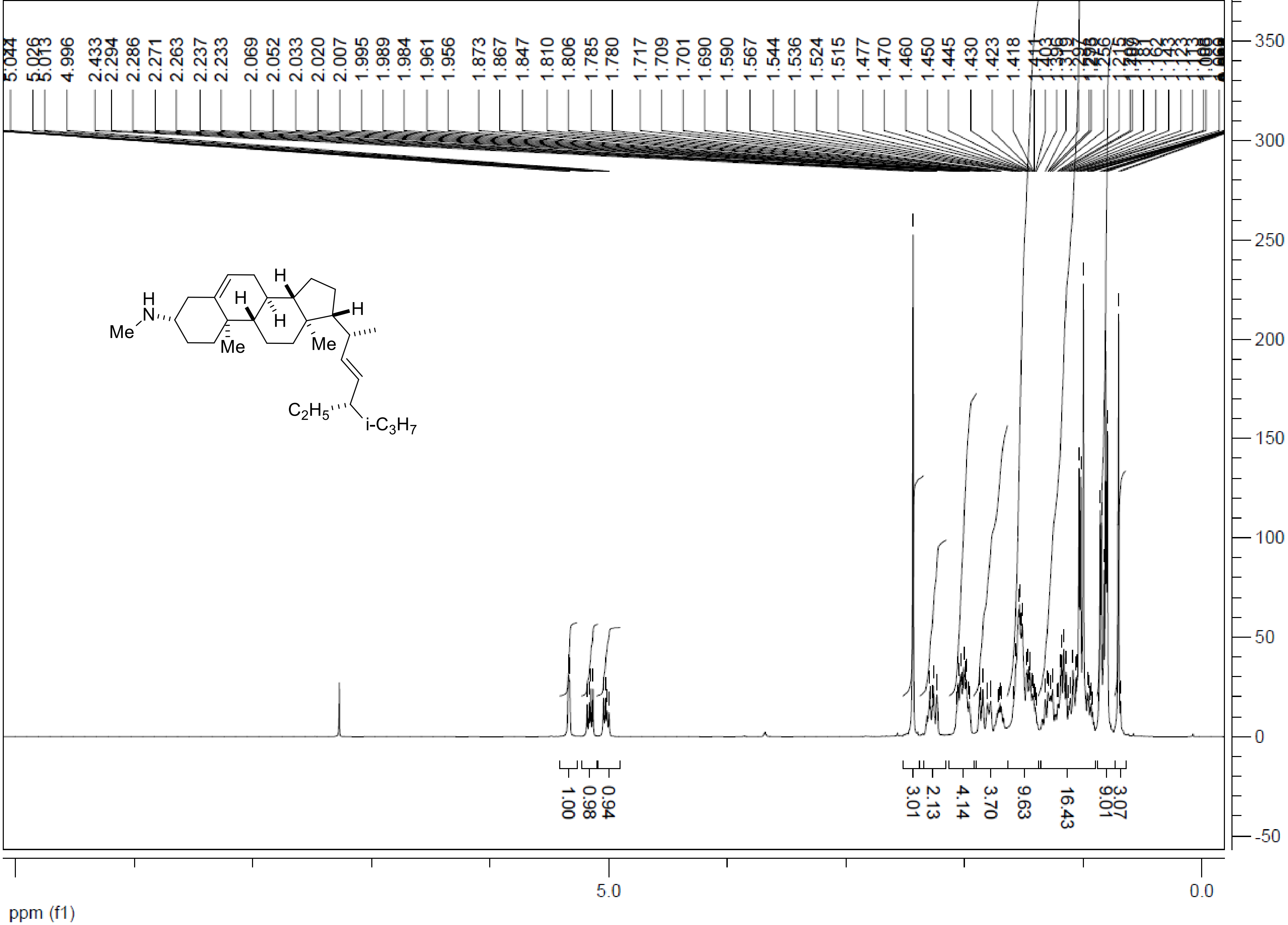
5.026
5.013
4.996
2.433
2.294
2.286
2.271
2.263
2.237
2.233
2.069
2.052
2.033
2.020
2.007
1.995
1.989
1.984
1.961
1.956
1.873
1.867
1.847
1.810
1.806
1.785
1.780
1.717
1.709
1.701
1.690
1.590
1.567
1.544
1.536
1.524
1.515
1.477
1.470
1.460
1.450
1.445
1.430
1.423
1.418
H
N
Me
H
H
H
H
Me
Me
C_2H_5
i-C_3H_7
1.00
0.98
0.94
3.01
2.13
4.14
3.70
9.63
16.43
9.01
3.07
350
300
250
200
150
100
50
0
-50
5.0
0.0
ppm (f1)

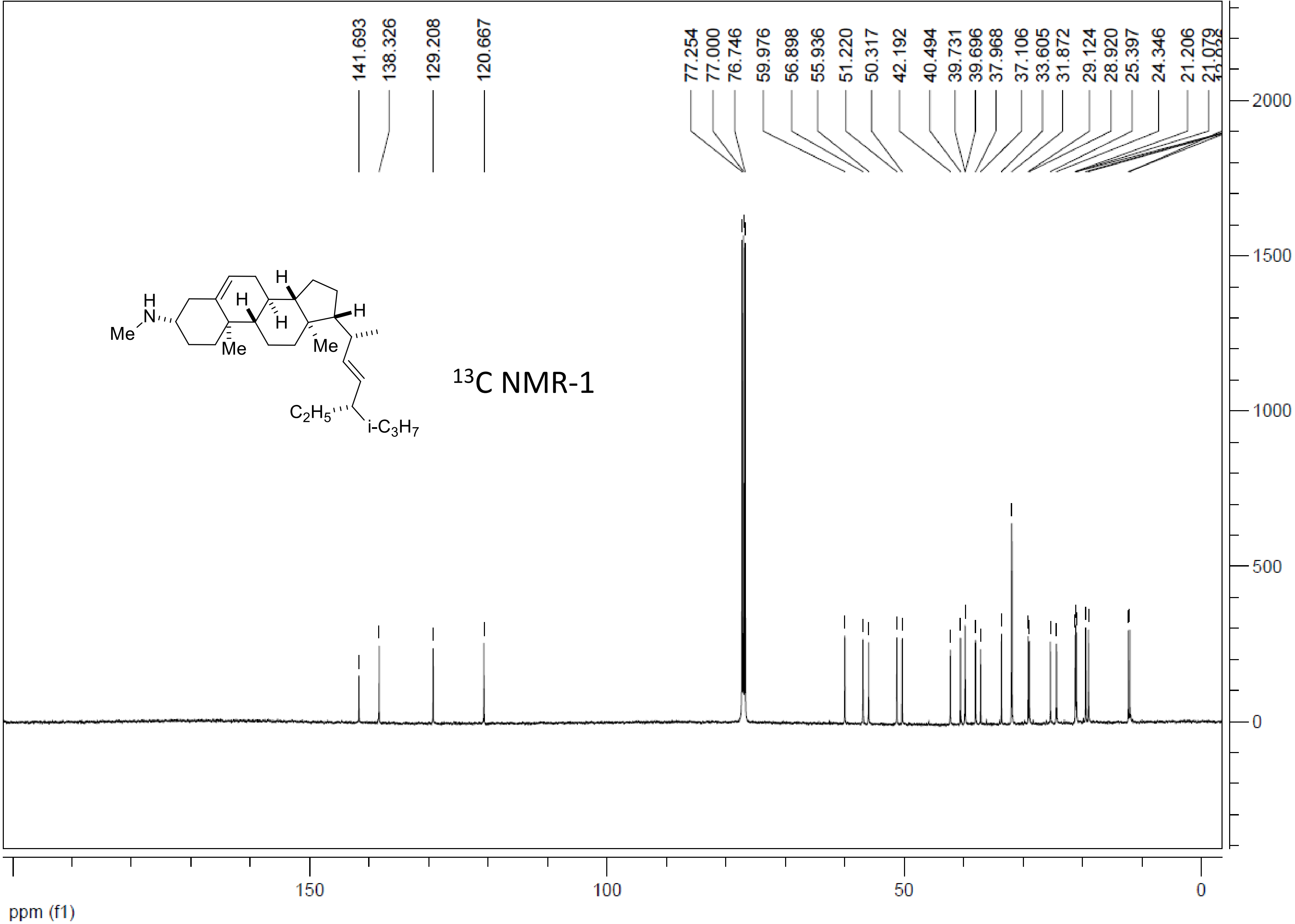
141.693
138.326
129.208
120.667
77.254
77.000
76.746
59.976
56.898
55.936
51.220
50.317
42.192
40.494
39.731
39.696
37.968
37.106
33.605
31.872
29.124
28.920
25.397
24.346
21.206
21.079
2000
1500
1000
500
0
150
100
50
0
ppm (f1)
H
N
Me
Me
Me
H
H
H
H
C_2H_5
i-C_3H_7
^{13}C NMR-1

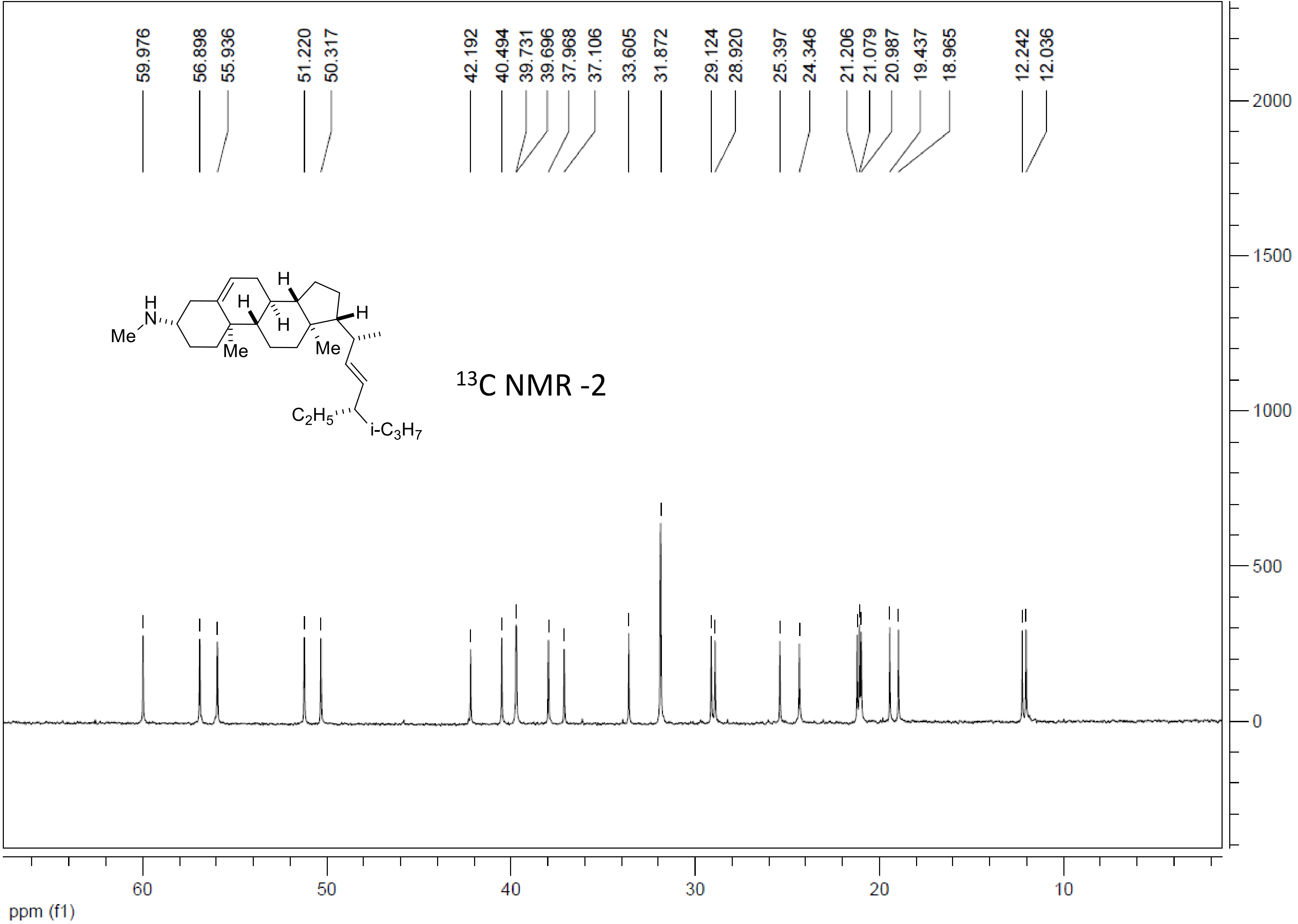
59.976
56.898
55.936
51.220
50.317
42.192
40.494
39.731
39.696
37.968
37.106
33.605
31.872
29.124
28.920
25.397
24.346
21.206
21.079
20.987
19.437
18.965
12.242
12.036
H
N
Me
H
H
H
Me
Me
H
C_2H_5
i-C_3H_7
^{13}C NMR -2
2000
1500
1000
500
0
60
50
40
30
20
10
ppm (f1)

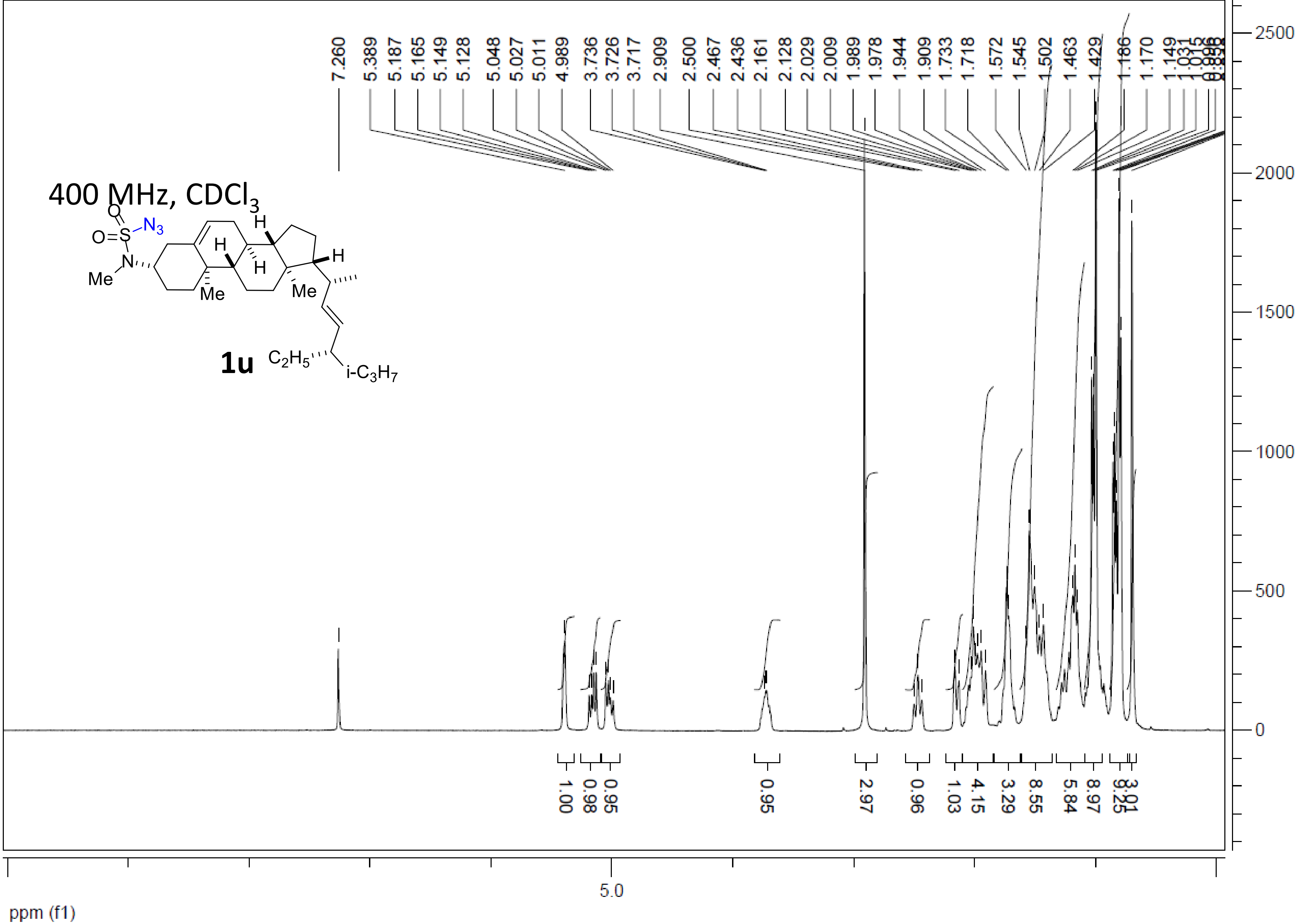
400 MHz, CDCl3
1u
7.260
5.389
5.187
5.165
5.149
5.128
5.048
5.027
5.011
4.989
3.736
3.726
3.717
2.909
2.500
2.467
2.436
2.161
2.128
2.029
2.009
1.989
1.978
1.944
1.909
1.733
1.718
1.572
1.545
1.502
1.463
1.429
1.186
1.170
1.149
1.031
1.015
1.00
0.98
0.95
0.95
2.97
0.96
1.03
4.15
3.29
8.55
5.84
8.97
3.01
2500
2000
1500
1000
500
0
5.0
ppm (f1)

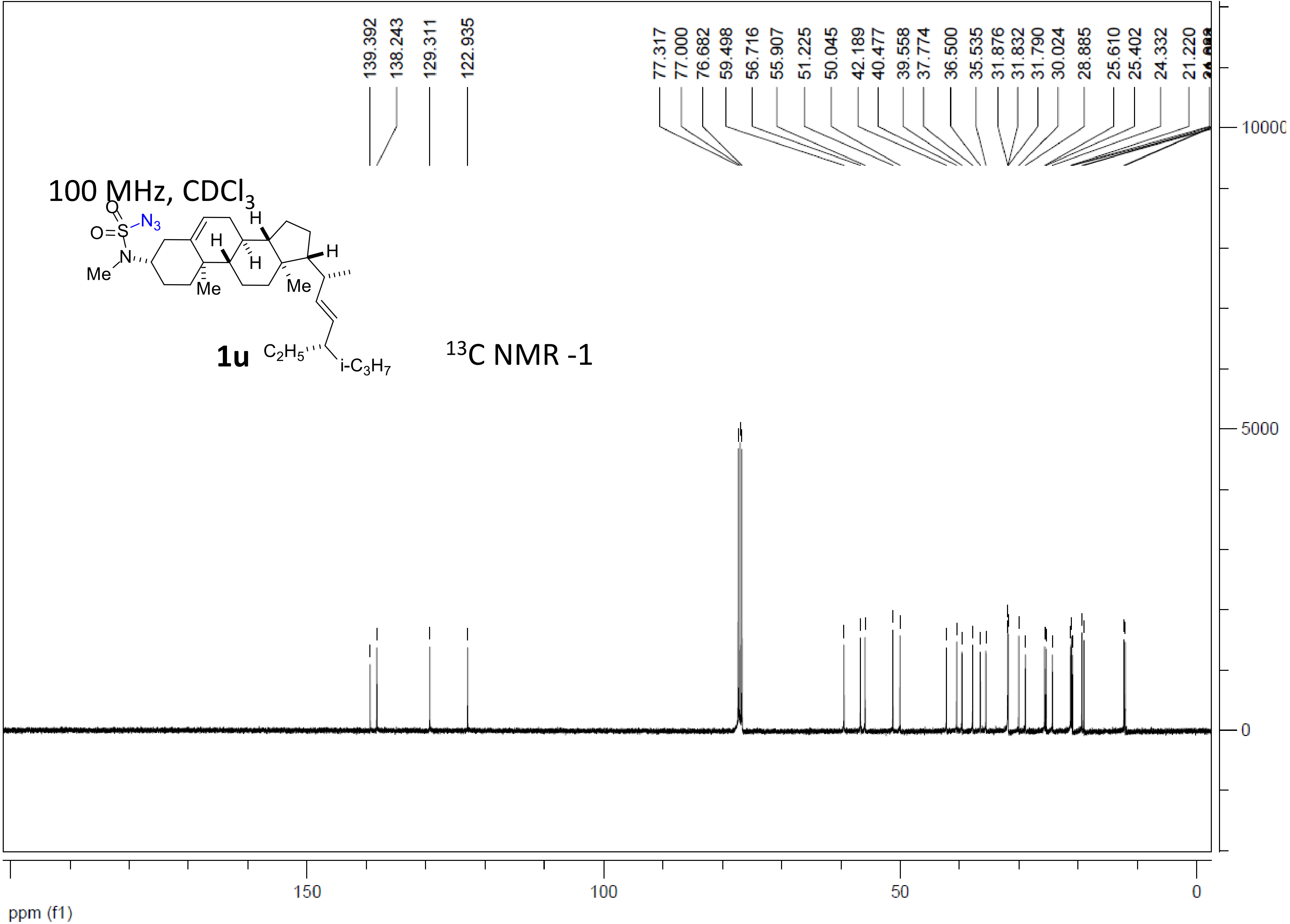
139.392
138.243
129.311
122.935
77.317
77.000
76.682
59.498
56.716
55.907
51.225
50.045
42.189
40.477
39.558
37.774
36.500
35.535
31.876
31.832
31.790
30.024
28.885
25.610
25.402
24.332
21.220
10000
5000
0
100 MHz, $CDCl_3$
O
O=S
N_3
N
Me
H
H
H
H
Me
Me
1u
C_2H_5
i-C_3H_7
^{13}C NMR -1
150
100
50
0
ppm (f1)

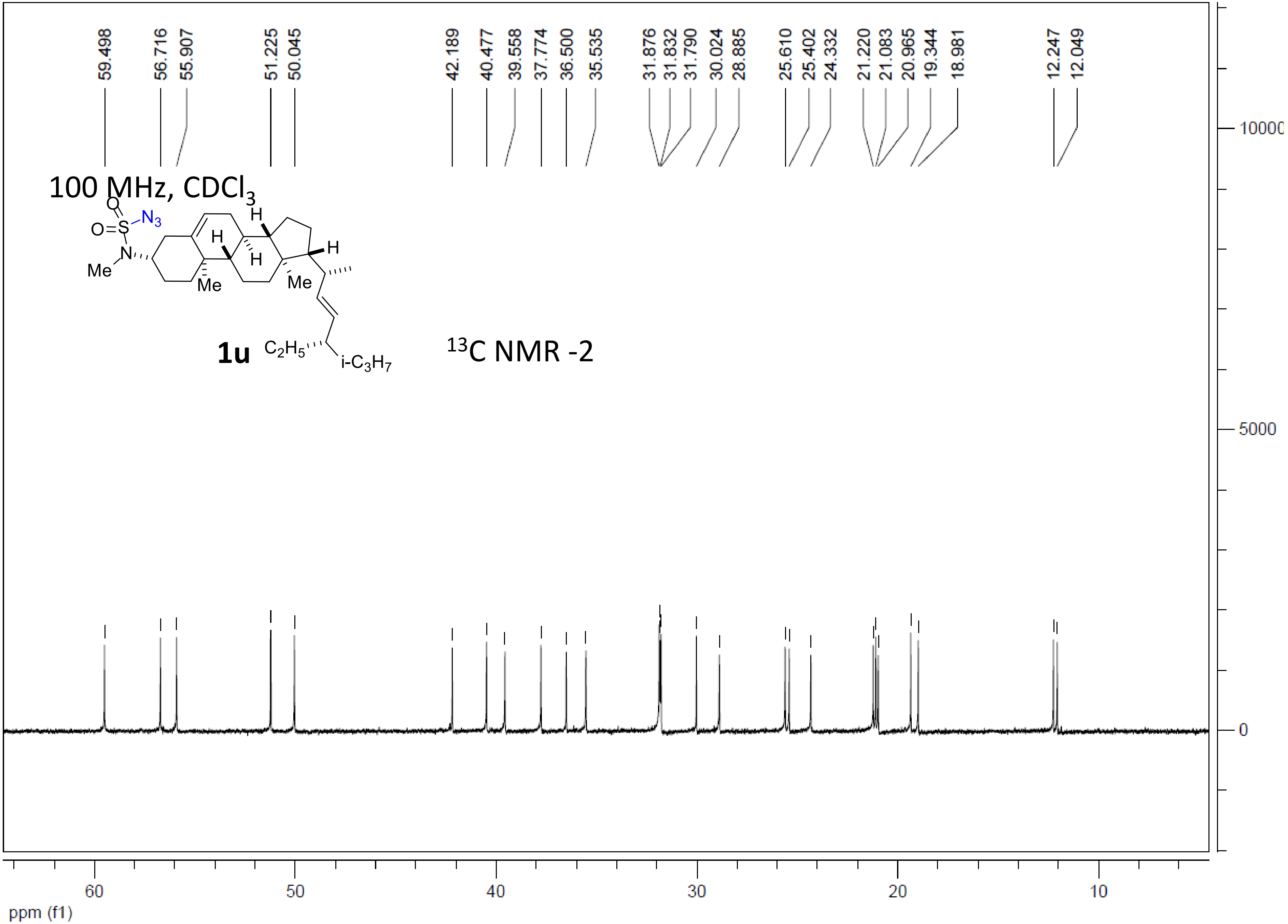
59.498
56.716
55.907
51.225
50.045
42.189
40.477
39.558
37.774
36.500
35.535
31.876
31.832
31.790
30.024
28.885
25.610
25.402
24.332
21.220
21.083
20.965
19.344
18.981
12.247
12.049
100 MHz, CDCl3
1u
13C NMR -2
10000
5000
0
60
50
40
30
20
10
ppm (f1)

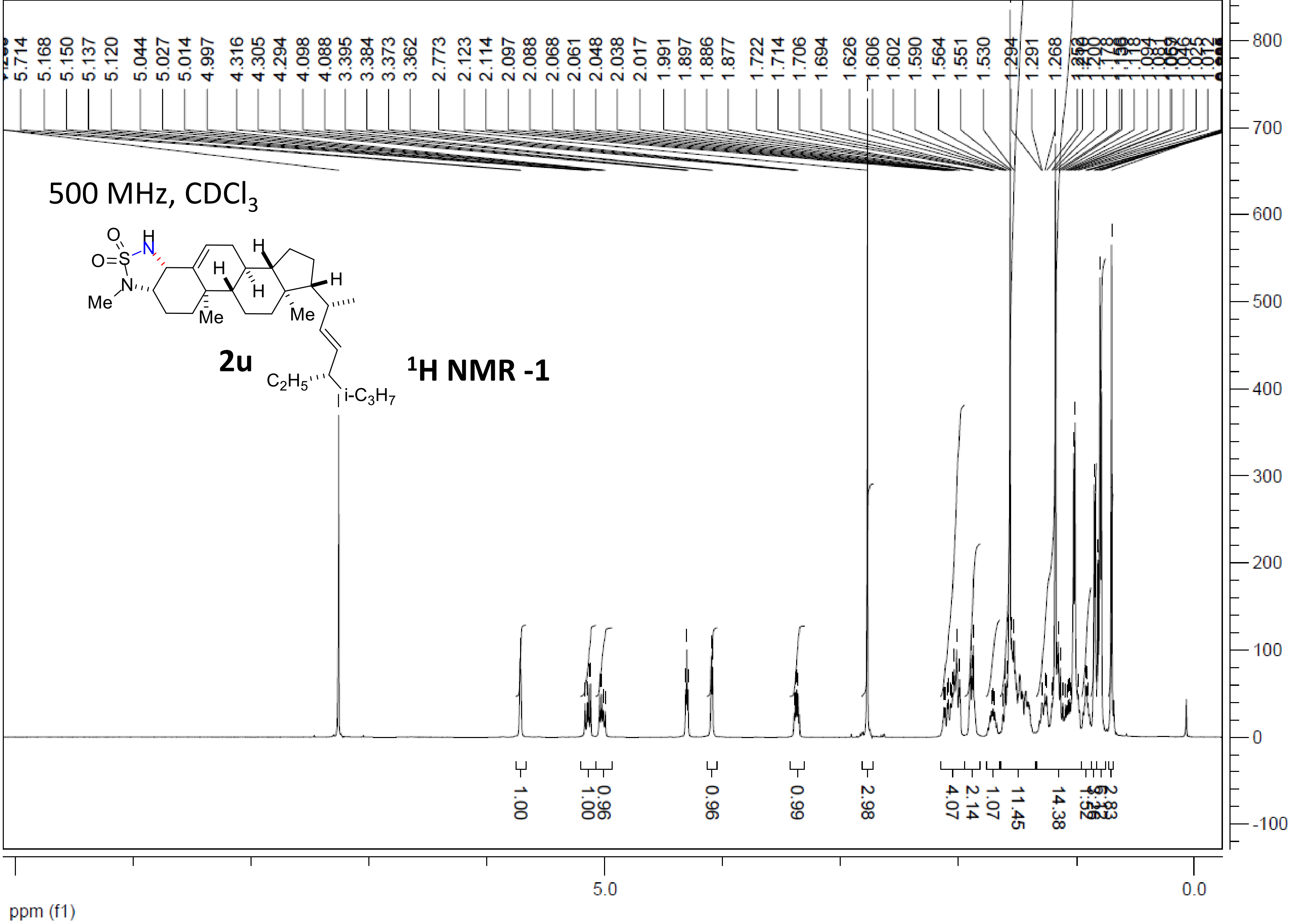
500 MHz, CDCl3
2u
1H NMR -1
O=S, N, H, Me, H, Me, Me, H, H, H, C2H5, i-C3H7
5.714 5.168 5.150 5.137 5.120 5.044 5.027 5.014 4.997 4.316 4.305 4.294 4.098 4.088 3.395 3.384 3.373 3.362 2.773 2.123 2.114 2.097 2.088 2.068 2.061 2.048 2.038 2.017 1.991 1.897 1.886 1.877 1.722 1.714 1.706 1.694 1.626 1.606 1.602 1.590 1.564 1.551 1.530 1.294 1.291 1.268
1.00 1.00 0.96 0.96 0.99 2.98 4.07 2.14 1.07 11.45 14.38 2.83
800 700 600 500 400 300 200 100 0 -100
5.0 0.0
ppm (f1)

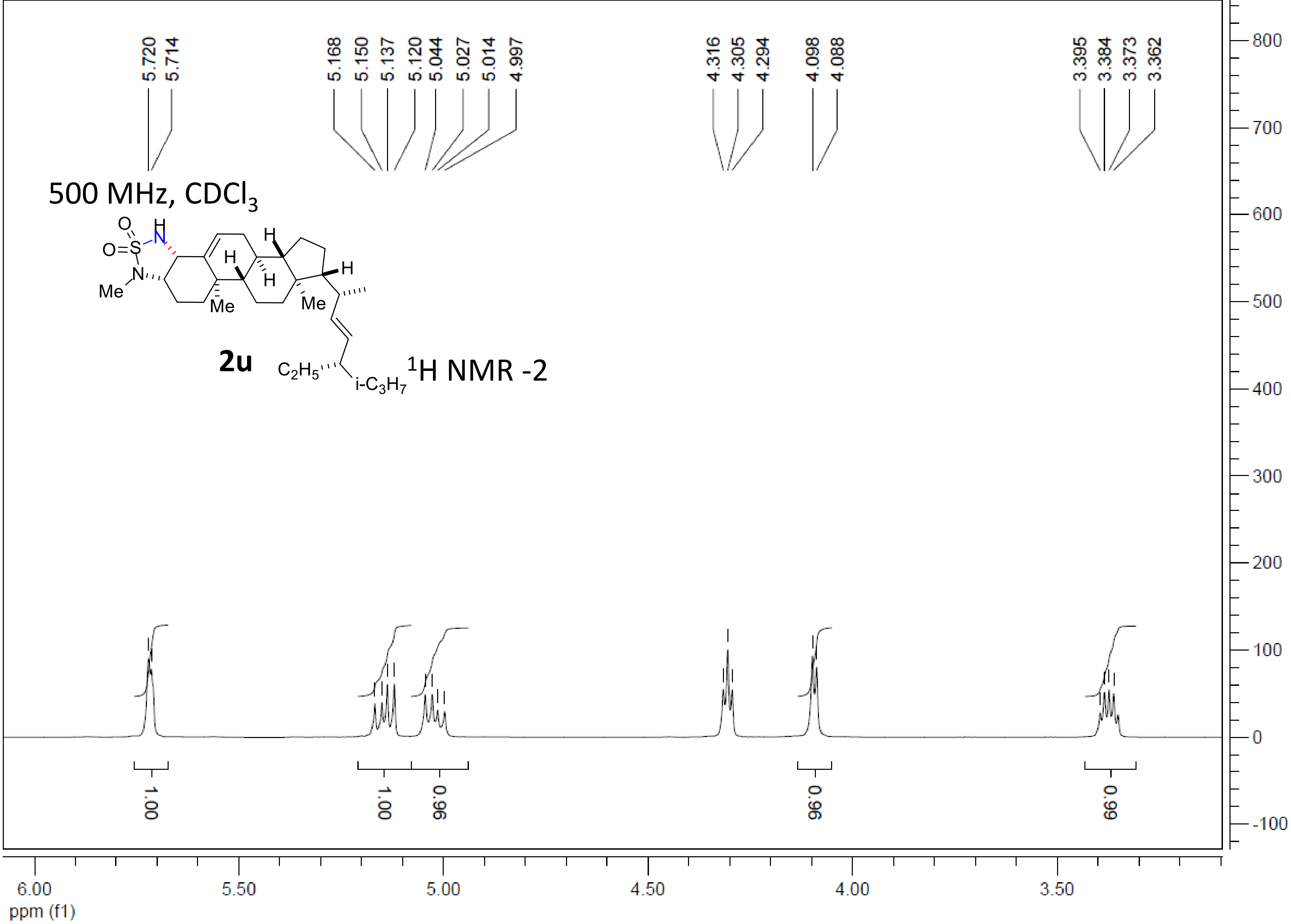
5.720
5.714
5.168
5.150
5.137
5.120
5.044
5.027
5.014
4.997
4.316
4.305
4.294
4.098
4.088
3.395
3.384
3.373
3.362
500 MHz, CDCl3
O
H
N
O=S
N
Me
H
H
H
H
Me
Me
2u
C2H5
i-C3H7
1H NMR -2
1.00
1.00
0.96
0.96
0.99
800
700
600
500
400
300
200
100
0
-100
6.00
5.50
5.00
4.50
4.00
3.50
ppm (f1)

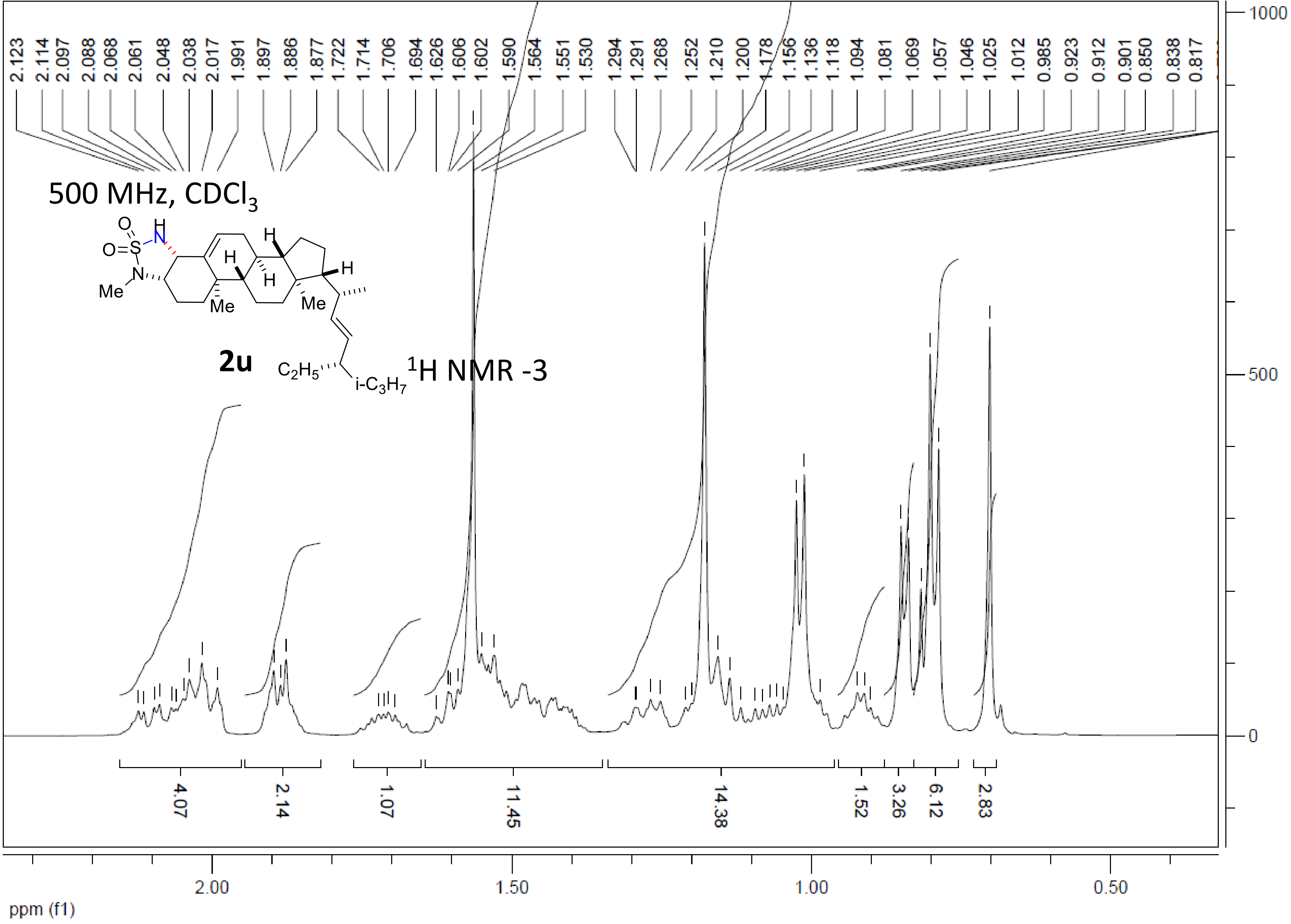
500 MHz, CDCl3
2u
1H NMR -3
O=S N H Me
H H H H Me Me
C2H5 i-C3H7
2.123 2.114 2.097 2.088 2.068 2.061 2.048 2.038 2.017 1.991 1.897 1.886 1.877 1.722 1.714 1.706 1.694 1.626 1.606 1.602 1.590 1.564 1.551 1.530 1.294 1.291 1.268 1.252 1.210 1.200 1.178 1.156 1.136 1.118 1.094 1.081 1.069 1.057 1.046 1.025 1.012 0.985 0.923 0.912 0.901 0.850 0.838 0.817
4.07 2.14 1.07 11.45 14.38 1.52 3.26 6.12 2.83
2.00 1.50 1.00 0.50
ppm (f1)
1000 500 0

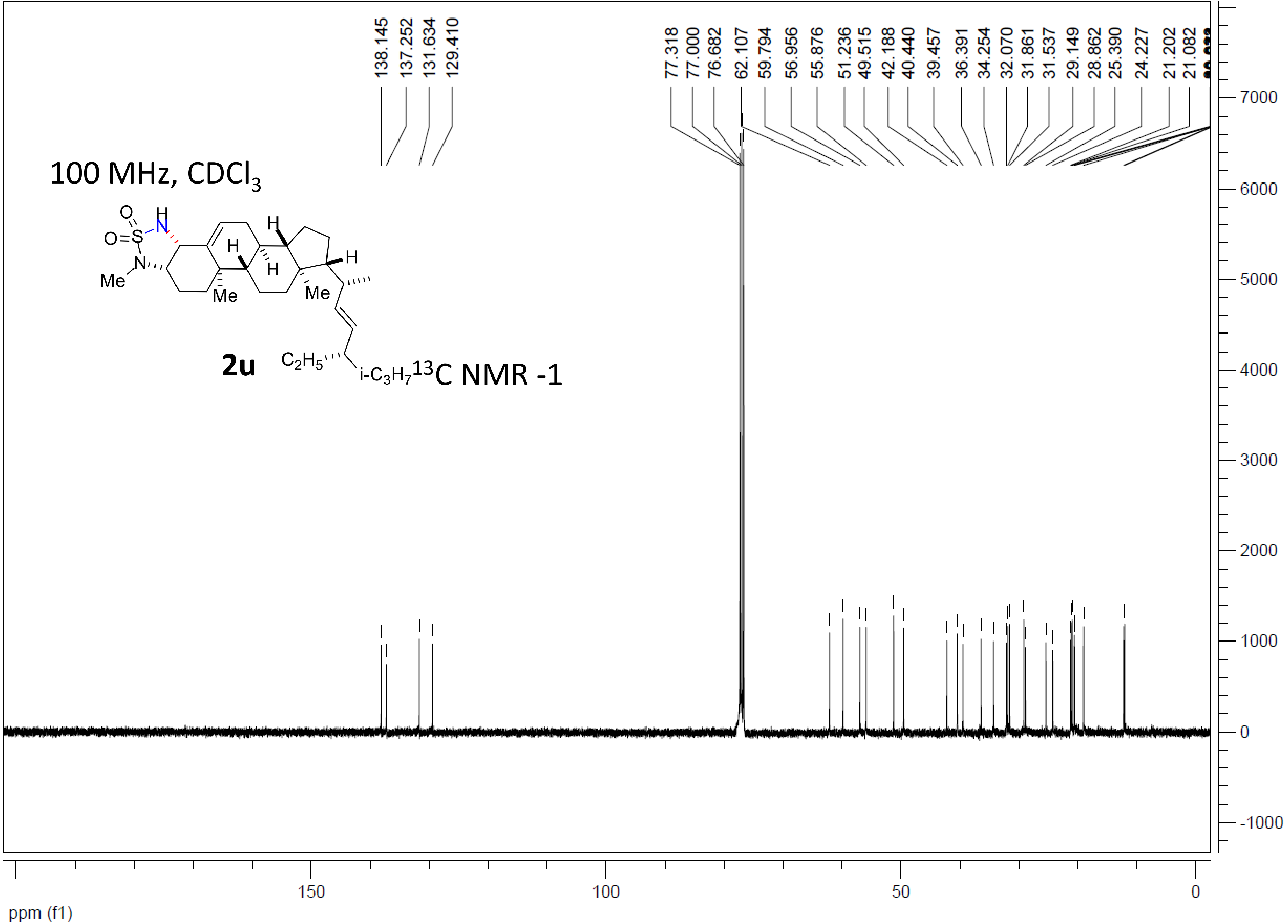

100 MHz, $CDCl_3$
2u
^{13}C NMR -1
138.145
137.252
131.634
129.410
77.318
77.000
76.682
62.107
59.794
56.956
55.876
51.236
49.515
42.188
40.440
39.457
36.391
34.254
32.070
31.861
31.537
29.149
28.862
25.390
24.227
21.202
21.082
7000
6000
5000
4000
3000
2000
1000
0
-1000
150
100
50
0
ppm (f1)

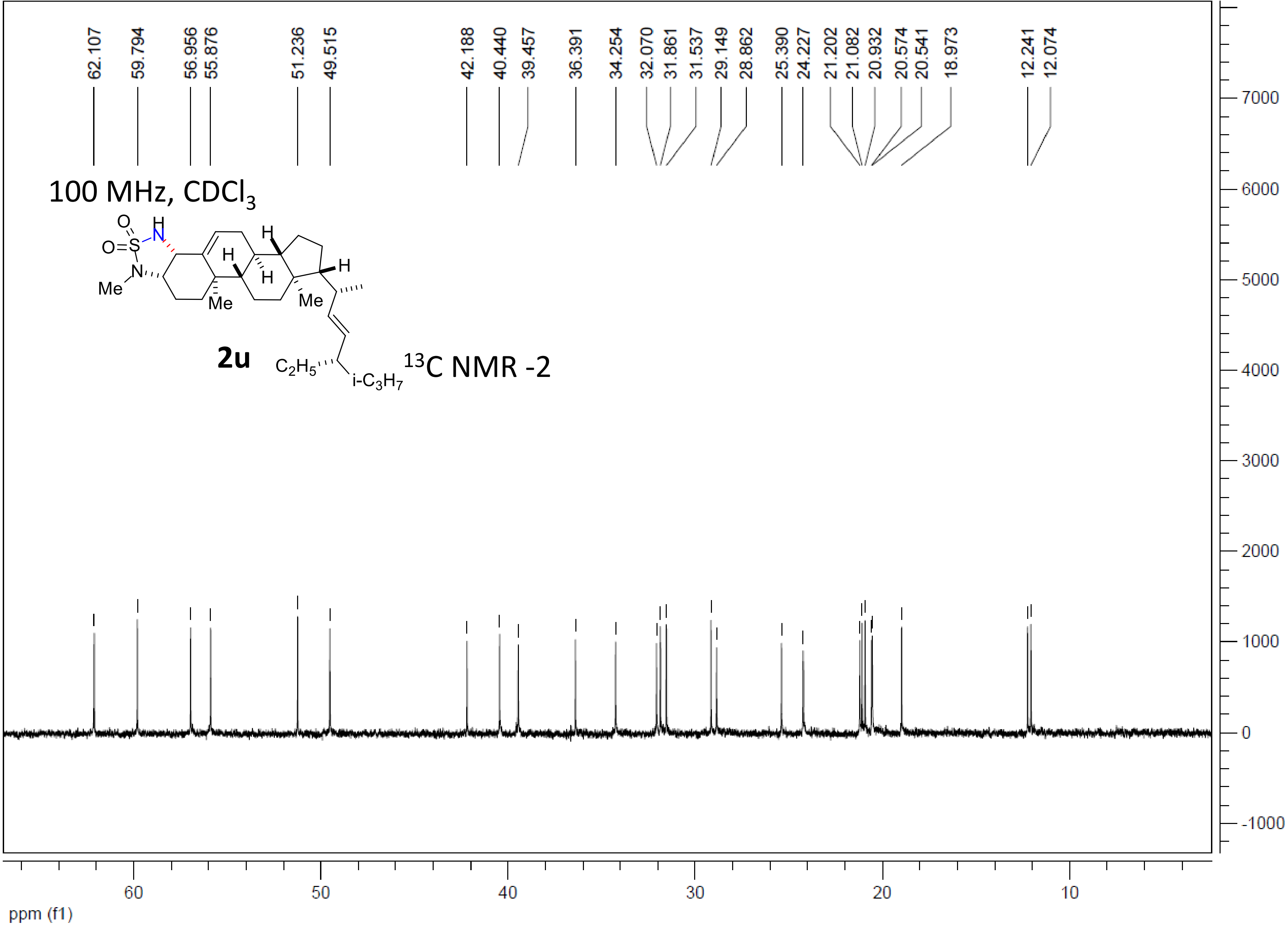
62.107
59.794
56.956
55.876
51.236
49.515
42.188
40.440
39.457
36.391
34.254
32.070
31.861
31.537
29.149
28.862
25.390
24.227
21.202
21.082
20.932
20.574
20.541
18.973
12.241
12.074
100 MHz, CDCl3
O
O=S
N
H
N
Me
H
H
H
H
Me
Me
2u
C2H5
i-C3H7
13C NMR -2
7000
6000
5000
4000
3000
2000
1000
0
-1000
60
50
40
30
20
10
ppm (f1)

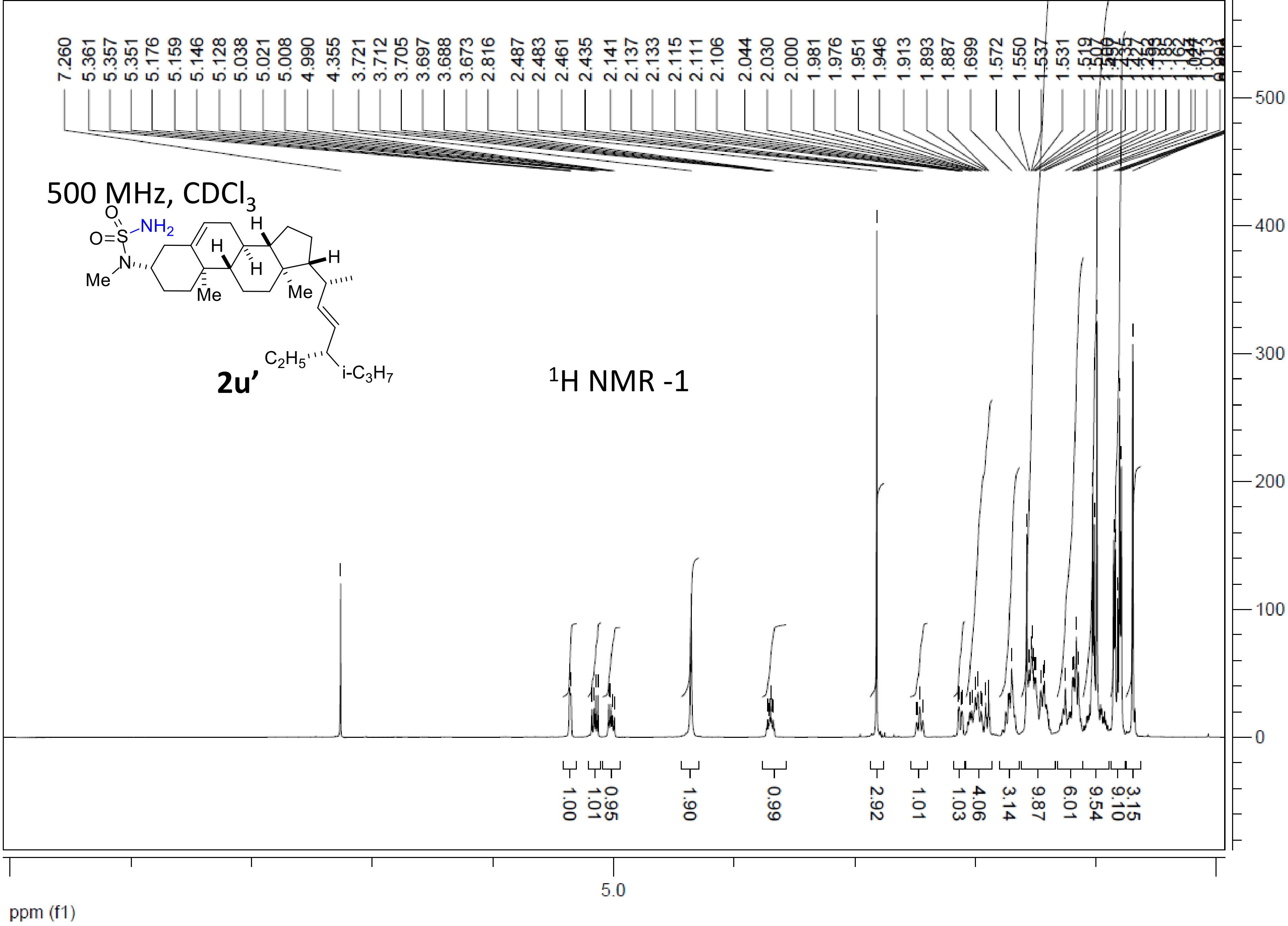
7.260
5.361
5.357
5.351
5.176
5.159
5.146
5.128
5.038
5.021
5.008
4.990
4.355
3.721
3.712
3.705
3.697
3.688
3.673
2.816
2.487
2.483
2.461
2.435
2.141
2.137
2.133
2.115
2.111
2.106
2.044
2.030
2.000
1.981
1.976
1.951
1.946
1.913
1.893
1.887
1.699
1.572
1.550
1.537
1.531
1.519
500 MHz, CDCl3
O
O=S
NH2
N
Me
H
H
H
H
Me
Me
C2H5
i-C3H7
2u’
1H NMR -1
500
400
300
200
100
0
1.00
1.01
0.95
1.90
0.99
2.92
1.01
1.03
4.06
3.14
9.87
6.01
9.54
9.10
3.15
5.0
ppm (f1)

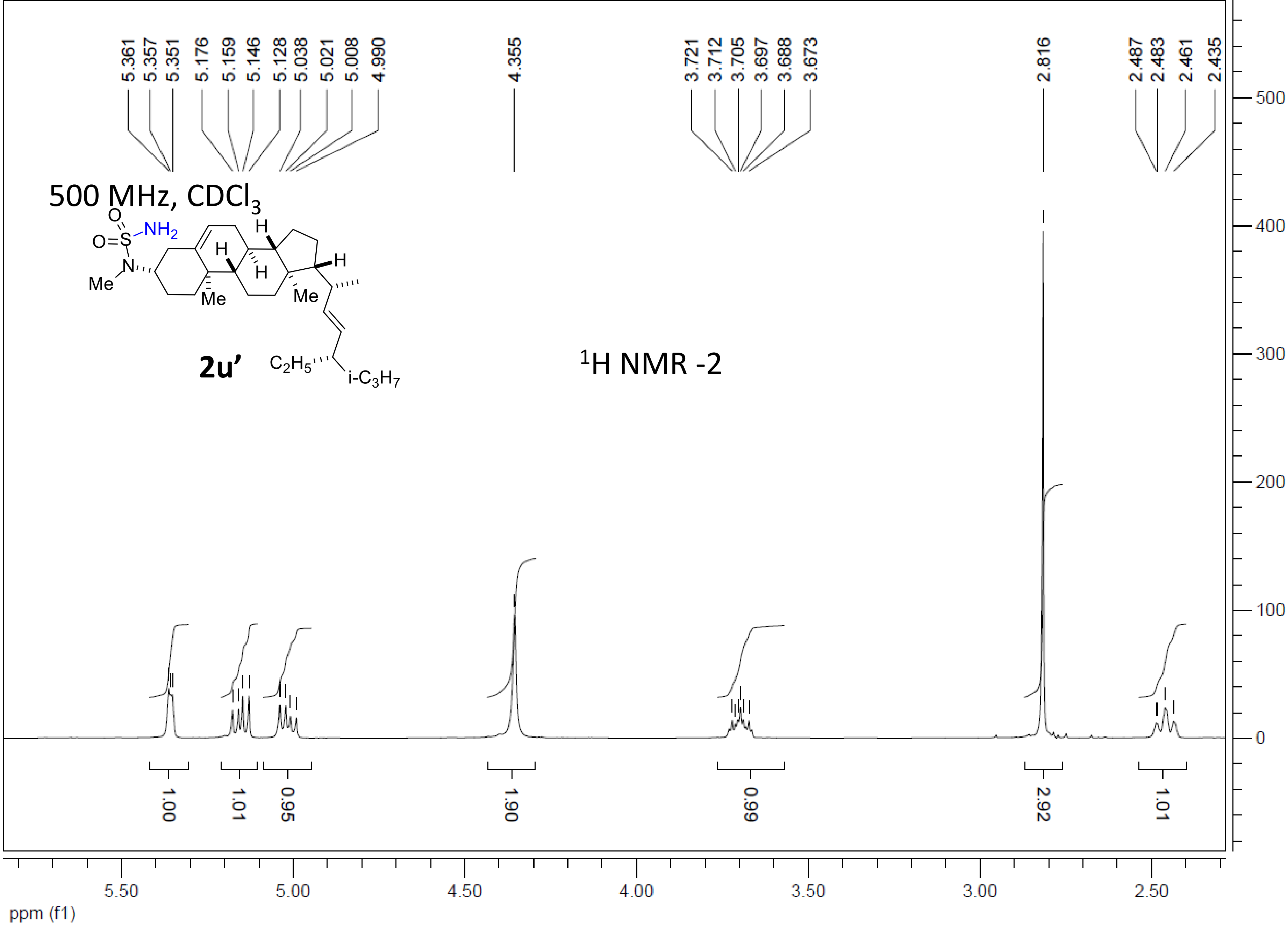
5.361
5.357
5.351
5.176
5.159
5.146
5.128
5.038
5.021
5.008
4.990
4.355
3.721
3.712
3.705
3.697
3.688
3.673
2.816
2.487
2.483
2.461
2.435
500 MHz, CDCl3
2u'
1H NMR -2
1.00
1.01
0.95
1.90
0.99
2.92
1.01
5.50
5.00
4.50
4.00
3.50
3.00
2.50
ppm (f1)
500
400
300
200
100
0

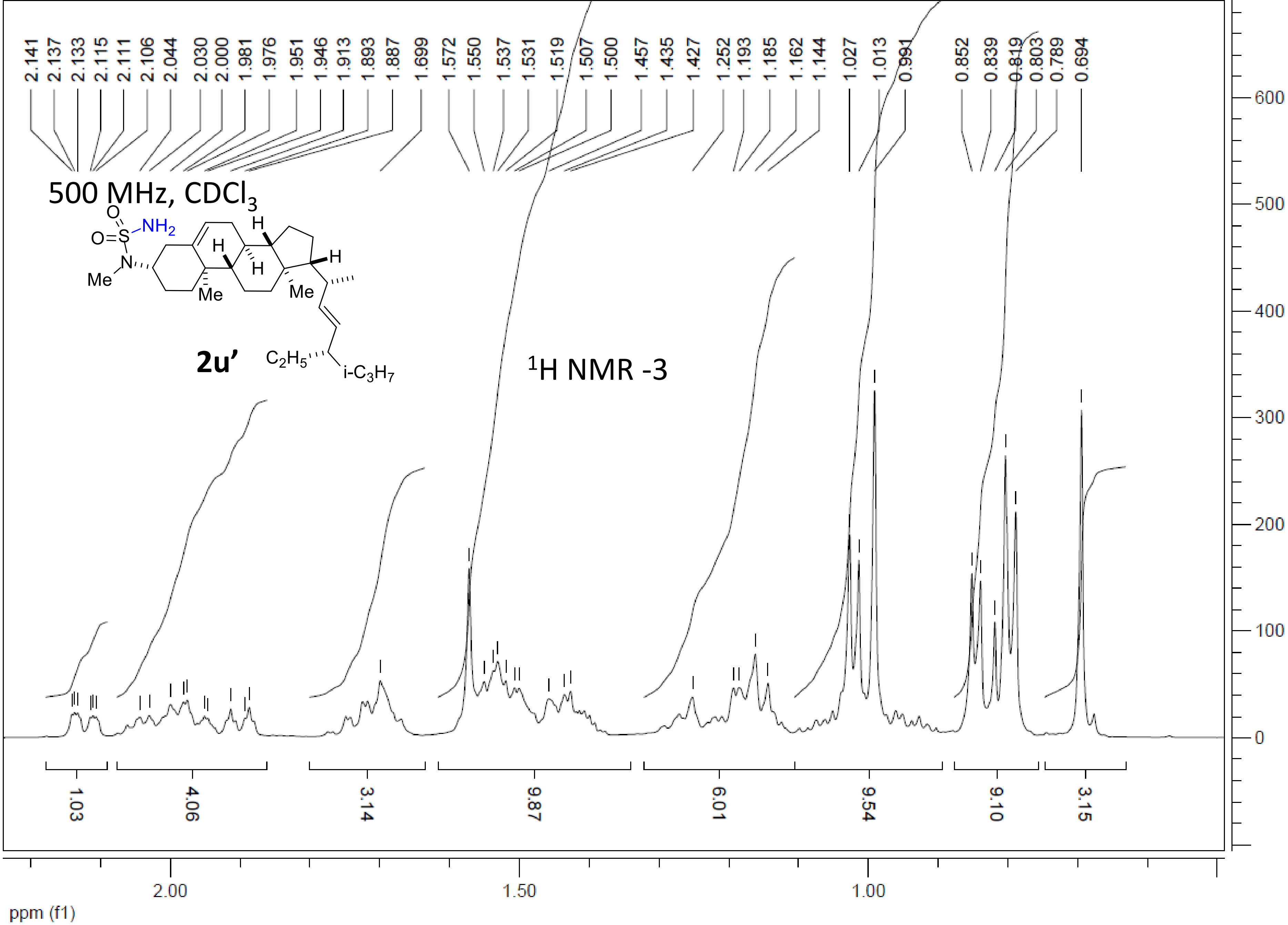
500 MHz, CDCl3
2u’
1H NMR -3
ppm (f1)
2.141 2.137 2.133 2.115 2.111 2.106 2.044 2.030 2.000 1.981 1.976 1.951 1.946 1.913 1.893 1.887 1.699 1.572 1.550 1.537 1.531 1.519 1.507 1.500 1.457 1.435 1.427 1.252 1.193 1.185 1.162 1.144 1.027 1.013 0.991 0.852 0.839 0.819 0.803 0.789 0.694
1.03
4.06
3.14
9.87
6.01
9.54
9.10
3.15
2.00
1.50
1.00

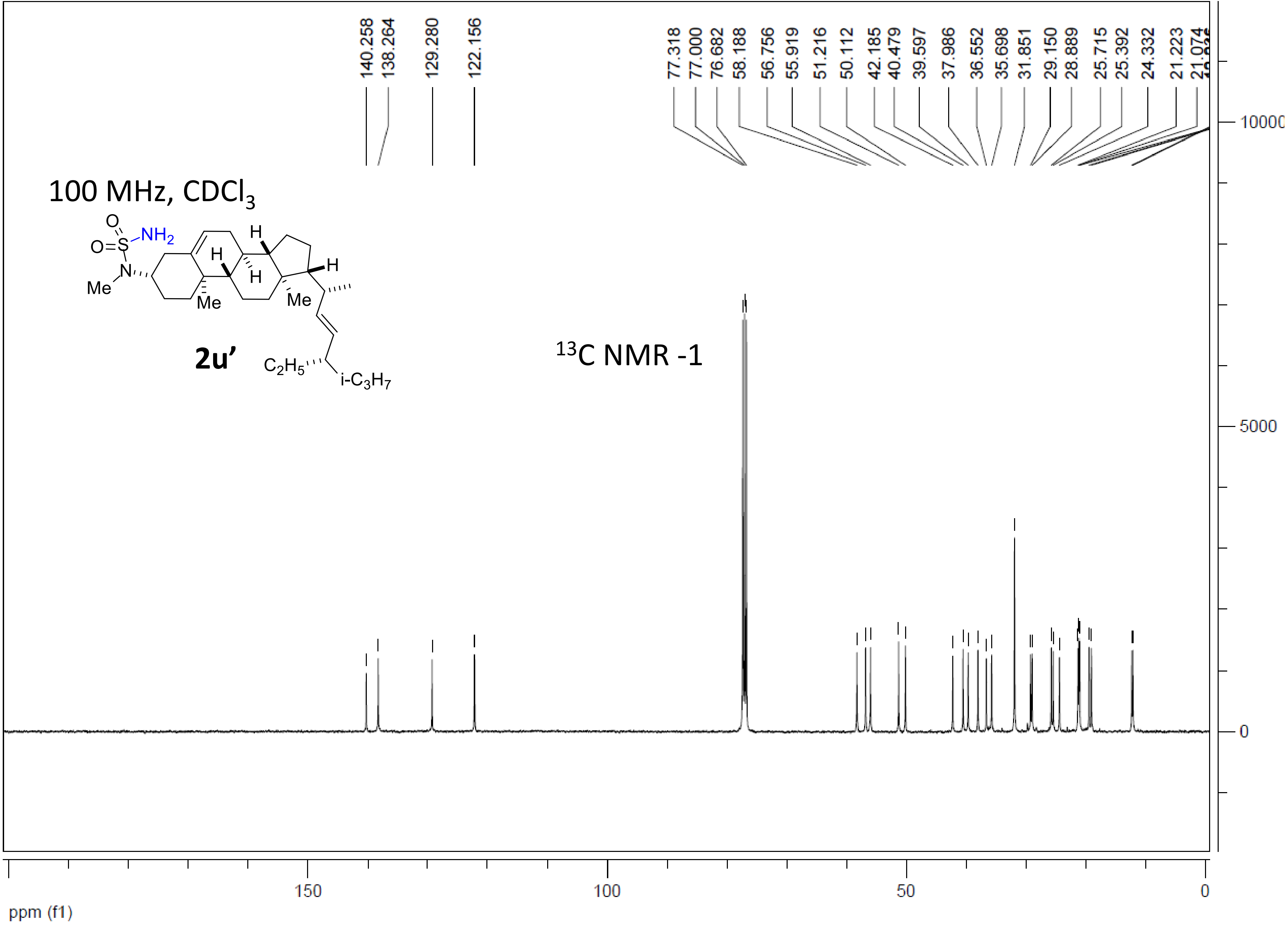

140.258
138.264
129.280
122.156
77.318
77.000
76.682
58.188
56.756
55.919
51.216
50.112
42.185
40.479
39.597
37.986
36.552
35.698
31.851
29.150
28.889
25.715
25.392
24.332
21.223
21.074
100 MHz, CDCl3
O
O=S
NH2
N
Me
H
H
H
H
Me
Me
2u'
C2H5
i-C3H7
13C NMR -1
10000
5000
0
150
100
50
0
ppm (f1)

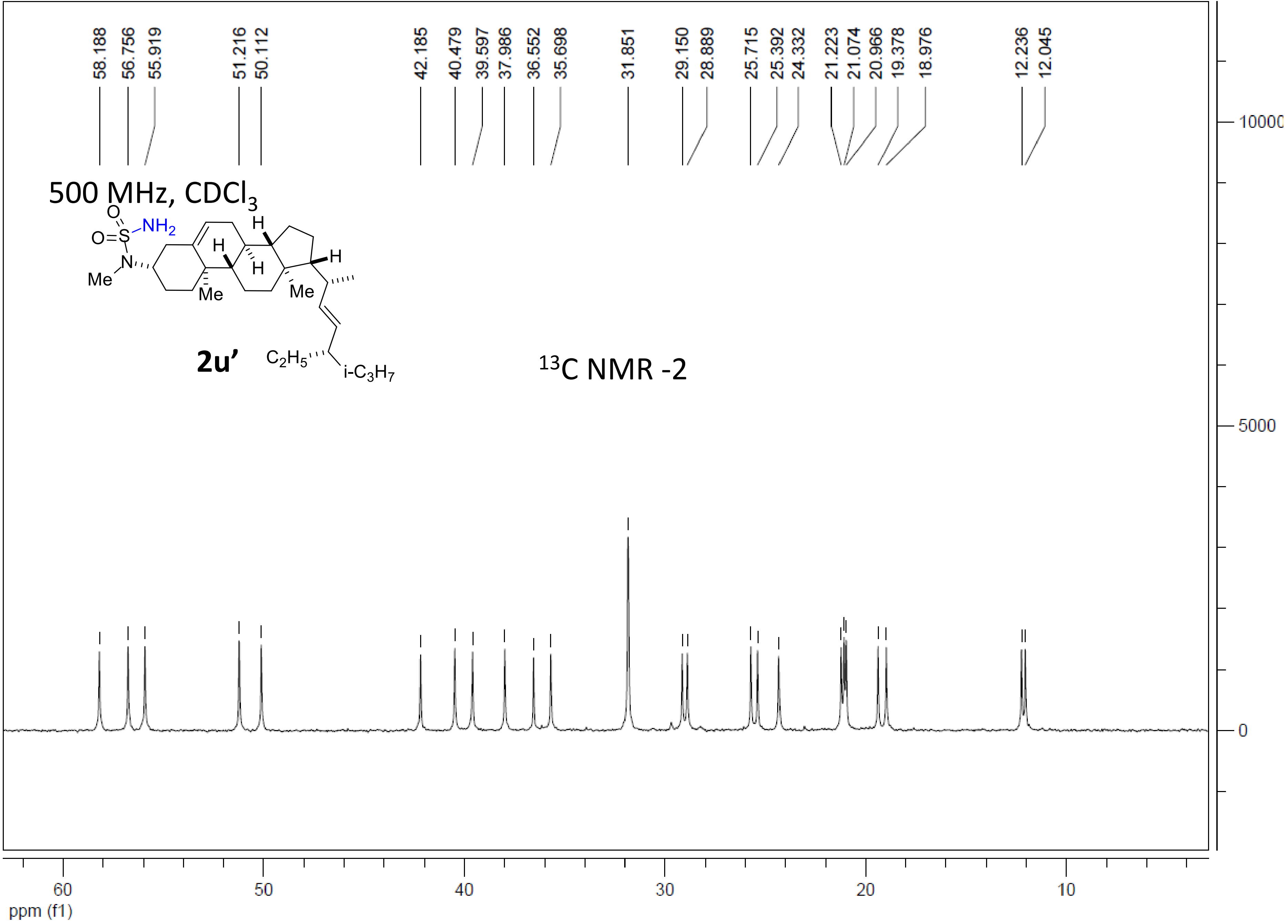
58.188
56.756
55.919
51.216
50.112
42.185
40.479
39.597
37.986
36.552
35.698
31.851
29.150
28.889
25.715
25.392
24.332
21.223
21.074
20.966
19.378
18.976
12.236
12.045
500 MHz, $CDCl_3$
2u'
^{13}C NMR -2
10000
5000
0
60
50
40
30
20
10
ppm (f1)

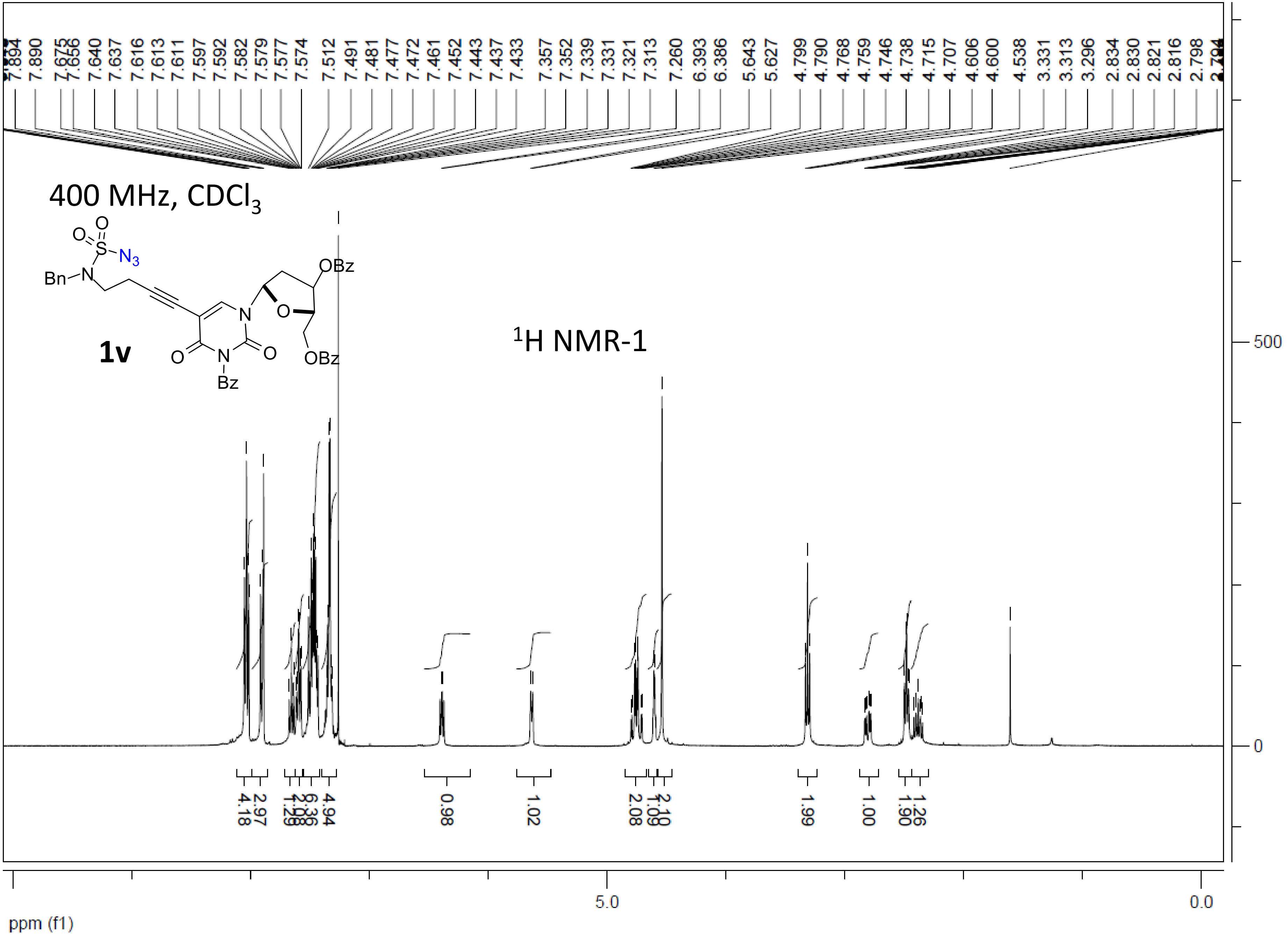
7.894
7.890
7.675
7.656
7.640
7.637
7.616
7.613
7.611
7.597
7.592
7.582
7.579
7.577
7.574
7.512
7.491
7.481
7.477
7.472
7.461
7.452
7.443
7.437
7.433
7.357
7.352
7.339
7.331
7.321
7.313
7.260
6.393
6.386
5.643
5.627
4.799
4.790
4.768
4.759
4.746
4.738
4.715
4.707
4.606
4.600
4.538
3.331
3.313
3.296
2.834
2.830
2.821
2.816
2.798
400 MHz, $CDCl_3$
Bn
N
O
S
N_3
OBz
N
O
1v
O
N
O
OBz
Bz
^{1}H NMR-1
500
0
4.18
2.97
1.29
4.08
6.36
4.94
0.98
1.02
2.08
1.09
2.10
1.99
1.00
1.90
1.26
5.0
0.0
ppm (f1)

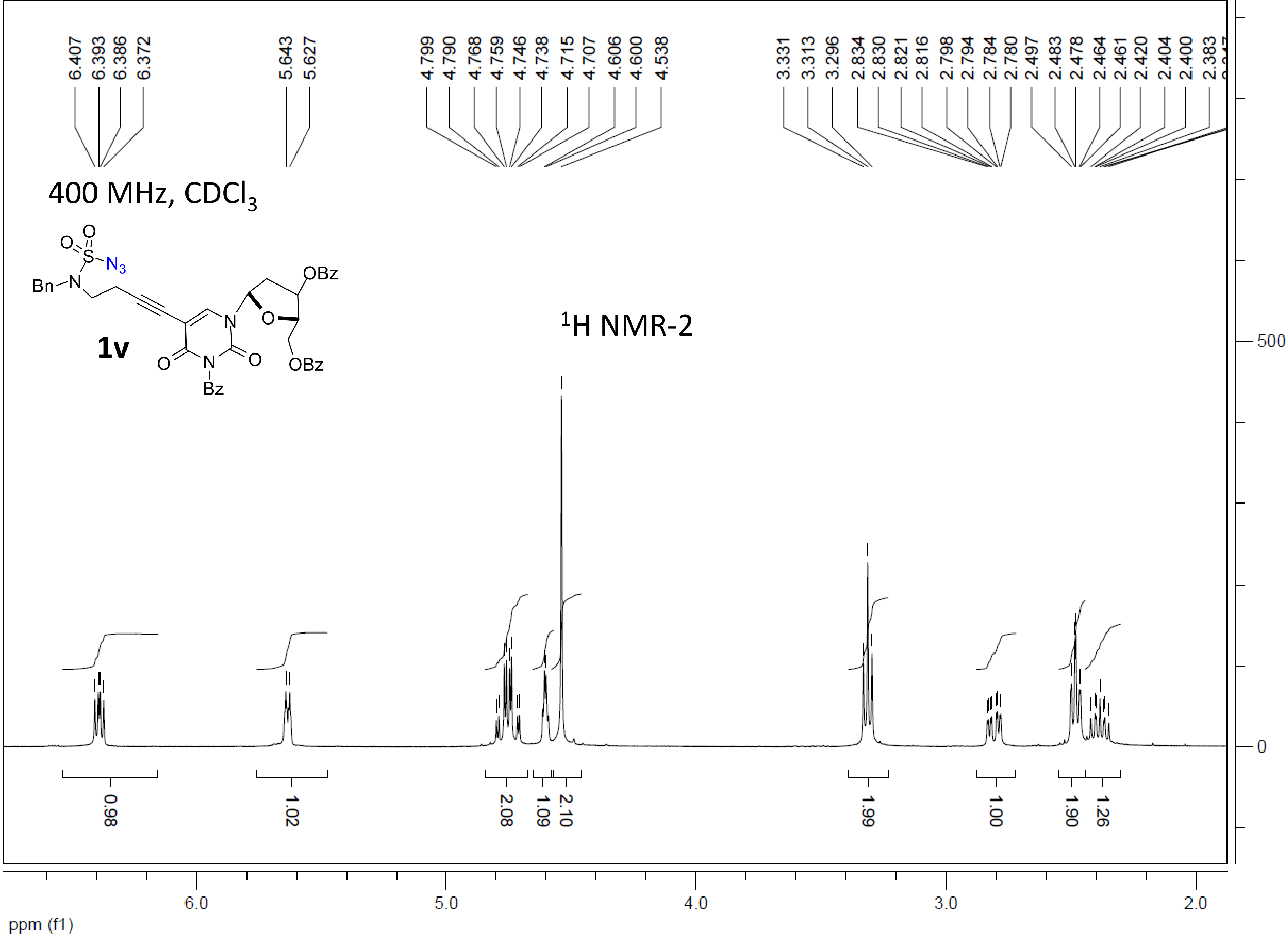

6.407
6.393
6.386
6.372
5.643
5.627
4.799
4.790
4.768
4.759
4.746
4.738
4.715
4.707
4.606
4.600
4.538
3.331
3.313
3.296
2.834
2.830
2.821
2.816
2.798
2.794
2.784
2.780
2.497
2.483
2.478
2.464
2.461
2.420
2.404
2.400
2.383
400 MHz, $CDCl_3$
O
O
S
N_3
Bn
N
OBz
N
O
1v
O
N
O
OBz
Bz
^{1}H NMR-2
500
0
0.98
1.02
2.08
1.09
2.10
1.99
1.00
1.90
1.26
6.0
5.0
4.0
3.0
2.0
ppm (f1)

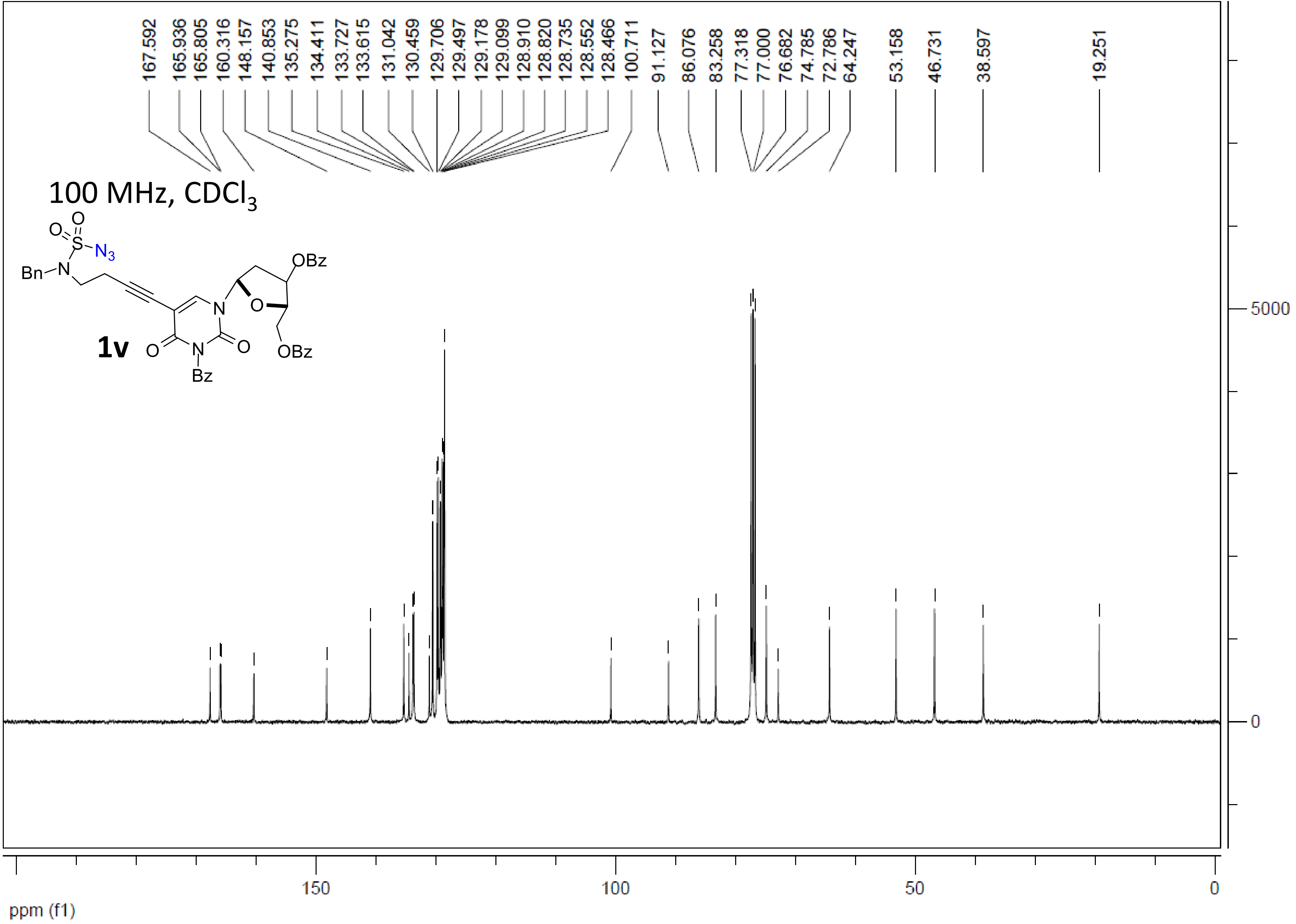
167.592
165.936
165.805
160.316
148.157
140.853
135.275
134.411
133.727
133.615
131.042
130.459
129.706
129.497
129.178
129.099
128.910
128.820
128.735
128.552
128.466
100.711
91.127
86.076
83.258
77.318
77.000
76.682
74.785
72.786
64.247
53.158
46.731
38.597
19.251
100 MHz, CDCl3
Bn
N
S
O
O
N3
OBz
OBz
O
N
O
N
O
Bz
1v
5000
0
150
100
50
0
ppm (f1)

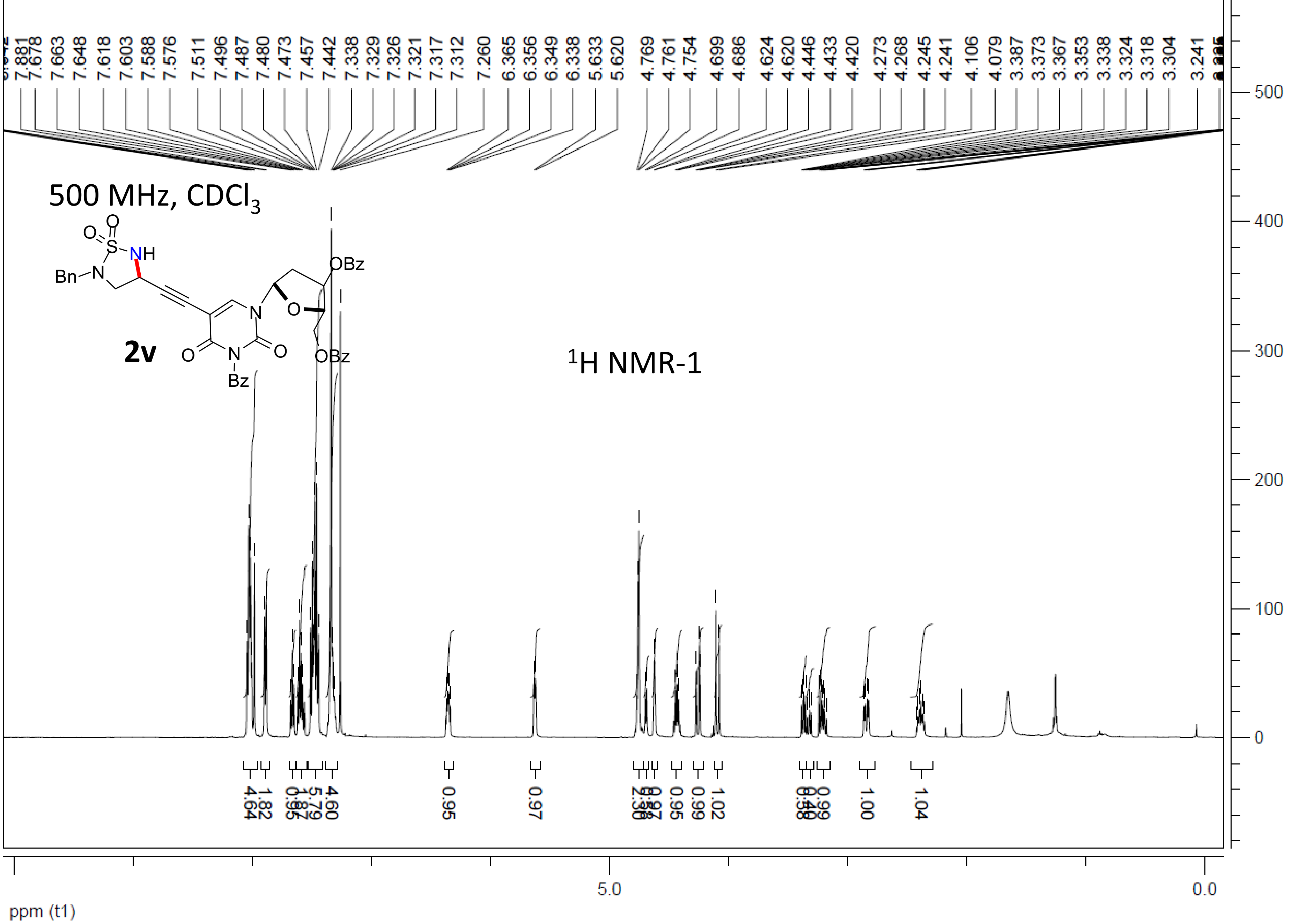

500 MHz, $CDCl_3$
O
O
S
N
H
Bn
N
OBz
O
N
O
N
O
Bz
OBz
2v
^{1}H NMR-1
7.881
7.678
7.663
7.648
7.618
7.603
7.588
7.576
7.511
7.496
7.487
7.480
7.473
7.457
7.442
7.338
7.329
7.326
7.321
7.317
7.312
7.260
6.365
6.356
6.349
6.338
5.633
5.620
4.769
4.761
4.754
4.699
4.686
4.624
4.620
4.446
4.433
4.420
4.273
4.268
4.245
4.241
4.106
4.079
3.387
3.373
3.367
3.353
3.338
3.324
3.318
3.304
3.241
500
400
300
200
100
0
4.64
1.82
0.95
1.87
5.79
4.60
0.95
0.97
2.30
0.95
0.99
1.02
0.99
1.00
1.04
5.0
0.0
ppm (t1)

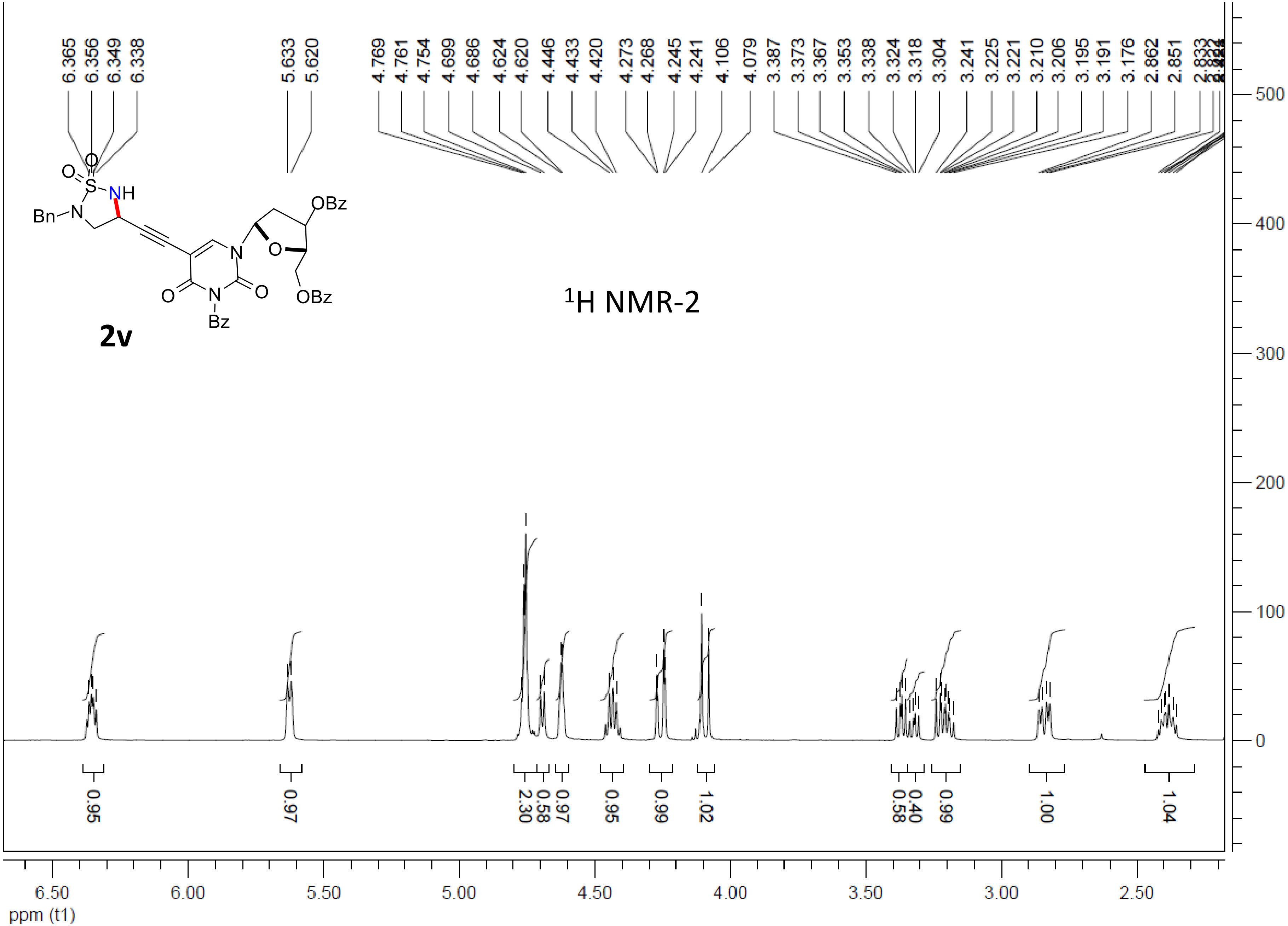

2v
1H NMR-2
ppm (t1)

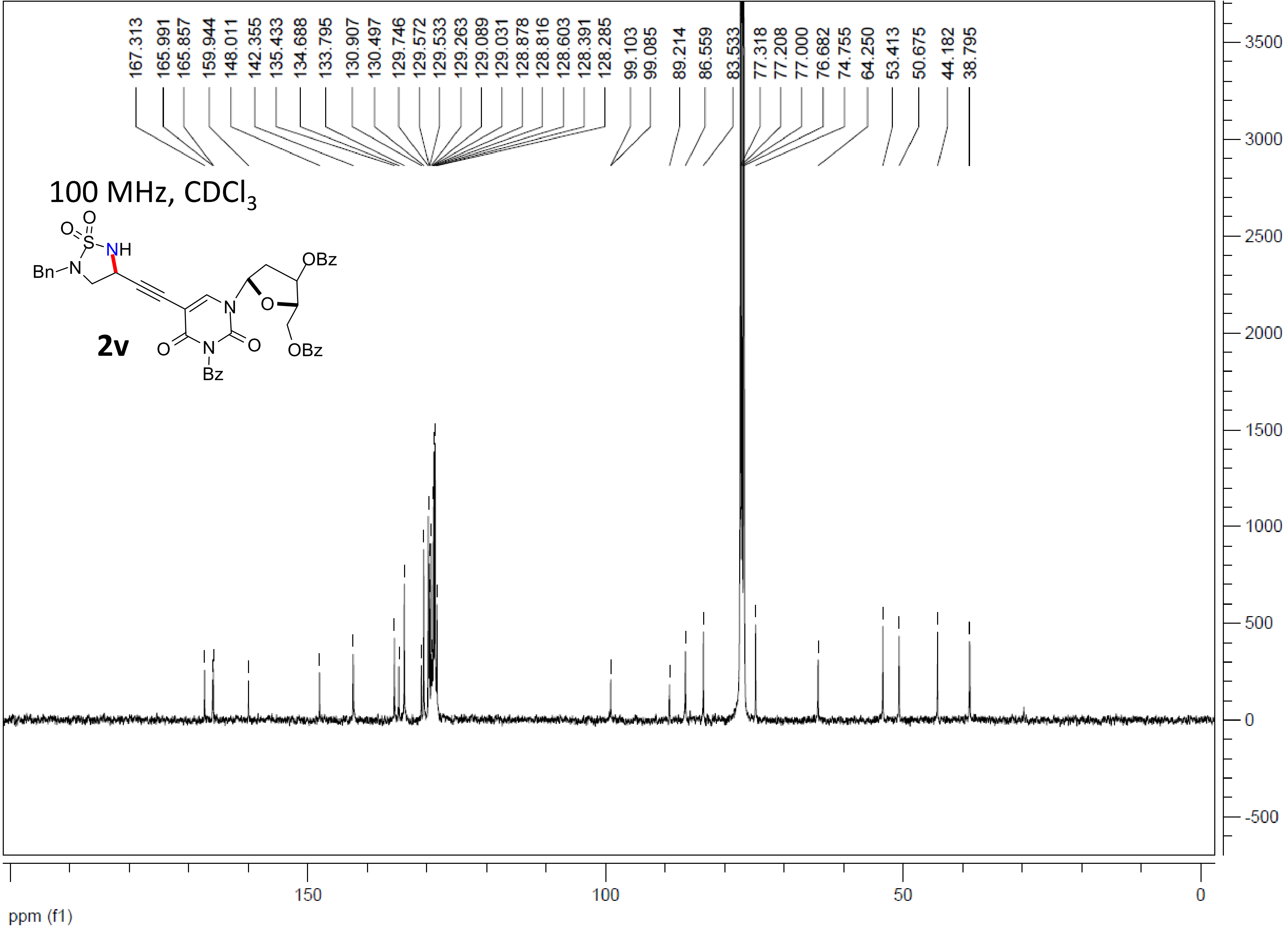
167.313
165.991
165.857
159.944
148.011
142.355
135.433
134.688
133.795
130.907
130.497
129.746
129.572
129.533
129.263
129.089
129.031
128.878
128.816
128.603
128.391
128.285
99.103
99.085
89.214
86.559
83.533
77.318
77.208
77.000
76.682
74.755
64.250
53.413
50.675
44.182
38.795
100 MHz, CDCl3
2v
OBz
Bz
Bn
NH
ppm (f1)

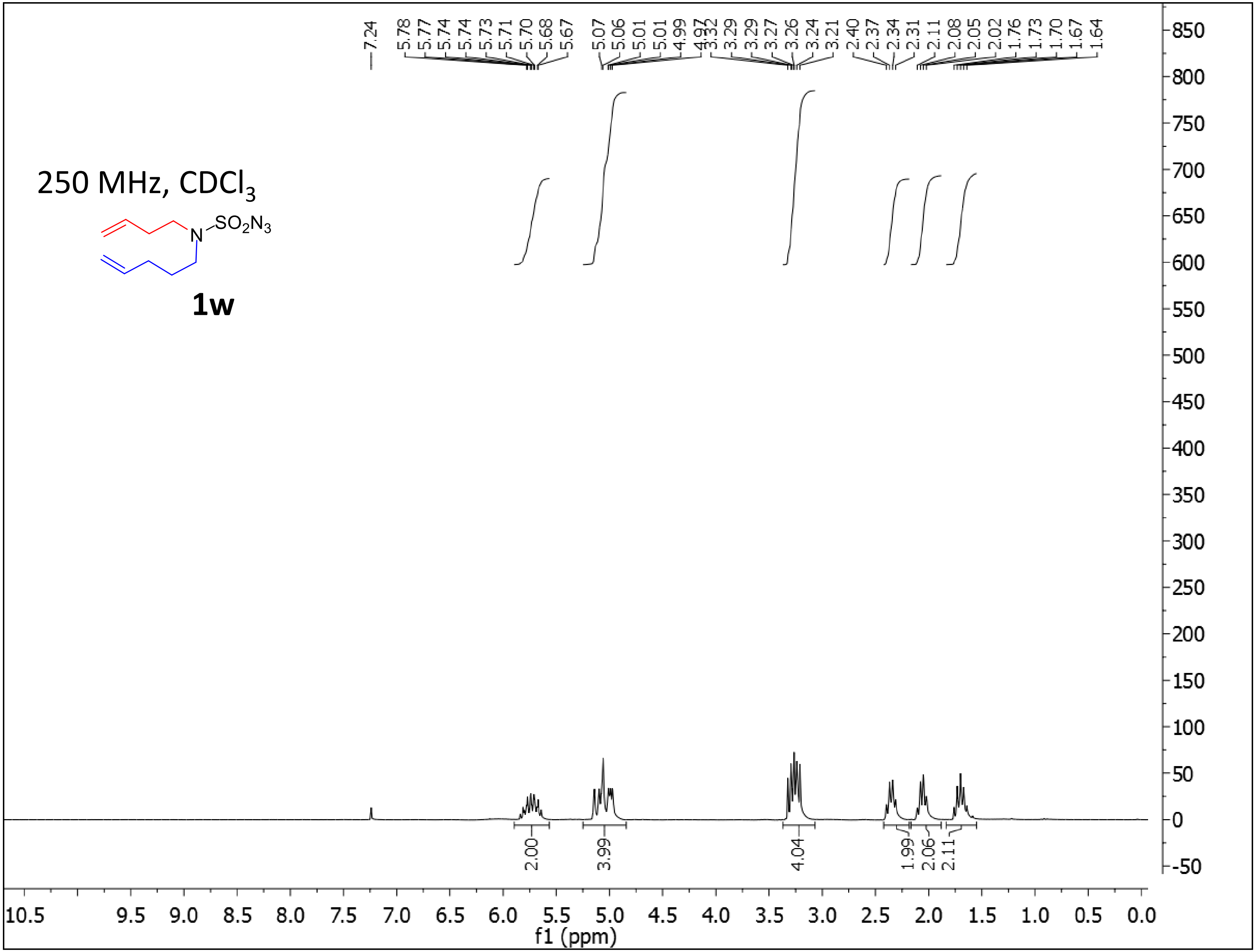
250 MHz, CDCl3
SO2N3
N
1w
7.24
5.78
5.77
5.74
5.74
5.73
5.71
5.70
5.68
5.67
5.07
5.06
5.01
5.01
4.99
4.97
3.32
3.29
3.29
3.27
3.26
3.24
3.21
2.40
2.37
2.34
2.31
2.11
2.08
2.05
2.02
1.76
1.73
1.70
1.67
1.64
2.00
3.99
4.04
1.99
2.06
2.11
f1 (ppm)

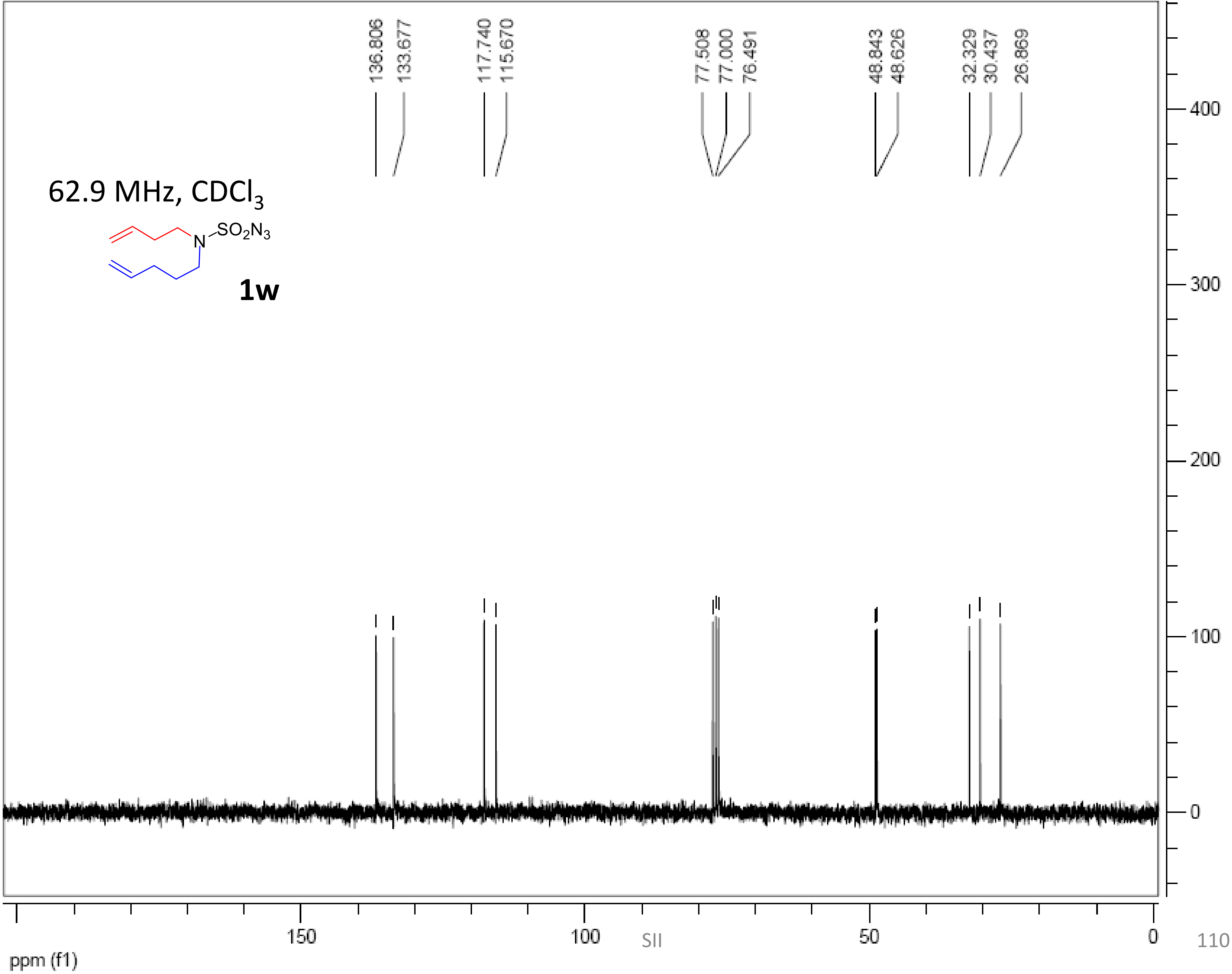
136.806
133.677
117.740
115.670
77.508
77.000
76.491
48.843
48.626
32.329
30.437
26.869
62.9 MHz, CDCl3
N
SO2N3
1w
400
300
200
100
0
150
100
50
0
ppm (f1)

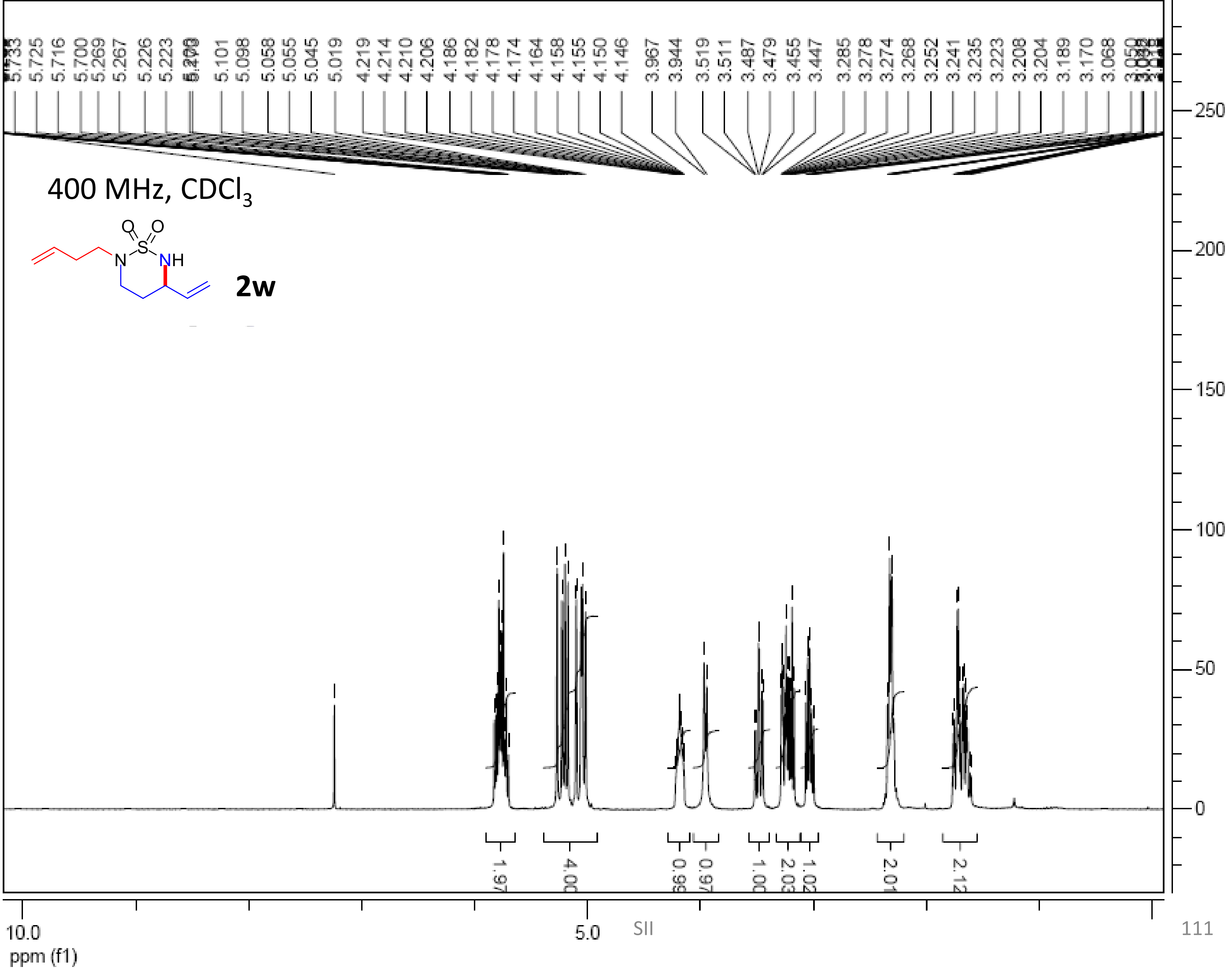

5.733
5.725
5.716
5.700
5.269
5.267
5.226
5.223
5.101
5.098
5.058
5.055
5.045
5.019
4.219
4.214
4.210
4.206
4.186
4.182
4.178
4.174
4.164
4.158
4.155
4.150
4.146
3.967
3.944
3.519
3.511
3.487
3.479
3.455
3.447
3.285
3.278
3.274
3.268
3.252
3.241
3.235
3.223
3.208
3.204
3.189
3.170
3.068
3.050
400 MHz, $CDCl_3$
O O
S
N
NH
2w
1.97
4.00
0.99
0.97
1.00
2.03
1.02
2.01
2.12
250
200
150
100
50
0
10.0
5.0
ppm (f1)

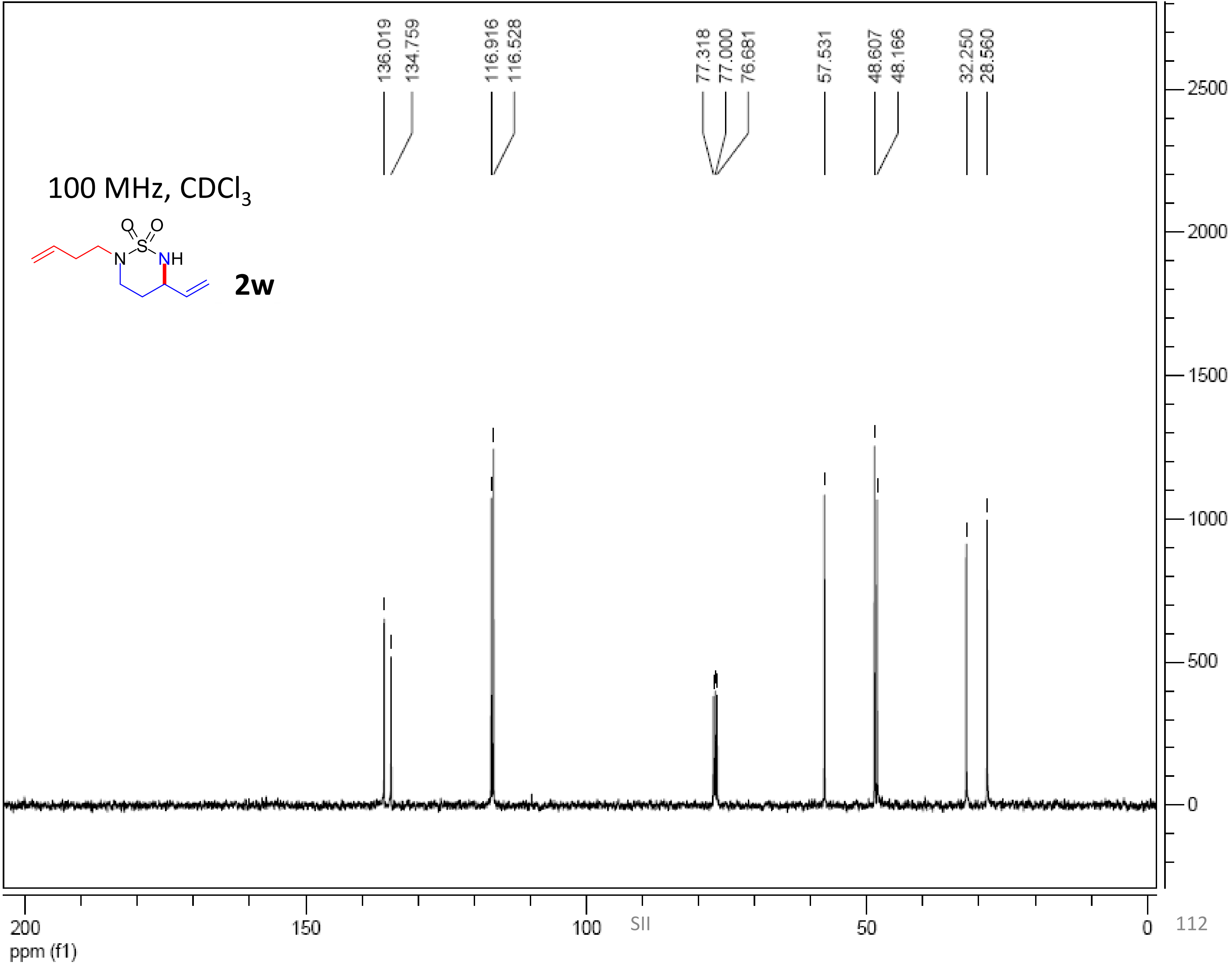
136.019
134.759
116.916
116.528
77.318
77.000
76.681
57.531
48.607
48.166
32.250
28.560
100 MHz, CDCl3
2w
2500
2000
1500
1000
500
0
200
150
100
50
0
ppm (f1)

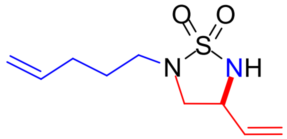

2w′

400 MHz, $CDCl_3$

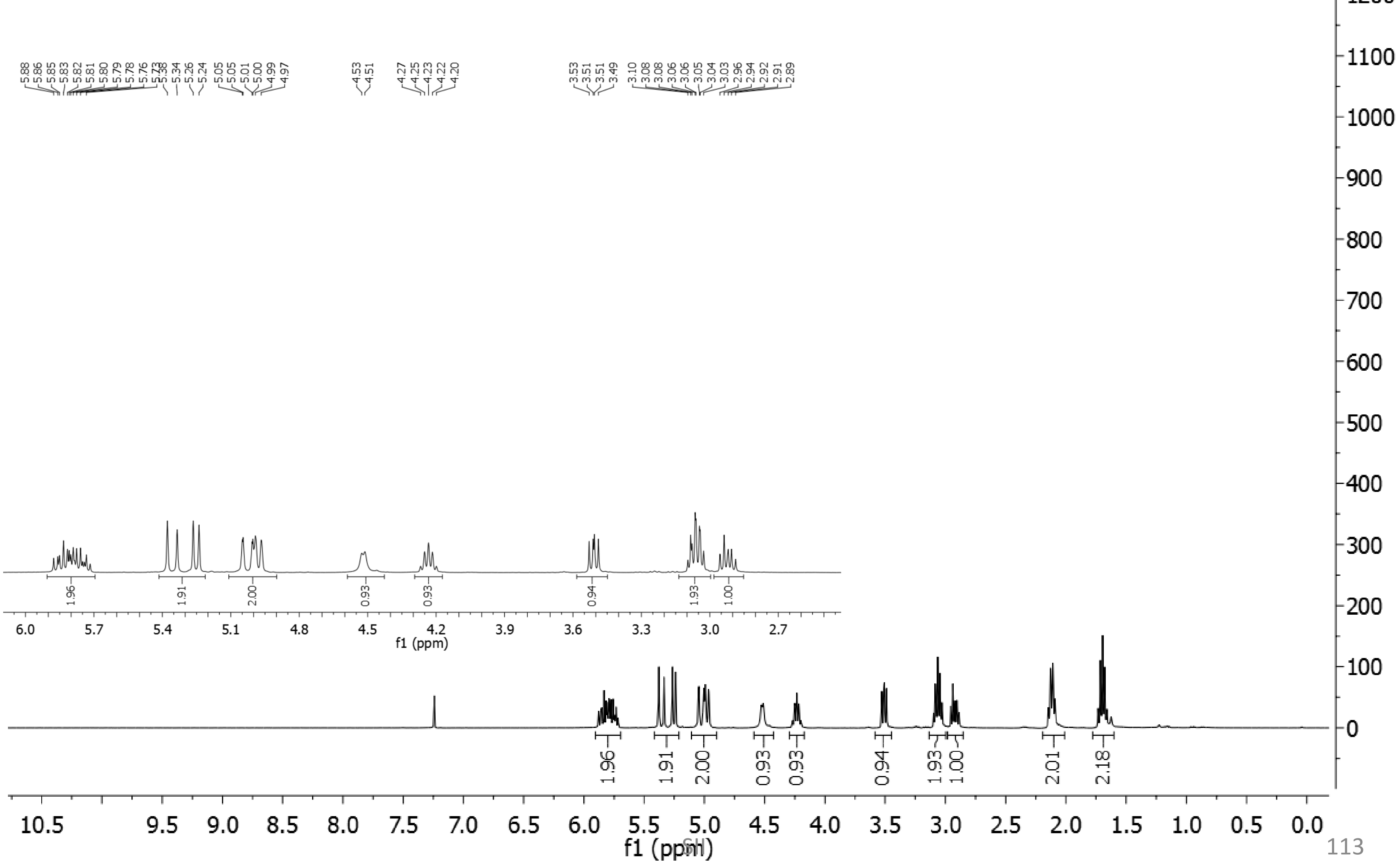

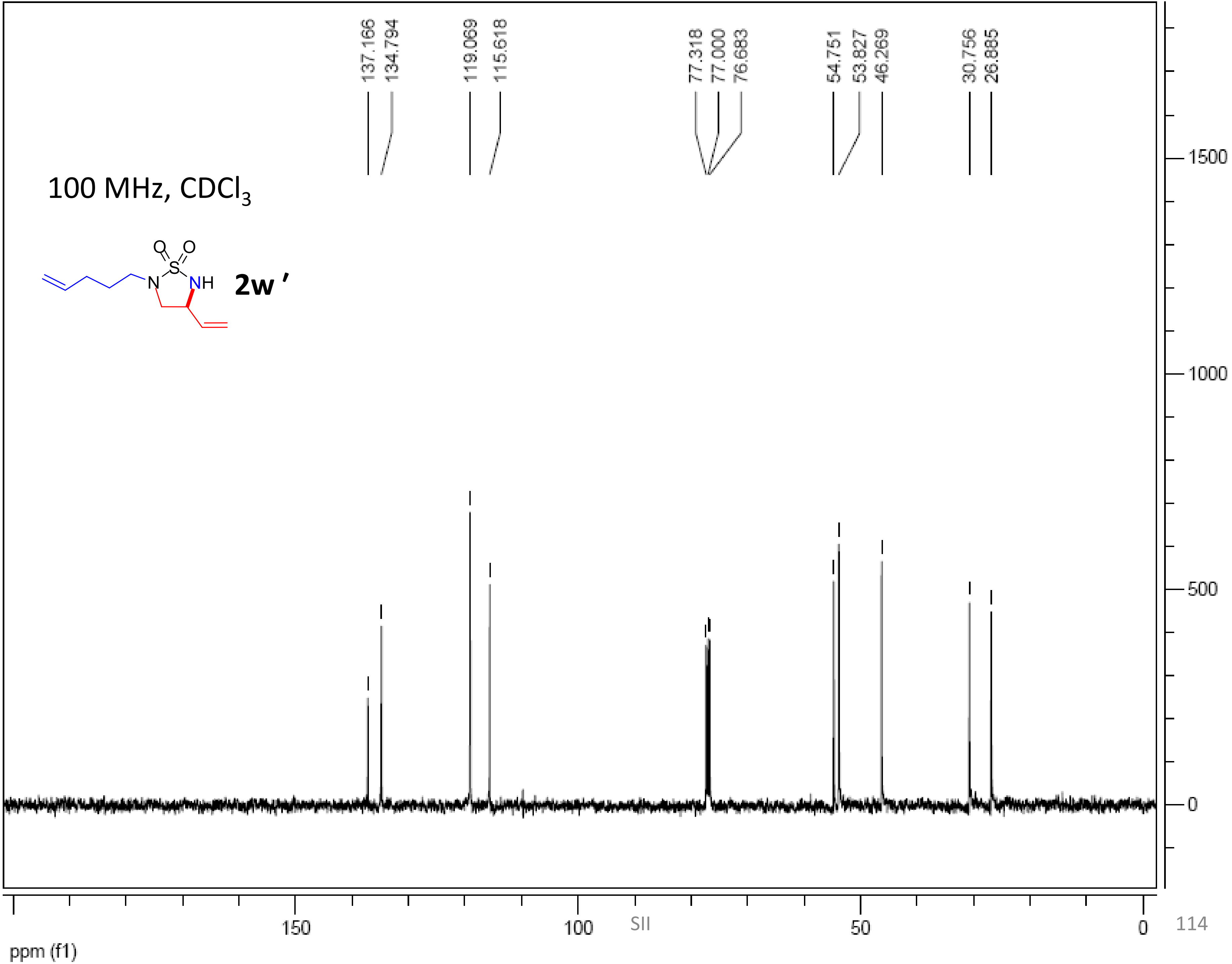

100 MHz, CDCl3
2w ʹ
137.166
134.794
119.069
115.618
77.318
77.000
76.683
54.751
53.827
46.269
30.756
26.885
1500
1000
500
0
150
100
50
0
ppm (f1)

400 MHz, $CDCl_3$

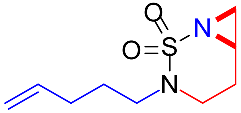

2w''

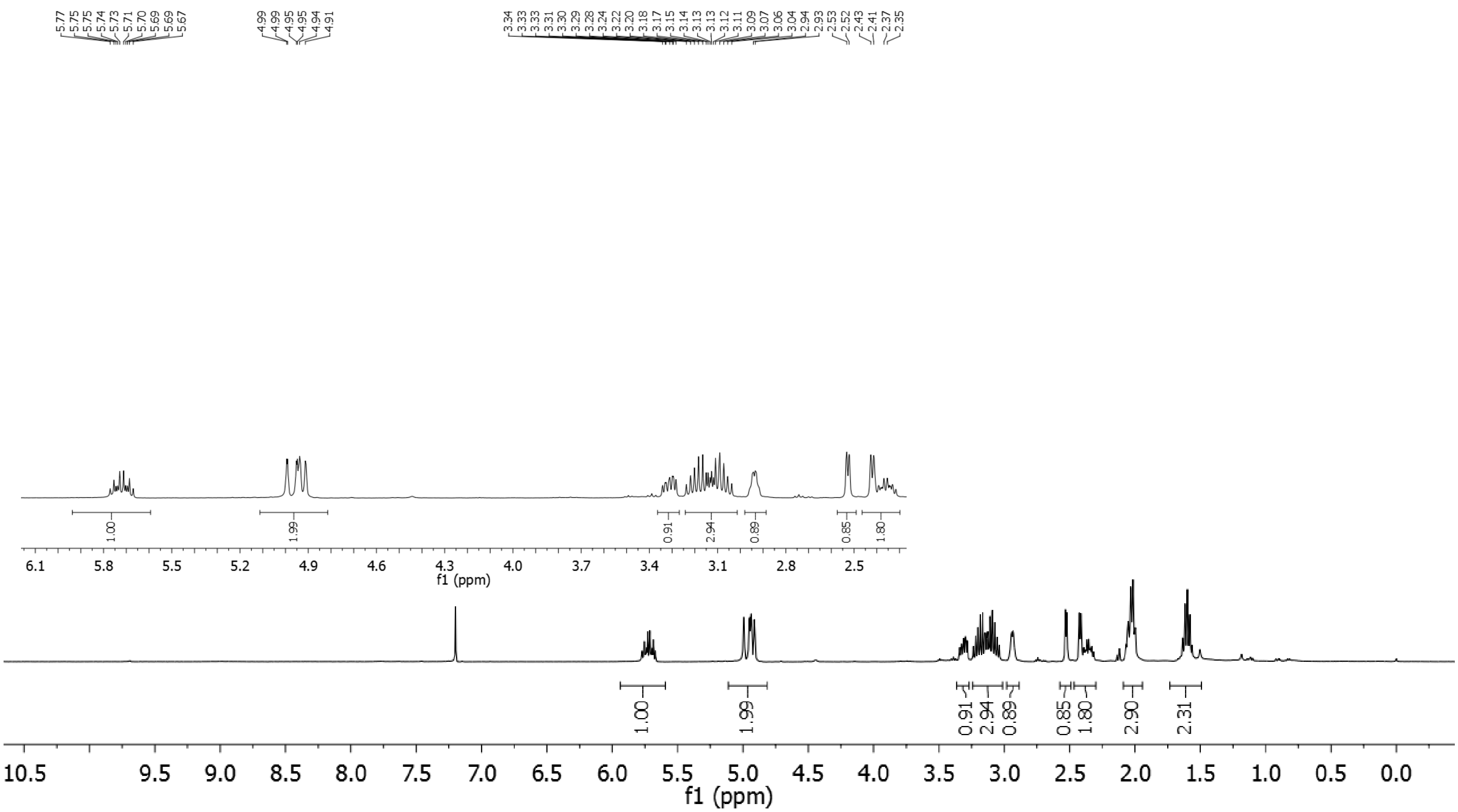

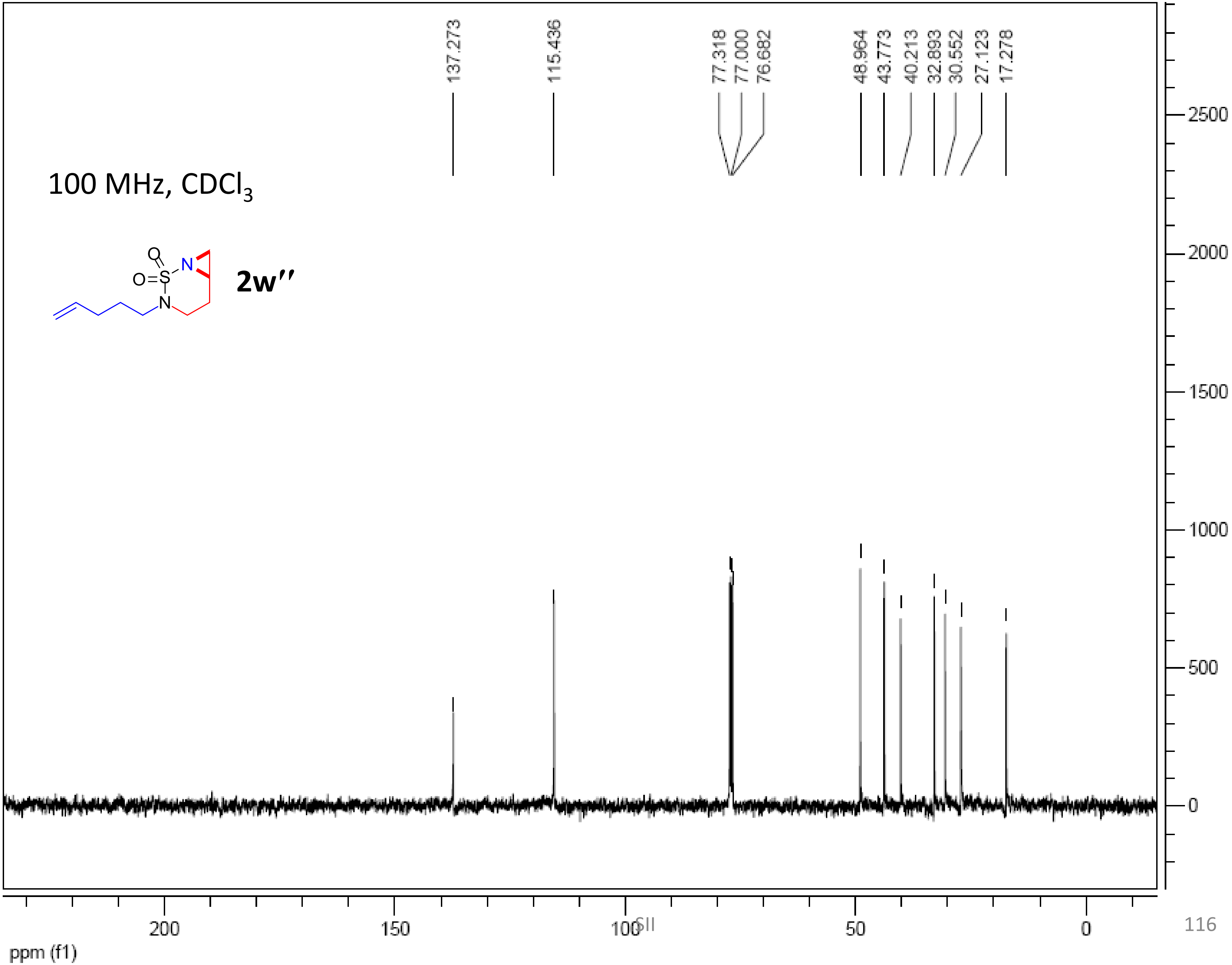

137.273
115.436
77.318
77.000
76.682
48.964
43.773
40.213
32.893
30.552
27.123
17.278
100 MHz, CDCl3
2w''
2500
2000
1500
1000
500
0
200
150
100
50
0
ppm (f1)

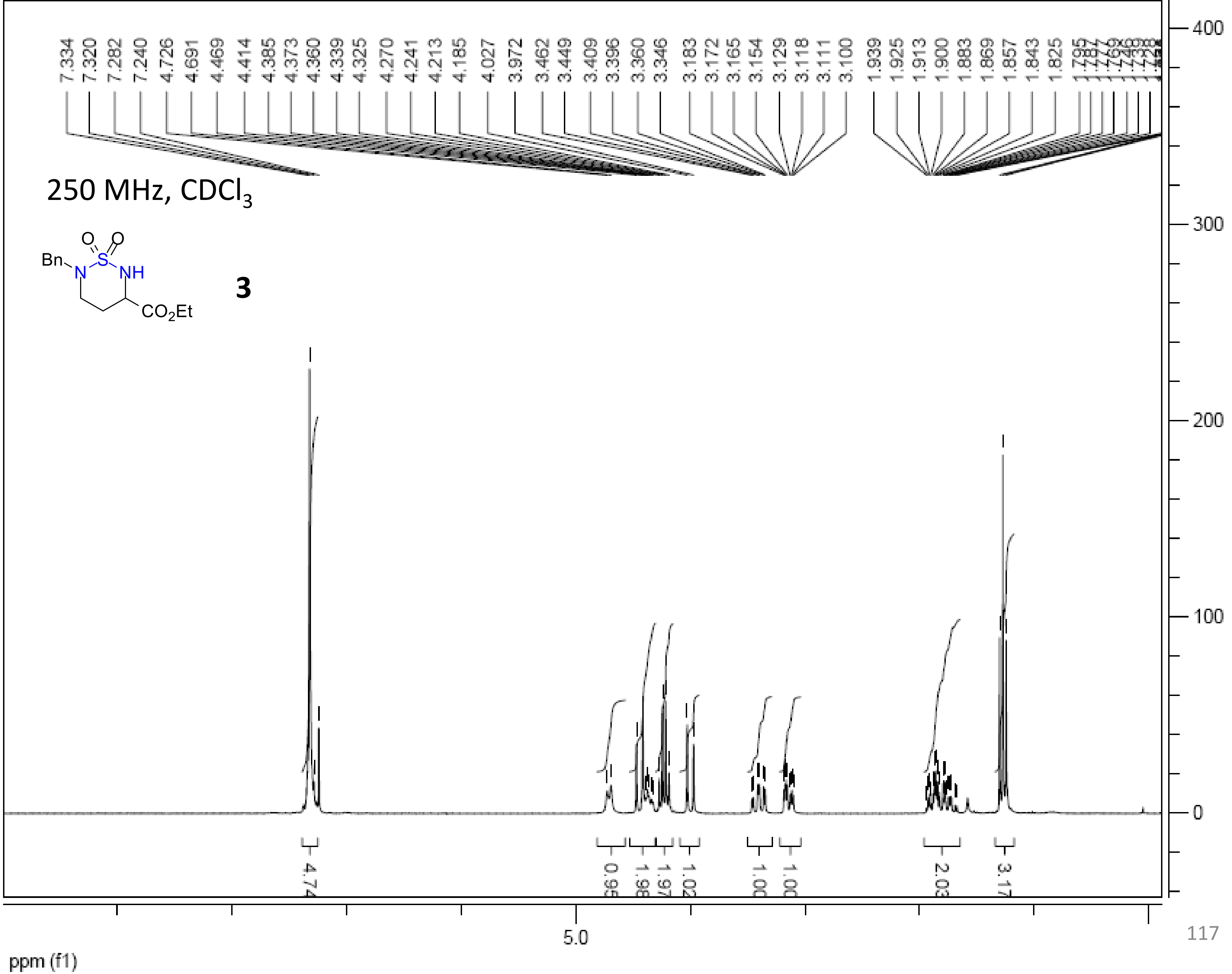
250 MHz, $CDCl_3$
Bn
N
S
NH
O
O
CO_2Et
3
7.334
7.320
7.282
7.240
4.726
4.691
4.469
4.414
4.385
4.373
4.360
4.339
4.325
4.270
4.241
4.213
4.185
4.027
3.972
3.462
3.449
3.409
3.396
3.360
3.346
3.183
3.172
3.165
3.154
3.129
3.118
3.111
3.100
1.939
1.925
1.913
1.900
1.883
1.869
1.857
1.843
1.825
1.795
4.74
0.95
1.98
1.97
1.02
1.00
1.00
2.03
3.17
400
300
200
100
0
5.0
ppm (f1)

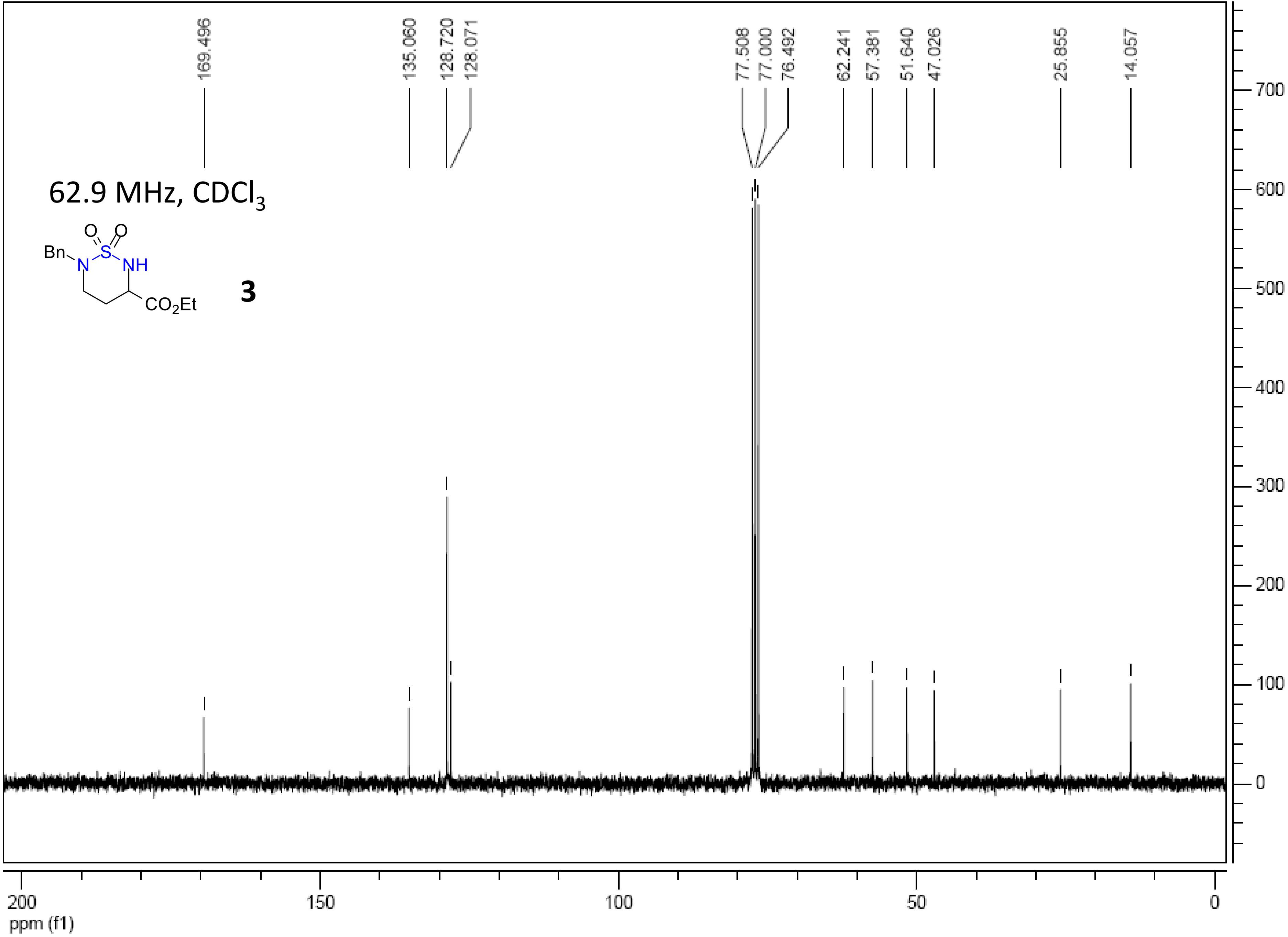
169.496
135.060
128.720
128.071
77.508
77.000
76.492
62.241
57.381
51.640
47.026
25.855
14.057
62.9 MHz, CDCl3
Bn
N
S
O
O
NH
CO2Et
3
700
600
500
400
300
200
100
0
200
150
100
50
0
ppm (f1)

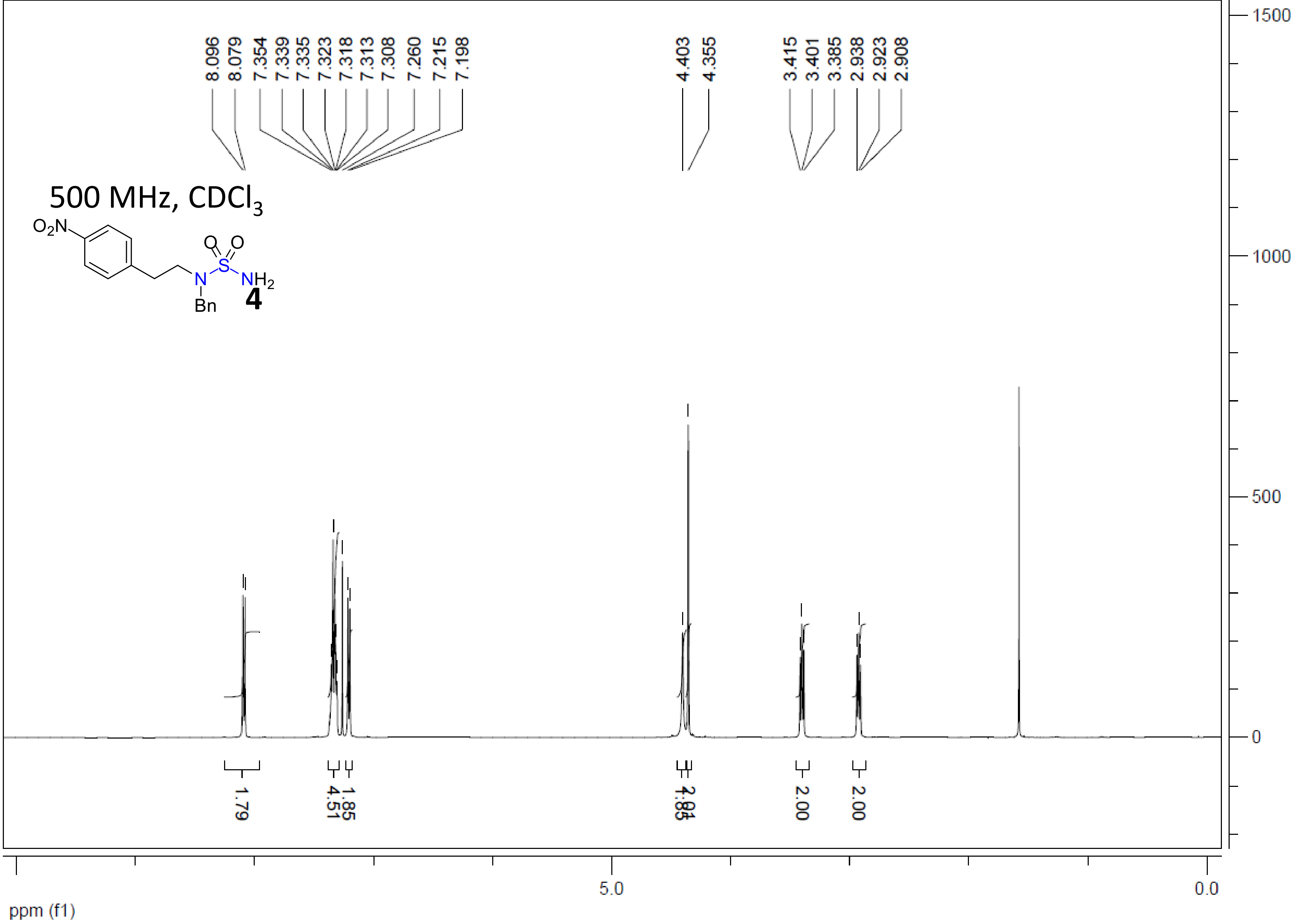

8.096
8.079
7.354
7.339
7.335
7.323
7.318
7.313
7.308
7.260
7.215
7.198
4.403
4.355
3.415
3.401
3.385
2.938
2.923
2.908
500 MHz, CDCl3
O2N
O
O
S
N
NH2
Bn
4
1500
1000
500
0
1.79
4.51
1.85
2.00
2.00
5.0
0.0
ppm (f1)

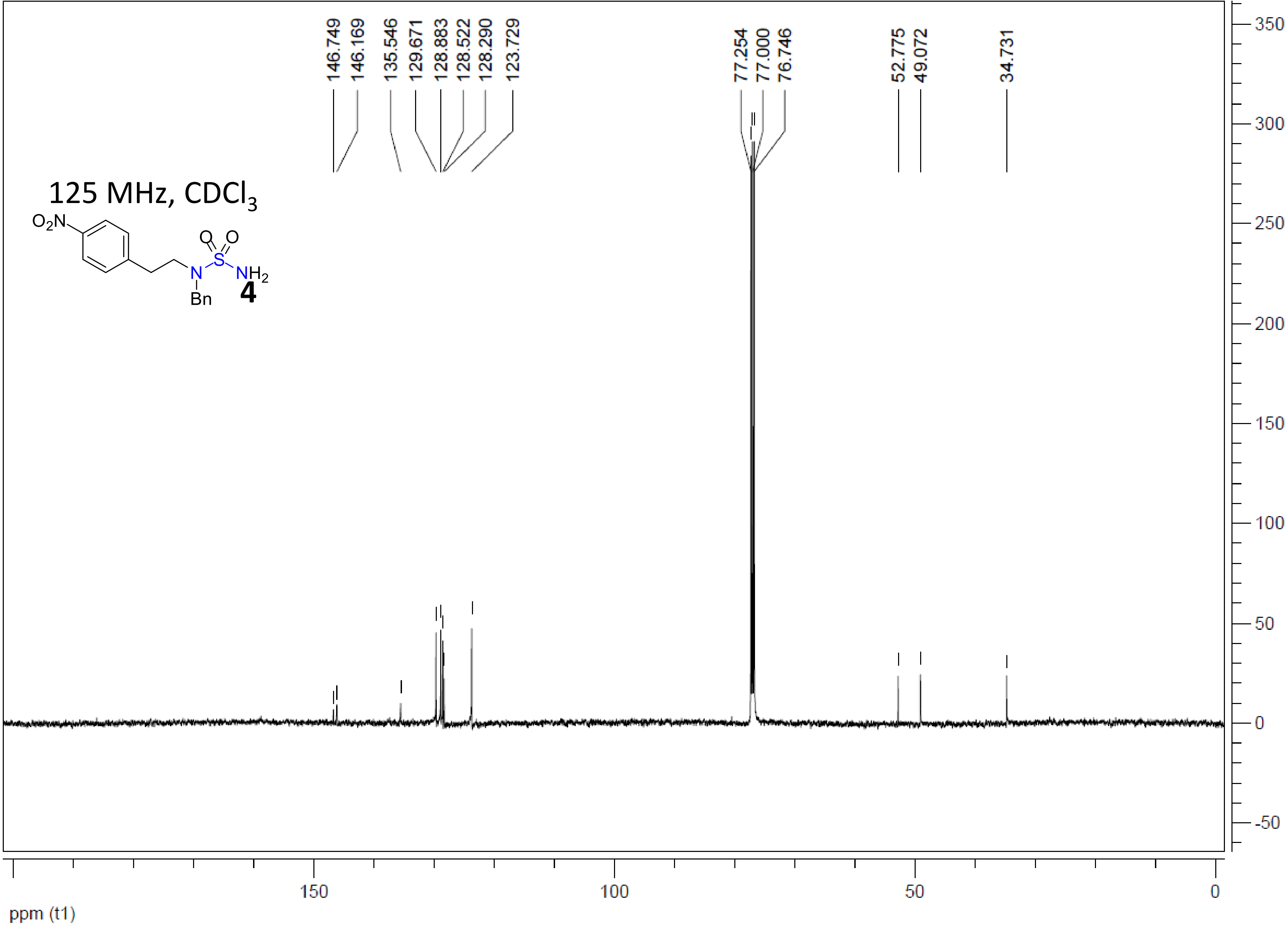

146.749
146.169
135.546
129.671
128.883
128.522
128.290
123.729
77.254
77.000
76.746
52.775
49.072
34.731
125 MHz, CDCl3
O2N
O
O
S
N
NH2
Bn
4
350
300
250
200
150
100
50
0
-50
150
100
50
0
ppm (t1)

400 MHz, $CDCl_3$

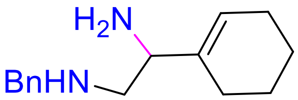

2m’

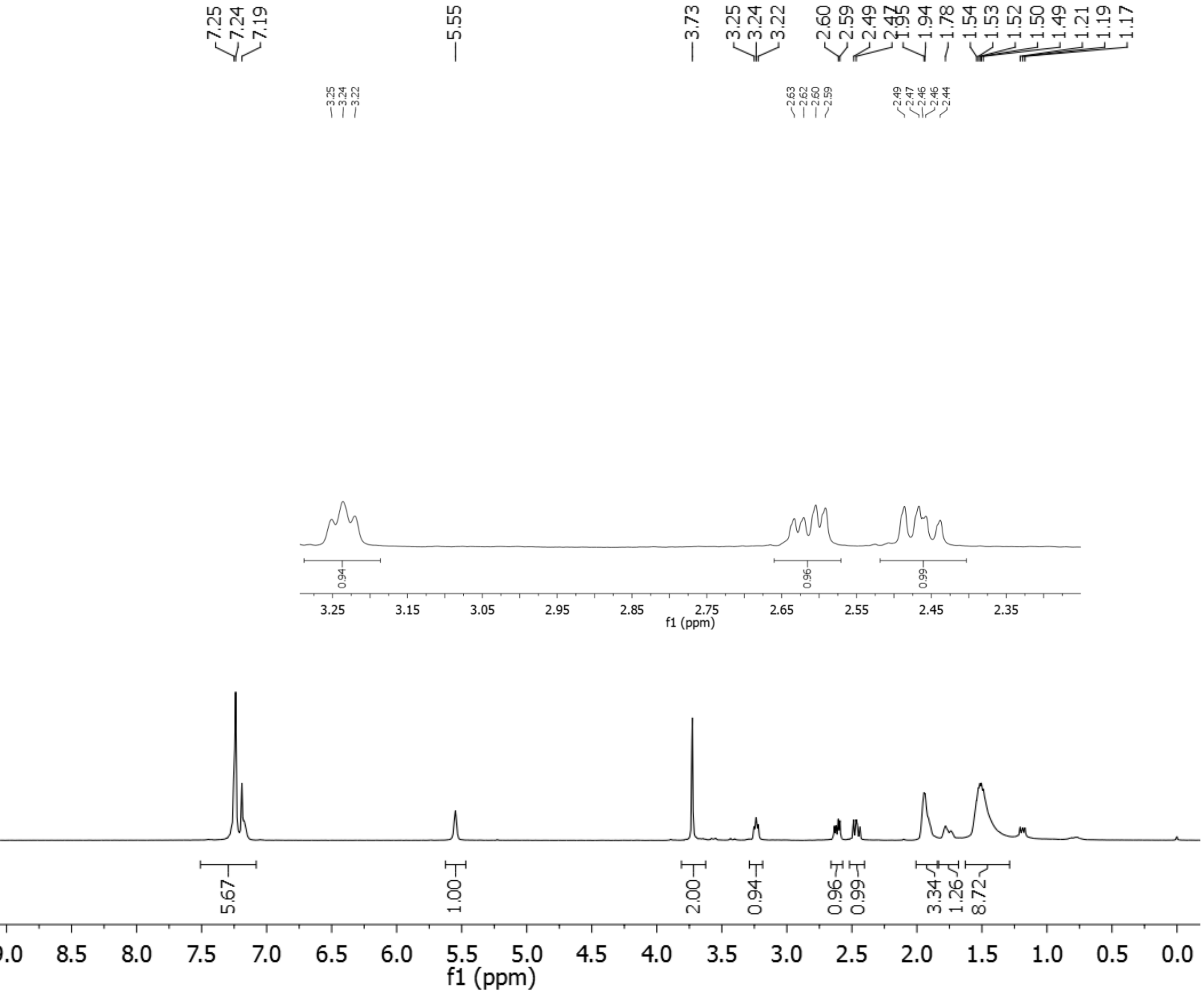

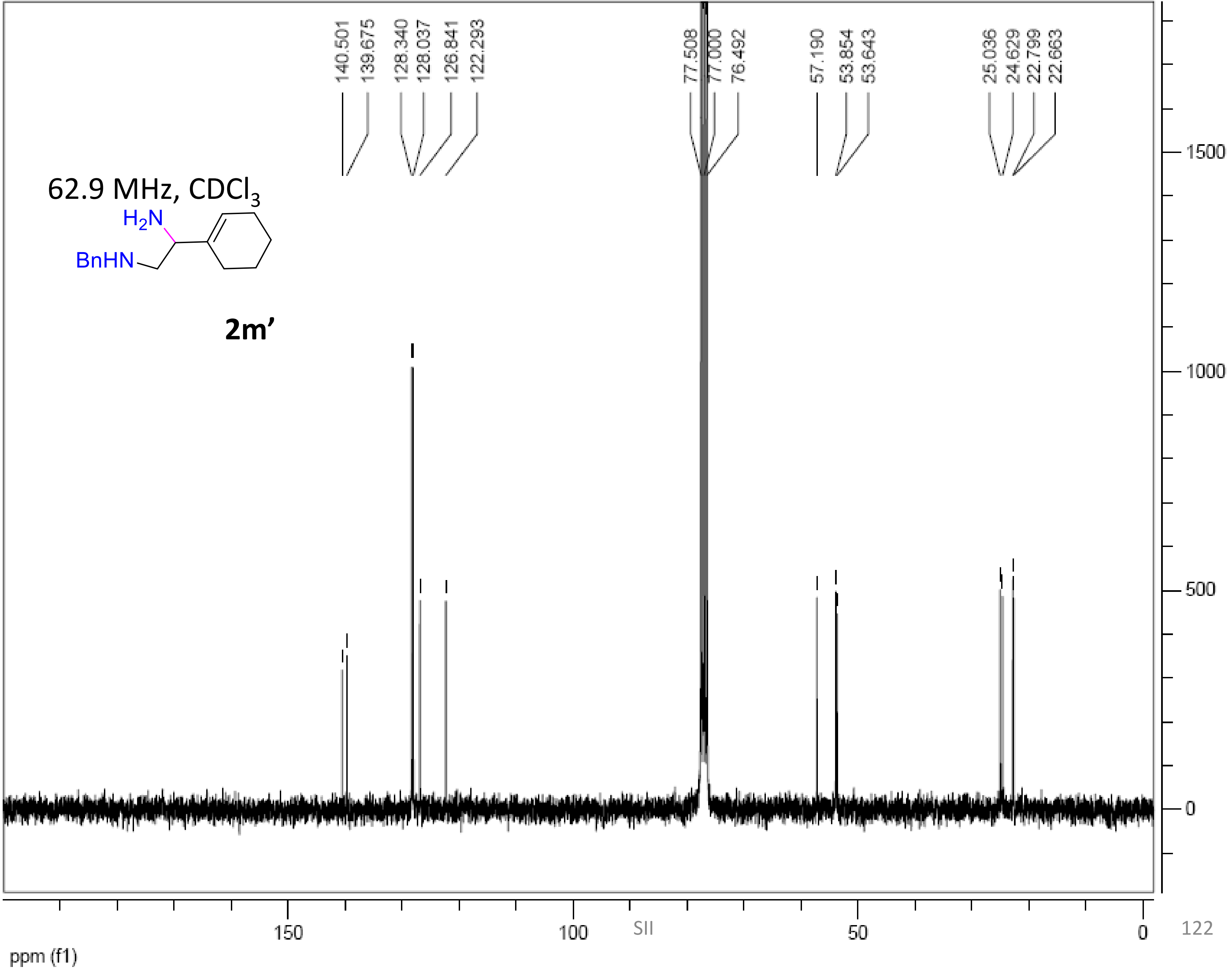
140.501
139.675
128.340
128.037
126.841
122.293
77.508
77.000
76.492
57.190
53.854
53.643
25.036
24.629
22.799
22.663
62.9 MHz, $CDCl_3$
H_2N
BnHN
2m'
1500
1000
500
0
150
100
50
0
ppm (f1)
